# Supplementary material for: Insertion of ammonia into alkenes to build aromatic N-heterocycles
Source: Nat Commun. 2022 Jan 20;13:425. doi: 10.1038/s41467-022-28099-w (PMC8776764; doi:10.1038/s41467-022-28099-w)
Supplement: Supplementary file 1 — Supplementary Information [file 41467_2022_28099_MOESM1_ESM.pdf]

Supplementary Information

Insertion of Ammonia into Alkenes to Build Aromatic *N*-Heterocycles

Shuai Liu[1,2] & Xu Cheng [1,3]*

[1] Institute of Chemistry and Biomedical Sciences, Jiangsu Key Laboratory of Advanced Organic Materials, School of Chemistry and Chemical Engineering, National Demonstration Center for Experimental Chemistry Education, Nanjing University, Nanjing, 210023, China

[2] School of Materials and Chemical Engineering, Xuzhou University of Technology, Xuzhou, 221018, China

[3] State Key Laboratory of Elemento-organic Chemistry, Nankai University, Tianjin, 300071, China

* e-mail: chengxu@nju.edu.cn

Supplementary Note S3
Supplementary Methods...S4
Supplementary References S175

1. Supplementary Note

1.1 General information

All reactions that required anhydrous conditions were carried with standard procedures under argon atmosphere. The solvents were dried by distillation over the appropriate drying reagents. Other chemicals were obtained from commercial sources (J&K, 3A chemicals, Innochem), and were used without further purification. Column chromatography was generally performed on silica gel (300-400 mesh) and reactions were monitored by thin-layer chromatography (TLC) using 254 nm UV light. ^{1}H NMR (400 MHz), ^{13}C NMR (100 MHz) and ^{19}F (376 MHz) were measured on Bruker Avance III 400 spectrometer. Chemical shifts are expressed in parts per million (ppm) with respect to tetramethylsilane. Coupling constants were reported as Hertz (Hz), signal shapes and splitting patterns were indicated as follows: s = singlet; d = doublet; t = triplet; q = quartet; m = multiplet. High-resolution mass spectra (HRMS) were recorded on Agilent mass spectrometer equipped with the ESI or APCI source and a Q-TOF detector. The X-ray analysis of crystal structure was carried out on Bruker SMART 1000 CCD.

2. Supplementary Methods

2.1 Optimization of electrochemical reaction conditions

Supplementary Table 1. Additional optimization of electrochemical reaction

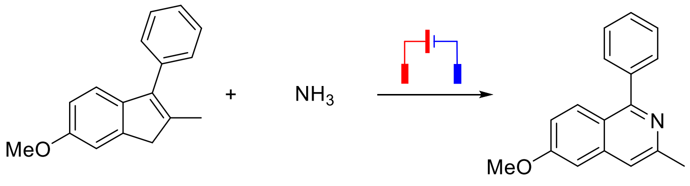

Entry	Solvent		Electrolyte		Yield (%)[g]
1[a]	CH_3OH	GF+ / Pt-	$Mg(ClO_4)_2$	4 V	30
2[a]	CH_3OH	GF+ / GF-	$Mg(ClO_4)_2$	4 V	16
3[a]	CH_3OH	GF+ / Ag-	$Mg(ClO_4)_2$	4 V	52
4[a]	CH_3OH	GF+ / Ag-	$Mg(ClO_4)_2$	3.5 V	50
5[a]	CH_3OH	GF+ / Ag-	$Mg(ClO_4)_2$	4.5 V	52
6[a]	CH_3OH	GF+ / Ag-	$Mg(ClO_4)_2$	5 V	47
7[b]	CH_3OH	GF+ / Ag-	$Mg(ClO_4)_2$	4 V	Trace
8[c]	CH_3OH	GF+ / Ag-	$Mg(ClO_4)_2$	4 V	24
9[d]	CH_3OH	GF+ / Ag-	$Mg(ClO_4)_2$	4 V	55
10[e]	CH_3OH/DCM	GF+ / Ag-	$Mg(ClO_4)_2$	4 V	68(65[h])
11[f]	CH_3OH/DCM	GF+ / Ag-	$Mg(ClO_4)_2$	4 V	64
12[e]	CH_3OH/DCM	GF+ / Ag-	LiCl	4 V	30
13[e]	CH_3OH/DCM	GF+ / Ag-	$LiBF_4$	4 V	67

[a] Reaction conditions: **1a** (0.1 mmol), supporting electrolyte (0.1 mmol), MeOH (5.0 mL), 0 °C, 3 h. [b] -10 °C instead of 0 °C. [c] 50 °C instead of 0 °C. [d] rt instead of 0 °C. [e] **1a** (0.1 mmol), supporting electrolyte (0.1 mmol), MeOH (4.0 mL) and DCM (1.0 mL), rt, 3 h. [f] **1a** (0.1 mmol), supporting electrolyte (0.1 mmol), MeOH (3.0 mL) and DCM (2.0 mL), rt, 3 h. [g] ^{1}H NMR yields. [h] Isolated yields. [i] GF=graphite felt electrodes.

Supplementary Table 2. Optimization of ammonia source

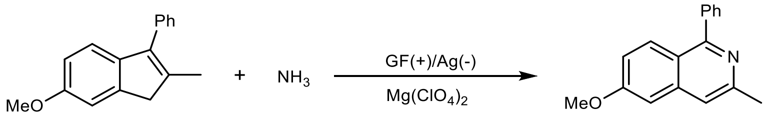

Entry	NH_3 source	Solvent	Yield (%)[b]
1[a]	NH_3 (7 mol/L in MeOH) 0.7 mmol (0.1 mL)	CH_3OH (3.9 mL)+DCM (1.0 mL)	54
2[a]	NH_3 (7 mol/L in MeOH) 1.4 mmol (1 mL)	CH_3OH (3.0 mL)+DCM (1.0 mL)	64

3[a]	NH_3 (7 mol/L in MeOH) 28.0 mmol (4 mL)	DCM (1.0 mL)	67
4	aq. NH_4OH (4 mL)	DCM	10

[a] Reaction conditions: **1a** (0.1 mmol), $Mg(ClO_4)_2$ (0.1 mmol), solvent (5.0 mL), 4.5 V cell potential, rt, Ar, 3 h. [b] ^1H NMR yields.

2.3. General procedures for electrochemical recation of alkenes

2.3.1. General procedure A

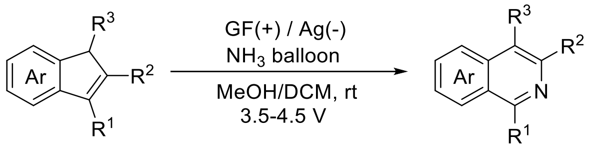

Supplementary Figure 1. General procedure A of ammonia insertion

A 10 mL three-necked heart-shaped flask was charged with the substrate alkene, $Mg(ClO_4)_2$ (0.1 mmol) and a magnetic stir bar. The flask was equipped with a rubber stopper, graphite felt (2 cm x 1 cm x 0.5 cm) as anode and Ag plate (2 cm x 1 cm) as cathode[1]. The flask was evacuated and backfilled with ammonia gas for three times, then an ammonia gas balloon was connected to this flask via a needle. Next, 5 mL of anhydrous solvent was added via syringe. The electrolysis with constant cell potential was carried out at room temperature. After the completion of reaction monitored with TLC and GC-MS, the mixture was concentrated under reduced pressure. The residue was purified by chromatography on silica gel to afford the desired product.

2.3.2. Gram scale reaction

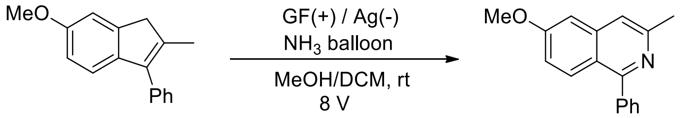

Supplementary Figure 2. Procedure of gram scale reaction.

As that shown in Supplementary Figure 2 and 3, a 400 mL rectangle flask was charged with substrate **1a** (10.0 g, 42 mmol), $LiBF_4$ (0.60 g, 6 mmol) and a magnetic stir bar. The flask was equipped with two pieces of graphite felt (8.5 cm x 6.5 cm x 0.5 cm, 6.5 cm x 6.5 cm x 0.5 cm) as anode (2 pieces) and one piece of silver flake (6.5 cm x 6.5 cm x 0.5 cm) as cathode were installed (as shown below). Two electrodes were separated and fixed with 1.0 cm stick. The graphite felt anode attached to a platinum wire, and cathode attached to a silver wire. The flask was evacuated once and backfilled with NH_3, and 200 mL of anhydrous MeOH and 100 mL of anhydrous DCM were added via syringe. The electrolysis under controlled cell potential (8 V due to the extended distance between anode and cathode) was carried out in a water bath of room temperature. After 14 hours (Supplementary Figure 4–6), the mixture was concentrated under reduced pressure. The residue was purified by chromatography on silica gel to afford the desired product **2a** (6.0 g, 57%).

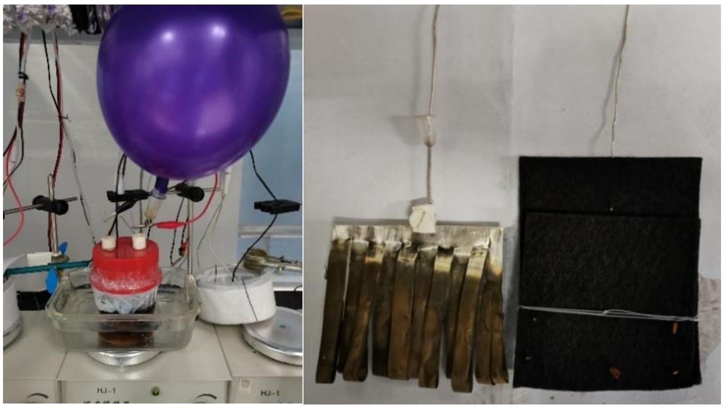

a **b**

Supplementary Figure 3. Reaction at 10-gram scale. **a** the whole reaction. **b** the material connection of electrodes

a

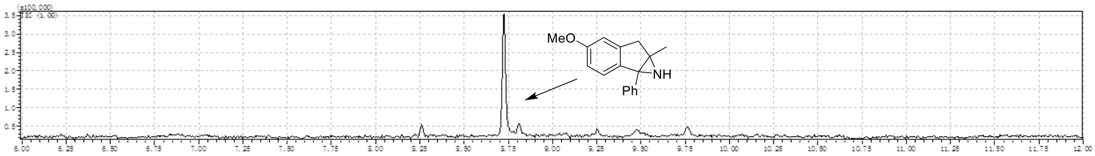

b

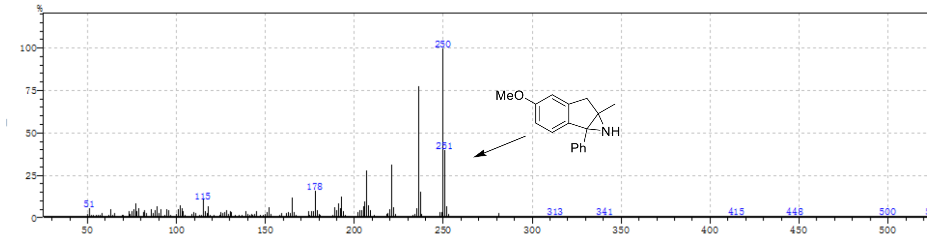

Supplementary Figure 4. Monitored by GC-MS for 4 hours **a** GCMS chromatography. **b** Mass table.

a

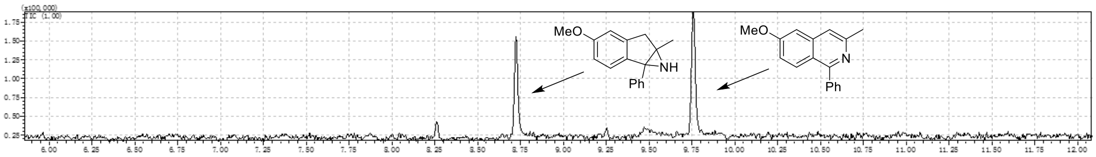

Supplementary Figure 5. Monitored by GC-MS for 8 hours. **a** GCMS chromatography.

a

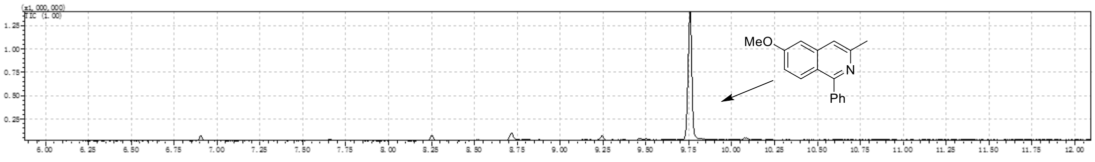

b

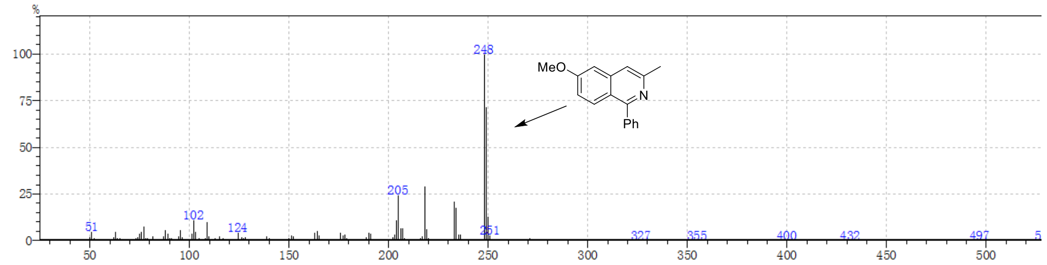

Supplementary Figure 6. Monitored by GC-MS for 14 hours. **a** GCMS chromatography. **b** Mass table.

2.4. Detection and characterization of intermediates 9

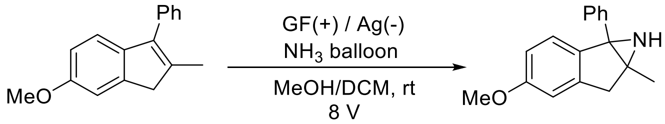

Supplementary Figure 7. Procedure of gram scale reaction.

As that showed in Supplementary Figure 7, a 10 mL three-necked heart-shaped flask was charged with the substrate **1a** (0.103 g, 0.5 mmol), $LiBF_4$ (9.6 mg, 0.1 mmol) and a magnetic stir bar. The flask was equipped with a rubber stopper, graphite felt (2 cm x 1 cm x 0.5 cm) as anode and Ag plate (2 cm x 1 cm) as cathode. The flask was evacuated and backfilled with ammonia gas for three times, then an ammonia gas balloon was connected to this flask via a needle. Next, 2 mL of anhydrous MeOH and 3 mL of anhydrous DCM were added via syringe. The electrolysis with constant cell potential (8.0 V) was carried out at room temperature. After 1.5 hours, the mixture was concentrated under reduced pressure (the bath temperature should be below 30 ^oC). The residue was purified by chromatography on basic silica gel to afford the desired product (12.0 mg, 10%).

Caution: the product was sensitive to acid and high temperature, and even decomposed at -20 ^oC after 7 days.

4-methoxy-6a-methyl-1a-phenyl-1,1a,6,6a-tetrahydroindeno[1,2-*b*]azirine (9)

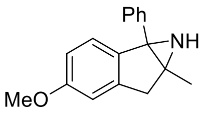

A yellow oil. **^{1}H NMR (400 MHz, Chloroform-*d*)** δ 7.39 – 7.26 (m, 5H), 7.15 (d, J = 8.4 Hz, 1H), 6.87 (s, 2H), 3.87 (s, 3H), 3.35 (q, J = 16.0 Hz, 2H), 1.80 (s, 1H), 1.72 (s, 3H). **^{13}C NMR (100 MHz, Chloroform-*d*)** δ 159.8, 142.5, 141.6, 140.4, 128.2, 127.3(0), 127.3(6), 125.2, 113.4, 110.1, 80.0, 72.3, 55.4, 47.8, 25.7. **HRMS m/z (ESI)** calcd. for $C_{17}H_{18}NO^+$ $(M + H)^+$ 252.1388, found 252.1390. Note: the compounds is not stable enough to get species with high purity. (Supplementary Figure 8,9)

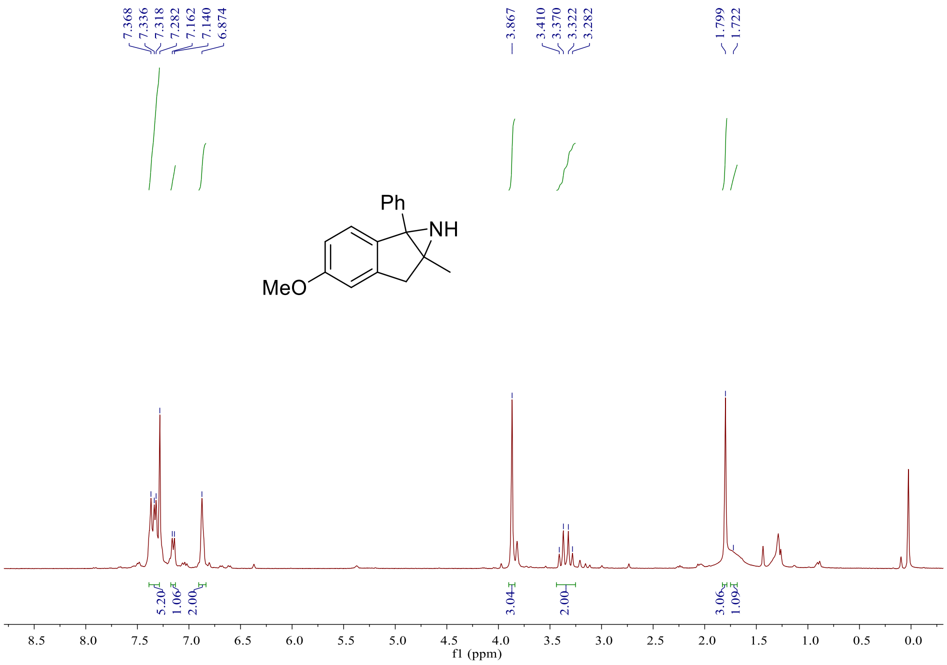

Supplementary Figure 8. ^{1}H NMR (400 MHz, $CDCl_3$) of **9**

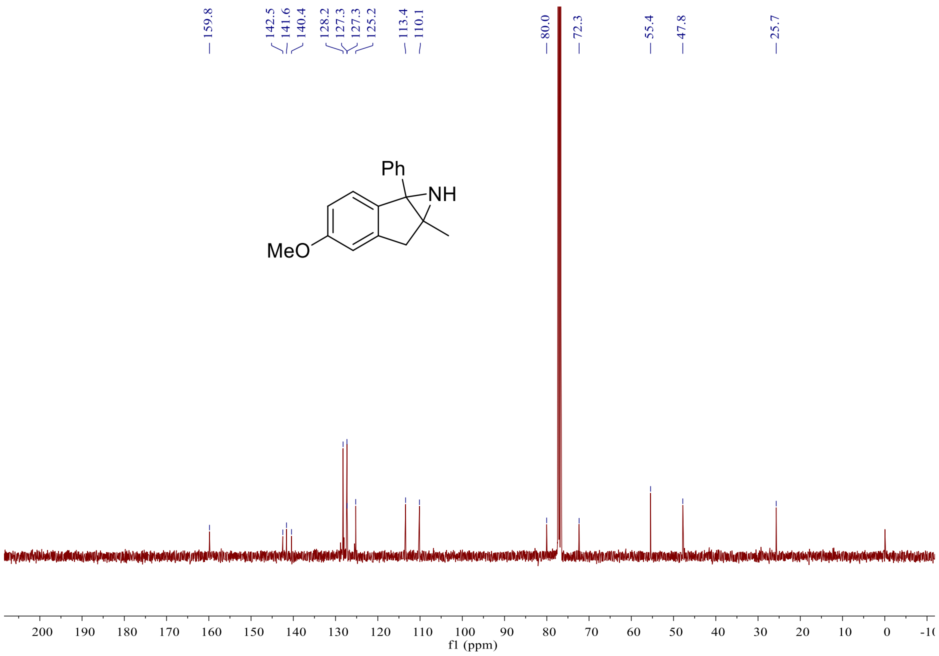

Supplementary Figure 9. ^{13}C NMR (100 MHz, $CDCl_3$) of **9**

2.5. Kinetic study species in reactions

2.5.1. Kinetic study of 9 and 2a

In Jobs Plot of **2a**, Dodecane (15 μL) was used as internal standard in GC-MS analysis of **2a** at different concentrations (0.05 mmol, 0.10 mmol, 0.15 mmol, 0.20 mmol and 0.25 mmol). The linear curve of peak area ratio y (**2a**/dodecane) and concentration x (**2a**) is drawn in Supplementary Figure 10 (y = 26.618x, R^2 = 0.9951).

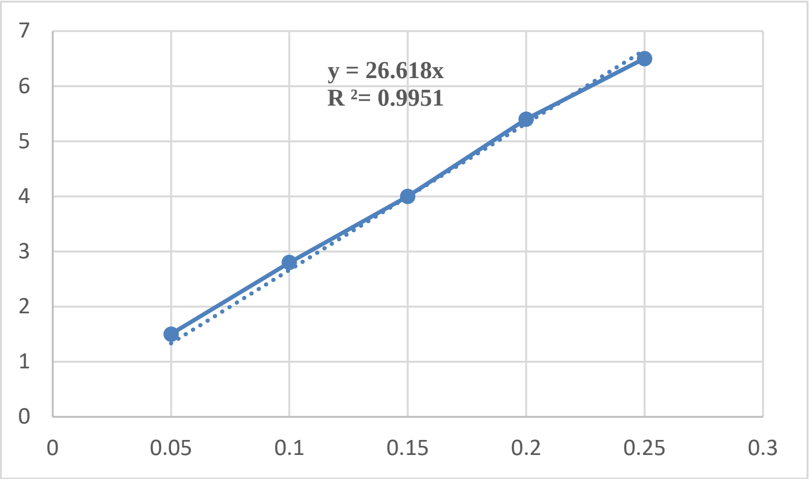

Supplementary Figure 10. The linear curve of peak area ratio y (**2a**/dodecane) and concentration x (**2a**)

Following general procedure A, a 10 mL three-necked heart-shaped flask was charged with the substrate 1a (0.1 mmol), alkene, $Mg(ClO_4)_2$ (0.1 mmol) and a magnetic stir bar. The flask was equipped with a rubber stopper, graphite felt (2 cm x 1 cm x 0.5 cm) as anode and Ag plate (2 cm x 1 cm) as cathode. The flask was evacuated and backfilled with ammonia gas for three times, then an ammonia gas balloon was connected to this flask via a needle. Next, 5 mL of anhydrous solvent and dodecane (15 μL) were added via syringe. The electrolysis with constant cell potential was carried out at room temperature. The reaction was monitored with GC-MS, then the variation of yield (**2a**) along reaction time was shown (Supplementary Figure 11).

a

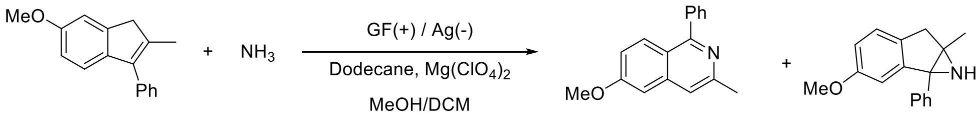

b

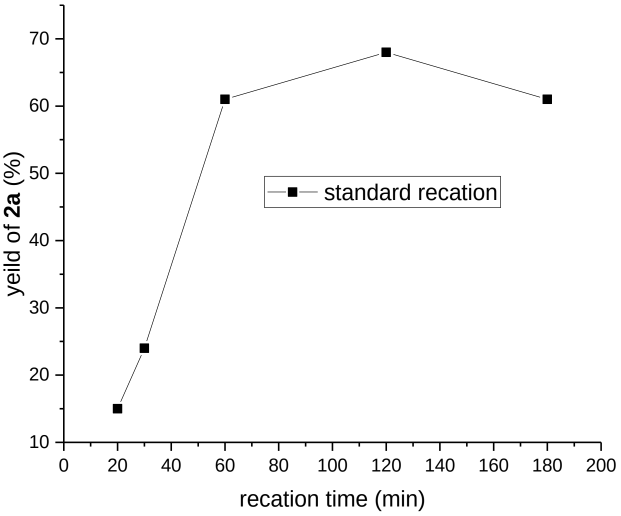

c

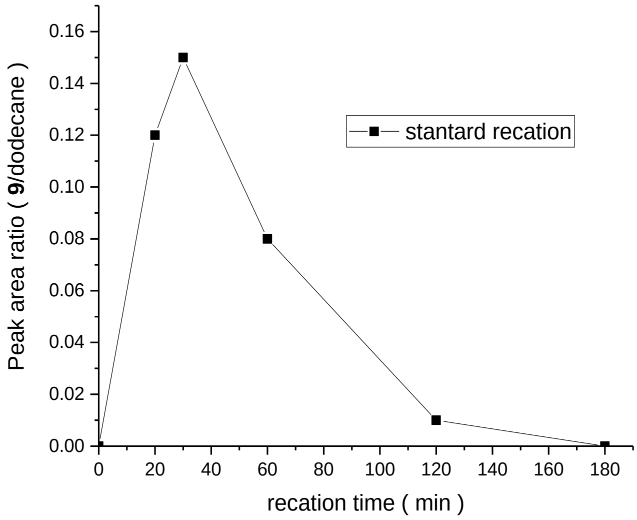

Supplementary Figure 11. Reaction profiles for the conversion of **1a**. **a** The reaction equation. **b** Tracking the yield of **2a**. **c** Tracking conversion of intermediate **9**.

2.5.2. Trapping experiment using radical clocks with different rates

Alkenes **S-1a**[2] 1-(1-cyclopropyl-2-methylprop-1-en-1-yl)-4-methoxybenzene was obtained with reported procedure. **S-1b** were prepared in the Supplementary Figure 12.

To a flask charged with isopropyltriphenylphosphonium iodide (1.30 g, 3 mmol, 1.5 equiv) in 10 mL of anhydrous THF was added *n*-butyllithium (1.6 mL, 2.5 M in hexanes, 2 mmol, 2 equiv) at 0 ℃ under argon. The reaction was then stirred for 0.5 h. (4-methoxyphenyl) (2-phenylcyclopropyl) methanone (0.504 g, 2 mmol, 1 equiv) in anhydrous THF (5 mL) was added dropwise. After completion of addition, the reaction was stirred overnight at room temperature. After being quenched with brine, the mixture was extracted with ethyl acetate for three times. The combined organic layers were washed with water, dried ($MgSO_4$), and concentrated. The residue was purified with flash chromatography to afford the desired product (0.334 g, 60%).

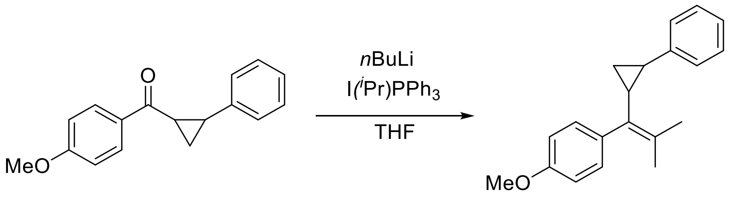

S-1b

Supplementary Figure 12. Synthesis of **S-1b**

1-methoxy-4-(2-methyl-1-(2-phenylcyclopropyl)prop-1-en-1-yl)benzene (S-1b)

The **S-1b** was obtained as a white oil. **^{1}H NMR (400 MHz, Chloroform-*d*)** δ 7.28 (t, *J* = 7.5 Hz, 2H), 7.17 (t, *J* = 7.3 Hz, 1H), 7.12 – 7.07 (m, 2H), 7.02 – 6.96 (m, 2H), 6.94 – 6.84 (m, 2H), 3.86 (s, 3H), 2.16 – 2.03 (m, 1H), 1.93 (s, 3H), 1.73 (dt, *J* = 8.9, 5.2 Hz, 1H), 1.57 (s, 3H), 1.19 – 1.04 (m, 1H), 0.84 (ddd, *J* = 8.7, 6.1, 4.8 Hz, 1H). **^{13}C NMR (100 MHz, Chloroform-*d*)** δ 157.8, 143.3, 135.0, 133.3, 130.8,

129.9, 128.2, 125.8, 125.3, 113.2, 55.2, 27.0, 23.6, 22.7, 20.5, 15.7. **HRMS m/z (ESI)** calcd. for $C_{20}H_{23}O^{+}$ $(M + H)^{+}$ 279.1749, found 279.1748.

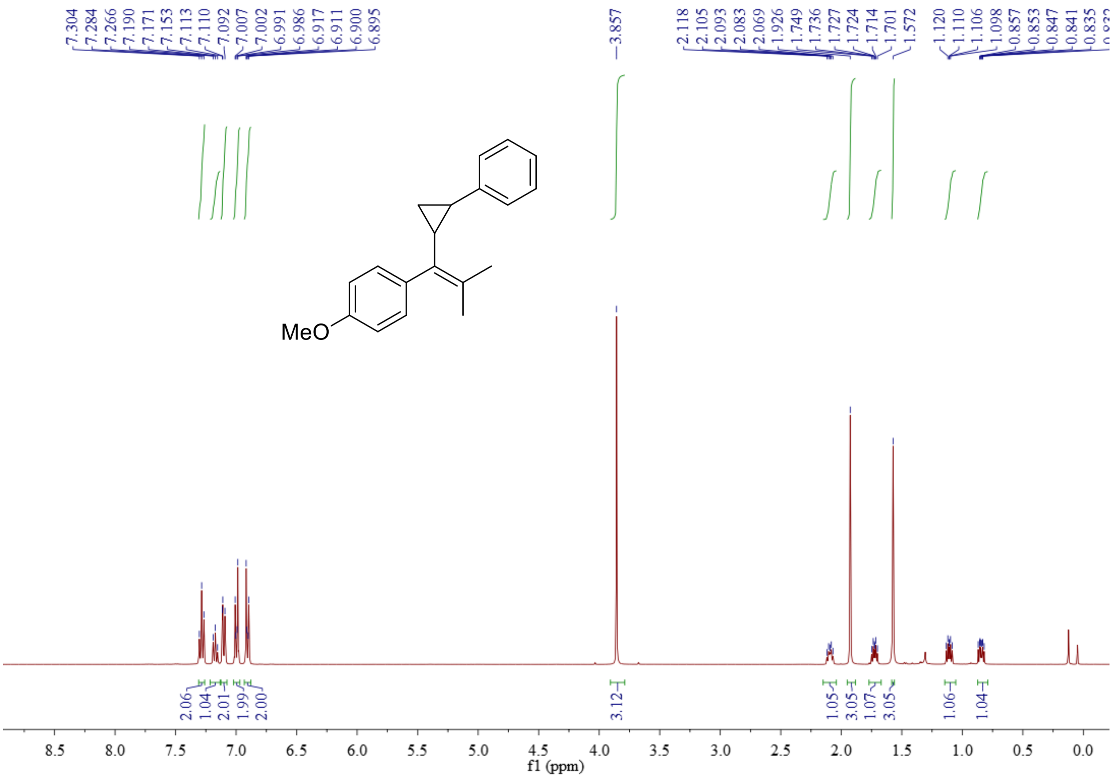

Supplementary Figure 13. ^{1}H NMR (400 MHz, $CDCl_3$) of **S-1b**

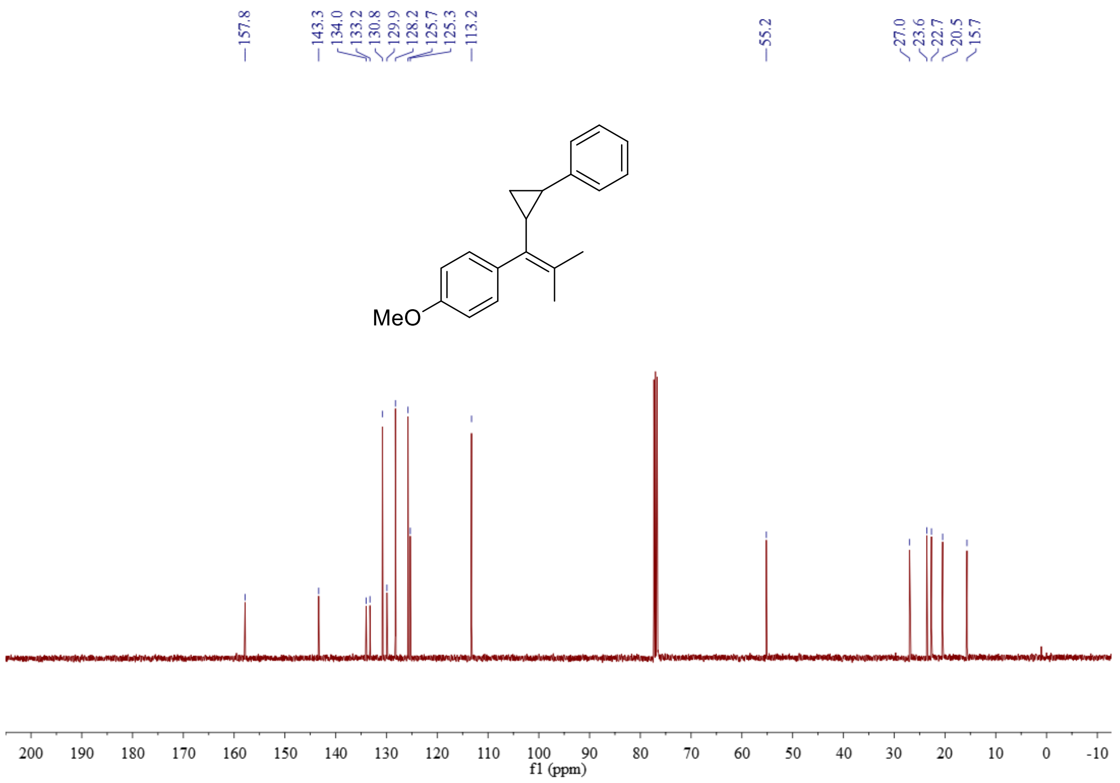

Supplementary Figure 14. ^{13}C NMR (100 MHz, $CDCl_3$) of **S-1b**

These substrates have only alkene as functional group, which would isolate the aziridination step from the overall formation of isoquinoline involving following steps. In substrate **S-1a**, the unsubstituted cyclopropyl group has a ring-opening rate at 6.7 X 10^7 s^{-1}.[3] In substrate **S-1b**, the phenyl-substituted cyclopropyl group has a ring-opening rate at 1.5 X 10^{11} s^{-1}.[4] The electrochemical aziridination using the standard conditions in Fig. 2 were carried out with **S-1a** and **S-1b**. The aziridine **S-2a** was obtained in 54% isolated yield (Supplementary Figure 15a, 16, 17). **S-1b** decomposed during the reaction, giving a complex mixture (Supplementary Figure 15b). The aziridination product was not observed. After attempt, one product was isolate. Though, the exact structure could not be identified, the cyclopropyl group disappeared as that was shown by ^1H NMR (Supplementary Figure 18,19). These results suggested the radical species is highly transient and unsubstituted cyclopropyl group was kept during the transformation.

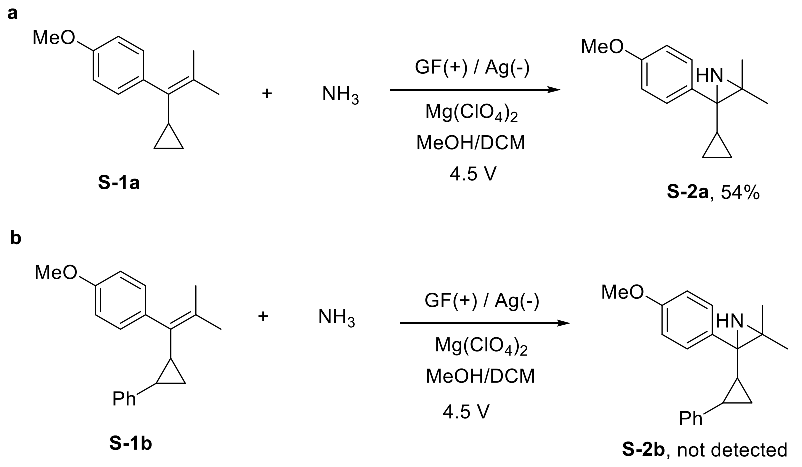

Supplementary Figure 15. Radical clock experiments **a** Reaction using S-1a as substrate. **b** Reaction using S-1b as substrate.

2-cyclopropyl-2-(4-methoxyphenyl)-3,3-dimethylaziridine (S-2a)

Product **S-2a** was obtained as a white liquid in 54% yield (23.4 mg). **^{1}H NMR (400 MHz, Chloroform-*d*)** δ 7.14 (d, J = 8.7 Hz, 2H), 6.85 (d, J = 8.7 Hz, 2H), 3.80 (s, 3H), 1.48 (s, 3H), 1.31 – 1.15 (m, 1H), 0.93 (s, 3H), 0.83 (s, 1H), 0.56 – 0.40 (m, 2H), 0.38 – 0.31 (m, 1H), 0.18 – 0.10 (m, 1H). **^{13}C NMR (100 MHz, Chloroform-*d*)** δ 158.1, 134.4, 129.0, 113.3, 55.2, 50.3, 41.6, 24.7, 21.4, 16.0, 4.7, 3.1. **HRMS m/z (ESI)** calcd. for $C_{14}H_{20}NO^{+}$ $(M + H)^{+}$ 218.1545, found 218.1549.

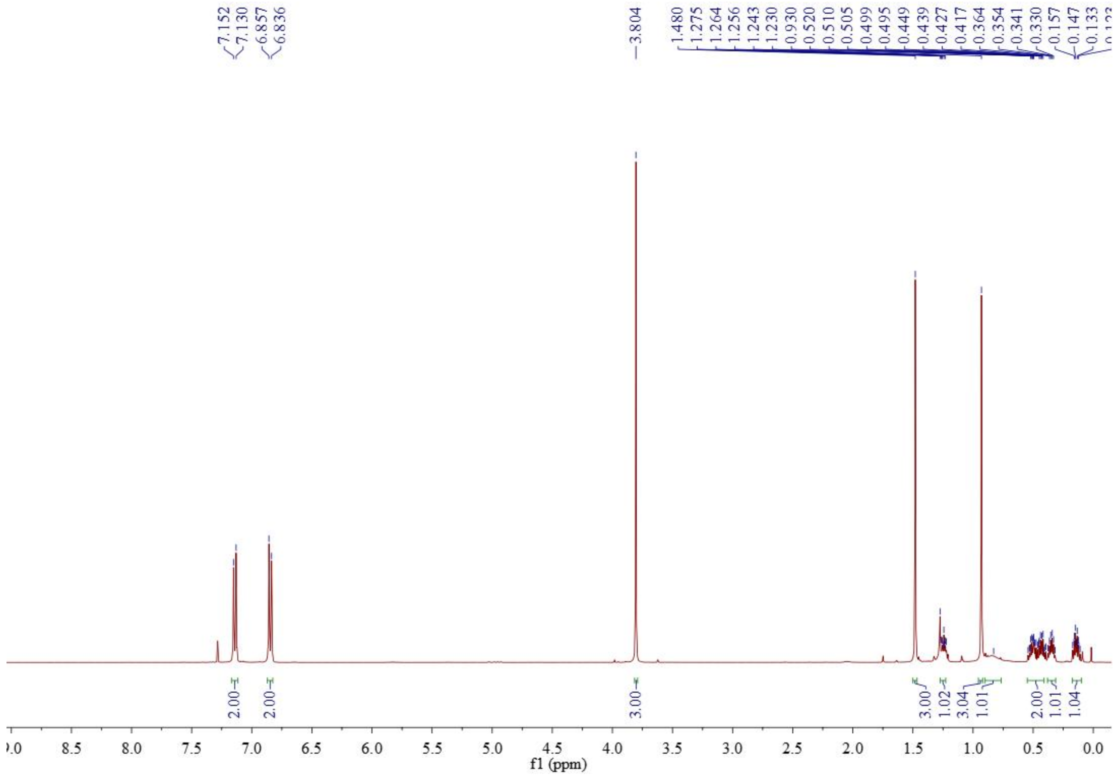

Supplementary Figure 16. ^{1}H NMR (400 MHz, $CDCl_3$) of **S-2a**

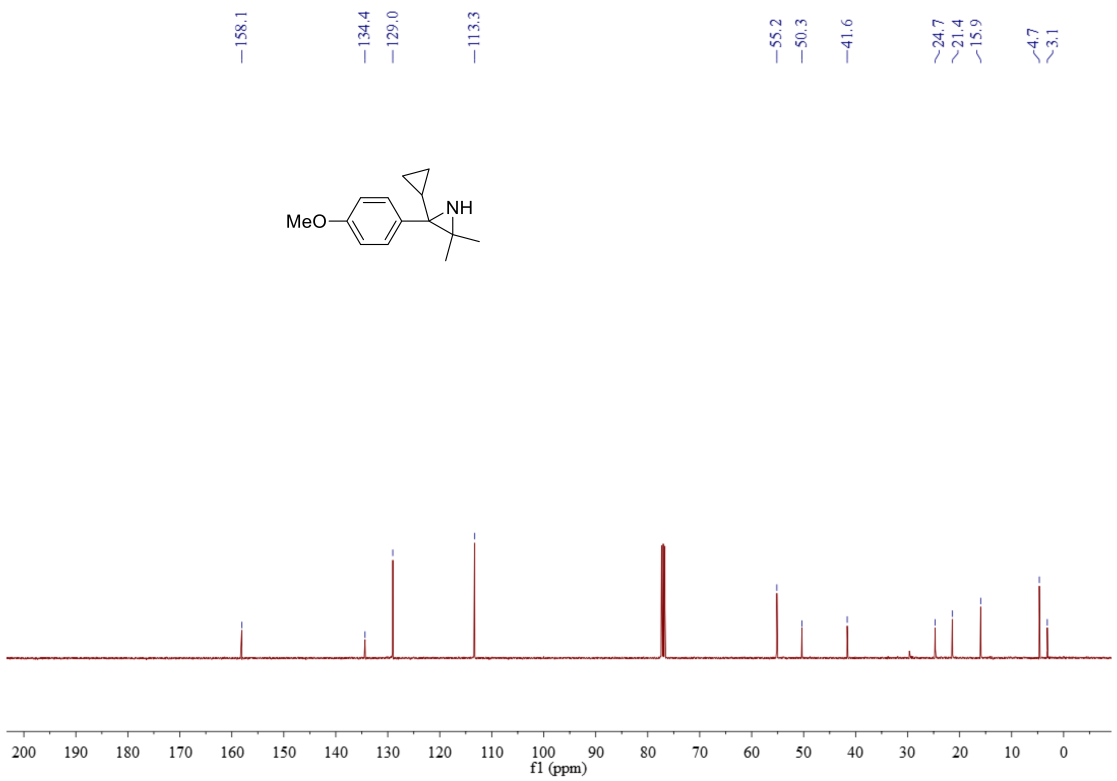

Supplementary Figure 17. ^{13}C NMR (100 MHz, $CDCl_3$) of **S-2a**

Unidentified product isolated from electrochemical reaction using S-1b

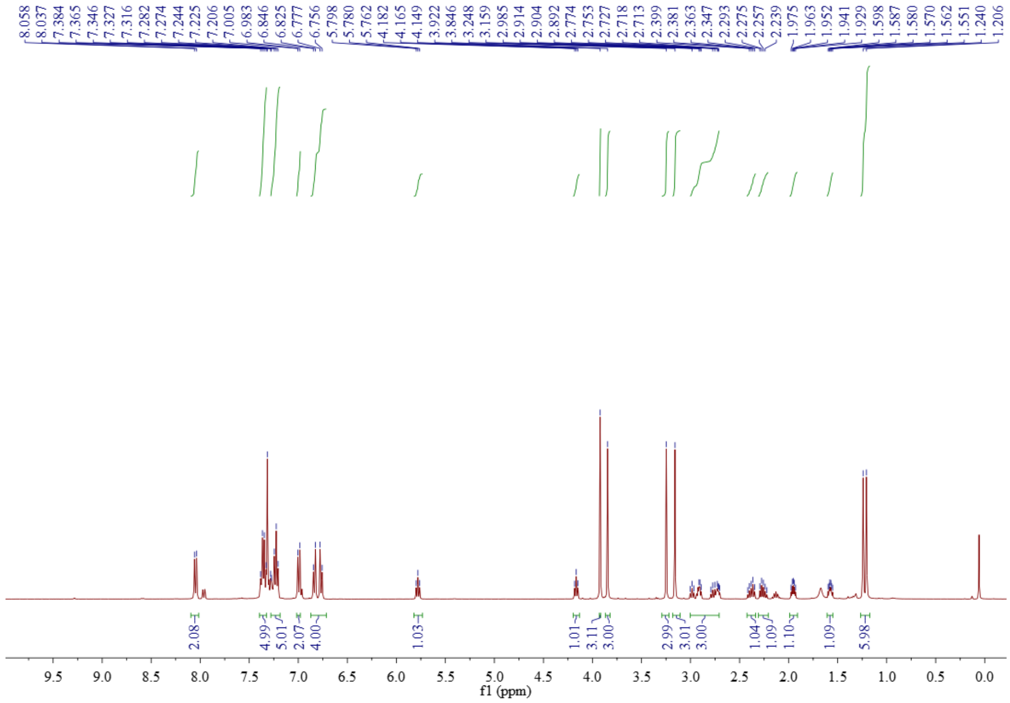

Supplementary Figure 18. ^{1}H NMR (400 MHz, $CDCl_3$) of unidentified product

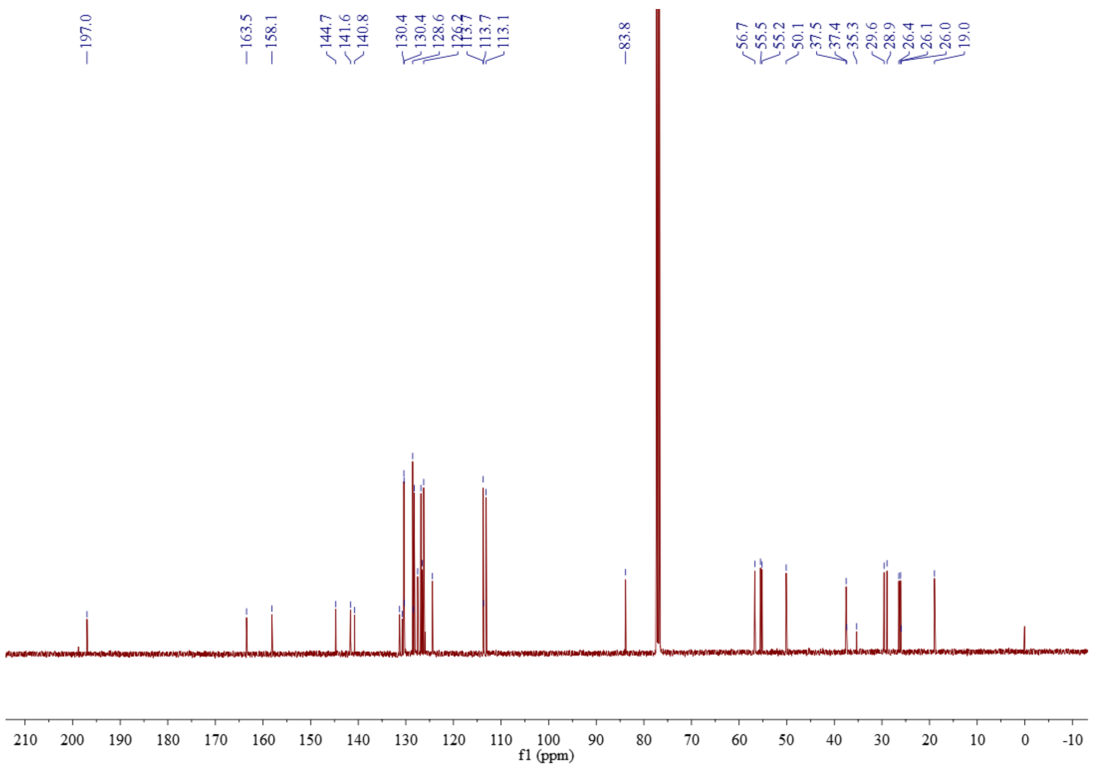

Supplementary Figure 19. ^{13}C NMR (100 MHz, $CDCl_3$) of unidentified product

2.5.3. Competition of alkenes

To test if the reaction proceeds via a nitrene intermediate, competition reactions employing two substrates in one cell were conducted. It was found the desired isoquinoline was obtained as normal reaction, and aziridine of other alkenes was not observed (Supplementary Figure 20).

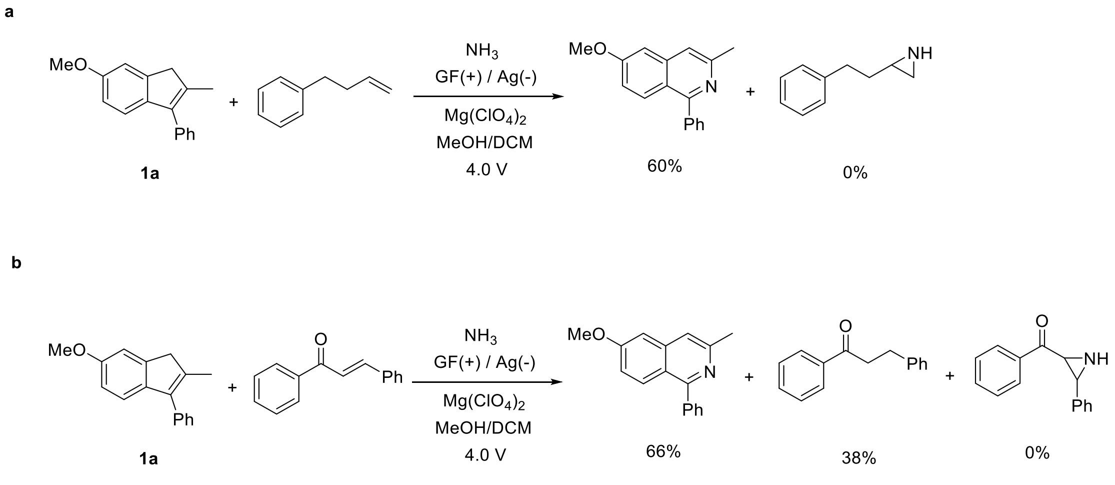

Supplementary Figure 20. Competition reaction using different alkenes. **a** Competition between **1a** and terminal alkene. **b** Competition between **1a** and electron-deficient alkene.

2.6. Cyclic voltammetry experiments of reactants

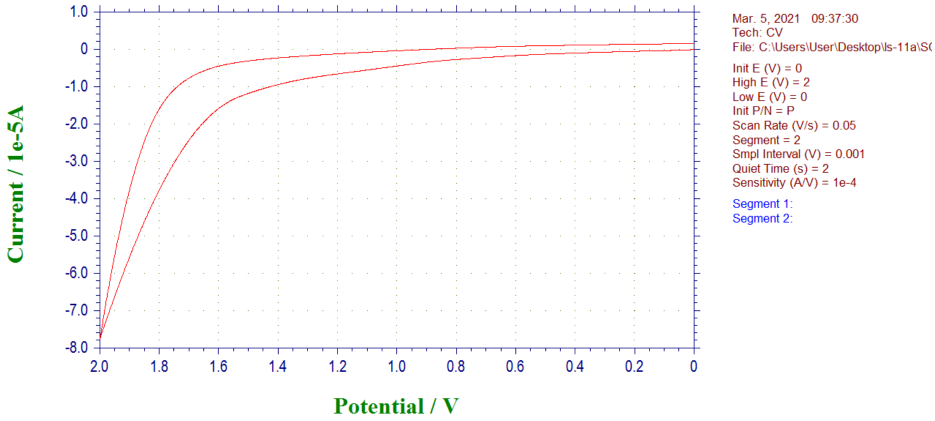

Supplementary Figure 21. Cyclic voltammograms of MeOH/DCM

A solution of $Mg(ClO_4)_2$ (0.1 mmol) in 4 mL MeOH and 1 mL DCM was subject to the cyclic voltammetry experiment. Electrodes included a 4.0 mm glassy carbon working electrode, a gauze platinum counter electrode and a saturated calomel reference electrode (SCE) via a salt bridge charged with a solution of $Mg(ClO_4)_2$ (0.02 M in MeOH/DCM). Potential sweep rate was 50 mV/s (Supplementary Figure 21).

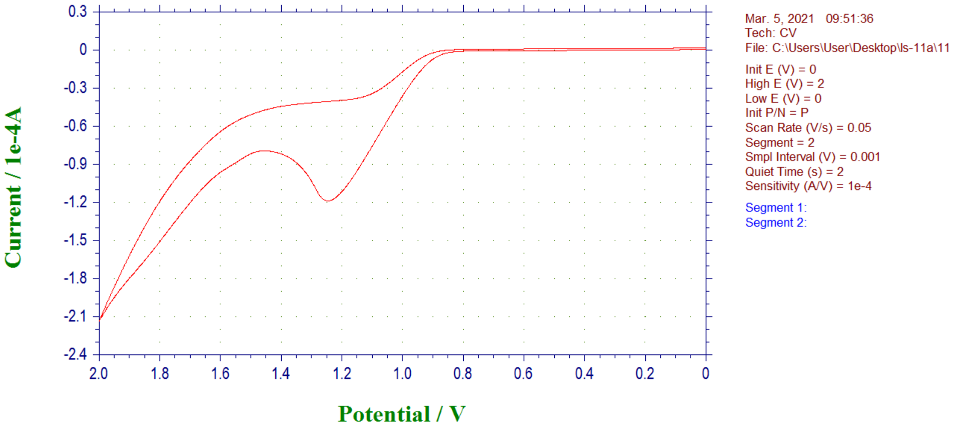

Supplementary Figure 22. Cyclic voltammograms of **1a** in MeOH/DCM

A solution of **1a** (0.05 mmol) and $Mg(ClO_4)_2$ (0.1 mmol) in 4 mL MeOH and 1 mL DCM was subject to the cyclic voltammetry experiment. Electrodes included a 4.0 mm glassy carbon working electrode, a gauze platinum counter electrode and a saturated calomel reference electrode (SCE) via a salt bridge charged with a solution of $Mg(ClO_4)_2$ (0.02 M in MeOH/DCM). Potential sweep rate was 50 mV/s (Supplementary Figure 22).

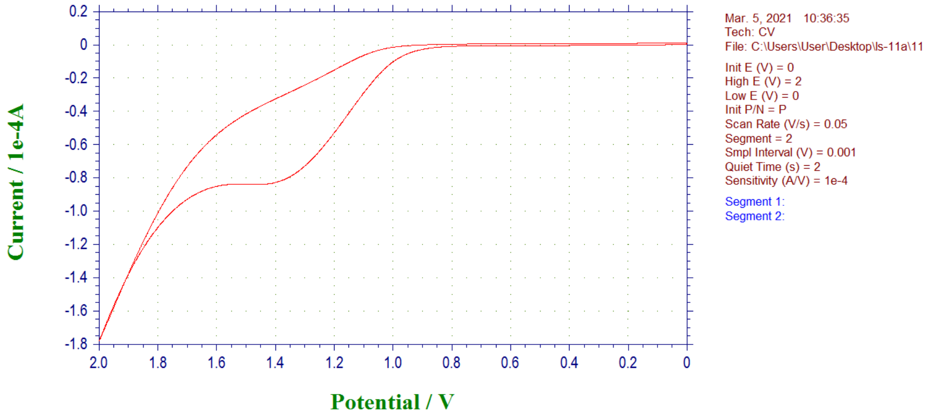

Supplementary Figure 23. Cyclic voltammograms of **1a** and 0.1 mmol NH_3 in MeOH/DCM

A solution of **1a** (0.05 mmol), 0.032 mL NH_3 (3.1 mol/L in MeOH) and $Mg(ClO_4)_2$ (0.1 mmol) in 4 mL MeOH and 1 mL DCM was subject to the cyclic voltammetry experiment. Electrodes included a 4.0 mm glassy carbon working electrode, a gauze platinum counter electrode and a saturated calomel reference electrode (SCE) via a salt bridge charged with a solution of $Mg(ClO_4)_2$ (0.02 M in MeOH/DCM). Potential sweep rate was 50 mV/s (Supplementary Figure 23).

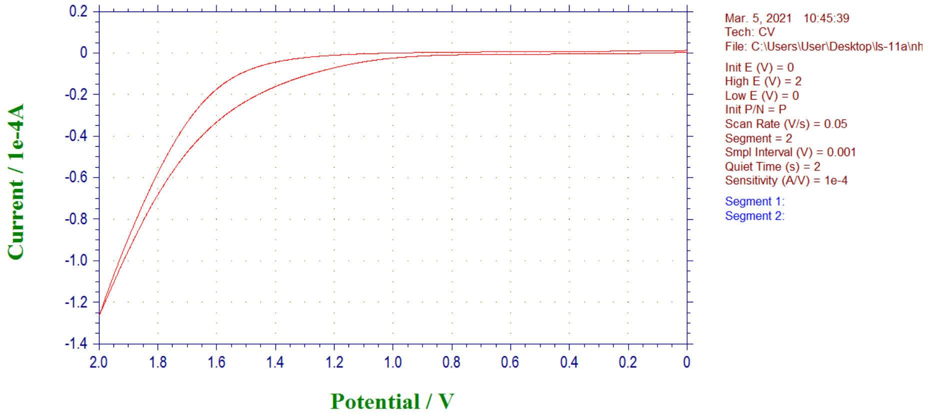

Supplementary Figure 24. Cyclic voltammograms of NH_3 in MeOH/DCM

A solution of 0.032 mL NH_3 (3.1mol/L in MeOH) and $Mg(ClO_4)_2$ (0.1 mmol) in 4 mL MeOH and 1 mL DCM was subject to the cyclic voltammetry experiment. Electrodes included a 4.0 mm glassy carbon working electrode, a gauze platinum counter electrode and a saturated calomel reference electrode (SCE) via a salt bridge charged with a solution of $Mg(ClO_4)_2$ (0.02 M in MeOH). Potential sweep rate was 50 mV/s (Supplementary Figure 24).

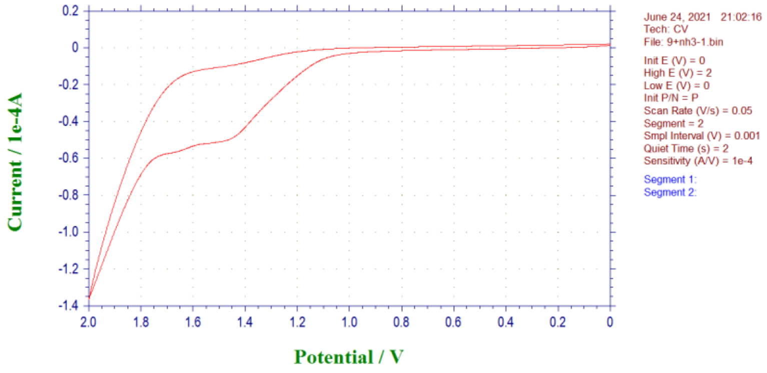

Supplementary Figure 25. Cyclic voltammograms of **9** and NH_3 in MeOH/DCM

A solution of **9** (0.05 mmol) 0.037 mL NH_3 (2.7 mol/L in MeOH) and $Mg(ClO_4)_2$ (0.1 mmol) in 4 mL MeOH and 1 mL DCM was subject to the cyclic voltammetry experiment. Electrodes included a 4.0 mm glassy carbon working electrode, a gauze platinum counter electrode and a saturated calomel reference electrode (SCE) via a salt bridge charged with a solution of $Mg(ClO_4)_2$ (0.02 M in MeOH). Potential sweep rate was 50 mV/s (Supplementary Figure 25).

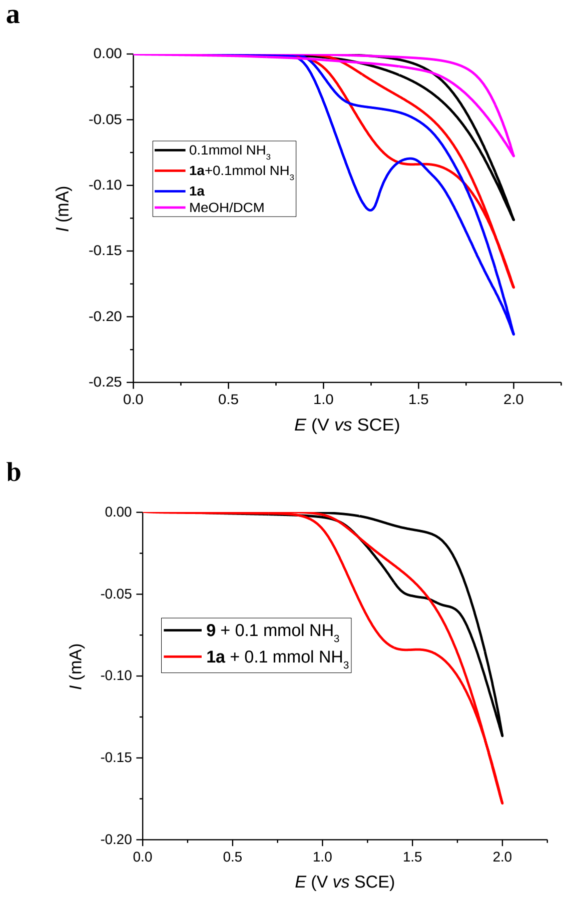

Supplementary Figure26. Overlapped CV results in MeOH/DCM. a Overlapped CVs of reactants. **b** Overlapped CVs of **9** and **1a**.

2.7. Measurement of charge consumption

The reaction was setup with a general procedure A equipped with a CHI730E bipotentiostat as power source with bulk electrolysis method (Supplementary Figure 27). Charge consumption was calculated as M*n*F/ Q, Q was read from the experiment as item "Total Q", M was the molar of **2a** obtained in the reaction (1*10^{-3} mol), "n" was the number of electrons corresponding to the generation of one molecule of product, and F was Faraday's constant: 96485. Faradaic efficiency FE = 0.2*65%*10^{-3}*4*96485/137.96 = 36%).

a

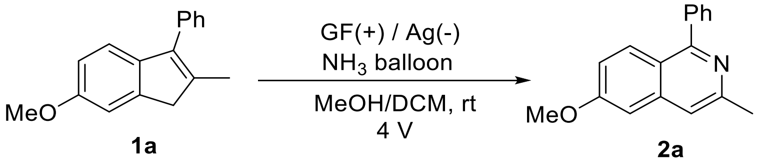

b

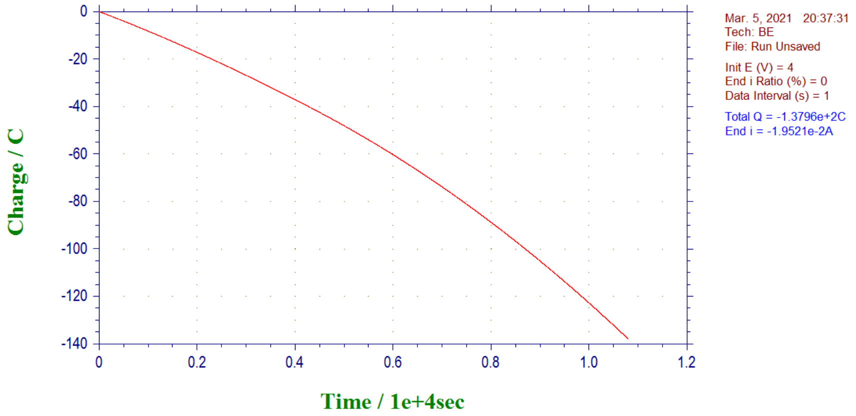

Supplementary Figure 27. Bulk electrolysis (BE) experiment of 1a. **a** **1a** (0.2 mmol), $Mg(ClO_4)_2$ (0.1 mmol) in 4 mL of anhydrous MeOH and 1 mL of anhydrous DCM, graphite felt electrode and silver electrode, ammonia balloon, rt, 4 V, 3 h. ^{1}H NMR yield Y= 65%. **b** The BE plot of the reaction.

2.8. Synthetic and analytical data of olefins

1a-1l, 1n-1t, 1al, 1au, 7 were prepared according to the known procedure.[5-7] **1m** was prepared according to the following procedure B. **1u** was prepared according to the following procedure C. **1v-1x, 1z** were prepared according to the following procedure D. **1y** was prepared according to the following procedure E. **1aa** was prepared according to the following procedure F. **1ab-1ad, 1ag-1ai** were prepared according to the following procedure G. **1ae, 1af** were prepared according to the following procedure H. **1aj** was prepared according to the following procedure I. **1ak** was prepared according to the known procedure.[8] **1am-1ap** were prepared according to the known procedure.[9] **1av** was prepared according to the known procedure[10]. The detailed operation steps are shown below.

General Procedure B

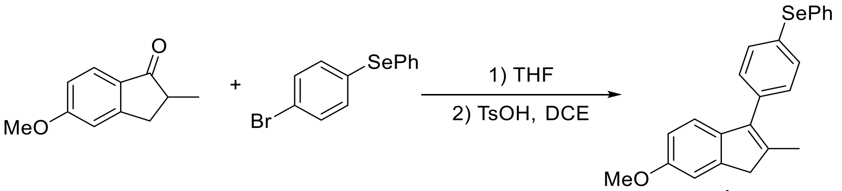

1m

Supplementary Figure 28. General Procedure B to prepare substrate

As that shown in Supplementary Figure 28, to a solution of (4-bromophenyl)(phenyl)selane (0.372 g, 1.5 mmol) in anhydrous THF (10 mL) at −78 °C was added a solution of ^{n}BuLi (1.6 M in THF, 1 mL, 1.6 mmol, 1.1 equiv) dropwise under argon. The reaction mixture was stirred at −78 °C for 30 min, Then 5-methoxy-2-methyl-2,3-dihydro-1H-inden-1-one (2.880 g, 24 mmol, 1.1 equiv) dissolved by THF (2 mL) was added dropwise. After completion of addition, the reaction mixture was brought to room temperature and stirred overnight. The reaction mixture was quenched with saturated aqueous NH_4Cl solution and extracted with ethyl acetate for three times. The combined organic layers were washed with water, dried over $MgSO_4$, filtered, and concentrated in vacuo. The resulting liquid was dissolved in 1,2-dichloroethane (10 mL). TsOH (5.0 mg) was added and the mixture was refluxed overnight. After cooling to room temperature, the reaction mixture was concentrated and purified by flash column chromatography to afford the desired product **1m** (0.118 g, 30%).

(4-(6-methoxy-2-methyl-1*H*-inden-3-yl)phenyl)(phenyl)selane (1m)

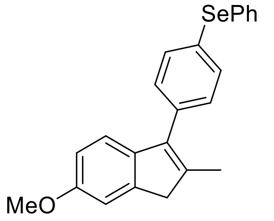

The substrate **1m** was obtained as a white oil. **^{1}H NMR (400 MHz, Chloroform-*d*)** δ 7.56 (d, *J* = 8.1 Hz, 4H), 7.40 – 7.30 (m, 5H), 7.19 – 7.01 (m, 2H), 6.88 – 6.60 (m, 1H), 3.85 (s, 3H), 3.44 (s, 2H), 2.13 (s, 3H). **^{13}C NMR (100 MHz, Chloroform-*d*)** δ 157.5, 144.1, 139.2, 138.6, 137.3, 134.8, 133.3, 132.7, 131.0, 130.0, 129.6, 129.4, 127.8, 127.5, 119.4, 111.5, 110.3, 55.6, 43.1, 14.8. **HRMS m/z (ESI)** calcd. for $C_{23}H_{21}OSe^{+}$ $(M + H)^{+}$ 393.0758, found 393.0748.

General Procedure C

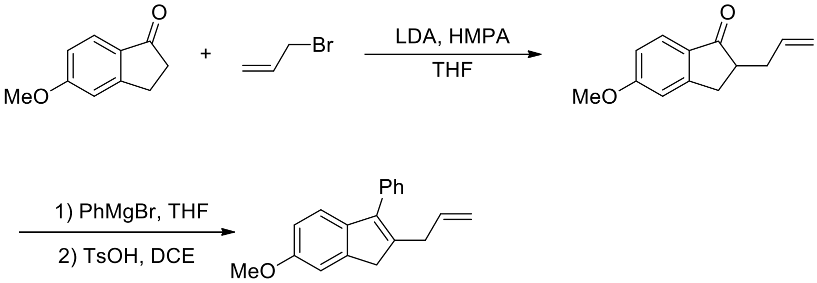

Supplementary Figure 29. General Procedure C to prepare substrate

As that shown in Supplementary Figure 29, to a solution of 5-methoxy-2,3-dihydro-1*H*-inden-1-one (3.564 g, 22 mmol) in anhydrous THF (40 mL) at −78 °C was added a solution of LDA (2 M in THF, 12 mL, 24 mmol, 1.1 equiv) dropwise under argon. The reaction mixture was stirred at −78 °C for 1 h, Then 3-bromoprop-1-ene (2.880 g, 24 mmol, 1.1 equiv) and HMPA (7.876 g, 44 mmol, 2.0 equiv) was

added dropwise. After completion of addition, the reaction mixture was brought to room temperature and stirred overnight. The reaction mixture was quenched with saturated aqueous NH_4Cl solution and extracted with ethyl acetate for three times. The combined organic layers were washed with water, dried ($MgSO_4$), filtered, and concentrated. The residue was purified by flash column chromatography to afford the white liquid.

To a solution of the above white liquid in anhydrous THF (40 mL) was added PhMgBr (24 mL, 1.0 M in THF, 24 mmol, 1.1 equiv) at 0 ℃ under argon. After completion of addition, the reaction mixture was brought to room temperature. After the completion of reaction monitored with TLC and GC-MS, the mixture was quenched with saturated aqueous NH_4Cl solution and extracted with ethyl acetate for three times. The combined organic layers were washed with water, dried over $MgSO_4$, filtered, and concentrated in vacuo. The resulting liquid was dissolved in 1,2-dichloroethane (20 mL). TsOH (20.0 mg) was added and the mixture was refluxed overnight. After cooling to room temperature, the reaction mixture was concentrated and purified by flash column chromatography to afford the desired product (1.800 g, overall yield: 31%).

2-allyl-6-methoxy-3-phenyl-1*H*-indene (1u)

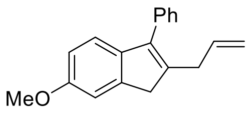

The substrate **1u** was obtained as a white solid. mp: 64 – 65 °C. **^{1}H NMR (400 MHz, Chloroform-*d*)** δ 7.55 – 7.35 (m, 5H), 7.17 (d, *J* = 8.3 Hz, 1H), 7.10 (d, *J* = 2.3 Hz, 1H), 6.83 (dd, *J* = 8.4, 2.4 Hz, 1H), 5.96 (ddt, *J* = 16.6, 10.1, 6.4 Hz, 1H), 5.30 – 4.87 (m, 2H), 3.86 (s, 3H), 3.48 (s, 2H), 3.25 (d, *J* = 6.4 Hz, 2H). **^{13}C NMR (100 MHz, Chloroform-*d*)** δ 157.7, 144.5, 139.8, 139.3, 139.0, 137.0, 135.4, 129.0, 128.4, 127.2, 120.0, 115.7, 111.6, 110.4, 55.6, 40.7, 33.6. **HRMS m/z (ESI)** calcd. for $C_{19}H_{19}O^+$ $(M + H)^+$ 263.1436, found 263.1427.

General Procedure D

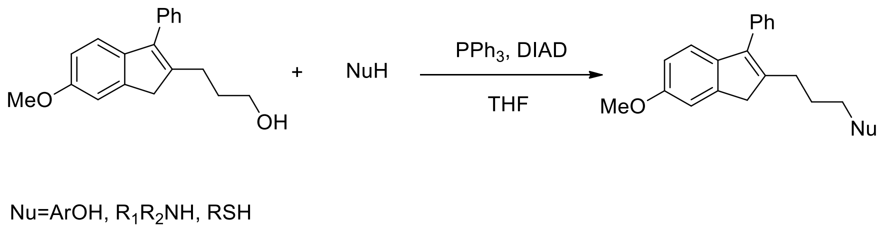

Supplementary Figure 30. General Procedure D to prepare substrate

As that shown in Supplementary Figure 30, 3-(6-methoxy-3-phenyl-1*H*-inden-2-yl)propan-1-ol (0.280 g, 1.0 mmol), NuH (1 mmol, 1.0 equiv) and Ph_3P (0.393 g, 1.5 mmol, 1.5 eq) were dissolved in THF (10 ml). The solution was stirred for 0.5 h under argon. To this solution, DIAD (0.303 g, 1.5 mmol, 1.5 equiv) was added gradually at 0 °C. The reaction mixture was stirred overnight at room temperature. The mixture was concentrated, and purified with flash chromatography to afford the desired product.

3-(3-(6-methoxy-3-phenyl-1*H*-inden-2-yl)propoxy)-13-methyl-6,7,8,9,11,12,13,14,15,16-decahydro-17*H*-cyclopenta[a]phenanthren-17-one (1v)

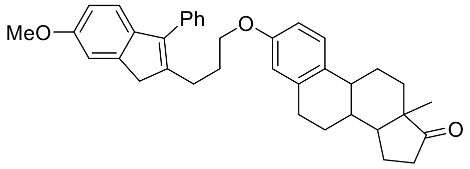

Following the general **procedure D** on 1.0 mmol scale the substrate **1v** was obtained as a white solid in 47% yield (0.250 g). mp: 162 – 163 °C. **^{1}H NMR (400 MHz, Chloroform-*d*)** δ 7.52 – 7.34 (m, 5H), 7.20 (d, *J* = 8.6 Hz, 1H), 7.10 (d, *J* = 2.9 Hz, 2H), 6.82 (dd, *J* = 8.3, 2.4 Hz, 1H), 6.67 (dd, *J* = 8.6, 2.6 Hz, 1H), 6.59 (d, *J* = 2.5 Hz, 1H), 3.93 (t, *J* = 6.4 Hz, 2H), 3.86 (s, 3H), 3.51 (s, 2H), 2.90 (dd, *J* = 8.6, 5.3 Hz, 2H), 2.76 – 2.65 (m, 2H), 2.53 (dd, *J* = 18.8, 8.5 Hz, 1H), 2.42 (d, *J* = 9.5 Hz, 1H), 2.27 (t, *J* = 8.5 Hz, 1H), 2.23 – 1.90 (m, 6H), 1.77 – 1.38 (m, 6H), 0.94 (s, 3H). **^{13}C NMR (100 MHz, Chloroform-*d*)** δ 221.0, 157.6, 156.9, 144.1, 141.4, 139.5, 139.0, 137.6, 135.6, 133.0, 129.1, 128.4, 127.1, 126.3, 119.8, 114.5, 112.2, 111.6, 110.4, 67.3, 55.7, 50.4, 48.0, 44.0, 40.5, 38.4, 35.9, 31.6, 29.7, 29.5, 26.6, 25.9, 25.4, 21.6, 13.9. **HRMS m/z (ESI)** calcd. for $C_{37}H_{40}ONa^+$ $(M + Na)^+$ 555.2875, found 555.2861.

5-((3-(6-methoxy-3-phenyl-1*H*-inden-2-yl)propyl)thio)-1-phenyl-1*H*-tetrazole (1w)

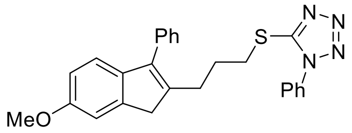

Following the general **procedure D** on 1.0 mmol scale the substrate **1w** was obtained as a colorless liquid in 64% yield (0.281 g). **^{1}H NMR (400 MHz, Chloroform-*d*)** δ 7.55 (d, *J* = 6.2 Hz, 5H), 7.48 – 7.40 (m, 2H), 7.36 (d, *J* = 6.8 Hz, 3H), 7.15 – 7.03 (m, 2H), 6.82 (dd, *J* = 8.3, 2.0 Hz, 1H), 3.85 (s, 3H), 3.49 (s, 2H), 3.34 (t, *J* = 7.2 Hz, 2H), 2.69 (t, *J* = 7.4 Hz, 2H), 2.13 (p, *J* = 7.3 Hz, 2H). **^{13}C NMR (100 MHz, Chloroform-*d*)** δ 157.8, 154.2, 144.1, 140.2, 139.8, 139.3, 135.4, 133.7, 130.0, 129.7, 129.0, 128.5, 127.2, 123.8, 119.9, 111.8, 110.4, 55.7, 40.3, 32.8, 29.1, 27.6. **HRMS m/z (ESI)** calcd. for $C_{26}H_{25}N_4OS^+$ $(M + H)^+$ 441.1749, found 441.1734.

***tert*-butyl (3-(6-methoxy-3-phenyl-1*H*-inden-2-yl)propyl)(tosyl)carbamate (1x)**

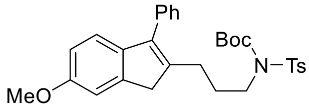

Following the general **procedure D** on 0.5 mmol scale the substrate **1x** was obtained as a red liquid in 44% yield (0.116 g). **^{1}H NMR (400 MHz, Chloroform-*d*)** δ 7.77 (d, *J* = 8.2 Hz, 2H), 7.48 (t, *J* = 7.5 Hz, 2H), 7.39 (d, *J* = 7.5 Hz, 3H), 7.30 (d, *J* = 8.1 Hz, 2H), 7.11 (d, *J* = 8.3 Hz, 2H), 6.82 (dd, *J* = 8.3, 2.1 Hz, 1H), 3.86 (s, 3H), 3.84 – 3.76 (m, 2H), 3.54 (s, 2H), 2.58 (t, *J* = 7.8 Hz, 2H), 2.45 (s, 3H), 2.18 – 1.95 (m, 2H), 1.33 (s, 9H). **^{13}C NMR (100 MHz, Chloroform-*d*)** δ 157.6, 150.9, 144.2, 144.1, 141.0, 139.4, 138.9, 137.4, 135.5, 129.2, 129.0, 128.5, 127.8, 127.1, 119.8, 111.7, 110.4, 84.1, 55.6, 47.0, 40.3, 30.2(2), 30.2(8), 27.9, 26.9, 26.2, 21.6. **HRMS m/z (ESI)** calcd. for $C_{31}H_{36}NO_5S^+$ $(M + H)^+$ 534.2314, found 534.2296.

7-(3-(6-methoxy-3-phenyl-1*H*-inden-2-yl)propyl)-7*H*-purine (1z)

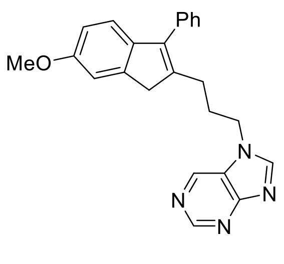

Following the general **procedure D** on 1.0 mmol scale the substrate **1z** was obtained as a colorless liquid in 73% yield (0.278 g). **^{1}H NMR (400 MHz, Chloroform-*d*)** δ 9.12 (s, 1H), 8.97 (s, 1H), 7.66 (s, 1H), 7.50 – 7.35 (m, 3H), 7.33 (dd, *J* = 8.0, 1.4 Hz, 2H), 7.16 – 7.02 (m, 2H), 6.81 (dd, *J* = 8.3, 2.4 Hz, 1H), 4.20 (t, *J* = 7.0 Hz, 2H), 3.84 (s, 3H), 3.48 (s, 2H), 2.58 (t, *J* = 7.4 Hz, 2H), 2.29 – 2.11 (m, 2H). **^{13}C NMR (100 MHz, Chloroform-*d*)** δ 157.8, 152.5, 151.3, 148.6, 145.2, 143.9, 139.9, 139.5, 139.0, 135.2, 134.1,

128.9, 128.7, 127.5, 120.1, 111.8, 110.4, 55.6, 43.4, 40.3, 29.6, 25.7. **HRMS m/z (ESI)** calcd. for $C_{24}H_{23}N_4O^+$ (M + H)$^+$ 383.1872, found 383.1879.

General Procedure E

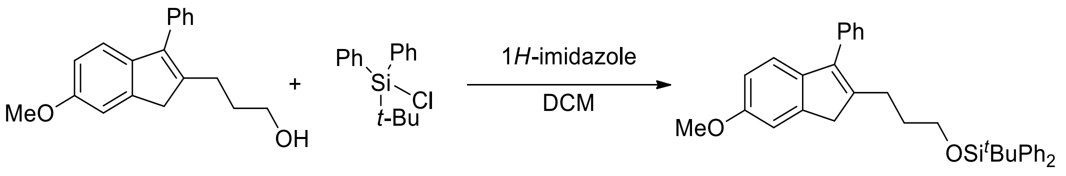

Supplementary Figure 31. General Procedure E to prepare substrate

As that shown in Supplementary Figure 31, a solution of 3-(6-methoxy-3-phenyl-1*H*-inden-2-yl)propan-1-ol (0.560 g, 2.0 mmol), *tert*-butylchlorodiphenylsilane (0.822 g, 3.0 mmol, 1.5 equiv), 1*H*-imidazole (0.204 g, 3.0 mmol, 1.5 equiv) in DCM (10 mL) was stirred at room temperature overnight. The reaction mixture was then quenched with saturated aqueous $NaHCO_3$ solution and extracted with DCM for three times. The organic layer was dried ($MgSO_4$), filtered, and concentrated. The residue was purified by flash chromatography on silica gel to afford the desired product.

***tert*-butyl(3-(6-methoxy-3-phenyl-1*H*-inden-2-yl)propoxy)diphenylsilane (1y)**

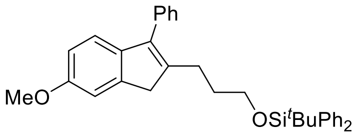

The substrate **1y** was obtained as a white solid in 66% yield (0.170 g). mp: 79 – 80 °C. **^{1}H NMR (400 MHz, Chloroform-*d*)** δ 7.67 (dd, *J* = 7.9, 1.4 Hz, 4H), 7.53 – 7.33 (m, 11H), 7.17 – 7.07 (m, 2H), 6.83 (dd, *J* = 8.3, 2.4 Hz, 1H), 3.87 (s, 3H), 3.71 (t, *J* = 6.3 Hz, 2H), 3.43 (s, 2H), 2.85 – 2.53 (m, 2H), 1.93 – 1.75 (m, 2H), 1.06 (s, 9H). **^{13}C NMR (100 MHz, Chloroform-*d*)** δ 157.5, 144.2, 142.3, 139.6, 138.3, 135.7, 135.6, 134.0, 129.6, 129.0, 128.4, 127.6, 126.9, 119.7, 111.5, 110.4, 63.7, 55.7, 40.5, 33.0, 26.9, 25.4, 19.2. **HRMS m/z (ESI)** calcd. for $C_{35}H_{39}O_2Si^+$ (M + H)$^+$ 519.2719, found 519.2701.

General Procedure F

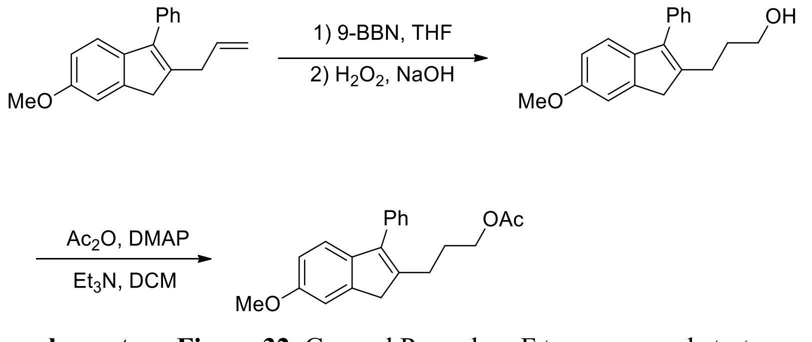

Supplementary Figure 32. General Procedure F to prepare substrate

As that shown in Supplementary Figure 32, To a flask charged with 2-allyl-6-methoxy-3-phenyl-1*H*-indene (1.310 g, 5 mmol) in anhydrous THF (5 mL) was added 9-BBN (20 mL, 0.5 M in THF, 10 mmol, 2 equiv) at 0 °C under argon. The reaction was allowed to warm to 50 °C spontaneously, and then was stirred for 2.0 h. H_2O_2 (5.0 mL) and NaOH (2 M, 5.0 mL) were added dropwise at 0 °C. After completion of addition, the reaction was allowed to being stirred overnight at room temperature. Then

the mixture was extracted with ethyl acetate for three times. The combined organic layers were washed with water, dried ($MgSO_4$), filtered, and concentrated. The residue was purified with flash chromatography to afford the desired product (1.12g, 80%).

Acetic anhydride (0.122 g, 1.2mmol, 1.2 equiv) was added dropwise to a solution of 3-(6-methoxy-3-phenyl-1*H*-inden-2-yl)propan-1-ol (0.280 g, 1 mmol), DMAP (12.2 mg, 0.1 mmol, 0.1 equiv) and triethylamine (0.152 g, 1.5 mmol, 1.5 equiv) in anhydrous CH_2Cl_2 (10 mL) at 0 ℃. The solution was stirred for 12 hours at room temperature. The reaction mixture was concentrated and extracted with ethyl acetate and saturated aqueous NH_4Cl. The organic layer was washed with water, dried ($MgSO_4$), filtered, and concentrated. The residue was purified by column chromatography to afford the desired product (0.275 g, 85%).

3-(6-methoxy-3-phenyl-1*H*-inden-2-yl)propyl acetate (1aa)

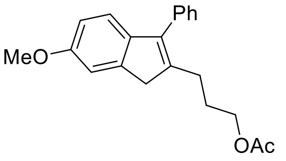

The substrate **1aa** was obtained as a colorless liquid. **^{1}H NMR (400 MHz, Chloroform-*d*)** δ 7.53 – 7.46 (m, 2H), 7.41 (d, *J* = 7.1 Hz, 3H), 7.16 – 7.08 (m, 2H), 6.84 (dd, *J* = 8.3, 2.4 Hz, 1H), 4.07 (t, *J* = 6.6 Hz, 2H), 3.87 (s, 3H), 3.49 (s, 2H), 2.77 – 2.49 (m, 2H), 1.99 (s, 3H), 1.97 – 1.85 (m, 2H). **^{13}C NMR (100 MHz, Chloroform-*d*)** δ 171.1, 157.7, 144.1, 140.9, 139.4, 139.3, 135.5, 129.1, 128.5, 127.1, 119.9, 111.7, 110.4, 63.9, 55.6, 40.3, 28.7, 25.2, 20.9. **HRMS m/z (ESI)** calcd. for $C_{21}H_{23}O^+$ $(M + H)^+$ 323.1647, found 323.1641.

General Procedure G

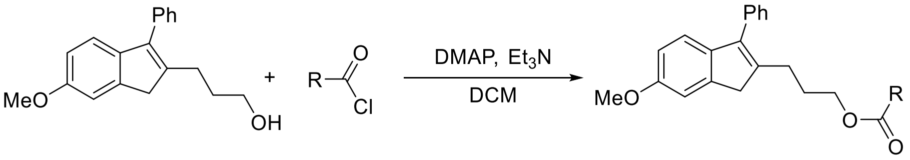

Supplementary Figure 33. General Procedure G to prepare substrate

As that shown in Supplementary Figure 33, to a flask containing 3-(6-methoxy-3-phenyl-1*H*-inden-2-yl)propan-1-ol (0.560 g, 2 mmol), DMAP (0.024 g, 0.2 mmol, 0.01 equiv) and chloride (3.0 mmol, 1.5 equiv) in 10 mL of anhydrous DCM was added a solution of Et_3N (0.303 g, 3.0 mmol, 1.5 equiv) in 5 mL anhydrous DCM at 0 ℃ under argon. The reaction was warmed slowly to room temperature overnight. The reaction mixture was then quenched with saturated aqueous $NaHCO_3$ solution and extracted with DCM for three times. The organic layer was dried ($MgSO_4$), filtered, and concentrated. The residue was purified by flash chromatography on silica gel to afford the desired product.

3-(6-methoxy-3-phenyl-1*H*-inden-2-yl)propyl cyclopropanecarboxylate (1ab)

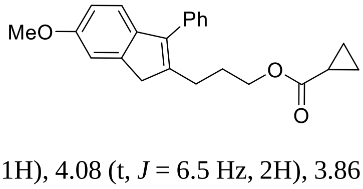

Following the general **procedure G** on 1.0 mmol scale the substrate **1ab** was obtained as a colorless liquid in 75% yield (0.261 g). **^{1}H NMR (400 MHz, Chloroform-*d*)** δ 7.49 (t, *J* = 7.5 Hz, 2H), 7.40 (d, *J* = 7.1 Hz, 3H), 7.12 (d, *J* = 8.9 Hz, 2H), 6.83 (dd, *J* = 8.3, 1.8 Hz, 1H), 4.08 (t, *J* = 6.5 Hz, 2H), 3.86 (s, 3H), 3.49 (s, 2H), 2.60 (t, *J* = 7.7 Hz, 2H), 1.93 (p, *J* = 6.8 Hz, 2H), 1.55 (tt, *J* = 8.5, 4.7 Hz, 1H), 0.96 (p, *J* = 4.2 Hz, 2H), 0.84 (dq, *J* = 7.6, 4.0 Hz, 2H). **^{13}C NMR (100**

MHz, Chloroform-*d*) δ 174.9, 157.6, 144.1, 141.0, 139.4, 139.1, 135.5, 129.0, 128.5, 127.1, 119.9, 111.6, 110.4, 64.0, 55.7, 40.3, 28.9, 25.3, 12.9, 8.4. **HRMS m/z (ESI)** calcd. for $C_{23}H_{25}O_3{}^+$ $(M + H)^+$ 349.1804, found 349.1809.

3-(6-methoxy-3-phenyl-1*H*-inden-2-yl)propyl cyclobutanecarboxylate (1ac)

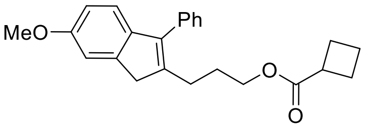

Following the general **procedure G** on 0.1 mmol scale the substrate **1ac** was obtained as a colorless liquid in 35% yield (0.126 g). **^{1}H NMR (400 MHz, Chloroform-*d*)** δ 7.48 (t, *J* = 7.5 Hz, 2H), 7.41 – 7.35 (m, 3H), 7.11 (d, *J* = 8.3 Hz, 2H), 6.82 (dd, *J* = 8.3, 2.4 Hz, 1H), 4.08 (t, *J* = 6.5 Hz, 2H), 3.86 (s, 3H), 3.48 (s, 2H), 3.06 (p, *J* = 8.5 Hz, 1H), 2.69 – 2.54 (m, 2H), 2.33 – 2.11 (m, 4H), 1.92 (dddt, *J* = 22.6, 15.7, 10.8, 5.7 Hz, 4H). **^{13}C NMR (100 MHz, Chloroform-*d*)** δ 175.5, 157.6, 144.1, 141.0, 139.4, 139.1, 135.5, 129.0, 128.5, 127.1, 119.9, 111.6, 110.4, 63.8, 55.7, 40.3, 38.1, 28.9, 25.3, 25.2, 18.4. **HRMS m/z (ESI)** calcd. for $C_{24}H_{27}O_3{}^+$ $(M + H)^+$ 363.1960, found 363.1953.

3-(6-methoxy-3-phenyl-1*H*-inden-2-yl)propyl cyclohexanecarboxylate (1ad)

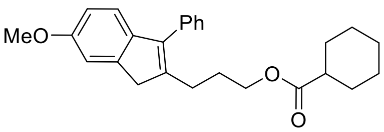

Following the general **procedure G** on 0.1 mmol scale the substrate **1ad** was obtained as a colorless liquid in 73% yield (0.284 g). **^{1}H NMR (400 MHz, Chloroform-*d*)** δ 7.52 – 7.44 (m, 2H), 7.41 – 7.35 (m, 3H), 7.11 (d, *J* = 8.4 Hz, 2H), 6.82 (dd, *J* = 8.3, 2.4 Hz, 1H), 4.06 (t, *J* = 6.4 Hz, 2H), 3.86 (s, 3H), 3.48 (s, 2H), 2.80 – 2.47 (m, 2H), 2.20 (tt, *J* = 11.3, 3.6 Hz, 1H), 1.99 – 1.86 (m, 2H), 1.86 – 1.67 (m, 4H), 1.43 – 1.09 (m, 6H). **^{13}C NMR (100 MHz, Chloroform-*d*)** δ 176.1, 157.6, 144.1, 141.0, 139.5, 139.1, 135.5, 129.0, 128.5, 127.1, 119.9, 111.6, 110.4, 63.6, 55.7, 43.2, 40.3, 29.0, 28.9, 25.7, 25.5, 25.4. **HRMS m/z (ESI)** calcd. for $C_{26}H_{31}O_3{}^+$ $(M + H)^+$ 391.2273, found 391.2265.

3-(6-methoxy-3-phenyl-1*H*-inden-2-yl)propyl 3-bromobenzoate (1ag)

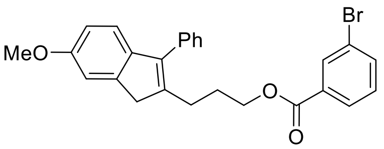

Following the general **procedure G** on 1.0 mmol scale the substrate **1ag** was obtained as a white solid in 87% yield (0.400 g). mp: 102 – 103 ºC. **^{1}H NMR (400 MHz, Chloroform-*d*)** δ 8.06 (s, 1H), 7.82 (d, *J* = 7.8 Hz, 1H), 7.69 (d, *J* = 8.5 Hz, 1H), 7.44 – 7.34 (m, 4H), 7.33 – 7.24 (m, 2H), 7.10 (d, *J* = 7.8 Hz, 2H), 6.82 (dd, *J* = 8.4, 2.3 Hz, 1H), 4.30 (t, *J* = 6.3 Hz, 2H), 3.86 (s, 3H), 3.50 (s, 2H), 2.70 (t, *J* = 7.6 Hz, 2H), 2.05 (p, *J* = 6.7 Hz, 2H). **^{13}C NMR (100 MHz, Chloroform-*d*)** δ 165.2, 157.7, 144.0, 140.6, 139.5, 139.4, 135.8, 135.4, 132.5, 132.2, 129.9, 128.9, 128.5, 128.1, 127.1, 122.4, 119.9, 111.7, 110.4, 64.8, 55.7, 40.3, 28.7, 25.3. **HRMS m/z (ESI)** calcd. for $C_{26}H_{24}BrO_3{}^+$ $(M + H)^+$ 463.0909, found 463.0892.

3-(6-methoxy-3-phenyl-1*H*-inden-2-yl)propyl furan-2-carboxylate (1ah)

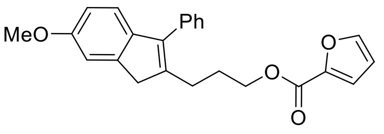

Following the general **procedure G** on 1.0 mmol scale the substrate **1ah** was obtained as a white solid in 76% yield (0.286 g). mp: 118 – 119 ºC. **^{1}H NMR (400 MHz, Chloroform-*d*)** δ 7.57 (d, *J* = 0.9 Hz, 1H), 7.47 – 7.29 (m, 5H), 7.13 – 7.06 (m, 2H), 7.07 –

7.01 (m, 1H), 6.81 (dd, *J* = 8.4, 2.2 Hz, 1H), 6.49 (dd, *J* = 3.5, 1.7 Hz, 1H), 4.29 (t, *J* = 6.5 Hz, 2H), 3.86 (s, 3H), 3.49 (s, 2H), 2.66 (t, *J* = 7.6 Hz, 2H), 2.03 (dt, *J* = 14.0, 6.6 Hz, 2H). **^{13}C NMR (100 MHz, Chloroform-*d*)** δ 158.7, 157.6, 146.2, 144.6, 144.1, 140.7, 139.4, 139.3, 135.4, 129.0, 128.5, 127.1, 119.9, 117.8, 111.8, 111.6, 110.4, 64.4, 55.7, 40.3, 28.8, 25.2. **HRMS m/z (ESI)** calcd. for $C_{24}H_{23}O_4{}^+$ (M + H) $^+$ 375.1596, found 375.1598.

3-(6-methoxy-3-phenyl-1*H*-inden-2-yl)propyl 3-chloro-2,2-dimethylpropanoate (1ai)

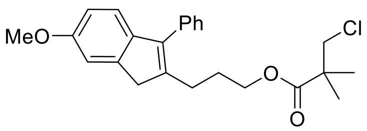

Following the general **procedure G** on 1.0 mmol scale the substrate **1ai** was obtained as a colorless liquid in 78% yield (0.310 g). **^{1}H NMR (400 MHz, Chloroform-*d*)** δ 7.55 – 7.45 (m, 2H), 7.43 – 7.34 (m, 3H), 7.11 (d, *J* = 8.4 Hz, 2H), 6.82 (dd, *J* = 8.3, 2.5 Hz, 1H), 4.13 (t, *J* = 6.3 Hz, 2H), 3.86 (s, 3H), 3.53 (s, 2H), 3.48 (s, 2H), 2.70 – 2.51 (m, 2H), 2.05 – 1.84 (m, 2H), 1.23 (s, 6H). **^{13}C NMR (100 MHz, Chloroform-*d*)** δ 1175.0, 144.0, 140.9, 139.4, 139.2, 135.5, 129.0, 128.5, 127.2, 119.9, 111.6, 110.4, 64.5, 55.7, 52.0, 44.6, 40.3, 28.9, 25.3, 23.2. **HRMS m/z (ESI)** calcd. for $C_{24}H_{28}ClO_3{}^+$ (M + H) $^+$ 399.1727, found 399.1720.

General Procedure H

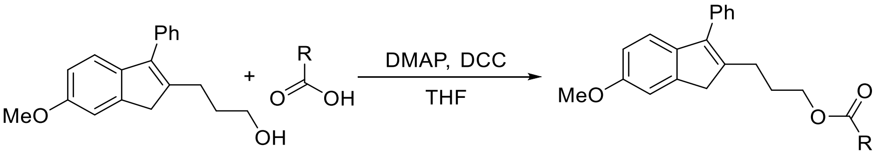

Supplementary Figure 34. General Procedure H to prepare substrate

As that shown in Supplementary Figure 34, 3-(6-methoxy-3-phenyl-1*H*-inden-2-yl) propan-1-ol (0.280 g, 1 mmol), RCOOH (1 mmol, 1.0 eq), DMAP (0.024 g, 0.2 mmol, 0.02 eq) and DCC (0.309 g, 1.5 mmol, 1.5 eq) were dissolved in THF (10 ml) under argon. The solution was stirred overnight at room temperature. The mixture was concentrated, and purified with flash chromatography to afford the desired product.

1-benzyl 2-(3-(6-methoxy-3-phenyl-1*H*-inden-2-yl)propyl) *(S)* pyrrolidine-1,2-dicarboxylate (1ae)

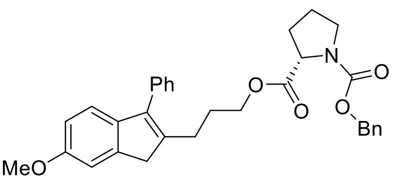

Following the general **procedure H** on 1.0 mmol scale the substrate **1ae** was obtained as a red a liquid in 70% yield (0.356 g). dr = 1.1:1. **^{1}H NMR (400 MHz, Chloroform-*d*)** 7.47 (t, *J* = 7.5 Hz, 2H), 7.38 – 7.26 (m, 8H), 7.09 (d, *J* = 7.9 Hz, 2H), 6.81 (d, *J* = 8.4 Hz, 1H), 5.29 – 4.85 (m, 2H), 4.31 (ddd, *J* = 27.0, 9.0, 3.3 Hz, 1H), 4.14 (t, *J* = 6.3 Hz, 1H), 3.98 (ddt, *J* = 16.0, 10.7, 5.4 Hz, 1H), 3.86 (s, 3H), 3.56 (ddq, *J* = 16.0, 9.5, 5.7, 5.1 Hz, 2H), 3.43 (d, *J* = 27.0 Hz, 2H), 2.52 (dt, *J* = 39.0, 7.7 Hz, 2H), 2.14 (ddt, *J* = 14.7, 10.4, 5.5 Hz, 1H), 1.89 (h, *J* = 9.2, 8.5 Hz, 4H), 1.77 (dt, *J* = 14.1, 7.0 Hz, 1H). **^{13}C NMR (100 MHz, Chloroform-*d*)** δ 172.7, 157.7, 154.3, 144.0, 140.6, 139.4, 139.2, 136.6, 135.5, 129.0, 128.5, 128.3, 127.9, 127.8, 127.1, 119.9, 111.7, 66.9, 64.6, 58.9, 55.7, 46.9, 40.3, 30.9, 28.7, 25.2, 23.5. **HRMS m/z (ESI)** calcd. for $C_{32}H_{34}NO_5{}^+$ (M + H) $^+$ 512.2437, found 512.2429.

3-(6-methoxy-3-phenyl-1*H*-inden-2-yl)propyl acetylphenylalaninate (1af)

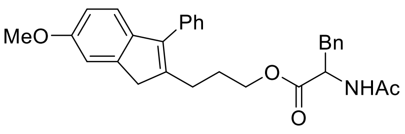

Following the general **procedure H** on 0.1 mmol scale the substrate **1af** was obtained as a red a liquid in 75% yield (0.351 g). **^{1}H NMR (400 MHz, Chloroform-*d*)** 7.47 (t, *J* = 7.5 Hz, 2H), 7.38 (t, *J* = 7.1 Hz, 3H), 7.33 – 7.15 (m, 3H), 7.14 – 7.02 (m, 4H), 6.82 (dd, *J* = 8.4, 2.1 Hz, 1H), 5.95 (d, *J* = 7.5 Hz, 1H), 4.80 (q, *J* = 6.8, 6.1 Hz, 1H), 4.17 – 4.02 (m, 2H), 3.86 (s, 3H), 3.46 (s, 2H), 3.04 (ddd, *J* = 32.7, 13.9, 6.0 Hz, 2H), 2.54 (t, *J* = 7.4 Hz, 2H), 1.98 (s, 3H), 1.88 (t, *J* = 7.0 Hz, 2H). **^{13}C NMR (100 MHz, Chloroform-*d*)** δ 171.7, 169.6, 157.7, 144.0, 140.5, 139.4, 135.9, 135.4, 129.2, 129.0, 128.5, 127.2, 127.1, 119.9, 111.7, 110.4, 65.0, 55.7, 53.2, 40.3, 37.9, 28.6, 25.2, 23.1. **HRMS m/z (ESI)** calcd. for $C_{30}H_{32}NO_4{}^+$ (M + H) $^+$ 470.2331, found 470.2321.

General Procedure I

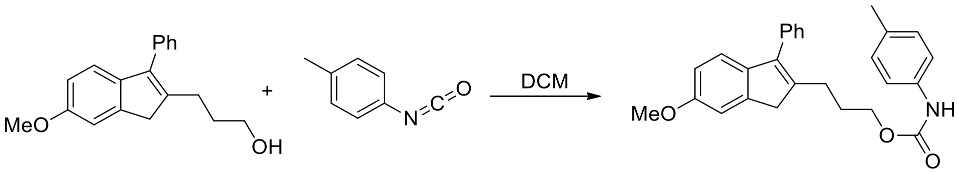

Supplementary Figure 35. General Procedure I to prepare substrate

As that shown in Supplementary Figure 35, to a flask containing 3-(6-methoxy-3-phenyl-1*H*-inden-2-yl)propan-1-ol (0.280 g, 1 mmol) in 5 mL of anhydrous DCM was added a solution of 1-isocyanato-4-methylbenzene (0.200 g, 1.5 mmol, 1.5 equiv) in 5 mL anhydrous DCM at 0 ℃ under argon. The reaction was warmed slowly to room temperature overnight. The reaction mixture was then quenched with water and extracted with DCM for three times. The organic layer was dried ($MgSO_4$), filtered, and concentrated. The residue was purified by flash chromatography on silica gel to afford the desired product (0.211 g, 51%).

3-(6-methoxy-3-phenyl-1*H*-inden-2-yl)propyl *p*-tolylcarbamate (1aj)

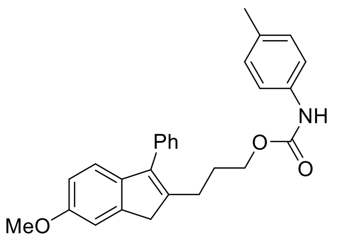

The substrate **1aj** was obtained as a white solid. mp: 86 – 87 °C. **^{1}H NMR (400 MHz, Chloroform-*d*)** 7.52 – 7.42 (m, 2H), 7.38 (dd, *J* = 7.6, 3.8 Hz, 3H), 7.22 (d, *J* = 7.3 Hz, 2H), 7.16 – 7.03 (m, 4H), 6.82 (dd, *J* = 8.4, 2.2 Hz, 1H), 6.30 (s, 1H), 4.13 (t, *J* = 6.4 Hz, 2H), 3.86 (s, 3H), 3.48 (s, 2H), 2.62 (t, *J* = 7.6 Hz, 2H), 2.33 (s, 3H), 1.94 (p, *J* = 6.6 Hz, 2H). **^{13}C NMR (100 MHz, Chloroform-*d*)** δ 157.6, 144.0, 140.9, 139.4, 139.2, 135.6, 129.5, 129.1, 128.5, 127.1, 119.9, 111.6, 110.4, 55.7, 40.3, 29.0, 25.2, 20.8. **HRMS m/z (ESI)** calcd. for $C_{27}H_{28}NO_3{}^+$ (M + H) $^+$ 414.2069, found 414.2060.

9. Characterization of products

6-methoxy-3-methyl-1-phenylisoquinoline (2a)

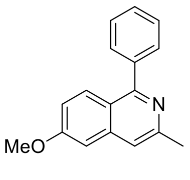

Following the general **procedure A** on 0.1 mmol scale with 4.0 V cell potential, and MeOH/DCM (4 ml/1 ml) as solvent, the substrate **2a** was obtained as a white solid in 65% yield (16.2 mg). mp: 77 – 78 °C. **^{1}H NMR (400 MHz, Chloroform-*d*)** δ 7.91 (d, *J* = 9.1 Hz, 1H), 7.67 (dd, *J* = 8.0, 1.4 Hz, 2H), 7.57 – 7.45 (m, 3H),

7.40 (s, 1H), 7.13 – 7.02 (m, 2H), 3.96 (s, 3H), 2.74 (s, 3H). **^{13}C NMR (100 MHz, Chloroform-*d*)** δ 160.6, 159.8, 151.5, 139.8, 139.7, 129.8, 129.4, 128.4, 128.3, 120.7, 119.0, 117.4, 103. 9, 55.4, 24.4. **HRMS m/z (ESI)** calcd. for $C_{17}H_{16}NO^+$ (M + H)$^+$ 250.1232, found 250.1229.

6-methoxy-3-methyl-1-(p-tolyl)isoquinoline (2b)

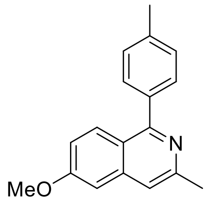

Following the general **procedure A** on 0.2 mmol scale with 4.0 V cell potential, and MeOH/DCM (3 ml/2 ml) as solvent, the substrate **2b** was obtained as a white solid in 64% yield (33.5 mg). mp 80 – 81 °C. **^{1}H NMR (400 MHz, Chloroform-*d*)** δ 7.94 (d, *J* = 9.2 Hz, 1H), 7.58 (d, *J* = 8.0 Hz, 2H), 7.41 – 7.30 (m, 3H), 7.12 – 6.99 (m, 2H), 3.96 (s, 3H), 2.73 (s, 3H), 2.47 (s, 3H). **^{13}C NMR (100 MHz, Chloroform-*d*)** δ 160.5, 159.8, 151.5, 139.6, 138.2, 137.0, 129.8, 129.5, 129.0, 120.7, 118.8, 117.2, 103.8, 55.4, 24.5, 21.4. **HRMS m/z (ESI)** calcd. for $C_{18}H_{18}NO^+$ (M + H)$^+$ 264.1388, found 264.1387.

1-(4-(tert-butyl)phenyl)-6-methoxy-3-methylisoquinoline (2c)

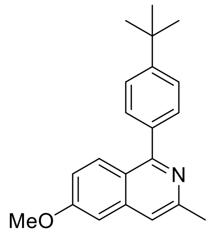

Following the general **procedure A** on 0.1 mmol scale with 4.0 V cell potential, and MeOH/DCM (4 ml/1 ml) as solvent, the substrate **2c** was obtained as a white solid in 61% yield (18.5 mg). mp: 97 – 98 °C. **^{1}H NMR (400 MHz, Chloroform-*d*)** δ 7.98 (d, *J* = 9.2 Hz, 1H), 7.66 – 7.51 (m, 4H), 7.39 (s, 1H), 7.15 – 6.97 (m, 2H), 3.97 (s, 3H), 2.72 (s, 3H), 1.41 (s, 9H). **^{13}C NMR (100 MHz, Chloroform-*d*)** δ 160.5, 159.9, 151.5, 151.3, 139.6, 137.0, 129.6, 129.5, 125.3, 120.7, 118.8, 117.1, 103.8, 55.4, 34.7, 31.4, 24.5. **HRMS m/z (ESI)** calcd. for $C_{21}H_{24}NO^+$ (M + H)$^+$ 306.1858, found 306.1860.

6-methoxy-1-(2-methoxyphenyl)-3-methylisoquinoline (2d)

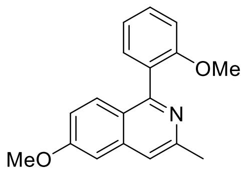

Following the general **procedure A** on 0.1 mmol scale with 4.0 V cell potential, and MeOH/DCM (3 ml/2 ml) as solvent, the substrate **2d** was obtained as a white solid in 73% yield (20.3 mg). mp: 140 – 141 °C. **^{1}H NMR (400 MHz, Chloroform-*d*)** δ 7.56 – 7.50 (m, 1H), 7.43 (td, *J* = 15.8, 7.6 Hz, 3H), 7.12 (t, *J* = 7.4 Hz, 1H), 7.08 – 6.99 (m, 3H), 3.95 (s, 3H), 3.71 (s, 3H), 2.73 (s, 3H). **^{13}C NMR (100 MHz, Chloroform-*d*)** δ 160.5, 157.9, 157.1, 151.5, 138.9, 131.2, 129.8, 129.7, 129.0, 121.7, 120.9, 118.7, 117.6, 111.1, 103.6, 55.5, 55.4, 24.5. **HRMS m/z (ESI)** calcd. for $C_{18}H_{18}NO_2^+$ (M + H)$^+$ 280.1338, found 280.1340.

6-methoxy-3-methyl-1-(4-(trifluoromethoxy)phenyl)isoquinoline (2e)

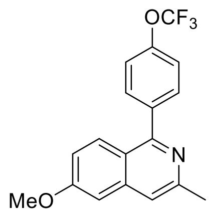

Following the general **procedure A** on 0.1 mmol scale with 4.0 V cell potential, and MeOH/DCM (4 ml/1 ml) as solvent, the substrate **2e** was obtained as a white solid in 69% yield (23.0 mg). mp: 68 – 69 °C. **^{1}H NMR (400 MHz, Chloroform-*d*)** δ 7.87 (d, *J* = 9.2 Hz, 1H), 7.71 (d, *J* = 8.6 Hz, 2H), 7.47 – 7.33 (m, 3H), 7.17 – 6.93 (m, 2H), 3.97 (s, 3H), 2.73 (s, 3H). **^{13}C NMR (100 MHz, Chloroform-*d*)** δ 160.7, 158.2, 151.6, 149.4, 139.7, 138.6, 131.3, 128.9, 124.37, 121.81, 120.9,

120.5(3)(q, *J* = 255.8Hz), 120.5(2), 119.3, 117.8, 104.0, 55.4, 24.4. **HRMS m/z (ESI)** calcd. for $C_{18}H_{15}F_3NO_2{}^+$ $(M + H)^+$ 334.1055, found 334.1052.

1-(3-chloro-4-fluorophenyl)-6-methoxy-3-methylisoquinoline (2f)

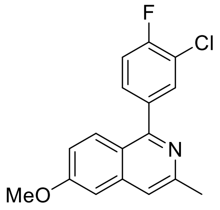

Following the general **procedure A** on 0.11 mmol scale with 4.0 V cell potential, and MeOH/DCM (4 ml/1 ml) as solvent, the substrate **2f** was obtained as a white solid in 61% yield (20.0 mg). mp: 135 – 136 °C. **^{1}H NMR (400 MHz, Chloroform-*d*)** δ 7.84 (d, *J* = 9.2 Hz, 1H), 7.75 (d, *J* = 7.0 Hz, 1H), 7.60 – 7.51 (m, 1H), 7.43 (s, 1H), 7.33 – 7.27 (m, 1H), 7.15 – 7.00 (m, 2H), 3.98 (s, 3H), 2.72 (s, 3H). **^{13}C NMR (100 MHz, Chloroform-*d*)** δ 160.7, 158.3(d, *J* = 248.8 Hz), 157.2, 151.5, 139.7, 137.0(d, *J* = 4.0 Hz), 132.1, 129.7(d, *J* = 7.3 Hz), 128.6, 121.2, 121.1, 120.4, 119.5, 118.0, 116.4(d, *J* = 21.3 Hz), 104.0, 55.5, 24.3. **^{19}F NMR (376 MHz, Chloroform-*d*)** δ -113.64. **HRMS m/z (ESI)** calcd. for $C_{17}H_{14}FClNO^+$ $(M + H)^+$ 302.0748, found 302.0749.

1-(4-chlorophenyl)-6-methoxy-3-methylisoquinoline (2g)

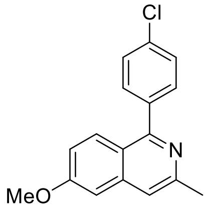

Following the general **procedure A** on 0.1 mmol scale with 4.5 V cell potential, and MeOH/DCM (3 ml/2 ml) as solvent, the substrate **2g** was obtained as a white solid in 66% yield (18.6 mg). mp: 139 – 140 °C. **^{1}H NMR (400 MHz, Chloroform-*d*)** δ 7.87 (d, *J* = 9.2 Hz, 1H), 7.71 – 7.59 (m, 2H), 7.54 – 7.49 (m, 2H), 7.42 (s, 1H), 7.17 – 6.99 (m, 2H), 3.97 (s, 3H), 2.73 (s, 3H). **^{13}C NMR (100 MHz, Chloroform-*d*)** δ 160.8, 158.3, 151.4, 139.8, 134.6, 131.3, 129.0, 128.6, 120.5, 119.4, 117.8, 103.9, 55.5, 24.3. **HRMS m/z (ESI)** calcd. for $C_{17}H_{15}ClNO^+$ $(M + H)^+$ 284.0842, found 284.0843.

1-(4-fluorophenyl)-6-methoxy-3-methylisoquinoline (2h)

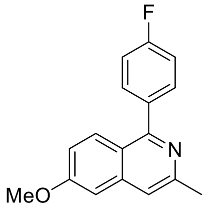

Following the general **procedure A** on 0.1 mmol scale with 4.5 V cell potential, and MeOH/DCM (3 ml/2 ml) as solvent, the substrate **2h** was obtained as a white solid in 60% yield (16.0 mg). mp: 117 – 118 °C. **^{1}H NMR (400 MHz, Chloroform-*d*)** δ 7.87 (d, *J* = 9.2 Hz, 1H), 7.66 (dd, *J* = 8.4, 5.6 Hz, 2H), 7.41 (s, 1H), 7.22 (t, *J* = 8.6 Hz, 2H), 7.15 – 6.96 (m, 2H), 3.97 (s, 3H), 2.72 (s, 3H). **^{13}C NMR (100 MHz, Chloroform-*d*)** δ 163.0 (d, *J* = 247.5 Hz), 160.6, 158.6, 151.5, 139.7, 135.9 (d, *J* = 3.3 Hz), 131.6 (d, *J* = 8.3 Hz), 129.1, 120.6, 119.2, 117.6, 115.3 (d, *J* = 21.4 Hz), 103.9, 55.5, 24.5. **HRMS m/z (ESI)** calcd. for $C_{17}H_{15}FNO^+$ $(M + H)^+$ 268.1138, found 268.1135.

1-(2-chlorophenyl)-6-methoxy-3-methylisoquinoline (2i)

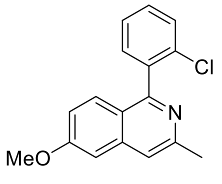

Following the general **procedure A** on 0.1 mmol scale with 4.5 V cell potential, and MeOH/DCM (3 ml/2 ml) as solvent, the substrate **2i** was obtained as a white solid in 51% yield (14.4 mg). mp: 156 – 157 °C. **^{1}H NMR (400 MHz, Chloroform-*d*)** δ 7.56 – 7.52 (m, 1H), 7.50 – 7.39 (m, 5H), 7.07 (d, *J* = 8.6 Hz, 2H), 3.96 (s, 3H), 2.74 (s, 3H). **^{13}C NMR (100 M Hz, Chloroform-*d*)** δ 160.8, 157.5, 151.4, 139.1, 133.3, 131.3, 129.7(3), 129.7(6), 129.0, 126.9, 121.1, 119.5, 118.2, 103.8, 55.5, 24.4. **HRMS m/z (ESI)** calcd. for $C_{17}H_{15}ClNO^+$ $(M + H)^+$ 284.0842, found 284.0843.

1-(3,5-bis(trifluoromethyl)phenyl)-6-methoxy-3-methylisoquinoline (2j)

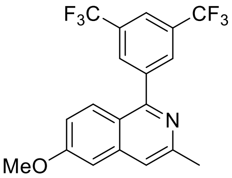

Following the general **procedure A** on 0.1 mmol scale with 4.0 V cell potential, and MeOH/DCM (4 ml/1 ml) as solvent, the substrate **2j** was obtained as a white solid in 50% yield (19.1 mg). mp: 96 – 97 °C. **^{1}H NMR (400 MHz, Chloroform-*d*)** δ 8.16 (s, 2H), 8.02 (s, 1H), 7.74 (d, *J* = 9.2 Hz, 1H), 7.49 (s, 1H), 7.16 (dd, *J* = 9.2, 2.5 Hz, 1H), 7.10 (d, *J* = 2.5 Hz, 1H), 3.99 (s, 3H), 2.74 (s, 3H). **^{13}C NMR (100 MHz, Chloroform-*d*)** δ 160.9, 156.2, 151.8, 141.9, 139.8, 131.8 (q, *J* = 33.5 Hz), 130.1 (q, *J* = 2.6 Hz), 127.8, 123.3 (q, *J* = 271.2 Hz), 122.2 (dt, *J* = 7.4, 3.7 Hz), 120.3, 120.1, 118.6, 104.2, 55.5, 24.2. **HRMS m/z (ESI)** calcd. for $C_{19}H_{14}F_6NO^+$ $(M + H)^+$ 386.0980, found 386.0978.

4-(6-methoxy-3-methylisoquinolin-1-yl)benzonitrile (2k)

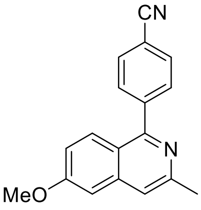

Following the general **procedure A** on 0.1 mmol scale with 4.0 V cell potential, and MeOH/DCM (4 ml/1 ml) as solvent, the substrate **2k** was obtained as a white solid in 65% yield (17.8 mg). mp: 199 – 200 °C. **^{1}H NMR (400 MHz, Chloroform-*d*)** δ 7.96 – 7.70 (m, 5H), 7.46 (s, 1H), 7.19 – 6.82 (m, 2H), 3.98 (s, 3H), 2.73 (s, 3H). **^{13}C NMR (100 MHz, Chloroform-*d*)** δ 160.8, 157.4, 151.7, 144.4, 139.8, 132.2, 130.7, 128.3, 120.3, 119.7, 118.7, 118.3, 112.2, 104.1, 55.5, 24.3. **HRMS m/z (ESI)** calcd. for $C_{18}H_{15}N_2O^+$ $(M + H)^+$ 275.1184, found 275.1182.

1-([1,1'-biphenyl]-4-yl)-6-methoxy-3-methylisoquinoline (2l)

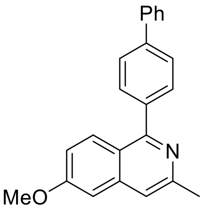

Following the general **procedure A** on 0.1 mmol scale with 4.0 V cell potential, and MeOH/DCM (3 ml/2 ml) as solvent, the substrate **2l** was obtained as a colorless liquid in 51% yield (16.4 mg). **^{1}H NMR (400 MHz, Chloroform-*d*)** δ 8.01 (d, *J* = 9.2 Hz, 1H), 7.77 (s, 4H), 7.70 (d, *J* = 7.0 Hz, 2H), 7.51 (t, *J* = 7.6 Hz, 2H), 7.42 (d, *J* = 5.8 Hz, 2H), 7.14 – 7.01 (m, 2H), 3.98 (s, 3H), 2.75 (s, 3H). **^{13}C NMR (100 MHz, Chloroform-*d*)** δ 160.6, 159.4, 151.6, 141.3, 140.9, 139.7, 138.8, 130.3, 129.3, 128.8, 127.5, 127.2, 127.1, 120.7, 119.0, 117.4, 103.9, 55.4, 24.5. **HRMS m/z (ESI)** calcd. for $C_{23}H_{20}NO^+$ $(M + H)^+$ 326.1545, found 326.1544.

6-methoxy-3-methyl-1-(4-(phenylselanyl)phenyl)isoquinoline (2m)

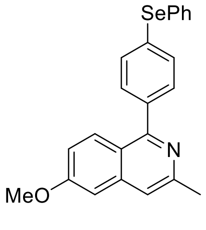

Following the general **procedure A** on 0.1 mmol scale with 3.5 V cell potential, and MeOH/DCM (3 ml/2 ml) as solvent, the substrate **2m** was obtained as a white oil in 60% yield (24.5 mg). **^{1}H NMR (400 MHz, Chloroform-*d*)** δ 7.90 (d, *J* = 9.1 Hz, 1H), 7.66 – 7.52 (m, 5H), 7.40 (s, 1H), 7.35 – 7.30 (m, 4H), 7.15 – 6.87 (m, 2H), 3.96 (s, 3H), 2.72 (s, 3H). **^{13}C NMR (100 MHz, Chloroform-*d*)** δ 160.6, 159.0, 151.5, 139.7, 138.9, 133.3, 132.7, 131.8, 130.8, 129.4, 129.1, 127.5, 120.6, 119.1, 117.6, 103.9, 55.4, 24.4. **HRMS m/z (ESI)** calcd. for $C_{23}H_{20}NOSe^+$ $(M + H)^+$ 406.0710, found 406.0711.

6-methoxy-3-methyl-1-(naphthalen-1-yl)isoquinoline (2n)

Following the general **procedure A** on 0.1 mmol scale with 4.0 V cell potential, and MeOH/DCM (4

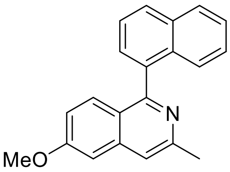

ml/1 ml) as solvent, the substrate **2n** was obtained as a white solid in 48% yield (14.4 mg). mp: 154 – 155 °C. **^{1}H NMR (400 MHz, Chloroform-*d*)** δ 7.97 (dd, *J* = 16.4, 8.0 Hz, 2H), 7.66 – 7.56 (m, 2H), 7.52 – 7.45 (m, 2H), 7.44 – 7.40 (m, 2H), 7.34 (ddd, *J* = 8.2, 6.7, 1.3 Hz, 1H), 7.10 (d, *J* = 2.5 Hz, 1H), 6.95 (dd, *J* = 9.2, 2.5 Hz, 1H), 3.97 (s, 3H), 2.77 (s, 3H). **^{13}C NMR (100 MHz, Chloroform-*d*)** δ 160.8, 159.3, 151.6, 139.2, 137.3, 133.7, 132.4, 129.5, 128.6, 128.2, 127.5, 126.2, 126.1, 125.8, 125.2, 122.3, 119.1, 117.7, 103.7, 55.4, 24.5. **HRMS m/z (ESI)** calcd. for $C_{21}H_{18}NO^+$ (M + H)$^+$ 300.1388, found 300.1387.

6-methoxy-3-methyl-1-(pyridin-2-yl)isoquinoline (2o)

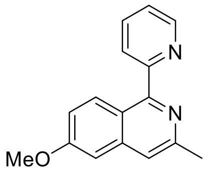

Following the general **procedure A** on 0.1 mmol scale with 4.0 V cell potential, and MeOH/DCM (4 ml/1 ml) as solvent, the substrate **2o** was obtained as a white solid in 60% yield (15.0 mg). mp: 90 – 91 °C. **^{1}H NMR (400 MHz, Chloroform-*d*)** δ 8.79 (d, *J* = 4.0 Hz, 1H), 8.38 (d, *J* = 9.3 Hz, 1H), 8.04 – 7.77 (m, 2H), 7.46 (s, 1H), 7.41 – 7.32 (m, 1H), 7.14 (dd, *J* = 9.3, 2.5 Hz, 1H), 7.04 (d, *J* = 2.4 Hz, 1H), 3.96 (s, 3H), 2.74 (s, 3H). **^{13}C NMR (100 MHz, Chloroform-*d*)** δ 60.5, 158.5, 156.7, 151.3, 148.7, 140.0, 136.8, 129.4, 125.2, 123.0, 120.7, 119.5, 118.6, 103.8, 55.4, 24.3. **HRMS m/z (ESI)** calcd. for $C_{16}H_{15}N_2O^+$ (M + H)$^+$ 251.1184 found 251.1181.

6-methoxy-3-methyl-1-(thiophen-3-yl)isoquinoline (2p)

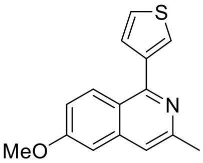

Following the general **procedure A** on 0.1 mmol scale with 4.0 V cell potential, and MeOH/DCM (3 ml/2 ml) as solvent, the substrate **2p** was obtained as a white solid in 60% yield (15.2 mg). mp: 83 – 84 °C. **^{1}H NMR (400 MHz, Chloroform-*d*)** δ 8.08 (d, *J* = 9.2 Hz, 1H), 7.67 (s, 1H), 7.55 – 7.43 (m, 2H), 7.37 (s, 1H), 7.11 (d, *J* = 9.4 Hz, 1H), 7.03 (s, 1H), 3.96 (s, 3H), 2.71 (s, 3H). **^{13}C NMR (100 MHz, Chloroform-*d*)** δ 160.6, 154.9, 151.5, 141.0, 139.6, 129.3, 129.0, 125.8, 125.5, 120.9, 119.1, 117.4, 103.9, 55.4, 24.4. **HRMS m/z (ESI)** calcd. for $C_{15}H_{14}NOS^+$ (M + H)$^+$ 256.0796 found 256.0795.

6-methoxy-3-methyl-1-(1-methyl-1H-indol-5-yl)isoquinoline (2q)

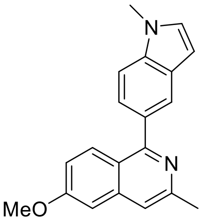

Following the general **procedure A** on 0.1 mmol scale with 4.0 V cell potential, and MeOH/DCM (3 ml/2 ml) as solvent, the substrate **2q** was obtained as a white solid in 41% yield (12.3 mg). mp: 61 – 62 °C. **^{1}H NMR (400 MHz, Chloroform-*d*)** δ 8.02 (d, *J* = 9.9 Hz, 1H), 7.96 – 7.85 (m, 1H), 7.56 (dd, *J* = 8.4, 1.5 Hz, 1H), 7.46 (d, *J* = 8.4 Hz, 1H), 7.38 (s, 1H), 7.14 (d, *J* = 3.1 Hz, 1H), 7.06 (dd, *J* = 6.2, 2.5 Hz, 2H), 6.58 (d, *J* = 3.0 Hz, 1H), 3.97 (s, 3H), 3.89 (s, 3H), 2.75 (s, 3H). **^{13}C NMR (100 MHz, Chloroform-*d*)** δ 161.1, 160.5, 151.3, 139.7, 136.8, 131.0, 130.1, 129.5, 128.4, 123.8, 122.7, 121.1, 118.6, 116.8, 109.0, 103.8, 101.6, 55.4, 33.0, 24.5. **HRMS m/z (ESI)** calcd. for $C_{20}H_{19}N_2O^+$ (M + H)$^+$ 303.1497, found 303.1502.

1-benzyl-6-methoxy-3-methylisoquinoline (2r)

Following the general **procedure A** on 0.1 mmol scale with 4.0 V cell potential, and MeOH/DCM (3 ml/2 ml) as solvent, the substrate **2r** was obtained as a white solid in 62% yield (16.3 mg). mp: 87 – 88

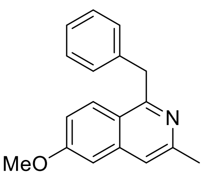

°C. **¹H NMR (400 MHz, Chloroform-*d*)** δ7.97 (d, *J* = 9.2 Hz, 1H), 7.32 (s, 1H), 7.26 (d, *J* = 4.4 Hz, 4H), 7.20 – 7.14 (m, 1H), 7.06 (dd, *J* = 9.2, 2.3 Hz, 1H), 6.98 (d, *J* = 2.1 Hz, 1H), 4.62 (s, 2H), 3.92 (s, 3H), 2.70 (s, 3H). **¹³C NMR (100 MHz, Chloroform-*d*)** δ 160.4, 158.9, 151.2, 139.7, 139.5, 128.4(4), 128.4(2), 127.7, 126.1, 121.0, 119.0, 117.4, 104.3, 55.4, 42.2, 24.4. **HRMS m/z (ESI)** calcd. for $C_{18}H_{18}NO^+$ $(M + H)^+$ 264.1388, found 264.1391.

1-ethyl-6-methoxy-3-methylisoquinoline (2s)

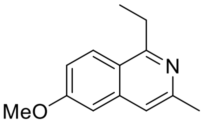

Following the general **procedure A** on 0.2 mmol scale with 4.0 V cell potential, and MeOH/DCM (4 ml/1 ml) as solvent, the substrate **2s** was obtained as a white solid in 51% yield (21.6 mg). mp: 72 – 73 °C. **¹H NMR (400 MHz, Chloroform-*d*)** δ 8.02 (d, *J* = 9.2 Hz, 1H), 7.25 (s, 1H), 7.13 (dd, *J* = 9.2, 2.4 Hz, 1H), 6.98 (d, *J* = 2.3 Hz, 1H), 3.94 (s, 3H), 3.25 (q, *J* = 7.6 Hz, 2H), 2.64 (s, 3H), 1.41 (t, *J* = 7.6 Hz, 3H). **¹³C NMR (100 MHz, Chloroform-*d*)** δ 162.4, 160.4, 151.1, 139.2, 127.1, 120.3, 118.7, 116.7, 104.3, 55.4, 28.9, 24.4, 14.6. **HRMS m/z (ESI)** calcd. for $C_{13}H_{16}NO^+$ $(M + H)^+$ 202.1232, found 202.1230.

1-cyclopropyl-6-methoxy-3-methylisoquinoline (2t)

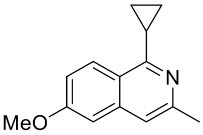

Following the general **procedure A** on 0.2 mmol scale with 4.0 V cell potential, and MeOH/DCM (4 ml/1 ml) as solvent, the substrate **2t** was obtained as a white solid in 54% yield (23.0 mg). mp: 88 – 89 °C. **¹H NMR (400 MHz, Chloroform-*d*)** δ 8.28 (d, *J* = 9.2 Hz, 1H), 7.22 – 7.09 (m, 2H), 6.97 (d, *J* = 2.6 Hz, 1H), 3.95 (s, 3H), 2.65 (tt, *J* = 8.2, 4.9 Hz, 1H), 2.58 (s, 3H), 1.26– 1.19 (m, 2H), 1.12 – 0.97 (m, 2H). **¹³C NMR (100 MHz, Chloroform-*d*)** δ 160.4, 160.3, 151.2, 138.8, 126.9, 121.6, 118.4, 115.9, 104.2, 55.4, 24.5, 13.58, 8.9. **HRMS m/z (ESI)** calcd. for $C_{14}H_{16}NO^+$ $(M + H)^+$ 214.1232, found 214.1234.

3-allyl-6-methoxy-1-phenylisoquinoline (2u)

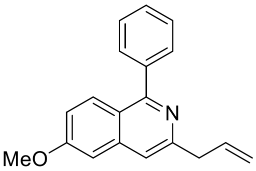

Following the general **procedure A** on 0.1 mmol scale with 4.0 V cell potential, and MeOH/DCM (4 ml/1 ml) as solvent, the substrate **2u** was obtained as a colorless liquid in 54% yield (14.8 mg). **¹H NMR (400 MHz, Chloroform-*d*)** δ 7.93 (d, *J* = 10.3 Hz, 1H), 7.68 (dd, *J* = 8.0, 1.4 Hz, 2H), 7.58 – 7.46 (m, 3H), 7.43 (s, 1H), 7.15 – 7.04 (m, 2H), 6.24 (ddt, *J* = 17.0, 10.1, 6.9 Hz, 1H), 5.45 – 5.10 (m, 2H), 3.97 (s, 3H), 3.79 (d, *J* = 6.8 Hz, 2H). **¹³C NMR (100 MHz, Chloroform-*d*)** δ 160. 6, 159.9, 153.4, 139.8, 139.7, 136.34, 129.9, 129.4, 128.4, 128.3, 121.0, 119.2, 117.0, 116.6, 104.2, 55.4, 42.7. **HRMS m/z (ESI)** calcd. for $C_{19}H_{18}NO^+$ $(M + H)^+$ 276.1388, found 276.1390.

3-((6-methoxy-1-phenylisoquinolin-3-yl)methoxy)-13-methyl-6,7,8,9,11,12,13,14,15,16-decahydro-17H-cyclopenta[a]phenanthren-17-one (2v)

Following the general **procedure A** on 0.1 mmol scale with 4.5 V cell potential, and MeOH/DCM (4

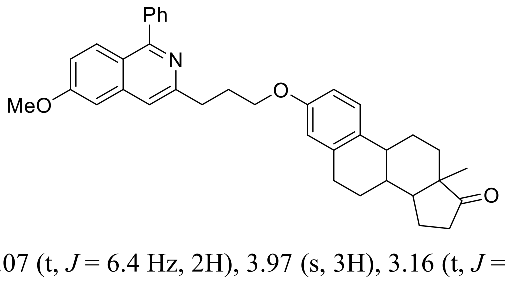

ml/3 ml) as solvent, the substrate **2v** was obtained as a colorless liquid in 60% yield (32.4 mg). **^{1}H NMR (400 MHz, Chloroform-*d*)** δ 7.94 (d, *J* = 9.1 Hz, 1H), 7.72 – 7.64 (m, 2H), 7.58 – 7.47 (m, 3H), 7.44 (s, 1H), 7.21 (d, *J* = 8.6 Hz, 1H), 7.15 – 7.03 (m, 2H), 6.76 (dd, *J* = 8.5, 2.6 Hz, 1H), 6.67 (d, *J* = 2.4 Hz, 1H), 4.07 (t, *J* = 6.4 Hz, 2H), 3.97 (s, 3H), 3.16 (t, *J* = 7.5 Hz, 2H), 2.90 (dt, *J* = 6.6, 3.2 Hz, 2H), 2.53 (dd, *J* = 18.8, 8.6 Hz, 1H), 2.45 – 2.33 (m, 3H), 2.31 – 2.23 (m, 1H), 2.21 – 1.92 (m, 4H), 1.70 – 1.40 (m, 6H), 0.93 (s, 3H). **^{13}C NMR (100 MHz, Chloroform-*d*)** δ 221.0, 160.6, 159.9, 157.1, 154.4, 139.9, 139.6, 137.7, 131.9, 129.9, 129.3, 128.4, 128.3, 126.3, 121.0, 119.2, 117.2, 114.6, 112.3, 104.1, 67.3, 55.5, 50.4, 48.0, 44.0, 38.4, 35.9, 34.6, 31.6, 29.7, 29.4, 26.6, 25.9, 21.6, 13.9. **HRMS m/z (ESI)** calcd. for $C_{37}H_{40}NO_3{}^+$ (M + H) $^+$ 546.3008, found 546.3010.

6-methoxy-1-phenyl-3-(3-((1-phenyl-1H-tetrazol-5-yl)thio)propyl)isoquinoline (2w)

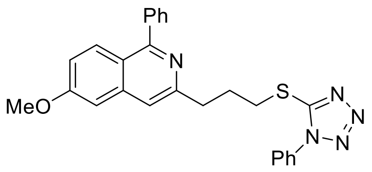

Following the general **procedure A** on 0.1 mmol scale with 4.0 V cell potential, and MeOH/DCM (4 ml/1 ml) as solvent, the substrate **2w** was obtained as a colorless liquid in 50% yield (22.4 mg). **^{1}H NMR (400 MHz, Chloroform-*d*)** δ 7.93 (d, *J* = 8.9 Hz, 1H), 7.70 – 7.62 (m, 2H), 7.62 – 7.46 (m, 8H), 7.43 (s, 1H), 7.15 – 7.05 (m, 2H), 3.97 (s, 3H), 3.59 – 3.49 (m, 2H), 3.17 – 3.10 (m, 2H), 2.44 (p, *J* = 7.2 Hz, 2H). **^{13}C NMR (100 MHz, Chloroform-*d*)** δ 160.7, 160.0, 154.4, 153.4, 139.7, 139.6, 133.7, 130.1, 129.9, 129.8, 129.3, 128.5, 128.3, 123.8, 121.0, 119.4, 117.3, 104.1, 55.5, 36.8, 32.9, 29.0. **HRMS m/z (ESI)** calcd. for $C_{26}H_{24}N_5OS^+$ (M + H) $^+$ 454.1702, found 454.1707.

***tert*-butyl (3-(6-methoxy-1-phenylisoquinolin-3-yl)propyl)(tosyl)carbamate (2x)**

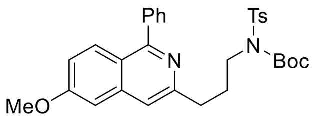

Following the general **procedure A** on 0.1 mmol scale with 4.0 V cell potential, and MeOH/DCM (4 ml/1 ml) as solvent, the substrate **2x** was obtained as a colorless liquid in 56% yield (30.5 mg). **^{1}H NMR (400 MHz, Chloroform-*d*)** δ 7.94 (d, *J* = 9.9 Hz, 1H), 7.83 (d, *J* = 8.3 Hz, 2H), 7.76 – 7.67 (m, 2H), 7.59 – 7.45 (m, 4H), 7.27 (d, *J* = 7.8 Hz, 2H), 7.10 (dq, *J* = 4.6, 2.6 Hz, 2H), 3.98 (s, 5H), 3.05 (t, *J* = 7.6 Hz, 2H), 2.44 (s, 3H), 2.35 (p, *J* = 7.7 Hz, 2H), 1.33 (s, 9H). **^{13}C NMR (100 MHz, Chloroform-*d*)** δ 160.6, 159.8, 154.1, 151.0, 143.9, 139.9, 139.6, 137.5, 130.0, 129.3, 129.2, 128.4, 128.3, 127.9, 121.0, 119.2, 117.0, 104.2, 84.0, 55.5, 46.9, 35.2, 30.1, 27.9, 21.6. **HRMS m/z (ESI)** calcd. for $C_{31}H_{35}N_2O_5S^+$ (M + H) $^+$ 547.2267, found 547.2273.

3-(3-((tert-butyldiphenylsilyl)oxy)propyl)-6-methoxy-1-phenylisoquinoline (2y)

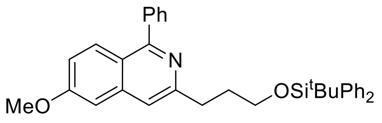

Following the general **procedure A** on 0.1 mmol scale with 4.5 V cell potential, and MeOH/DCM (4 ml/1 ml) as solvent, the substrate **2y** was obtained as a colorless liquid in 53% yield (28.0 mg). **^{1}H NMR (400 MHz, Chloroform-*d*)** δ 7.93 (d, *J* = 9.2 Hz, 1H), 7.75 – 7.62 (m, 6H), 7.57 – 7.46 (m, 3H), 7.47 – 7.32 (m, 7H), 7.10 (dd, *J* = 9.2, 2.5 Hz, 1H), 7.06 (d, *J* = 2.5 Hz, 1H), 3.98 (s, 3H), 3.81 (t, *J* = 6.3 Hz, 2H), 3.17 – 3.02 (m, 2H), 2.22 – 2.11 (m, 2H), 1.10

(s, 9H). **^{13}C NMR (100 MHz, Chloroform-*d*)** δ 160.5, 159.7, 155.1, 139.9, 139.6, 135.6, 134.1, 129.9, 129.5, 129.3, 128.3(3), 128.3(8), 127.6, 120.9, 119.0, 117.0, 104.1, 63.4, 55.4, 34.5, 32.6, 26.9, 19.3. **HRMS m/z (ESI)** calcd. for $C_{35}H_{38}NO_2Si^+$ $(M + H)^+$ 532.2672, found 532.2677.

3-(3-(7*H*-purin-7-yl)propyl)-6-methoxy-1-phenylisoquinoline (2z)

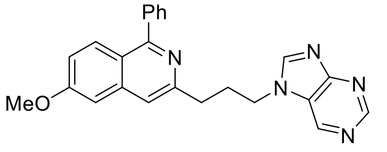

Following the general **procedure A** on 0.1 mmol scale with 4.0 V cell potential, and MeOH/DCM (4 ml/1 ml) as solvent, the substrate **2z** was obtained as a colorless liquid in 57% yield (22.4 mg). **^{1}H NMR (400 MHz, Chloroform-*d*)** δ 9.12 (s, 1H), 9.01 (s, 1H), 8.23 (s, 1H), 7.94 (d, *J* = 9.2 Hz, 1H), 7.72 – 7.62 (m, 2H), 7.60 – 7.47 (m, 3H), 7.35 (s, 1H), 7.12 (dd, *J* = 9.3, 2.5 Hz, 1H), 7.03 (d, *J* = 2.5 Hz, 1H), 4.46 (t, *J* = 7.0 Hz, 2H), 3.97 (s, 3H), 3.02 (t, *J* = 7.2 Hz, 2H), 2.55 (p, *J* = 7.2 Hz, 2H). **^{13}C NMR (100 MHz, Chloroform-*d*)** δ 160.7, 160.0, 152.9, 152.5, 151.5, 148.5, 145.8, 139.6, 139.6, 134.1, 129.8, 129.4, 128.6, 128.4, 121.0, 119.5, 117.4, 104.0, 55.5, 43.3, 34.6, 29.6. **HRMS m/z (ESI)** calcd. for $C_{24}H_{22}N_5O^+$ $(M + H)^+$ 396.1824, found 396.1829.

3-(6-methoxy-1-phenylisoquinolin-3-yl)propyl acetate (2aa)

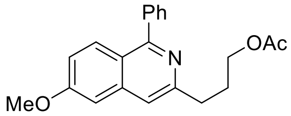

Following the general **procedure A** on 0.1 mmol scale with 4.5 V cell potential, and MeOH/DCM (4 ml/1 ml) as solvent, the substrate **2aa** was obtained as a colorless liquid in 74% yield (24.7 mg). **^{1}H NMR (400 MHz, Chloroform-*d*)** δ 7.93 (d, *J* = 9.0 Hz, 1H), 7.68 (dd, *J* = 7.9, 1.5 Hz, 2H), 7.58 – 7.44 (m, 3H), 7.41 (s, 1H), 7.15 – 7.05 (m, 2H), 4.20 (t, *J* = 6.6 Hz, 2H), 3.97 (s, 3H), 3.08 – 3.02 (m, 2H), 2.23 (dt, *J* = 14.2, 6.7 Hz, 2H), 7.41 (s, 3H). **^{13}C NMR (100 MHz, Chloroform-*d*)** δ 171.2, 160.6, 159.9, 154.1, 139.8, 139.6, 129.9, 129.3, 128.4, 128.4, 128.3, 121.0, 119.2, 117.1, 104.1, 64.2, 55.4, 34.5, 28.7, 21.0. **HRMS m/z (ESI)** calcd. for $C_{21}H_{22}NO_3^+$ $(M + H)^+$ 336.1600, found 336.1601.

3-(6-methoxy-1-phenylisoquinolin-3-yl)propyl cyclopropanecarboxylate (2ab)

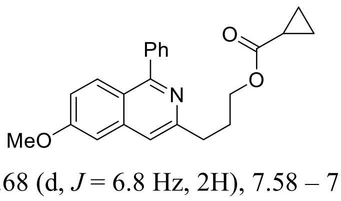

Following the general **procedure A** on 0.1 mmol scale with 4.2 V cell potential, and MeOH/DCM (4 ml/1 ml) as solvent, the substrate **2ab** was obtained as a white solid in 64% yield (23.0 mg). mp: 90.1 – 91.2 °C. **^{1}H NMR (400 MHz, Chloroform-*d*)** δ 7.94 (d, *J* = 9.0 Hz, 1H), 7.68 (d, *J* = 6.8 Hz, 2H), 7.58 – 7.44 (m, 3H), 7.42 (s, 1H), 7.10 (d, *J* = 9.3 Hz, 2H), 4.21 (t, *J* = 6.5 Hz, 2H), 3.97 (s, 3H), 3.22 – 2.91 (m, 2H), 2.23 (p, *J* = 6.7 Hz, 2H), 1.61 (td, *J* = 8.1, 4.2 Hz, 1H), 1.12 – 0.93 (m, 2H), 0.85 (dq, *J* = 7.6, 4.0 Hz, 2H). **^{13}C NMR (100 MHz, Chloroform-*d*)** δ 175.1, 160.6, 159.9, 154.1, 139.8, 139.6, 129.9, 129.4, 128.5, 128.4, 121.0, 119.2, 117.1, 104.1, 64.2, 55.5, 34.6, 28.8, 12.9, 8.4. **HRMS m/z (ESI)** calcd. for $C_{23}H_{24}NO_3^+$ $(M + H)^+$ 362.1756, found 362.1758.

3-(6-methoxy-1-phenylisoquinolin-3-yl)propyl cyclobutanecarboxylate (2ac)

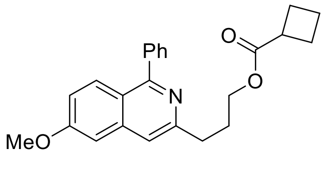

Following the general **procedure A** on 0.1 mmol scale with 4.0 V cell potential, and MeOH/DCM (4 ml/1 ml) as solvent, the substrate **2ac** was obtained as a colorless liquid in 50% yield (18.6 mg). **^{1}H NMR (400 MHz, Chloroform-*d*)** δ (d, *J* = 9.0 Hz, 1H), 7.68 (d, *J* = 7.8 Hz,

2H), 7.59 – 7.43 (m, 3H), 7.41 (s, 1H), 7.10 (d, *J* = 11.0 Hz, 2H), 4.21 (t, *J* = 6.1 Hz, 2H), 3.97 (s, 3H), 3.15 (p, *J* = 8.4 Hz, 1H), 3.05 (t, *J* = 7.5 Hz, 2H), 2.42 – 2.13 (m, 6H), 1.96 (ddd, *J* = 33.9, 16.8, 10.1 Hz, 2H). **^{13}C NMR (100 MHz, Chloroform-*d*)** δ 175.6, 160.6, 159.9, 154.1, 139.8, 139.6, 129.9, 129.4, 128.4, 128.3, 121.0, 119.2, 117.1, 104.1, 64.0, 55.5, 38.2, 34.6, 28.7, 25.3, 18.4. **HRMS m/z (ESI)** calcd. for $C_{24}H_{26}NO_3^+$ (M + H)$^+$ 376.1913, found 376.1917.

3-(6-methoxy-1-phenylisoquinolin-3-yl)propyl cyclohexanecarboxylate (2ad)

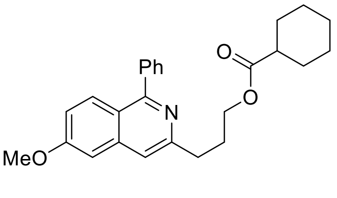

Following the general **procedure A** on 0.1 mmol scale with 4.0 V cell potential, and MeOH/DCM (4 ml/1 ml) as solvent, the substrate **2ad** was obtained as a colorless liquid in 51% yield (20.4 mg). **^{1}H NMR (400 MHz, Chloroform-*d*)** δ 7.93 (d, *J* = 9.0 Hz, 1H), 7.68 (dd, *J* = 8.0, 1.4 Hz, 2H), 7.58 – 7.44 (m, 3H), 7.41 (s, 1H), 7.14 – 7.02 (m, 2H), 4.19 (t, *J* = 6.5 Hz, 2H), 3.97 (s, 3H), 3.17 – 2.96 (m, 2H), 2.34 – 2.16 (m, 3H), 1.91 (d, *J* = 13.2 Hz, 2H), 1.81 – 1.59 (m, 4H), 1.53 – 1.38 (m, 2H), 1.36 – 1.17 (m, 2H). **^{13}C NMR (100 MHz, Chloroform-*d*)** δ 176.3, 160.6, 159.9, 154.2, 139.8, 139.6, 129.9, 129.4, 128.4, 128.3, 121.0, 119.2, 117.1, 104.1, 63.8, 55.5, 43.3, 34.6, 29.1, 28.7, 25.8, 25.5. **HRMS m/z (ESI)** calcd. for $C_{26}H_{30}NO_3^+$ (M + H)$^+$ 404.2226, found 404.2230.

1-benzyl 2-(3-(6-methoxy-1-phenylisoquinolin-3-yl)propyl) *(S)* pyrrolidine-1,2-dicarboxylate (2ae)

Following the general **procedure A** on 0.1 mmol scale with 4.0 V cell potential, and MeOH/DCM (4 ml/1 ml) as solvent, the substrate **2ae** was obtained as a colorless liquid in 62% yield (32.5 mg). dr = 1.1:1. **^{1}H NMR (400 MHz, Chloroform-*d*)** δ 7.93 (dd, *J* = 9.2, 3.0 Hz, 1H), 7.67 (dd, *J* = 6.3, 1.7 Hz, 2H), 7.58 – 7.45 (m, 3H), 7.45 – 7.28 (m, 5H), 7.29 – 7.18 (m, 1H), 7.15 – 7.02 (m, 2H), 5.26 – 5.07 (m, 2H), 4.42 (ddd, *J* = 25.3, 8.6, 3.5 Hz, 1H), 4.27 (td, *J* = 6.4, 1.8 Hz, 1H), 4.23 – 4.06 (m, 1H), 3.97 (d, *J* = 4.3 Hz, 3H), 3.75 – 3.46 (m, 2H), 3.00 (dt, *J* = 37.9, 7.6 Hz, 2H), 2.24 (dq, *J* = 13.4, 6.8 Hz, 2H), 2.12 (p, *J* = 6.6 Hz, 1H), 2.08 – 1.86 (m, 3H). **^{13}C NMR (100 MHz, Chloroform-*d*)** δ 172.7, 160.6, 159.9, 154.4, 153.8, 139.8, 139.6, 136.6, 129.9, 129.3, 128.5, 128.3, 127.8, 127.7, 121.0, 119.3, 117.1, 104.1, 67.0, 64.7, 59.0, 55.5, 47.0, 34.3, 31.0, 28.6, 23.5. **HRMS m/z (ESI)** calcd. for $C_{32}H_{33}N_2O_5^+$ (M + H)$^+$ 525.2389, found 525.2394.

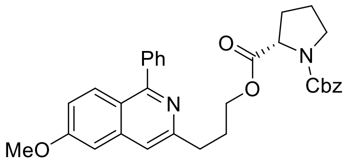

3-(6-methoxy-1-phenylisoquinolin-3-yl)propyl acetylphenylalaninate (2af)

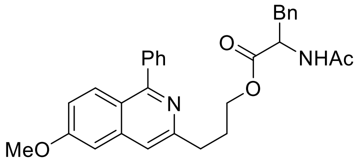

Following the general **procedure A** on 0.1 mmol scale with 4.5 V cell potential, and MeOH/DCM (3 ml/2 ml) as solvent, the substrate **2af** was obtained as a colorless liquid in 56% yield (26.8 mg). **^{1}H NMR (400 MHz, Chloroform-*d*)** δ 7.93 (d, *J* = 9.0 Hz, 1H), 7.67 (dd, *J* = 7.9, 1.4 Hz, 2H), 7.57 – 7.49 (m, 3H), 7.40 (s, 1H), 7.32 – 7.20 (m, 3H), 7.11 (ddd, *J* = 13.4, 4.9, 1.9 Hz, 4H), 6.01 (d, *J* = 7.7 Hz, 1H), 4.95 – 4.87 (m, 1H), 4.35 – 4.17 (m, 2H), 3.97 (s, 3H), 3.14 (t, *J* = 5.4 Hz, 2H), 3.04 – 2.96 (m, 2H), 2.22 (p, *J* = 6.6 Hz, 2H), 2.00 (s, 3H). **^{13}C NMR (100 MHz, Chloroform-*d*)** δ 171.8, 169.7, 160.7, 160.0, 153.7, 139.7, 139.6, 135.9, 129.9, 129.4, 129.3, 128.6, 128.5, 128.4, 127.1, 121.0, 119.4, 117.3, 104.1, 65.2, 55.5, 53.2, 38.0, 34.3, 28.5, 23.2. **HRMS m/z (ESI)** calcd. for $C_{30}H_{31}N_2O_4^+$ (M + H)$^+$ 483.2284,found 483.2283.

3-(6-methoxy-1-phenylisoquinolin-3-yl)propyl 3-bromobenzoate (2ag)

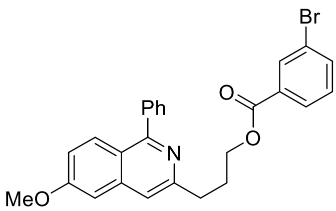

Following the general **procedure A** on 0.1 mmol scale with 4.0 V cell potential, and MeOH/DCM (4 ml/2 ml) as solvent, the substrate **2ag** was obtained as a colorless liquid in 50% yield (23.5 mg). **^{1}H NMR (400 MHz, Chloroform-*d*)** δ 8.15 (s, 1H), 7.92 (d, *J* = 8.8 Hz, 2H), 7.68 (d, *J* = 6.7 Hz, 3H), 7.59 – 7.46 (m, 3H), 7.43 (s, 1H), 7.33 – 7.23 (m, 1H), 7.14 – 6.98 (m, 2H), 4.48 (t, *J* = 6.4 Hz, 2H), 3.97 (s, 3H), 3.14 (t, *J* = 7.3 Hz, 2H), 2.39 (p, *J* = 6.7 Hz, 2H). **^{13}C NMR (100 MHz, Chloroform-*d*)** δ 165.3, 160.6, 160.0, 153.9, 139.8, 139.6, 135.8, 132.5, 132.3, 129.9, 129.8, 129.4, 128.5, 128.3, 128.1, 122.4, 121.0, 119.3, 117.2, 104.1, 65.2, 55.5, 34.8, 28.6. **HRMS m/z (ESI)** calcd. for $C_{26}H_{23}BrNO_3{}^+$ $(M + H)^+$ 476.0861, found 476.0860.

3-(6-methoxy-1-phenylisoquinolin-3-yl)propyl furan-2-carboxylate (2ah)

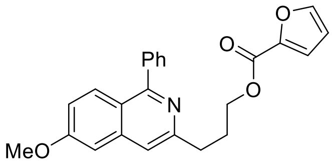

Following the general **procedure A** on 0.1 mmol scale with 4.5 V cell potential, and MeOH/DCM (4 ml/1 ml) as solvent, the substrate **2ah** was obtained as a colorless liquid in 61% yield (23.6 mg). **^{1}H NMR (400 MHz, Chloroform-*d*)** δ 7.93 (d, *J* = 9.0 Hz, 1H), 7.70 – 7.65 (m, 2H), 7.58 – 7.46 (m, 4H), 7.43 (s, 1H), 7.15 (d, *J* = 3.1 Hz, 1H), 7.12 – 7.05 (m, 2H), 6.49 (dd, *J* = 3.5, 1.7 Hz, 1H), 4.45 (t, *J* = 6.5 Hz, 2H), 3.97 (s, 3H), 3.12 (t, *J* = 7.5 Hz, 2H), 2.36 (p, *J* = 6.6 Hz, 2H). **^{13}C NMR (100 MHz, Chloroform-*d*)** δ 160.6, 160.0, 158.9, 153.9, 146.2, 144.8, 139.8, 139.6, 129.9, 129.3, 128.5, 128.3, 121.0, 119.2, 117.8, 117.2, 111.8, 104.1, 64.7, 55.5, 34.6, 28.7. **HRMS m/z (ESI)** calcd. for $C_{24}H_{22}NO_4{}^+$ $(M + H)^+$ 388.1549, found 388.1552.

3-(6-methoxy-1-phenylisoquinolin-3-yl)propyl 3-chloro-2,2-dimethylpropanoate (2ai)

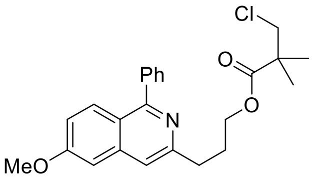

Following the general **procedure A** on 0.1 mmol scale with 4.0 V cell potential, and MeOH/DCM (3 ml/2 ml) as solvent, the substrate **2ai** was obtained as a colorless liquid in 58% yield (23.8 mg). **^{1}H NMR (400 MHz, Chloroform-*d*)** δ 7.93 (d, *J* = 9.0 Hz, 1H), 7.74 – 7.62 (m, 2H), 7.56 – 7.45 (m, 3H), 7.41 (s, 1H), 7.15 – 7.05 (m, 2H), 4.24 (t, *J* = 6.5 Hz, 2H), 3.97 (s, 3H), 3.65 (s, 2H), 3.07 (dd, *J* = 8.4, 6.7 Hz, 2H), 2.25 (p, *J* = 6.5 Hz, 2H), 1.32 (s, 6H). **^{13}C NMR (100 MHz, Chloroform-*d*)** δ 175.1, 160.6, 160.0, 153.9, 139.8, 139.6, 129.9, 129.4, 128.5, 128.3, 121.0, 119.3, 117.2, 104.1, 64.7, 55.5, 52.2, 44.7, 34.5, 28.6, 23.3. **HRMS m/z (ESI)** calcd. for $C_{24}H_{27}ClNO_3{}^+$ $(M + H)^+$ 412.1679, found 412.1680.

3-(6-methoxy-1-phenylisoquinolin-3-yl)propyl p-tolylcarbamate (2aj)

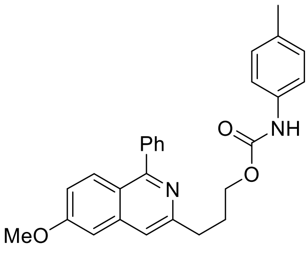

Following the general **procedure A** on 0.1 mmol scale with 4.0 V cell potential, and MeOH/DCM (4 ml/1 ml) as solvent, the substrate **2aj** was obtained as a colorless liquid in 42% yield (17.9 mg). **^{1}H NMR (400 MHz, Chloroform-*d*)** δ 7.93 (d, *J* = 9.2 Hz, 1H), 7.70 – 7.63 (m, 2H), 7.51 (ddt, *J* = 14.2, 10.1, 4.7 Hz, 3H), 7.42 (s, 1H), 7.26 (d, *J* = 8.1 Hz, 2H), 7.12 (d, *J* = 8.6 Hz, 2H), 7.10 – 7.02 (m, 2H), 6.61 (s, 1H), 4.29 (t,

J = 6.4 Hz, 2H), 3.96 (s, 3H), 3.13 – 3.05 (m, 2H), 2.32 (s, 3H), 2.26 (dt, J = 13.6, 6.6 Hz, 2H). **^{13}C NMR (100 MHz, Chloroform-*d*)** δ 1160.6, 160.0, 154.1, 153.8, 139.8, 139.6, 135.4, 132.9, 129.9, 129.5, 129.4, 128.5, 128.4, 121.0, 119.3, 118.7, 117.2, 104.1, 64.8, 55.5, 34.5, 29.1, 20.8. **HRMS m/z (ESI)** calcd. for $C_{27}H_{27}N_2O_3{}^+$ (M + H)$^+$ 427.2022, found 427.2023.

3-phenylisoquinoline (2ak)

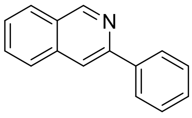

Following the general **procedure A** on 0.2 mmol scale with 4.0 V cell potential, and MeOH/DCM (4 ml/1 ml) as solvent, the substrate **2ak** was obtained as a white solid in 40% yield (16.4 mg). **^{1}H NMR (400 MHz, Chloroform-*d*)** δ 9.37 (s, 1H), 8.16 (d, J = 7.5 Hz, 2H), 8.10 (s, 1H), 8.02 (d, J = 8.1 Hz, 1H), 7.90 (d, J = 8.2 Hz, 1H), 7.72 (t, J = 7.5 Hz, 1H), 7.61 (t, J = 7.5 Hz, 1H), 7.54 (t, J = 7.6 Hz, 2H), 7.45 (t, J = 7.3 Hz, 1H). **^{13}C NMR (100 MHz, Chloroform-*d*)** δ 152.4, 151.3, 139.63, 136.7, 130.5, 128.8, 128.5, 127.8, 127.6, 127.1, 127.0, 126.9, 116.5. Spectroscopic data matches with previously reported data.[11]

1-(4-methoxyphenyl)-3-methylisoquinoline (2al)

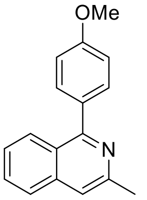

Following the general **procedure A** on 0.2 mmol scale with 4.0 V cell potential, and MeOH/DCM (4 ml/1 ml) as solvent, the substrate **2al** was obtained as a white solid in 42% yield (21.0 mg). mp 88−89°C. **^{1}H NMR (400 MHz, Chloroform-*d*)** δ 8.07 (d, J = 8.4 Hz, 1H), 7.79 (d, J = 8.2 Hz, 1H), 7.65 (ddd, J = 14.2, 7.4, 1.5 Hz, 3H), 7.50 – 7.42 (m, 2H), 7.13 – 7.05 (m, 2H), 3.92 (s, 3H), 2.76 (s, 3H). **^{13}C NMR (100 MHz, Chloroform-*d*)** δ 160.0, 150.8, 137.7, 132.3, 131.3, 129.8, 127.6, 126.3, 126.0, 125.0, 117.6, 113.8, 55.4, 24.4. **HRMS m/z (ESI)** calcd. for $C_{17}H_{16}NO^+$ (M + H)$^+$ 250.1232, found 250.1234.

6-methoxy-1,3,4-trimethylisoquinoline (2am)

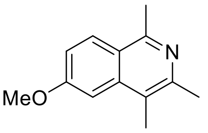

Following the general **procedure A** on 0.2 mmol scale with 4.0 V cell potential, and MeOH/DCM (4 ml/1 ml) as solvent, the substrate **2am** was obtained as a white solid in 66% yield (26.4 mg). mp: 104 – 105 °C. **^{1}H NMR (400 MHz, Chloroform-*d*)** δ 8.01 (d, J = 9.9 Hz, 1H), 7.16 (d, J = 7.4 Hz, 2H), 3.98 (s, 3H), 2.88 (s, 3H), 2.67 (s, 3H), 2.52 (s, 3H). **^{13}C NMR (100 MHz, Chloroform-*d*)** δ 160.4, 154.7, 148.3, 137.8, 128.0, 121.4, 121.1, 117.5, 101.6, 55.3, 22.9, 22.2, 14.1. **HRMS m/z (ESI)** calcd. for $C_{13}H_{16}NO^+$ (M + H)$^+$ 202.1232, found 202.1230.

6-methoxy-1-(4-methoxyphenyl)-3,4-dimethylisoquinoline (2an)

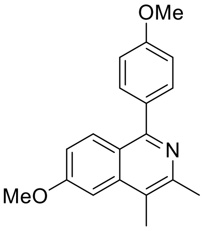

Following the general **procedure A** on 0.1 mmol scale with 4.0 V cell potential, and MeOH/DCM (4 ml/1 ml) as solvent, the substrate **2an** was obtained as a white solid in 51% yield (15.0 mg). mp: 100 – 101 °C. **^{1}H NMR (400 MHz, Chloroform-*d*)** δ 7.98 (d, J = 9.2 Hz, 1H), 7.79 – 7.53 (m, 2H), 7.21 (d, J = 2.4 Hz, 1H), 7.15 – 6.98 (m, 3H), 4.00 (s, 3H), 3.91 (s, 3H), 2.76 (s, 3H), 2.59 (s, 3H). **^{13}C NMR (100 MHz, Chloroform-*d*)** δ 160.3, 159.7, 157.0, 148.9, 138.5, 132.7, 131.2, 130.0, 121.6, 120.8, 117.6, 113.8, 101.3, 55.4, 55.3, 23.2, 14.2. **HRMS m/z (ESI)** calcd. for $C_{19}H_{20}NO_2{}^+$ (M + H)$^+$ 294.1494, found 294.1497.

1,3,4-trimethyl-6-(prop-2-yn-1-yloxy)isoquinoline (2ao)

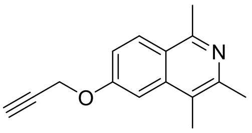

Following the general **procedure A** on 0.2 mmol scale with 4.0 V cell potential, and MeOH/DCM (4 ml/1 ml) as solvent, the substrate **2ao** was obtained as a white solid in 43% yield (19.2 mg). mp: 133 – 134 ºC. **^{1}H NMR (400 MHz, Chloroform-*d*)** δ 8.06 (d, *J* = 9.1 Hz, 1H), 7.33 (d, *J* = 2.3 Hz, 1H), 7.23 (dd, *J* = 9.1, 2.3 Hz, 1H), 4.89 (d, *J* = 2.3 Hz, 2H), 2.93 (s, 3H), 2.71 (s, 3H), 2.61 (t, *J* = 2.3 Hz, 1H), 2.53 (s, 3H). **^{13}C NMR (100 MHz, Chloroform-*d*)** δ 158.5, 154.7, 147.8, 137.8, 132.6, 128.3, 121.7, 117.8, 103.5, 77.8, 76.2, 55.9, 22.4, 21.8, 14.0. **HRMS m/z (ESI)** calcd. for $C_{15}H_{16}NO^+$ $(M + H)^+$ 226.1232, found 226.1231.

6-(benzyloxy)-1,3,4-trimethylisoquinoline (2ap)

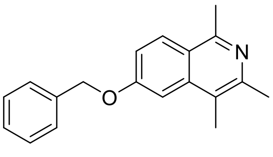

Following the general **procedure A** on 0.1 mmol scale with 4.0 V cell potential, and MeOH/DCM (3 ml/2 ml) as solvent, the substrate **2ap** was obtained as a white solid in 51% yield (10.5 mg). mp: 102 – 103 ºC. **^{1}H NMR (400 MHz, Chloroform-*d*)** δ 8.03 (d, *J* = 8.9 Hz, 1H), 7.52 (d, *J* = 7.4 Hz, 2H), 7.44 (t, *J* = 7.4 Hz, 2H), 7.38 (t, *J* = 7.2 Hz, 1H), 7.25 (dd, *J* = 11.4, 2.2 Hz, 2H), 5.25 (s, 2H), 2.89 (s, 3H), 2.68 (s, 3H), 2.49 (s, 3H). **^{13}C NMR (100 MHz, Chloroform-*d*)** δ 159.6, 154.6, 148.1, 137.8, 136.4, 128.7, 128.2, 128.1, 127.7, 121.5, 121.3, 117.9, 103.2, 70.1, 22.7, 22.0, 14.0. **HRMS m/z (ESI)** calcd. for $C_{19}H_{20}NO^+$ $(M + H)^+$ 278.1545, found 278.1547.

2,3,4,5,6-pentaphenylpyridine (2aq)

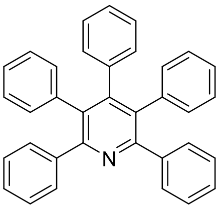

Following the general **procedure A** on 0.05 mmol scale with 5.5 V cell potential, and MeOH/Toluene (3 ml/2 ml) as solvent, the substrate **2aq** was obtained as a white solid in 44% yield (10.0 mg). **^{1}H NMR (400 MHz, Chloroform-*d*)** δ 7.44 (dd, *J* = 6.6, 2.9 Hz, 4H), 7.24 – 7.16 (m, 6H), 7.08 – 6.99 (m, 6H), 6.97 – 6.89 (m, 7H), 6.81 (dd, *J* = 6.4, 3.0 Hz, 2H). **^{13}C NMR (100 MHz, Chloroform-*d*)** δ 156.4, 150.2, 140.9, 138.4, 138.2, 133.7, 131.3, 130.4, 130.2, 127.5, 127.4, 127.3, 126.9, 126.2(2), 126.2(5). Spectroscopic data matches with previously reported data.[12]

6-methoxy-3-methyl-1,1'-biisoquinoline (2at)

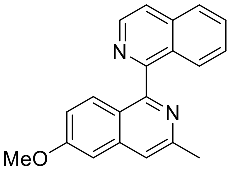

Following the general **procedure A** on 0.1 mmol scale with 4.0 V cell potential, and MeOH/DCM (3 ml/2 ml) as solvent, the substrate **2at** was obtained as a white solid in 60% yield (18.1 mg). mp: 210 – 211 ºC. **^{1}H NMR (400 MHz, Chloroform-*d*)** δ 8.72 (d, *J* = 5.7 Hz, 1H), 7.94 (d, *J* = 8.5 Hz, 1H), 7.81 (d, *J* = 5.7 Hz, 1H), 7.74 – 7.66 (m, 2H), 7.56 (s, 1H), 7.53 – 7.41 (m, 2H), 7.10 (d, *J* = 2.2 Hz, 1H), 7.01 (dd, *J* = 9.2, 2.3 Hz, 1H), 3.96 (s, 3H), 2.77 (s, 3H). **^{13}C NMR (100 MHz, Chloroform-*d*)** δ 160.8, 158.4, 157.0, 151.4, 142.1, 139.6, 136.8, 130.3, 128.8, 127.8, 127.4, 127.3, 126.9, 121.8, 120.9, 119.6, 118.5, 103.7, 55.5, 24.4. **HRMS m/z (ESI)** calcd. for $C_{20}H_{17}N_2O^+$ $(M + H)^+$ 301.1341, found 301.1346.

2.10. Electrochemical synthesis of diazine from pyrrole S-3

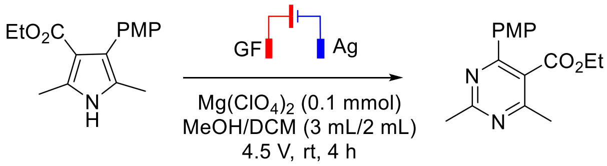

Supplementary Figure 36. Stepwise electrochemical synthesis of diazine from pyrrole **S-3.**

S-3 was prepared according to the known procedure[13]. As that shown in Supplementary Figure 36, following the general procedure A, a 10 mL three-necked heart-shaped flask was charged with **S-3** (0.1 mmol), $Mg(ClO_4)_2$ (0.1 mmol) and a magnetic stir bar. The flask was equipped with a rubber stopper, graphite felt (2 cm x 1 cm x 0.5 cm) as anode and Ag plate (2 cm x 1 cm) as cathode. The flask was evacuated and backfilled with ammonia gas for three times, then an ammonia gas balloon was connected to this flask via a needle. Next, 3 mL of anhydrous MeOH and 2 mL of anhydrous DCM was added via syringe. The electrolysis with 4.5 V cell potential was carried out at room temperature. After 4 hours, the mixture was concentrated under reduced pressure. The residue was purified by chromatography on silica gel to afford the desired product **2ar** (9 mg, 38%).

2.11. 3-step electrochemical synthesis of diazine from hantzsch ester

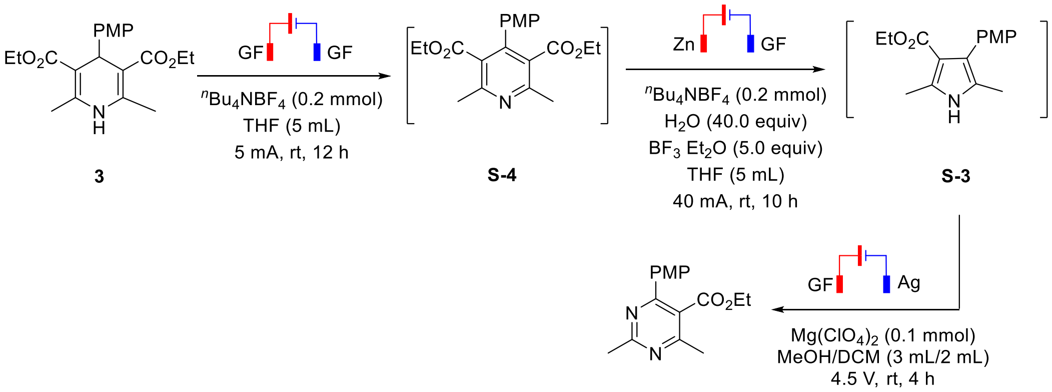

Supplementary Figure 37. Tandem electrochemical synthesis of diazine from **3.**

According to our previous work,[13] a 10 mL three-necked flask was charged with the diethyl 4-(4-methoxyphenyl)- 2,6-dimethyl-1,4-dihydropyridine-3,5-dicarboxylate (0.180 g, 0.5 mmol), $^{n}Bu_4NBF_4$ (65.8 mg, 0.2 mmol) and a magnetic stir bar. The flask was equipped with a rubber stopper, graphite felt (2 cm x 1 cm x 0.5 cm) as anode and cathode. The graphite felt anode attached to a platinum wire and cathode attached to a copper wire. The flask was evacuated and backfilled with argon for 3 times. Anhydrous THF (5 mL) was added and the mixture was stirred under 5 mA electrolysis for 12 h. Then the anode was changed to zinc plate and $BF_3{\cdot}Et_2O$ (0.400 g, 2.5 mmol, 5 equiv) and H_2O (0.36 g, 20 mmol, 40 equiv) was added. The mixture was stirred under 40 mA electrolysis for 10 h. When the reaction was finished, the mixture was extracted with EtOAc. The organic layers were washed with brine, dried over Na_2SO_4, filtered and concentrated. The residue was used directly in next step without further purification

Following the general procedure A, a 10 mL three-necked heart-shaped flask was charged with the residue, $Mg(ClO_4)_2$ (0.1 mmol) and a magnetic stir bar. The flask was equipped with a rubber stopper,

graphite felt (2 cm x 1 cm x 0.5 cm) as anode and Ag plate (2 cm x 1 cm) as cathode. The flask was evacuated and backfilled with ammonia gas for three times, then an ammonia gas balloon was connected to this flask via a needle. Next, 3 mL of anhydrous MeOH and 2 mL of anhydrous DCM was added via syringe. The electrolysis with 4.5 V cell potential was carried out at room temperature, After 4 hours, the mixture was concentrated under reduced pressure. The residue was purified by chromatography on silica gel to afford the desired product **2ar** (Supplementary Figure 37).

4-(4-methoxyphenyl)-2,6-dimethylpyrimidine-5-carboxylate (2ar)

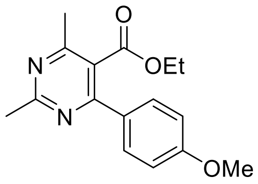

The substrate **2ar** was obtained as a colorless liquid in three steps with 12% yield (17.0 mg). **^{1}H NMR (400 MHz, Chloroform-*d*)** δ 7.65 (d, J = 8.8 Hz, 2H), 6.98 (d, J = 8.8 Hz, 2H), 4.25 (q, J = 7.1 Hz, 2H), 3.87 (s, 3H), 2.77 (s, 3H), 2.59 (s, 3H), 1.16 (t, J = 7.1 Hz, 3H). **^{13}C NMR (100 MHz, Chloroform-*d*)** δ 168.6, 167.6, 164.6, 162.8, 161.2, 130.2, 129.9, 122.6, 114.0, 61.8, 55.4, 26.17, 22.5, 13.8. **HRMS m/z (ESI)** calcd. for $C_{16}H_{19}N_2O_3{}^+$ (M + H)$^+$ 287.1396, found 287.1395.

2.12. Synthesis of moxaverine

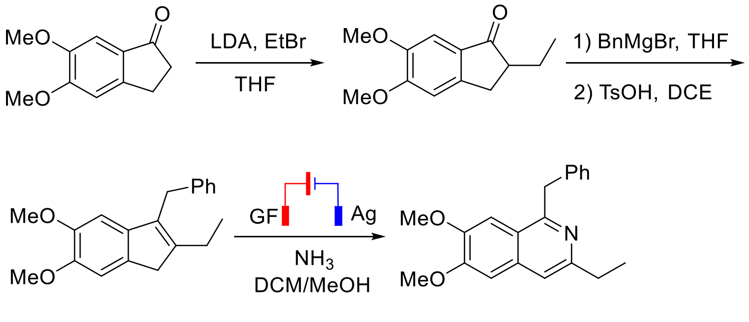

Supplementary Figure 38. Synthesis of Moxaverine with ammonia insertion.

As that shown in Supplementary Figure 38, to a solution of 5,6-dimethoxy-2,3-dihydro-1*H*-inden-1-one (1.920 g, 10 mmol) in anhydrous THF (40 mL) at −78 °C was added a solution of LDA (2 M in THF, 6 mL, 12 mmol, 1.2 equiv) dropwise under argon. The reaction mixture was stirred at −78 °C for 1 h, Then EtBr (1.296 g, 12 mmol, 1.2 equiv) and HMPA (3.580 g, 20 mmol, 2.0 equiv) was added dropwise. After completion of addition, the reaction mixture was brought to room temperature and stirred overnight. The reaction mixture was quenched with saturated aqueous NH_4Cl solution and extracted with ethyl acetate for three times. The combined organic layers were washed with water, dried ($MgSO_4$), filtered, and concentrated. The residue was purified by flash column chromatography to afford the white liquid (0.900 g, 41%).

A flask charged with 2-ethyl-5,6-dimethoxy-2,3-dihydro-1*H*-inden-1-one (0.440 g, 2 mmol) in 10 mL of anhydrous THF was added BnMgBr (4 mL, 1.0 M in THF, 4 mmol, 2.0 equiv) at 0 ℃ under argon. After completion of addition, the reaction mixture was brought to room temperature. After the completion of reaction monitored with TLC and GC-MS, the mixture was quenched with saturated aqueous NH_4Cl solution and extracted with ethyl acetate for three times. The combined organic layers were washed with water, dried over $MgSO_4$, filtered, and concentrated in vacuo. The resulting liquid was

dissolved in 1,2-dichloroethane (10 mL). TsOH (10.0 mg) was added and the mixture was refluxed overnight. After cooling to room temperature, the reaction mixture was concentrated and purified by flash column chromatography to afford the white solid (0.455 g, 78%).

A 10 mL three-necked heart-shaped flask was charged with the substrate 3-benzyl-2-ethyl-5,6-dimethoxy-1*H*-indene (29.4 mg, 0.1 mmol), $Mg(ClO_4)_2$ (22.0 mg, 0.1 mmol) and a magnetic stir bar. The flask was equipped with a rubber stopper, graphite felt (2 cm x 1 cm x 0.5 cm) as anode and Ag plate (2 cm x 1 cm) as cathode. The flask was evacuated and backfilled with ammonia gas for three times, then an ammonia gas balloon was connected to this flask via a needle. Next, 3 mL of anhydrous MeOH and 2 mL of anhydrous DCM were added via syringe. The electrolysis with constant cell potential (4.5 V) was carried out at room temperature. After 3 hours, the mixture was concentrated under reduced pressure. The residue was purified by chromatography on silica gel to afford the desired product (19.0 mg, 62%).

3-benzyl-2-ethyl-5,6-dimethoxy-1*H*-indene (1ar)

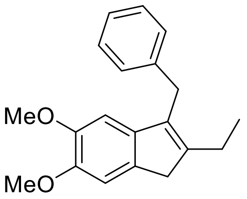

The substrate **1ar** was obtained as a white solid. mp: 57 – 58 °C. **^{1}H NMR (400 MHz, Chloroform-*d*)** 7.33 – 7.14 (m, 5H), 7.05 (s, 1H), 6.66 (s, 1H), 3.94 – 3.87 (m, 5H), 3.78 (s, 3H), 3.37 (s, 2H), 2.56 (q, *J* = 7.6 Hz, 2H), 1.19 (t, *J* = 7.6 Hz, 3H). **^{13}C NMR (100 MHz, Chloroform-*d*)** δ 148.0, 146.4, 144.8, 139.9, 139.4, 134.7, 134.0, 128.4, 128.3, 125.9, 108.0, 103.3, 56.3, 56.1, 39.7, 31.4, 21.8, 14.6. **HRMS m/z (ESI)** calcd. for $C_{20}H_{23}O_2{}^+$ $(M + H)^+$ 295.1698, found 295.1697.

3-ethyl-6,7-dimethoxy-1-phenethylisoquinoline (2ar)

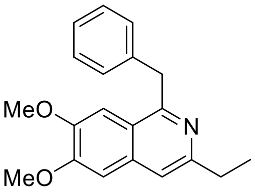

The substrate **2ar** was obtained as a white solid. **^{1}H NMR (400 MHz, Chloroform-*d*)** δ 7.32 – 7.22 (m, 6H), 7.17 (t, *J* = 6.8 Hz, 1H), 7.00 (s, 1H), 4.61 (s, 2H), 3.99 (s, 3H), 3.85 (s, 3H), 2.98 (q, *J* = 7.6 Hz, 2H), 1.42 (t, *J* = 7.6 Hz, 3H). **^{13}C NMR (100 MHz, Chloroform-*d*)** δ 157.1, 154.8, 152.3, 149.1, 139.9, 134.3, 128.5, 128.5, 126.1, 121.1, 115.4, 104.9, 104.3, 55.9, 55.8, 42.8, 31.1, 14.3. Spectroscopic data matches with previously reported data.[14]

2.13. Synthesis of 6-methoxy-3-methyl -[1,1'- biisoquinoline] 2,2'-dioxide

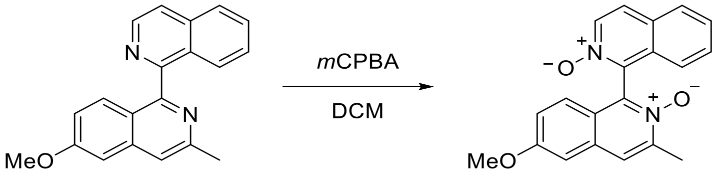

Supplementary Figure 39. Synthesis of 6-methoxy-3-methyl -[1,1'- biisoquinoline] 2,2'-dioxide.

As that shown Supplementary Figure 39, to a solution of 6-methoxy-3-methyl-1,1'-biisoquinoline (0.255 g, 0.85 mmol) in DCM (10 mL) was added 3-chloroperbenzoic acid (0.688 g, 3.4 mmol, 4.0 equiv) portion-wise at 0 °C, and then stirred at room temperature for 24 h. The reaction mixture was quenched with dimethyl sulfide (1 mL) and concentrated. The residue was triturated twice in ethyl acetate, filtered, and dried to afford the desired product.[15]

6-methoxy-3-methyl-[1,1'-biisoquinoline] 2,2'-dioxide (2at)

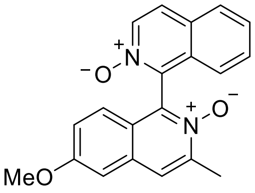

The substrate **2at** was obtained as a white solid in 71% yield (0.20 g). mp > 300 °C. **^{1}H NMR (400 MHz, DMSO-*d*$_6$)** δ 8.39 (d, *J* = 7.2 Hz, 1H), 8.21 – 8.02 (m, 3H), 7.65 (t, *J* = 7.5 Hz, 1H), 7.53 (t, *J* = 7.6 Hz, 1H), 7.45 (d, *J* = 2.1 Hz, 1H), 7.13 (dd, *J* = 9.2, 2.2 Hz, 1H), 6.93 (dd, *J* = 23.1, 8.7 Hz, 2H), 3.91 (s, 3H), 2.53 (s, 3H). **^{13}C NMR (100 MHz, DMSO-*d*$_6$)** 159.4, 146.2, 137.9, 137.7, 136.8, 130.5, 130.2, 129.3, 128.7, 128.5, 128.0, 125.8, 125.1, 123.6, 123.6, 121.9, 106.0, 56.1, 17.8. **HRMS m/z (ESI)** calcd. for $C_{20}H_{17}N_2O_3{}^{+}$ (M + H) $^{+}$ 333.1239, found 333.1232. **HPLC analysis**: Venusil CO (hexane/Isopropanol = 55/45, flow rate 0.5 mL/min, λ = 254 nm), ^{t}R (*R*)-isomer = 37.8 min, (*S*)-isomer = 55.4 min (Supplementary Figure 34, the (*R*) or (*S*)-isomer was judged according to the known literature[15]).

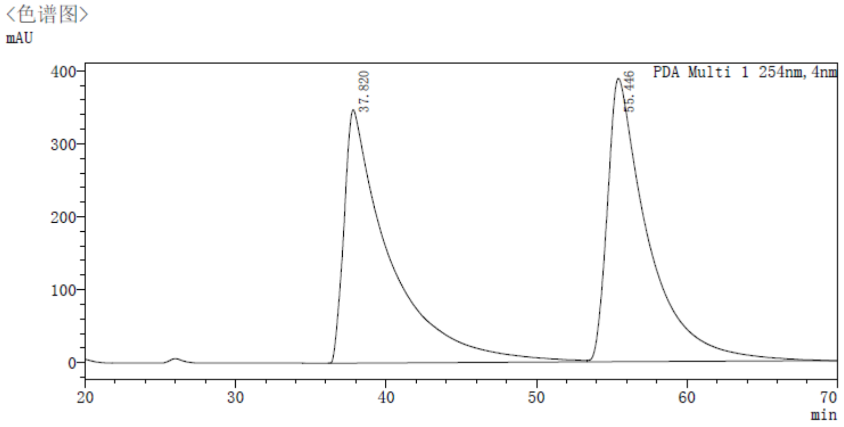

〈峰表〉
PDA Ch1 254nm

峰号	保留时间	高度	面积	面积%
1	37.820	347307	73223570	50.106
2	55.446	388270	72912825	49.894
总计		735577	146136395	100.000

Supplementary Figure 40. Chiral HPLC spectrum of racemic **2at** at 30 °C

HPLC analysis: Venusil CO (hexane/Isopropanol = 55/45, flow rate 0.5 mL/min, λ = 254 nm), ^{t}R (*R*)-isomer = 190.1 min, (*S*)-isomer = 208.2 min. 27 min – 34 min (heated up form 30 ºC to 45 ºC), 34 min – 154 min (maintain 45 ºC for two hours), 154 min – 180 min (cooled down form 45 ºC to 30 ºC), Supplementary Figure 41.

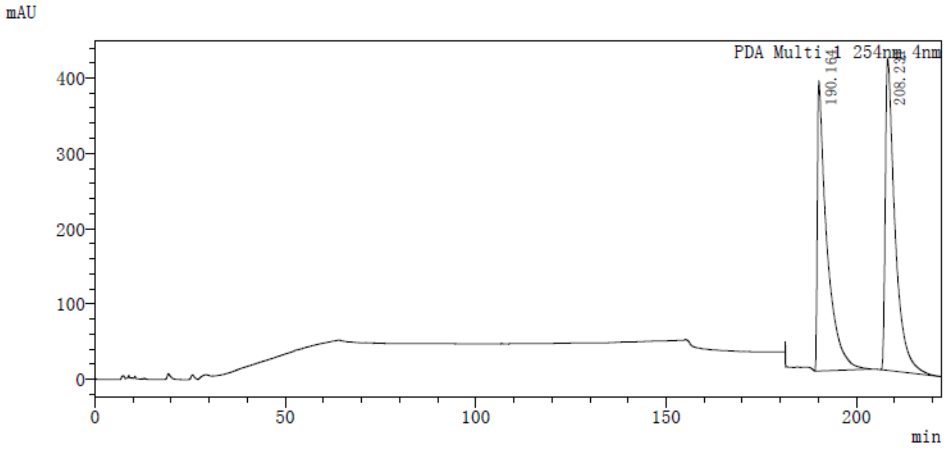

〈峰表〉

PDA Ch1 254nm

峰号	保留时间	面积	高度	面积%
1	190.164	67608770	384790	47.740
2	208.231	74008870	413570	52.260
总计		141617640	798361	100.000

Supplementary Figure 41. Chiral HPLC spectrum of racemic **2at** with heating sequence.

2.14 NMR spectra

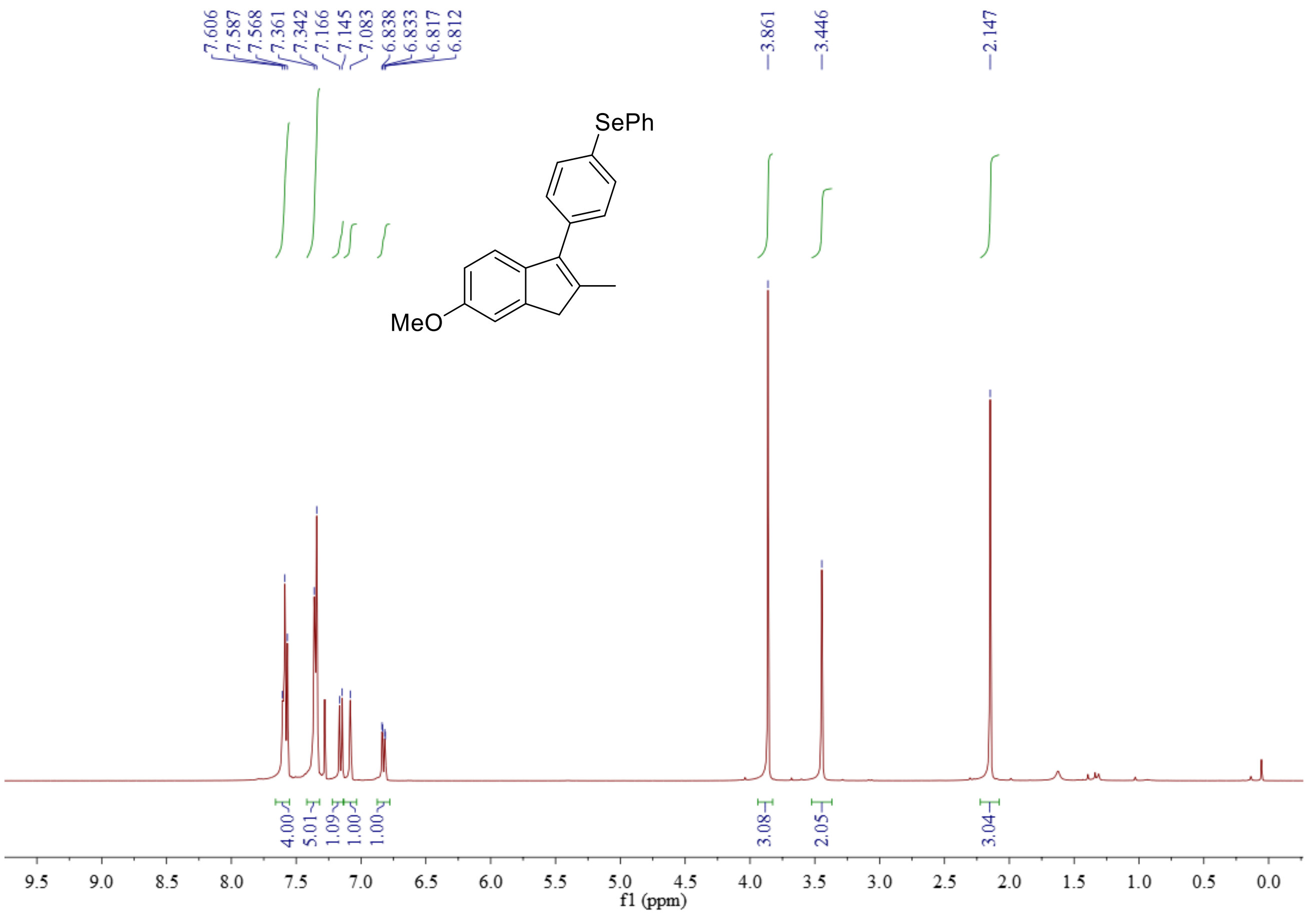

Supplementary Figure 42. ^{1}H NMR (400 MHz, $CDCl_3$) of **1m**

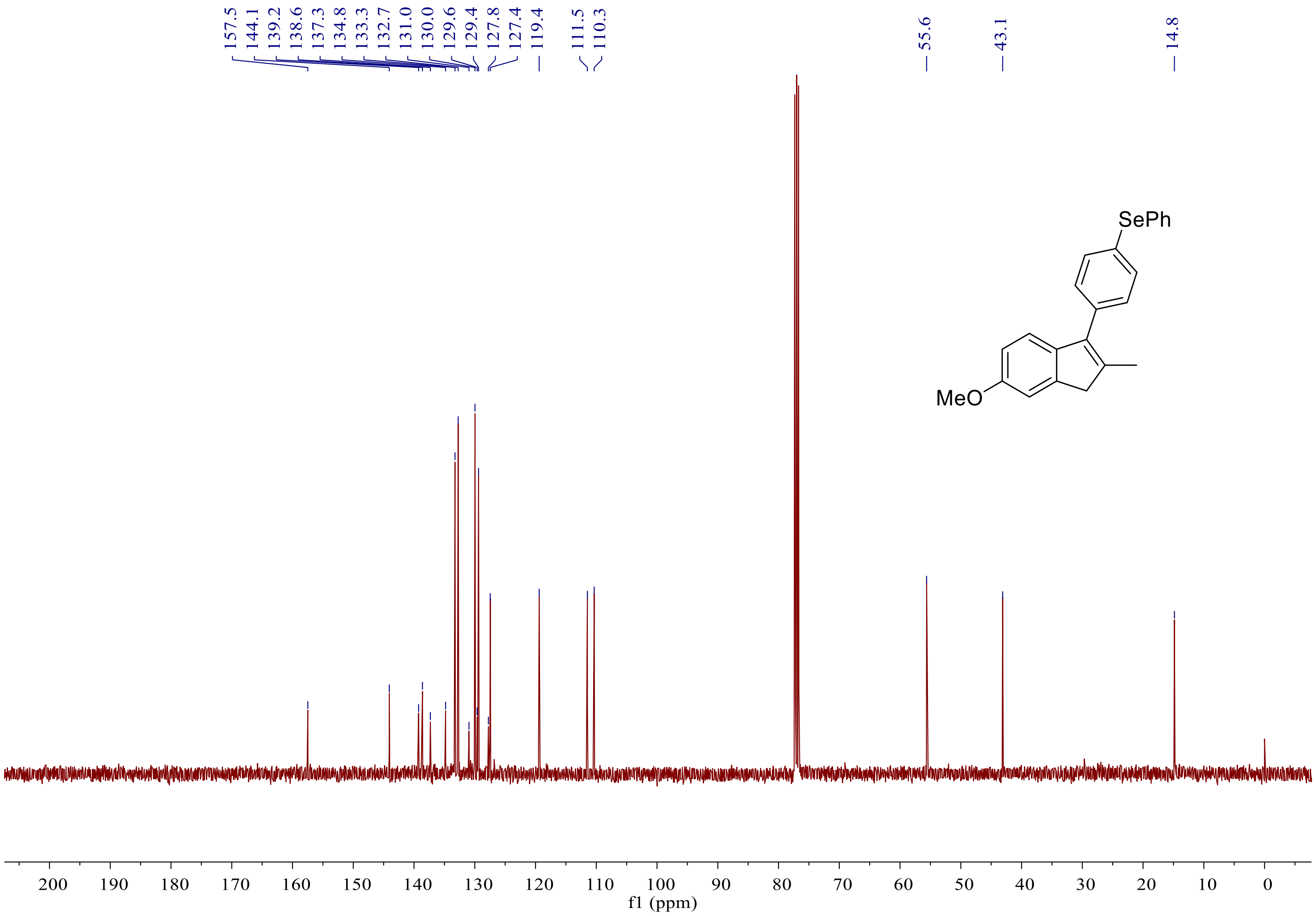

Supplementary Figure 43. ^{13}C NMR (100 MHz, $CDCl_3$) of **1m**

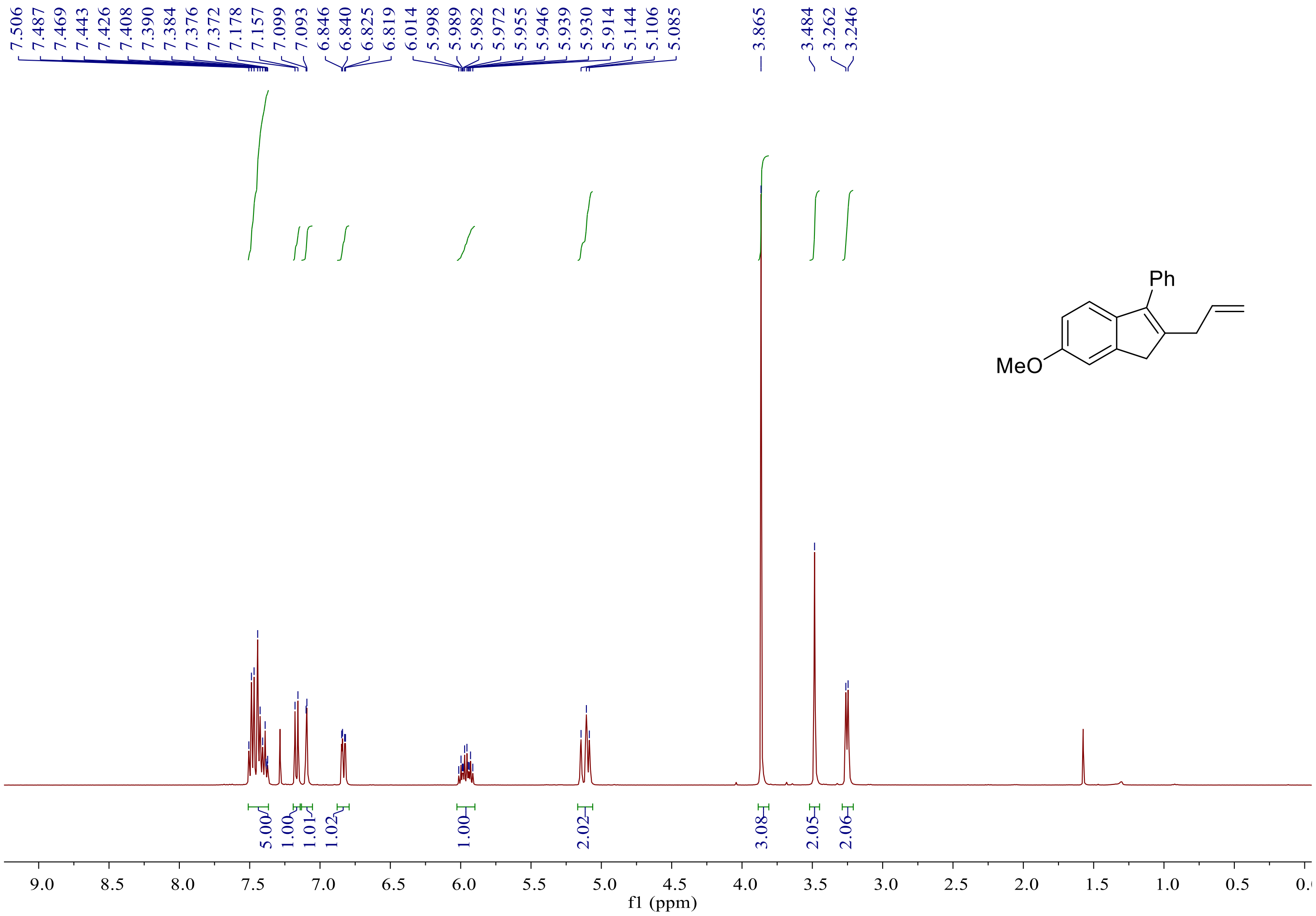

Supplementary Figure 44. ^{1}H NMR (400 MHz, $CDCl_3$) of **1u**

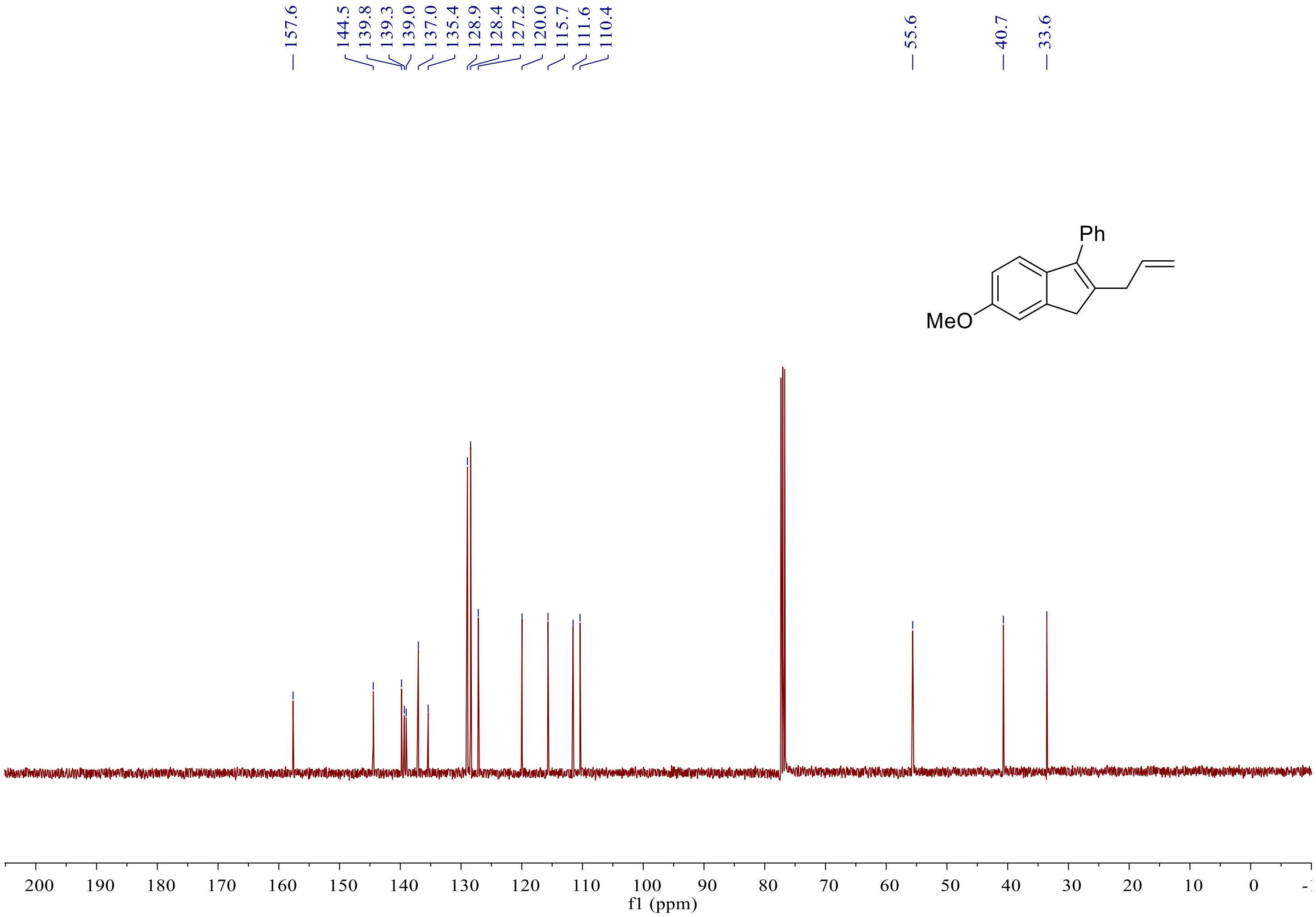

Supplementary Figure 45. ^{13}C NMR (100 MHz, $CDCl_3$) of **1u**

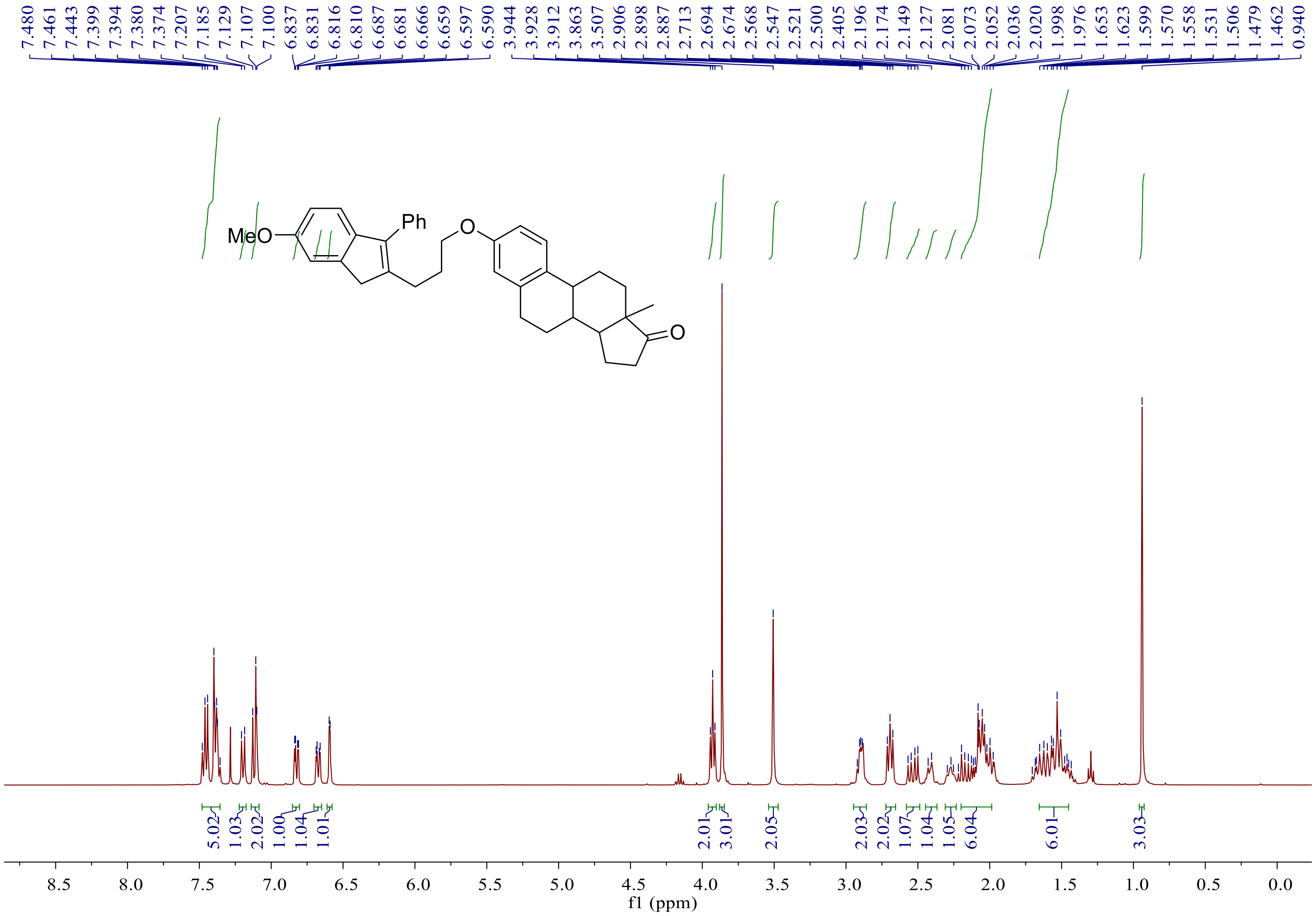

Supplementary Figure 46. ^{1}H NMR (400 MHz, $CDCl_3$) of **1v**

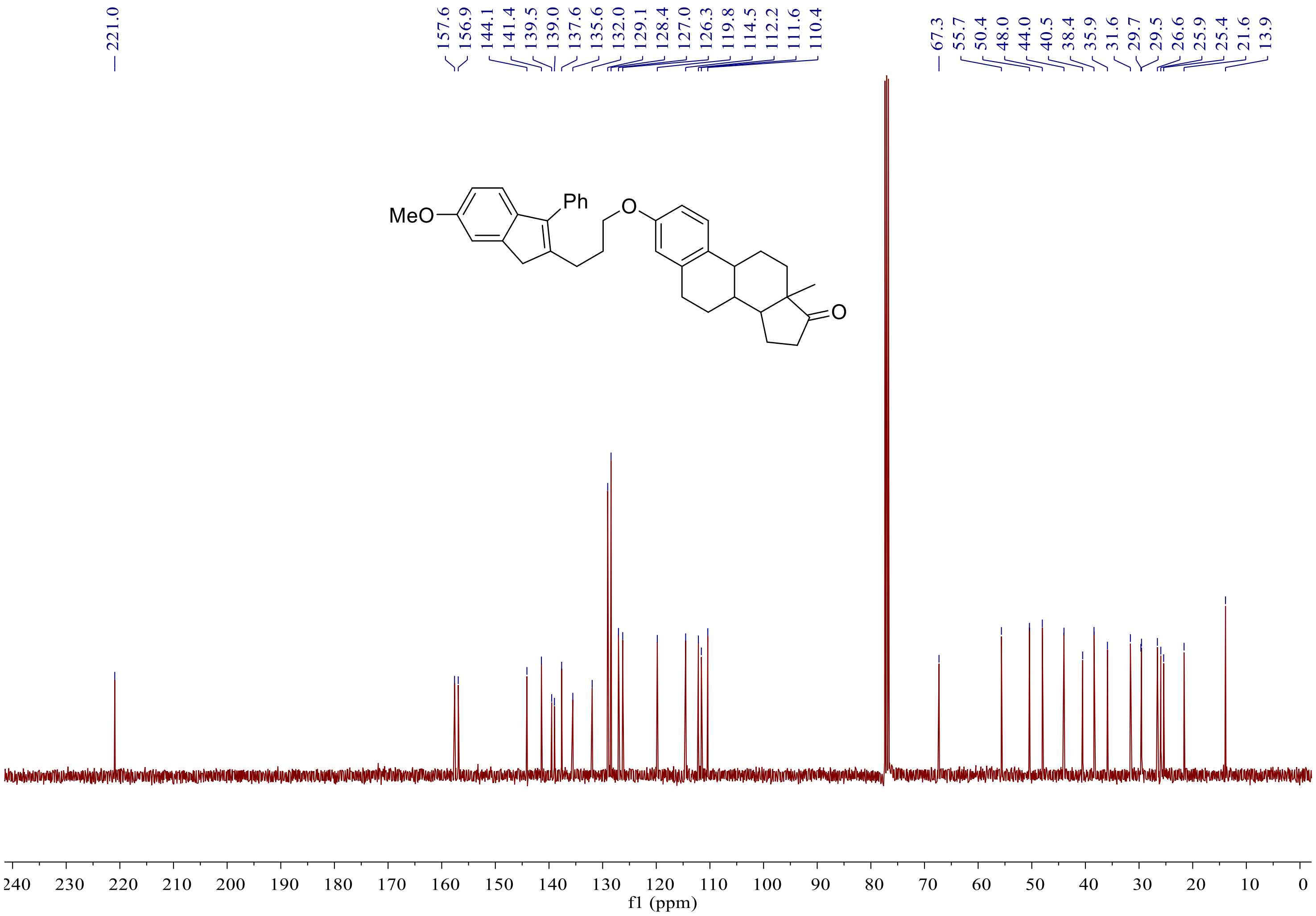

Supplementary Figure 47. ^{13}C NMR (100 MHz, $CDCl_3$) of **1v**

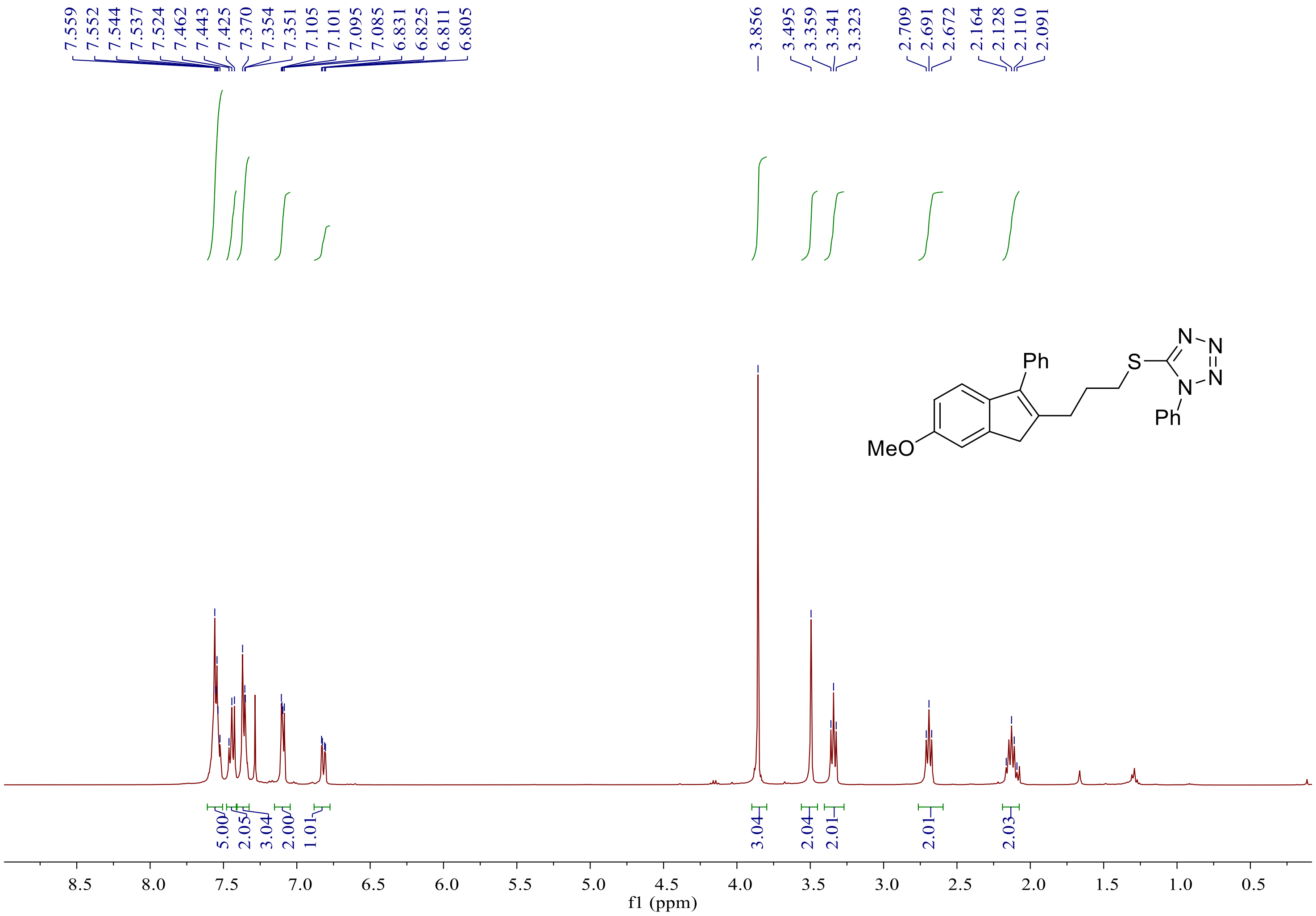

Supplementary Figure 48. ^{1}H NMR (400 MHz, $CDCl_3$) of **1w**

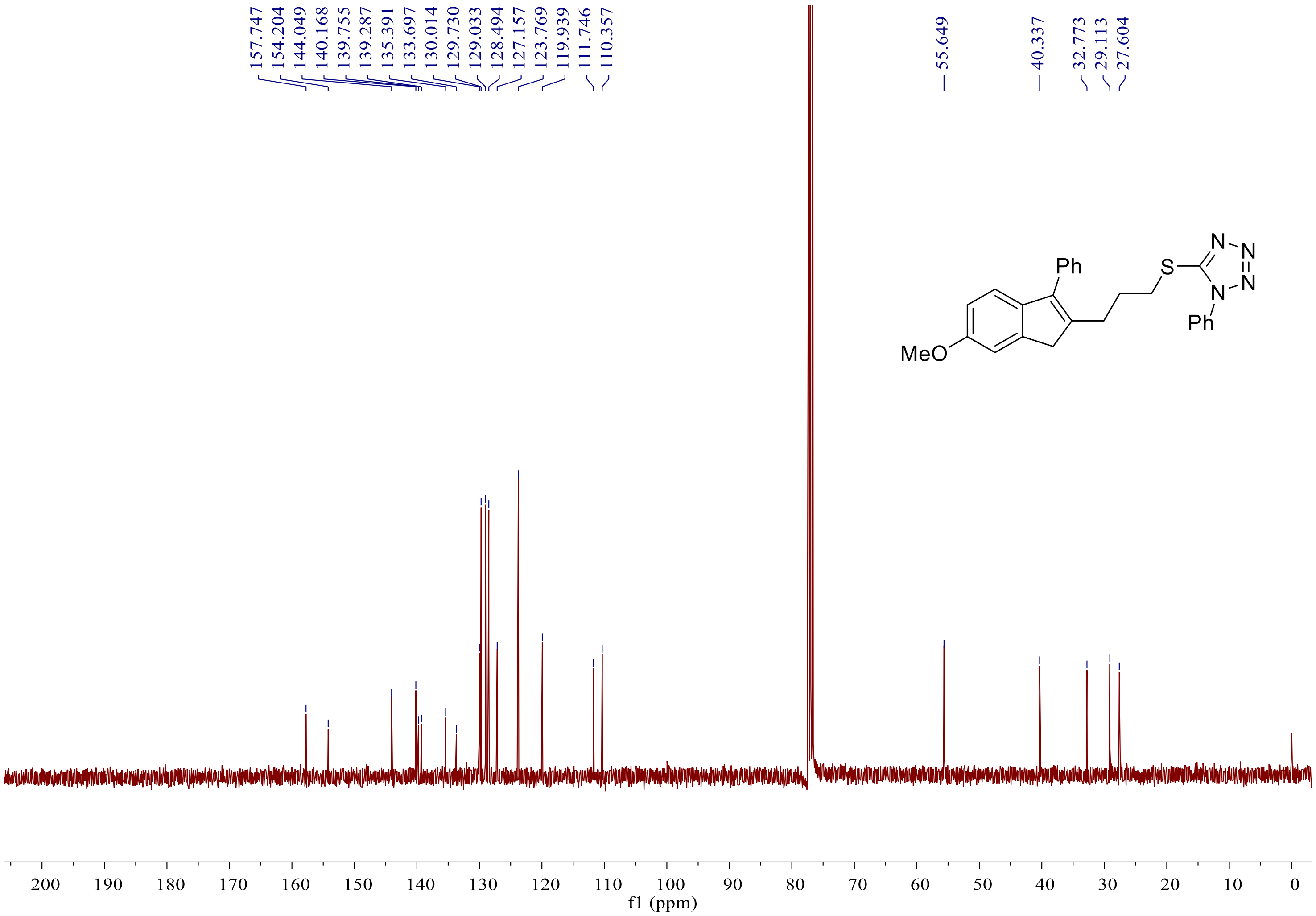

Supplementary Figure 49. ^{13}C NMR (100 MHz, $CDCl_3$) of **1w**

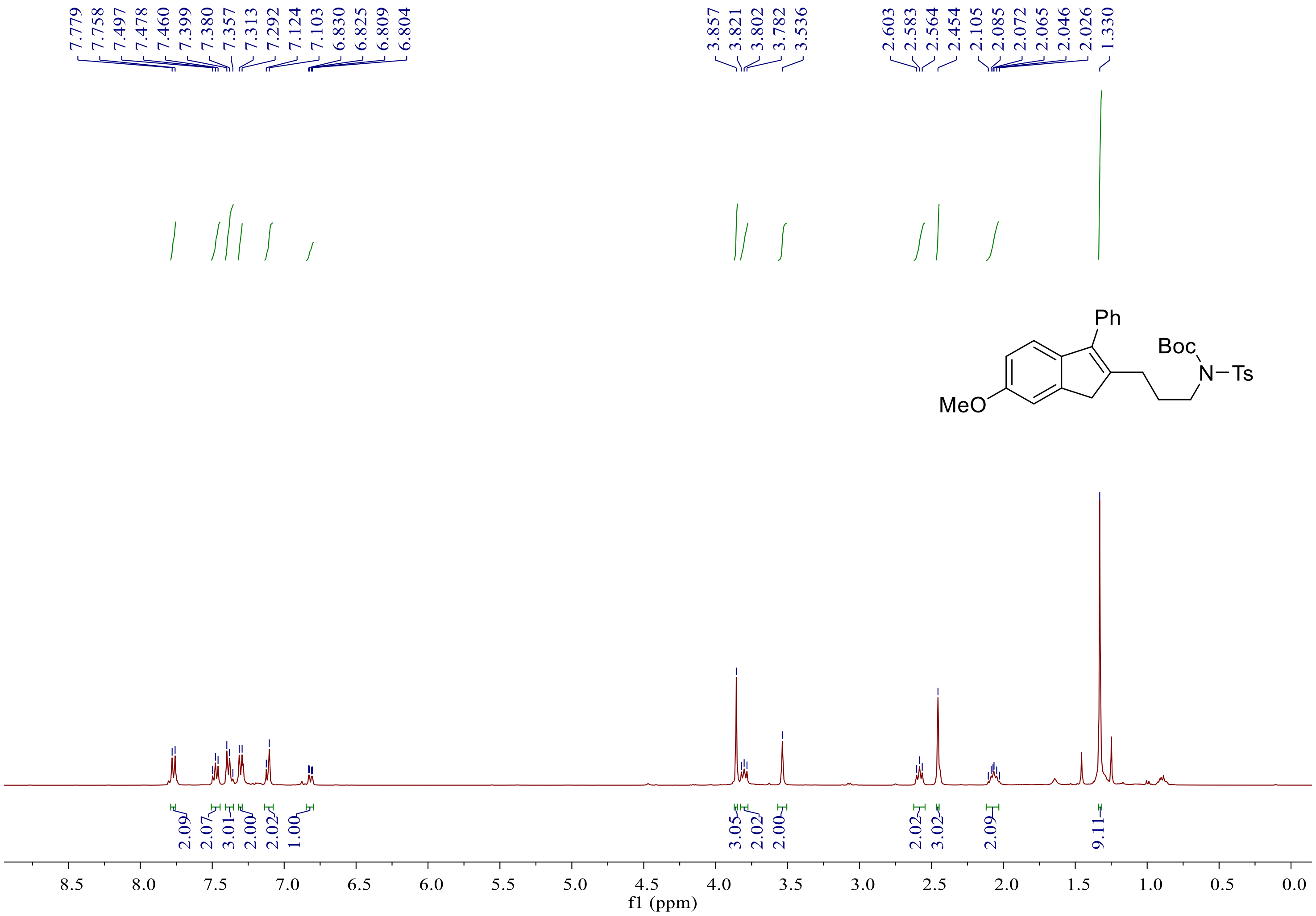

Supplementary Figure 50. ^{1}H NMR (400 MHz, $CDCl_3$) of **1x**

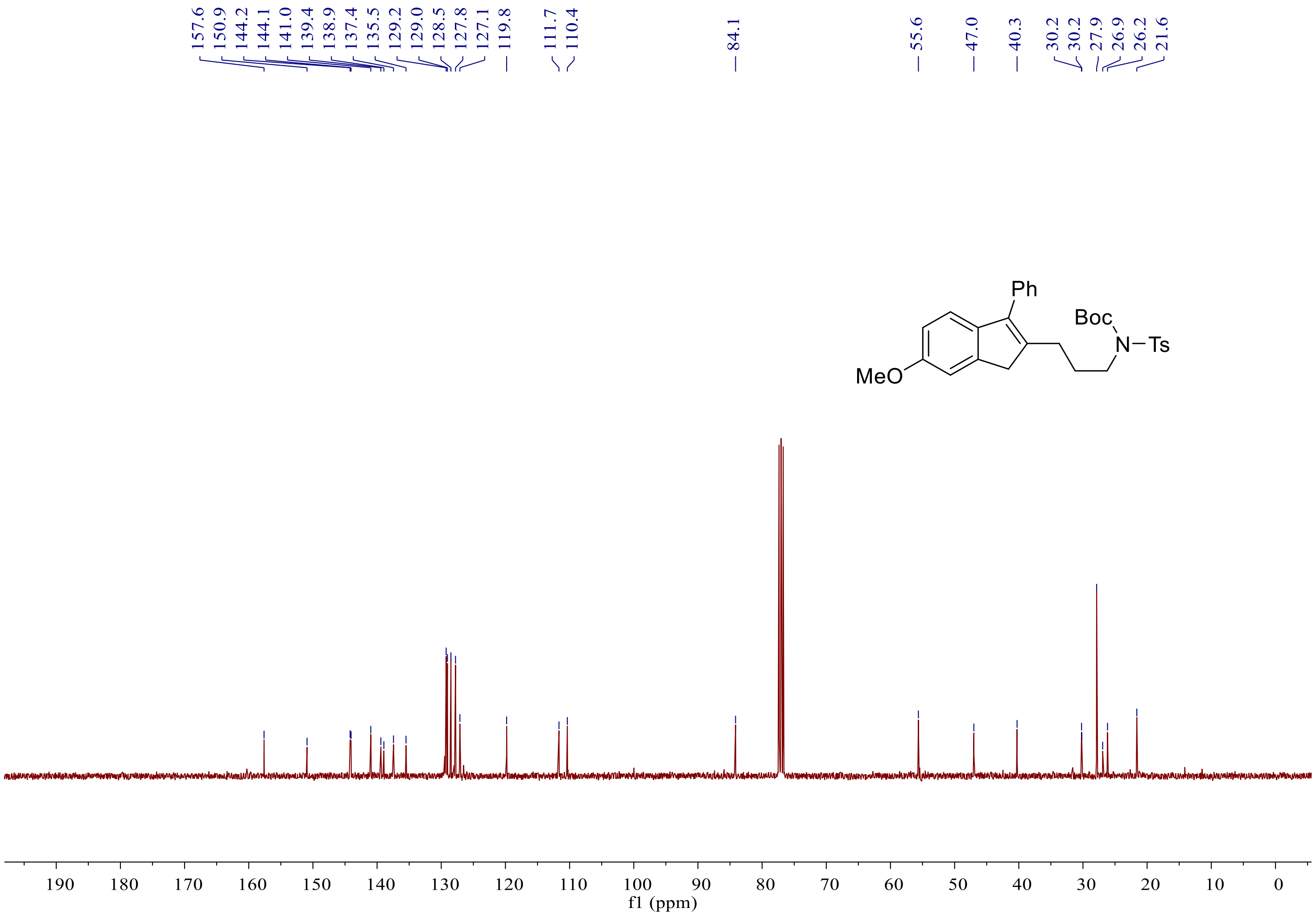

Supplementary Figure 51. ^{13}C NMR (100 MHz, $CDCl_3$) of **1x**

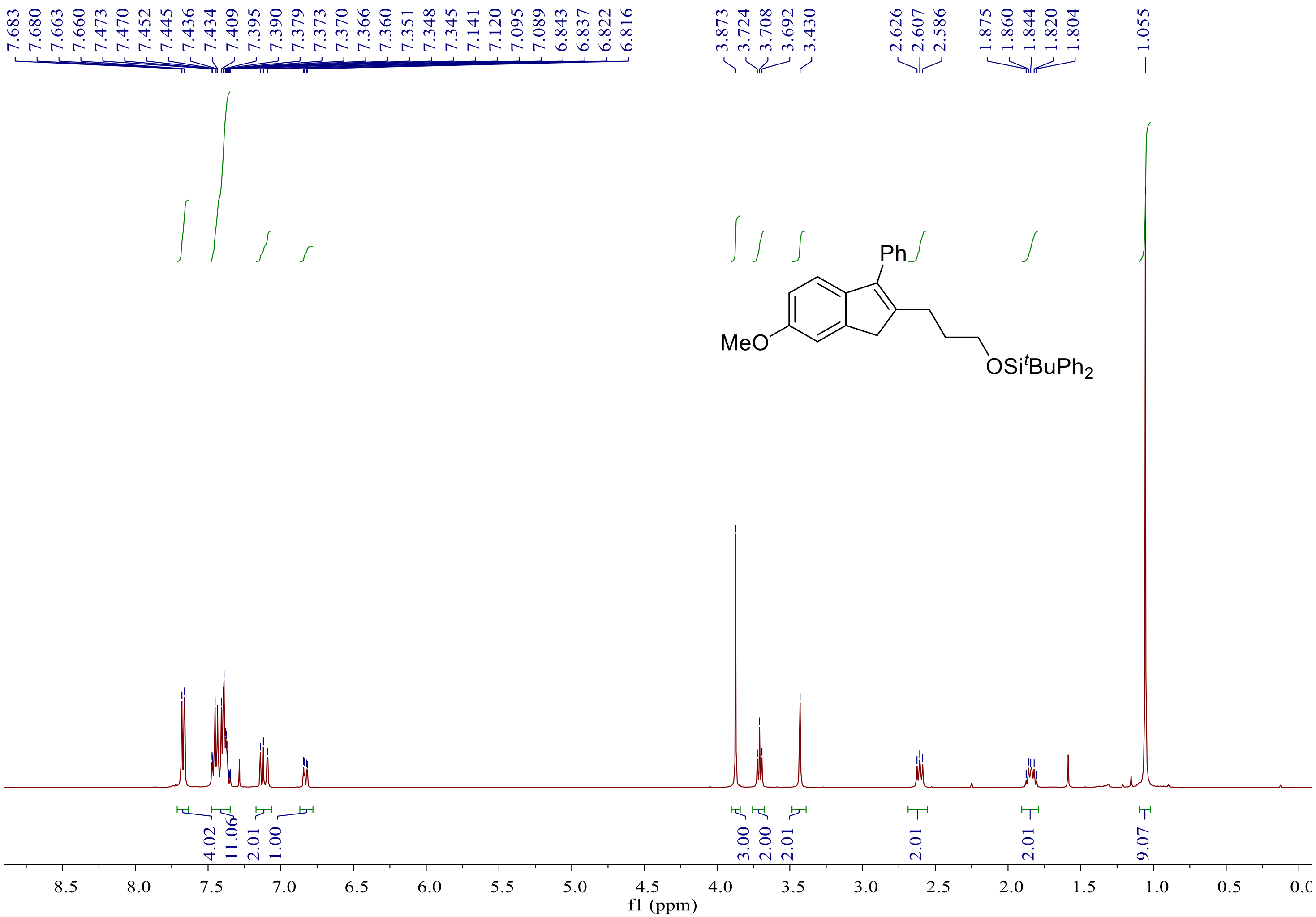

Supplementary Figure 52. ^{1}H NMR (400 MHz, $CDCl_3$) of **1y**

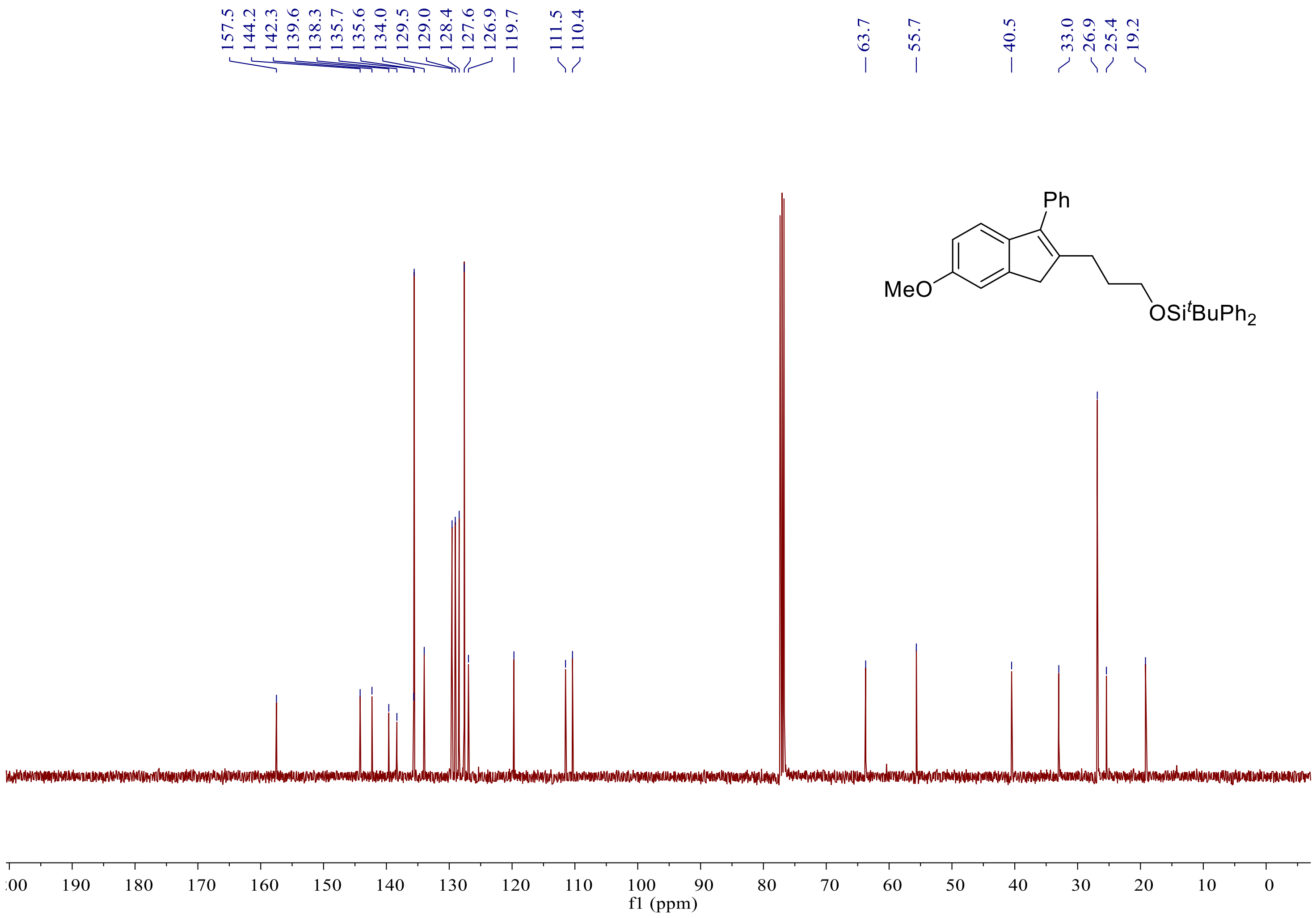

Supplementary Figure 53. ^{13}C NMR (100 MHz, $CDCl_3$) of **1y**

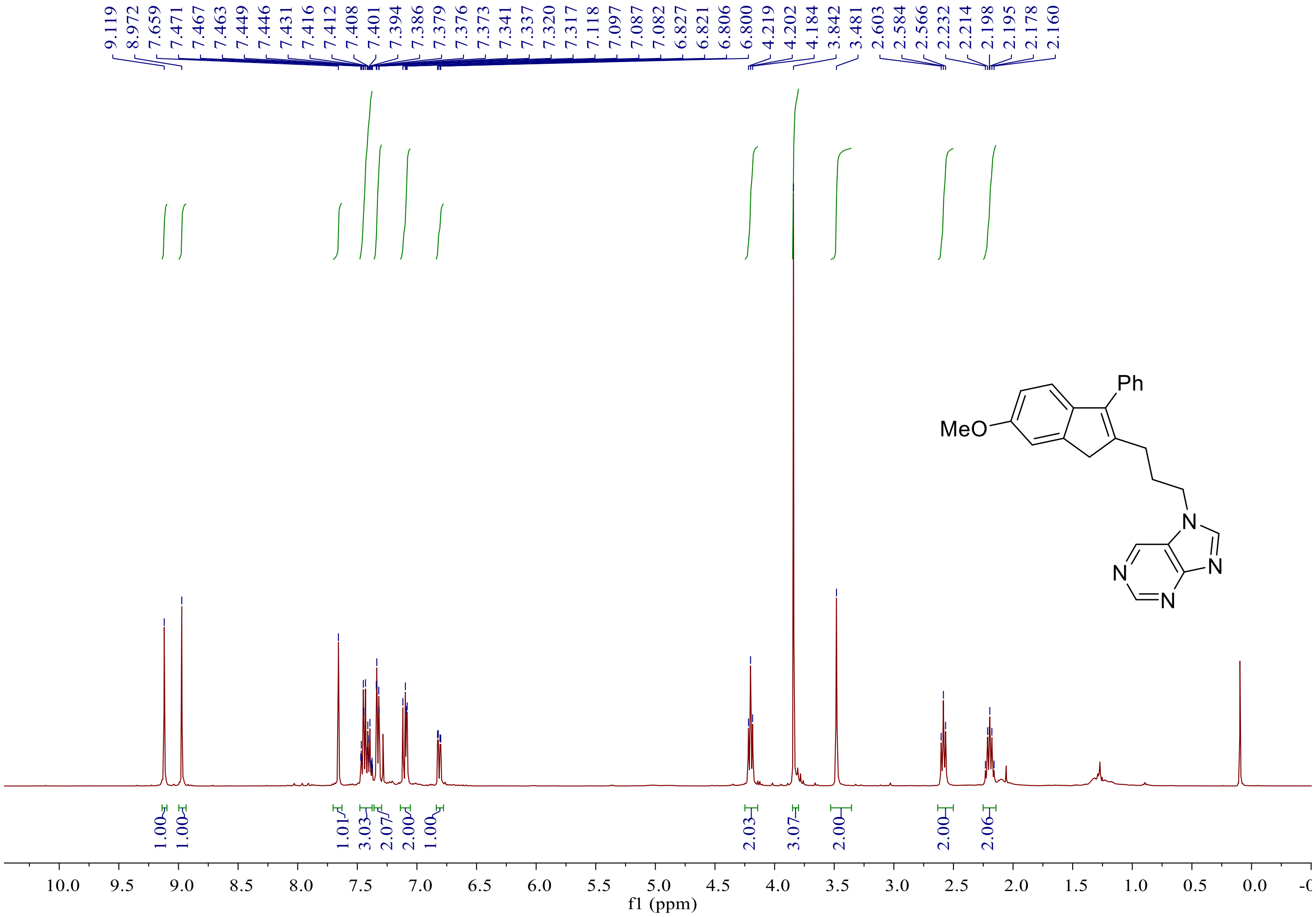

Supplementary Figure 54. ^{1}H NMR (400 MHz, $CDCl_3$) of **1z**

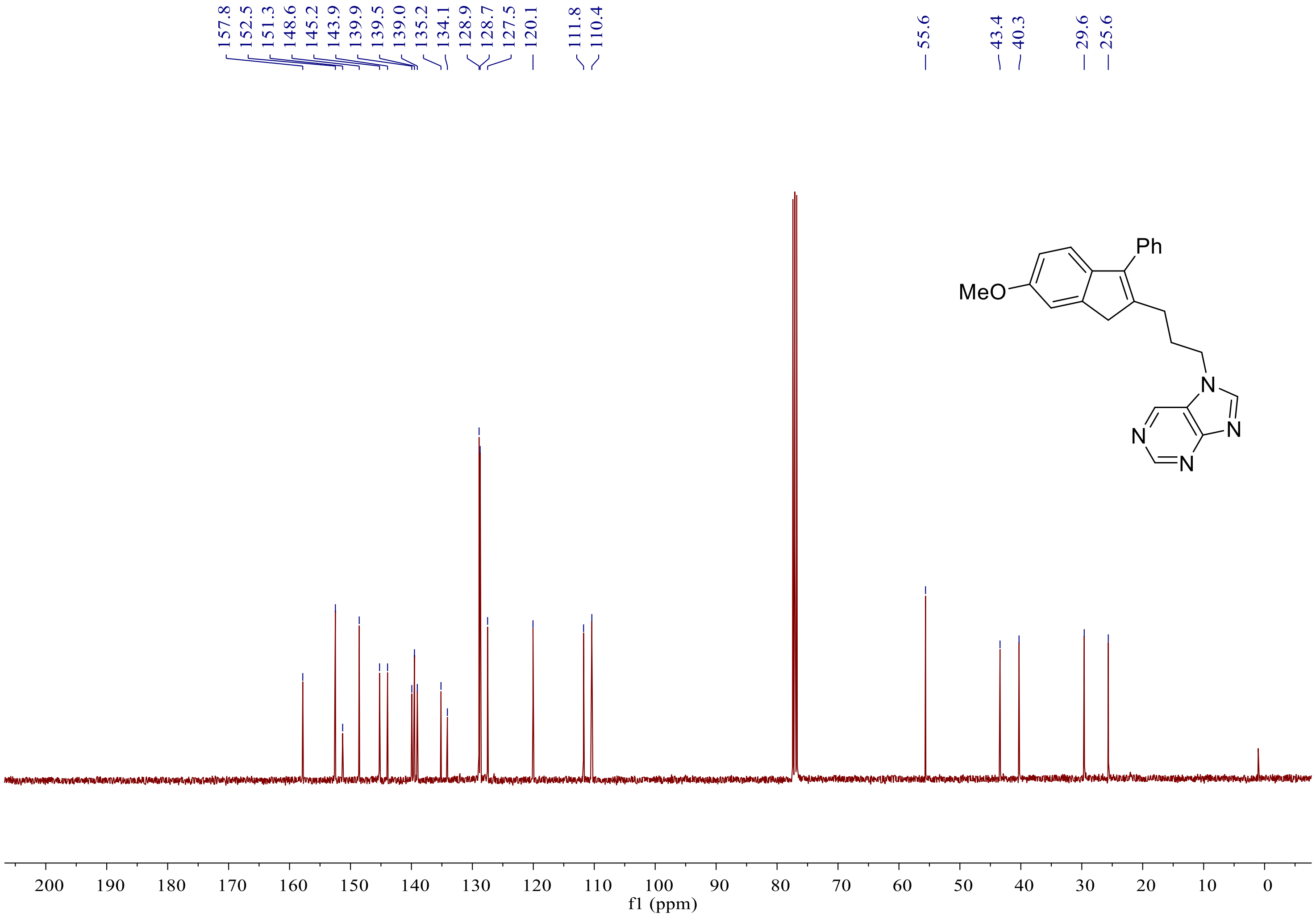

Supplementary Figure 55. ^{13}C NMR (100 MHz, $CDCl_3$) of **1z**

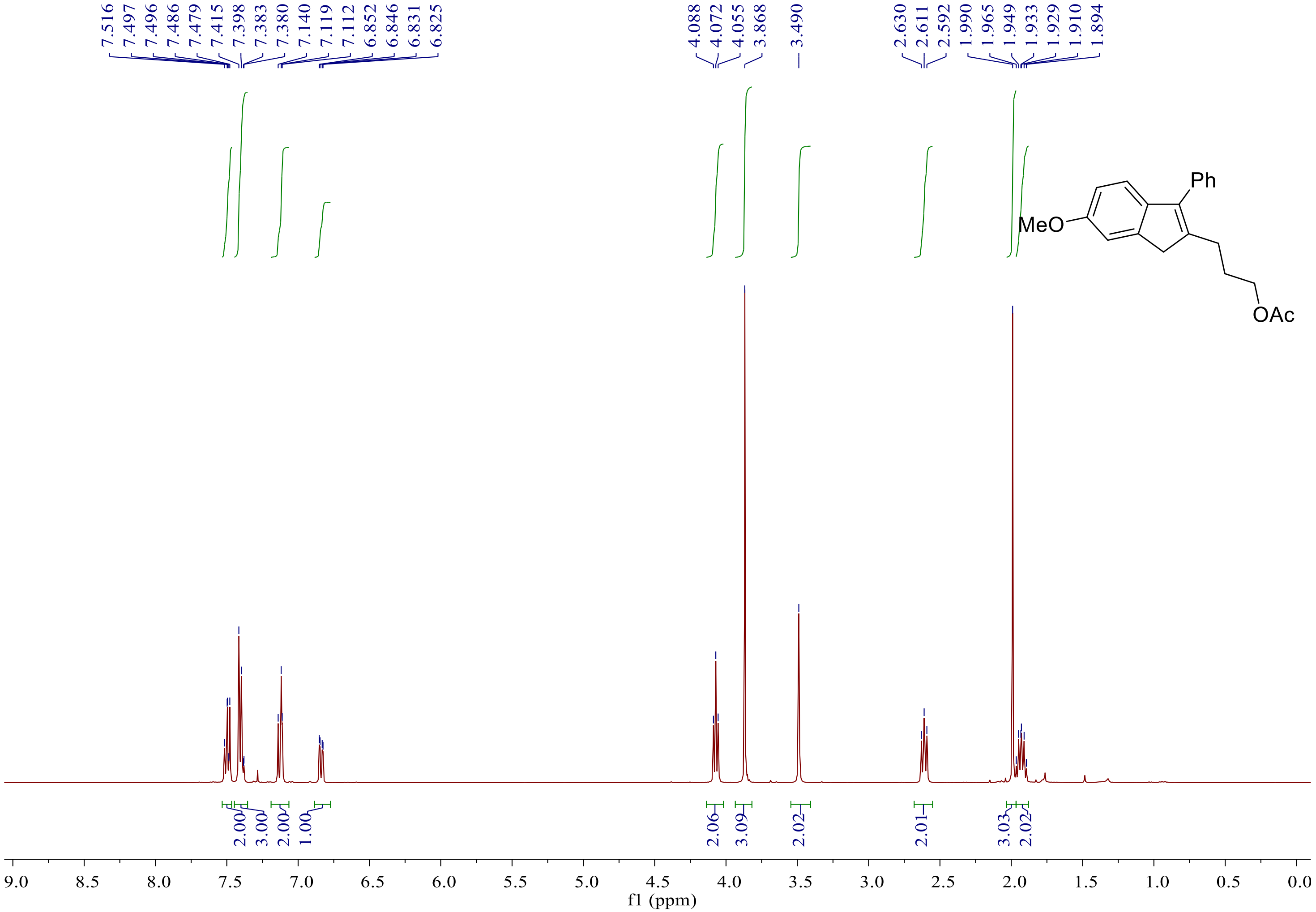

Supplementary Figure 56. ^{1}H NMR (400 MHz, $CDCl_3$) of **1aa**

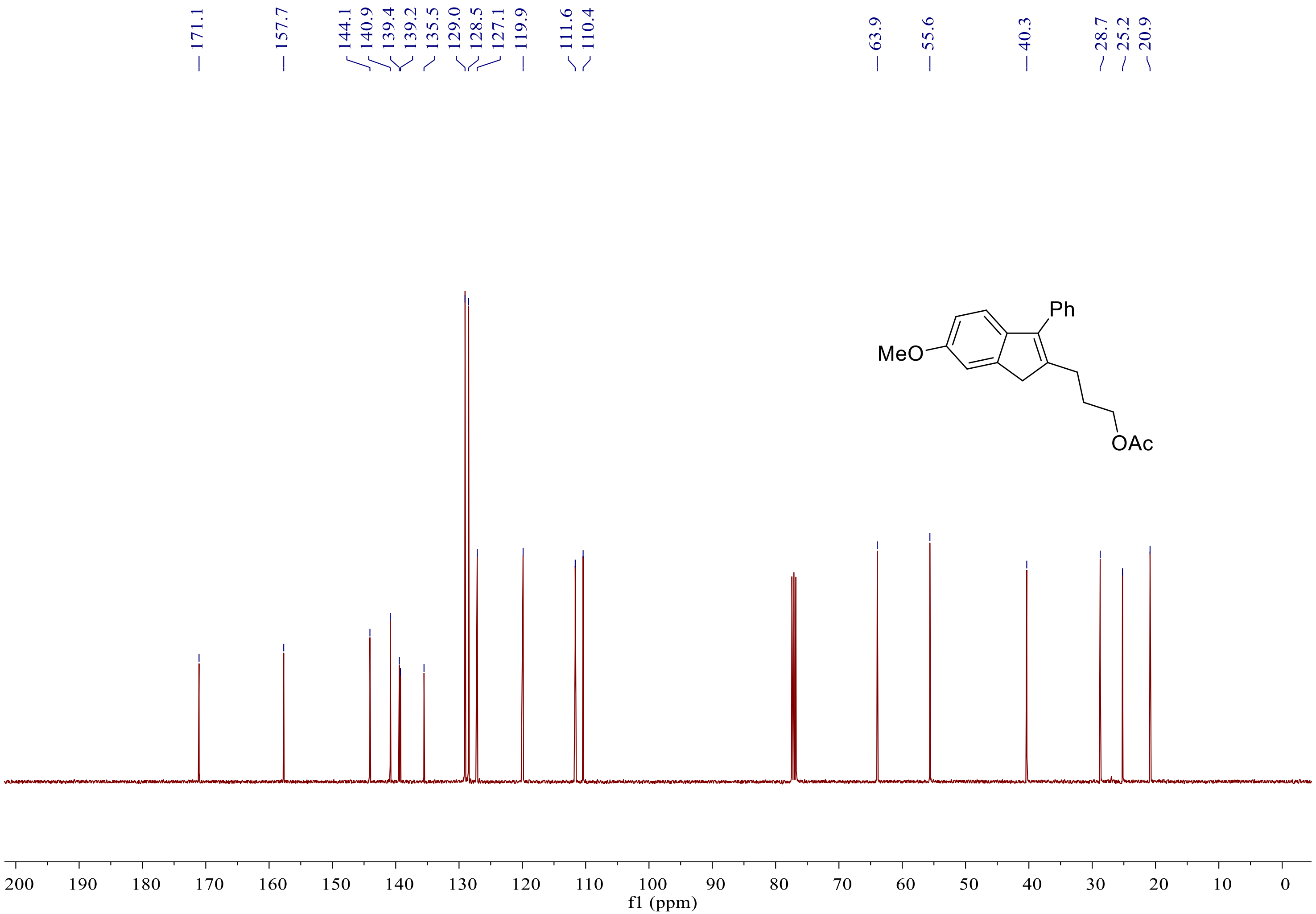

Supplementary Figure 57. ^{13}C NMR (100 MHz, $CDCl_3$) of **1aa**

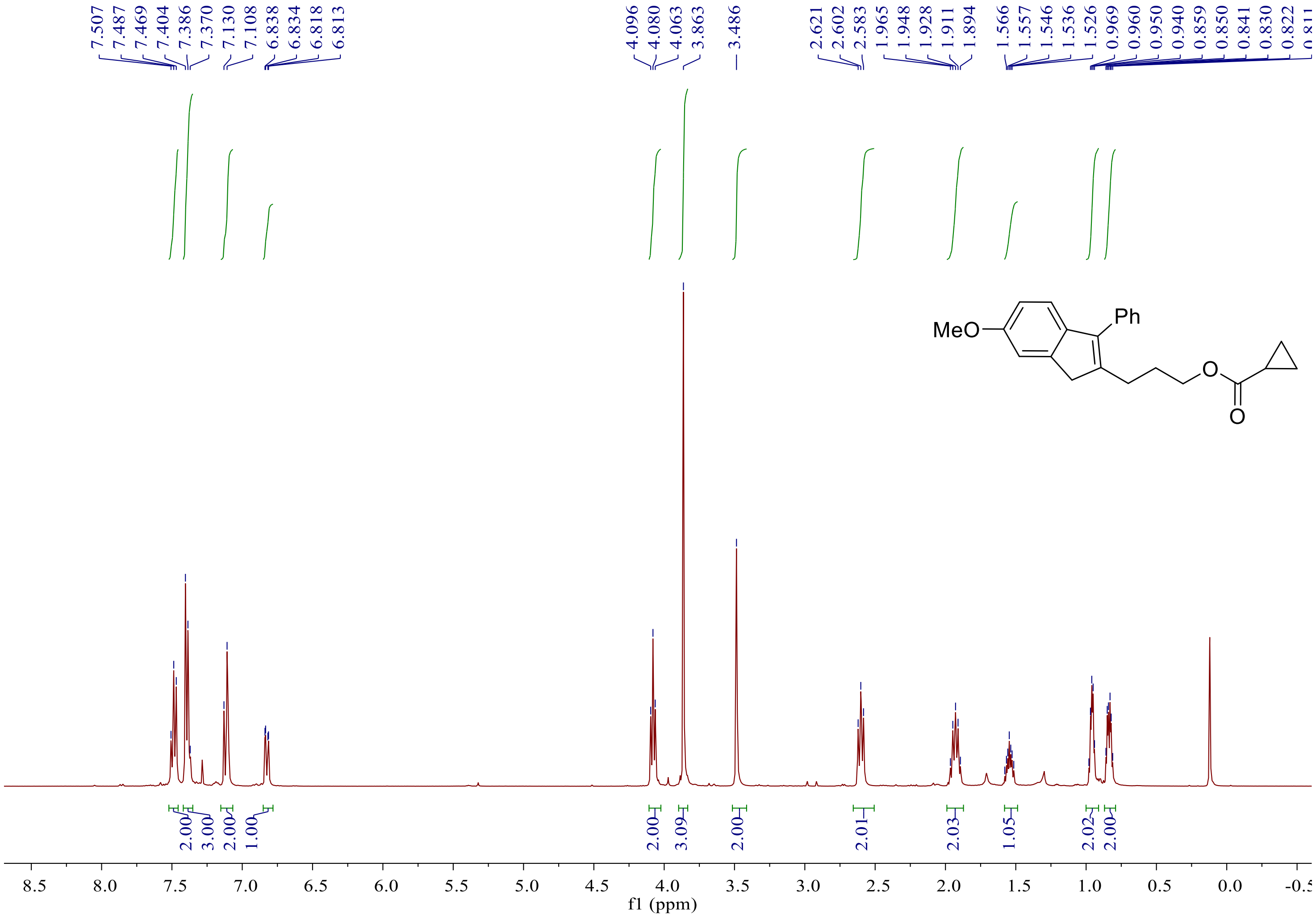

Supplementary Figure 58. ^{1}H NMR (400 MHz, $CDCl_3$) of **1ab**

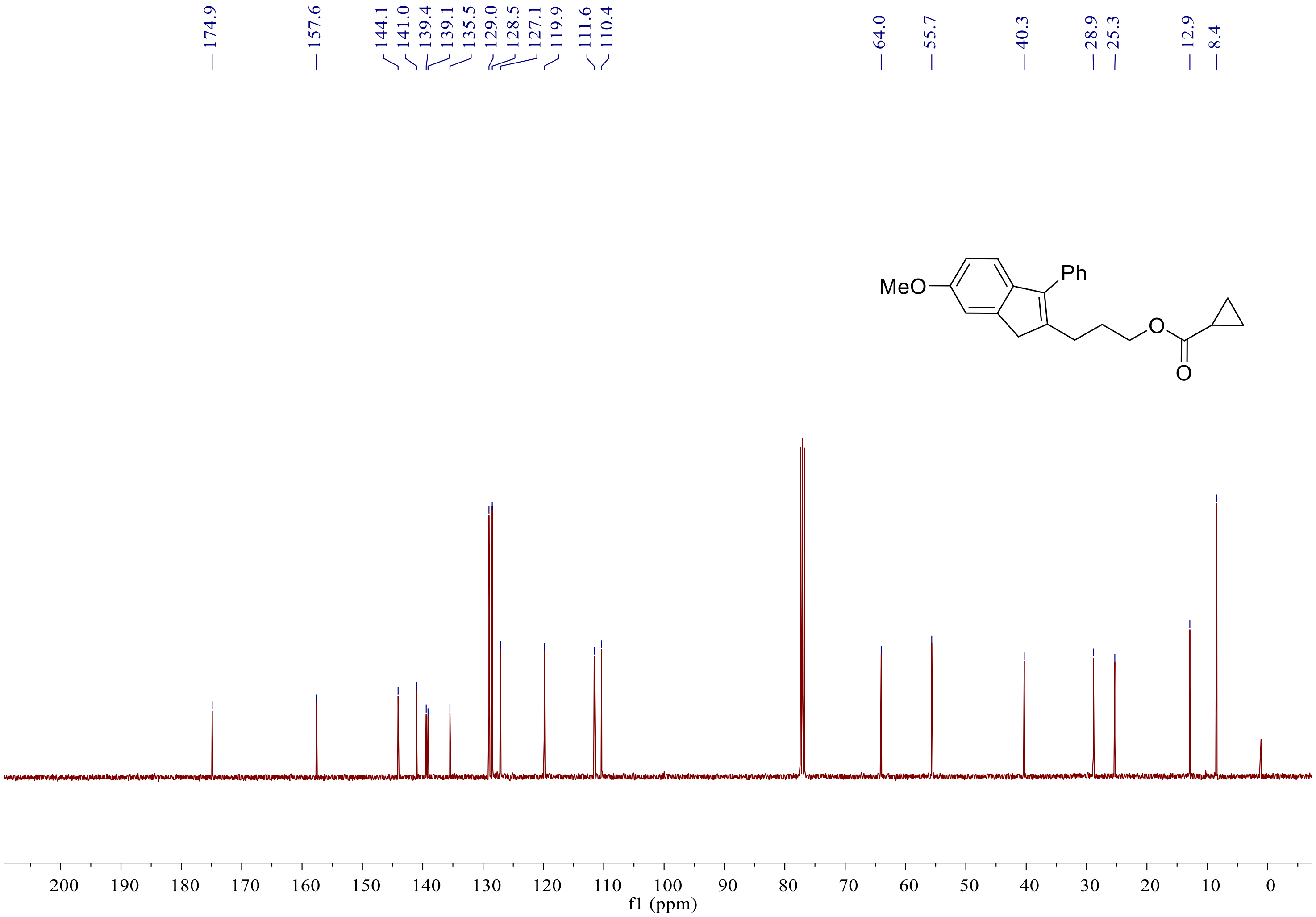

Supplementary Figure 59. ^{13}C NMR (100 MHz, $CDCl_3$) of **1ab**

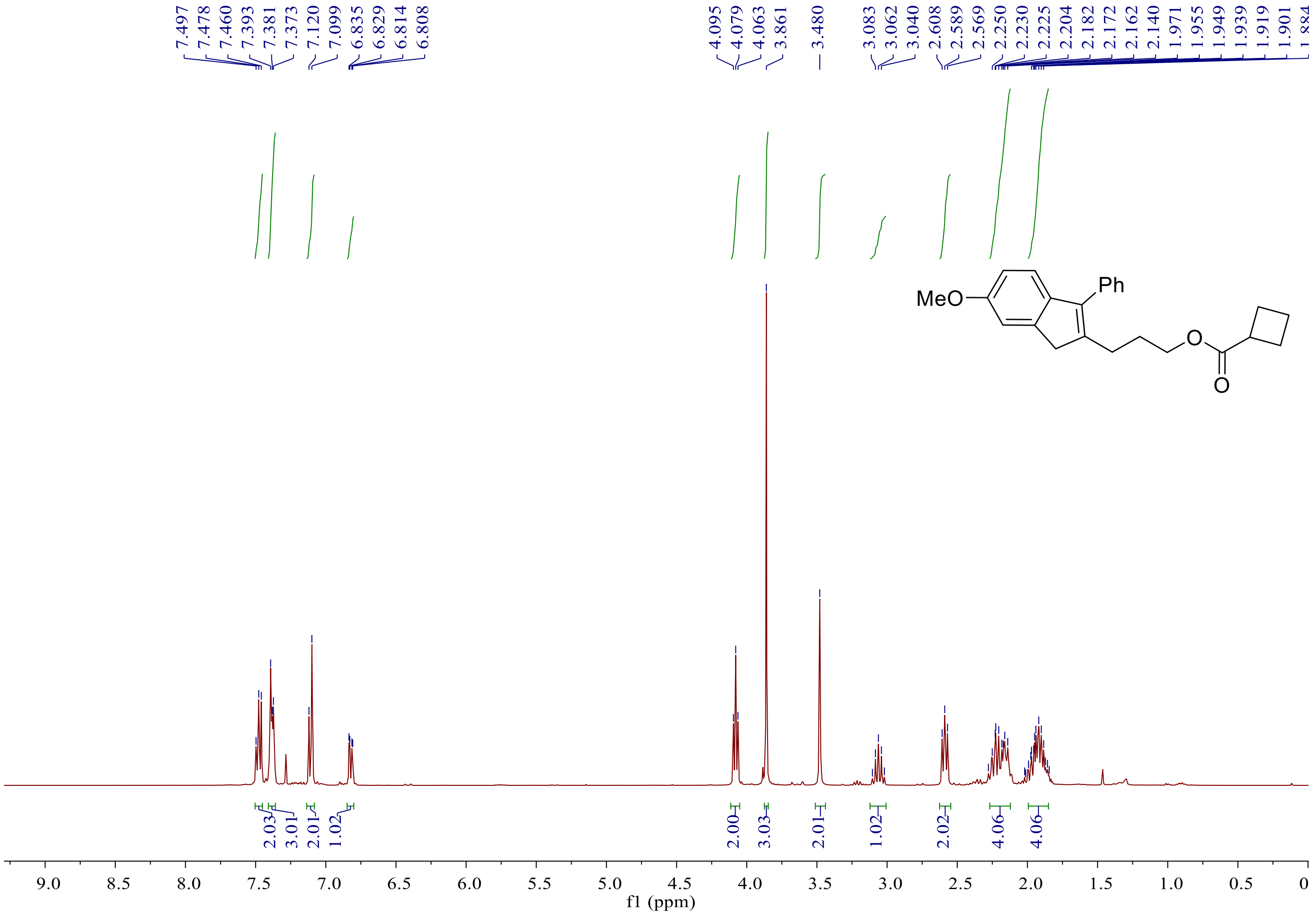

Supplementary Figure 60. ^{1}H NMR (400 MHz, $CDCl_3$) of **1ac**

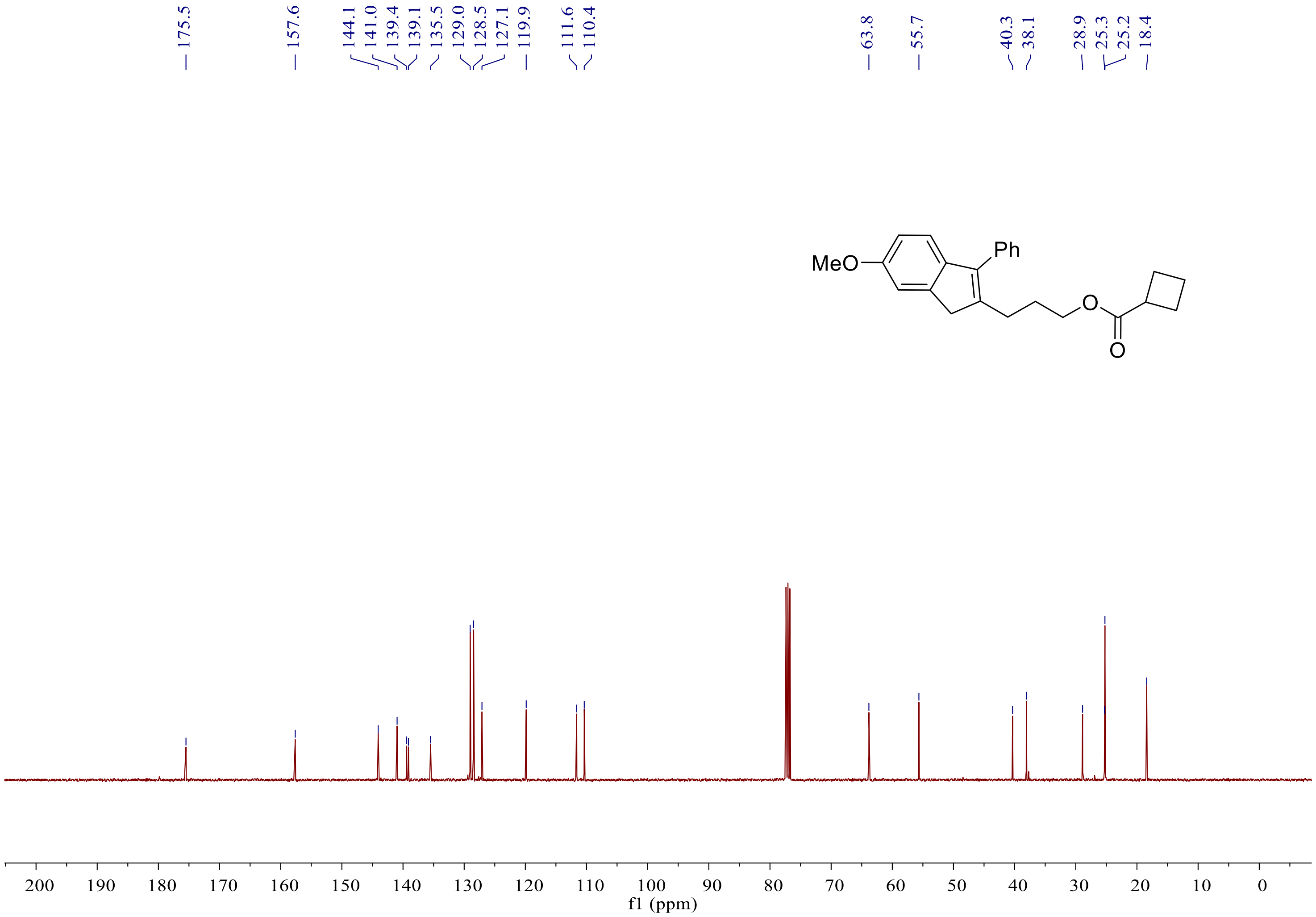

Supplementary Figure 61. ^{13}C NMR (100 MHz, $CDCl_3$) of **1ac**

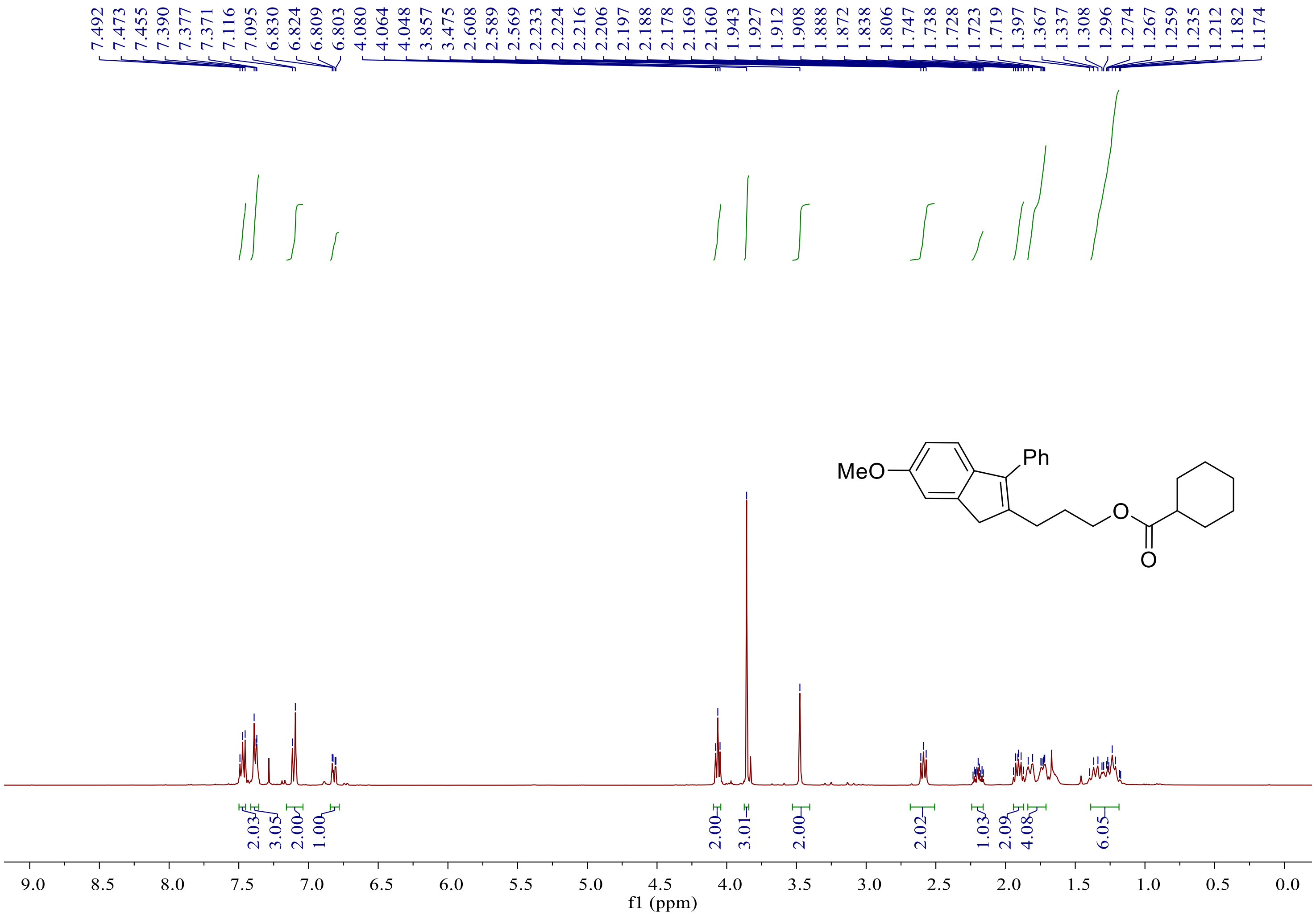

Supplementary Figure 62. ^{1}H NMR (400 MHz, $CDCl_3$) of **1ad**

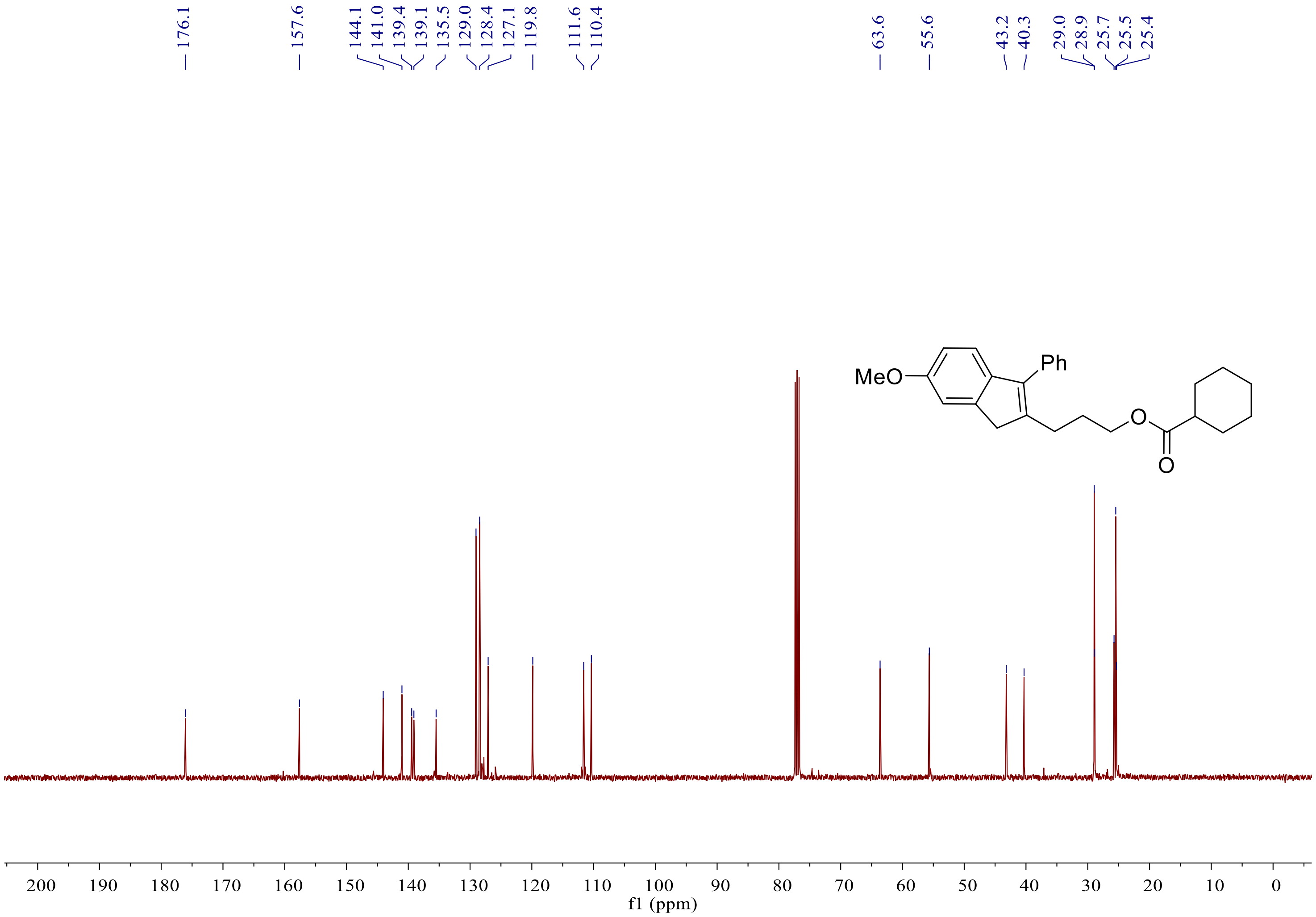

Supplementary Figure 63. ^{13}C NMR (100 MHz, $CDCl_3$) of **1ad**

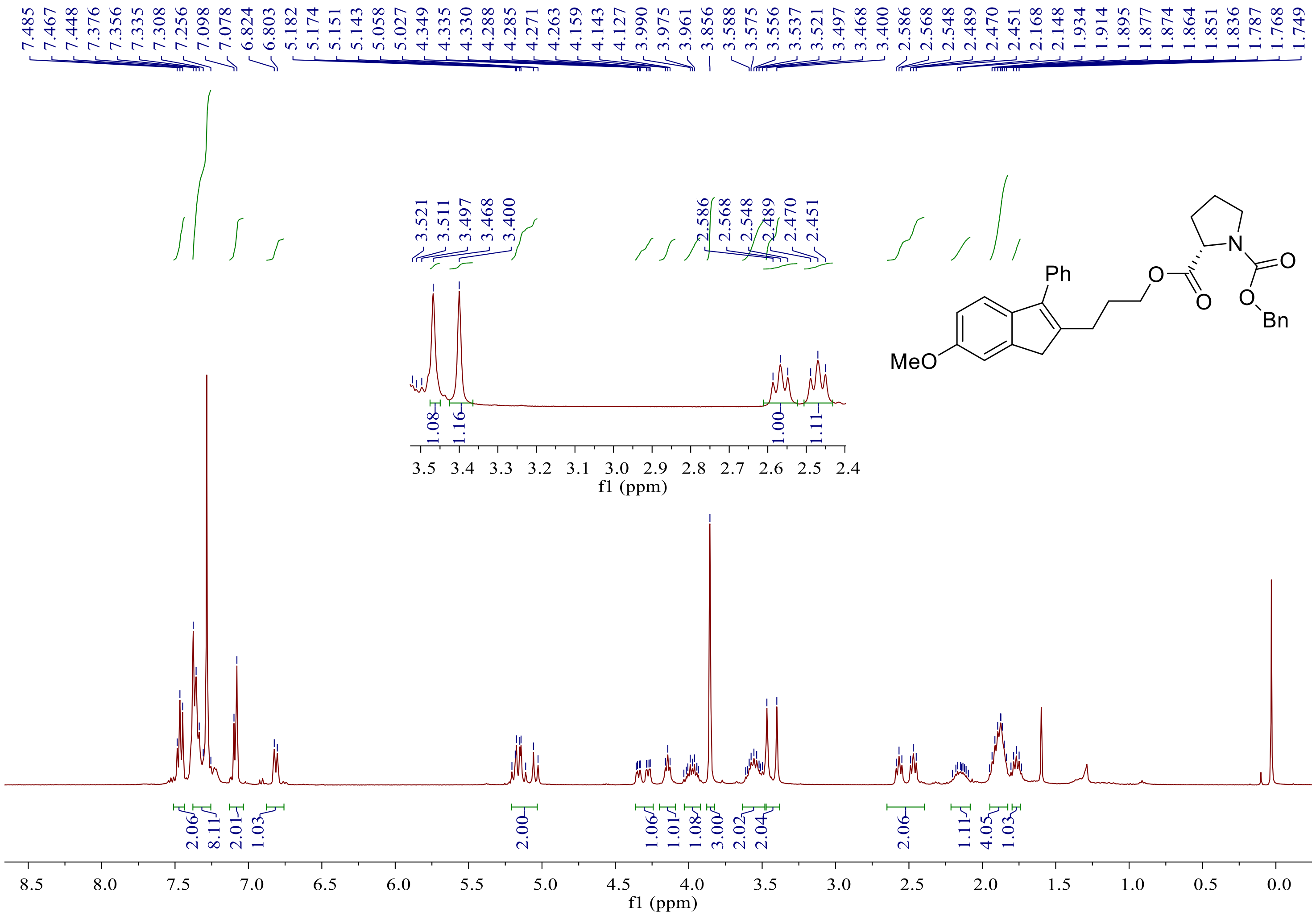

Supplementary Figure 64. ^{1}H NMR (400 MHz, $CDCl_3$) of **1ae**

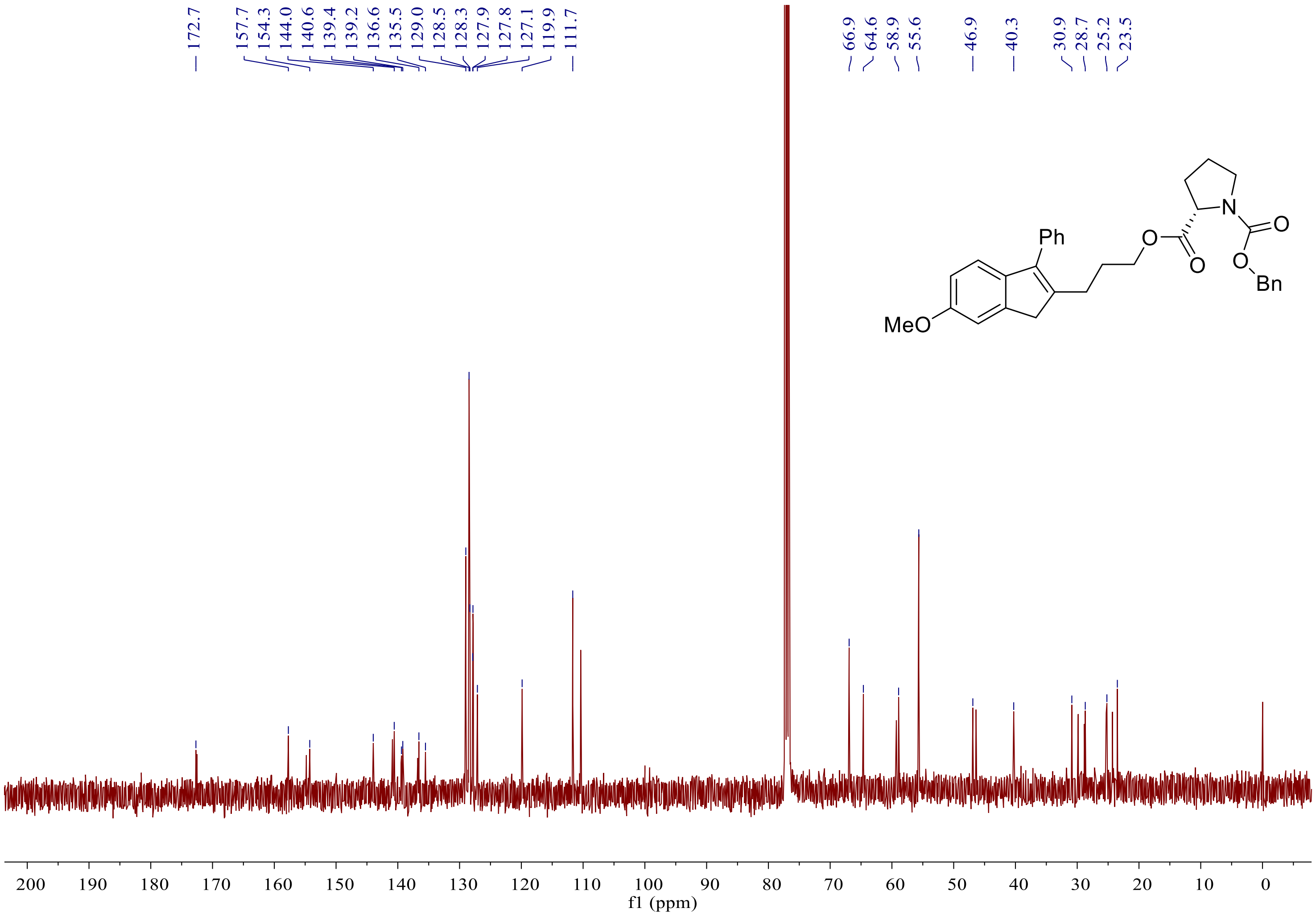

Supplementary Figure 65. ^{13}C NMR (100 MHz, $CDCl_3$) of **1ae**

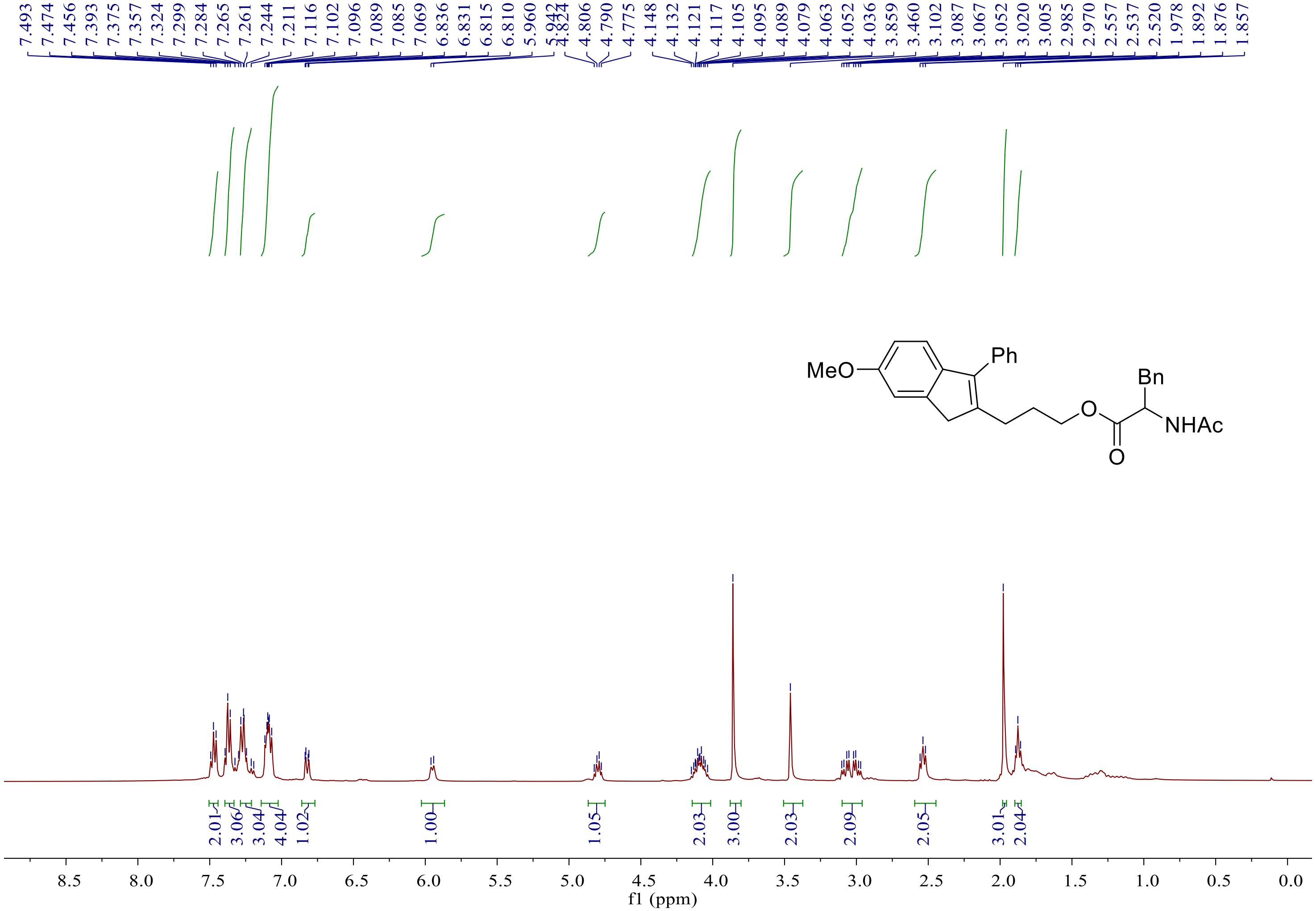

Supplementary Figure 66. ^{1}H NMR (400 MHz, $CDCl_3$) of **1af**

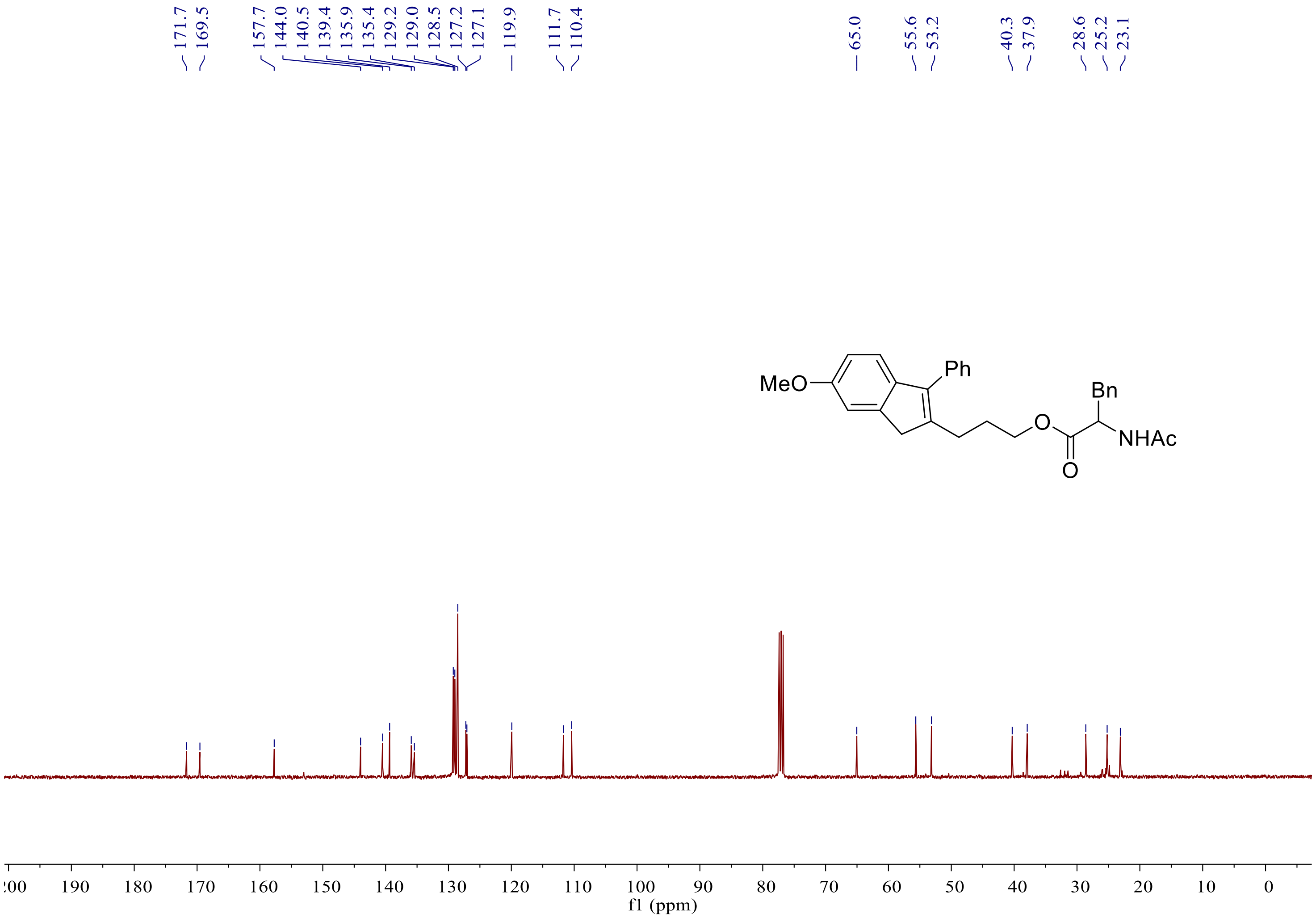

Supplementary Figure 67. ^{13}C NMR (100 MHz, $CDCl_3$) of **1af**

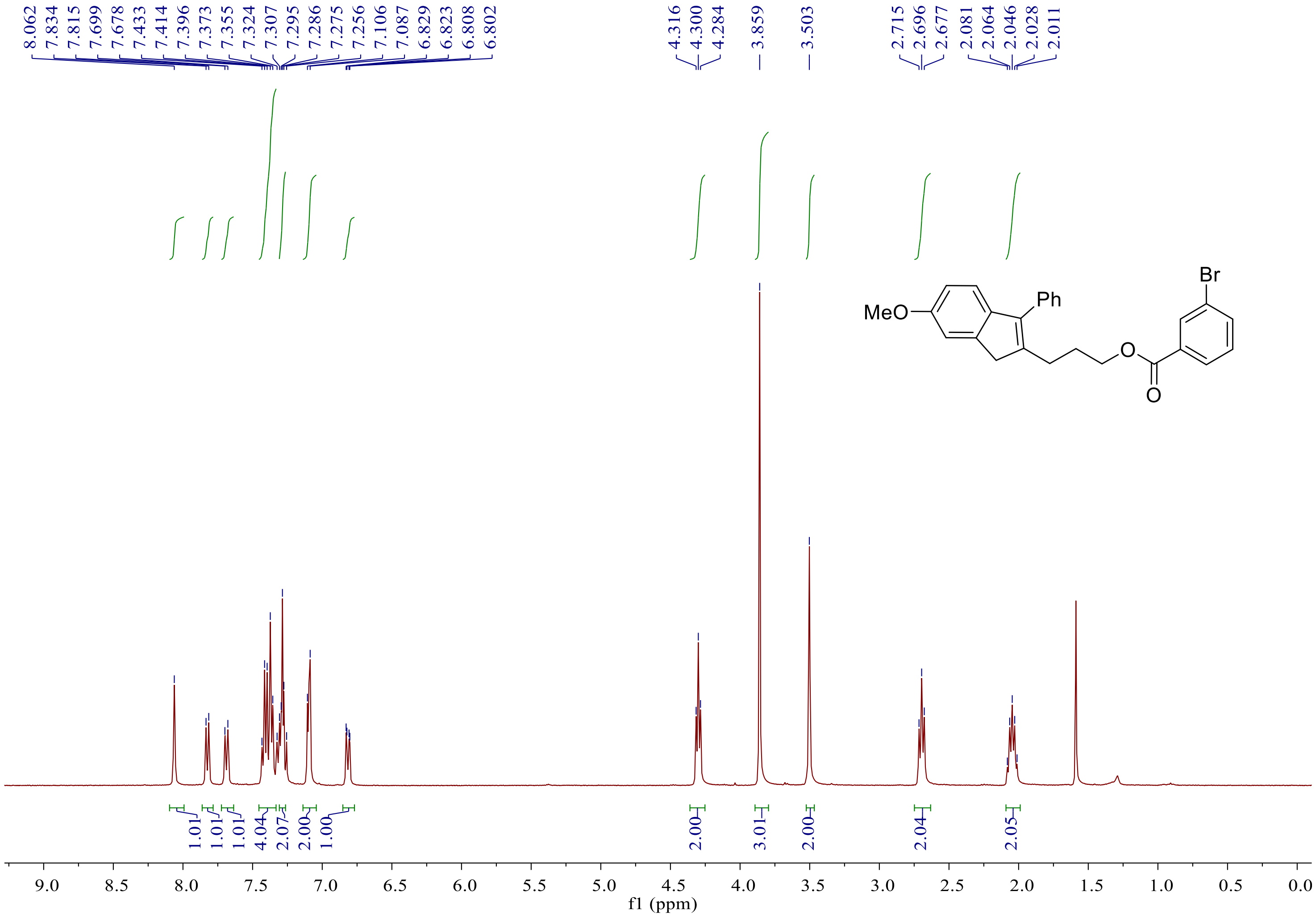

Supplementary Figure 68. ^{1}H NMR (400 MHz, $CDCl_3$) of **1ag**

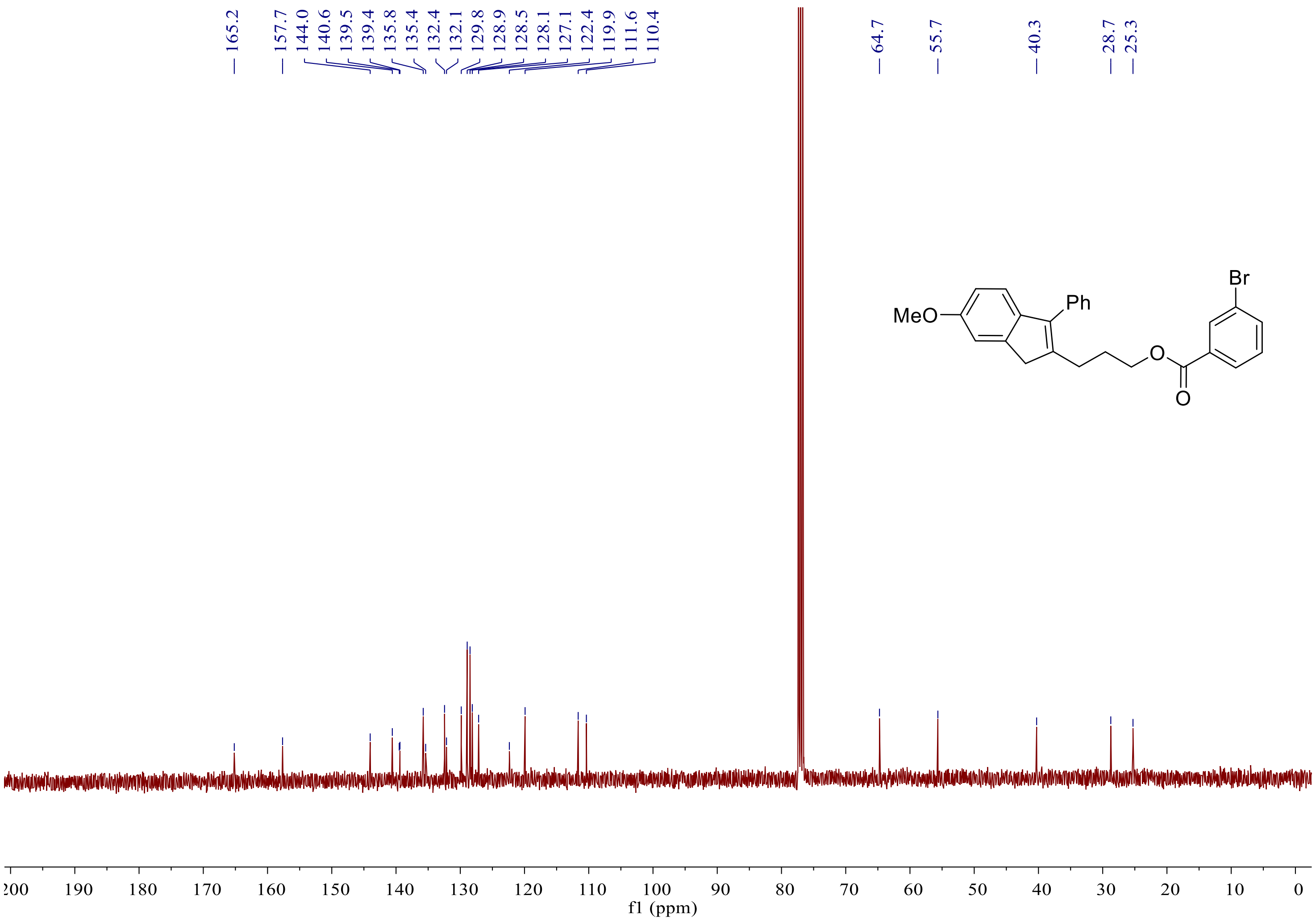

Supplementary Figure 69 ^{13}C NMR (100 MHz, $CDCl_3$) of **1ag**

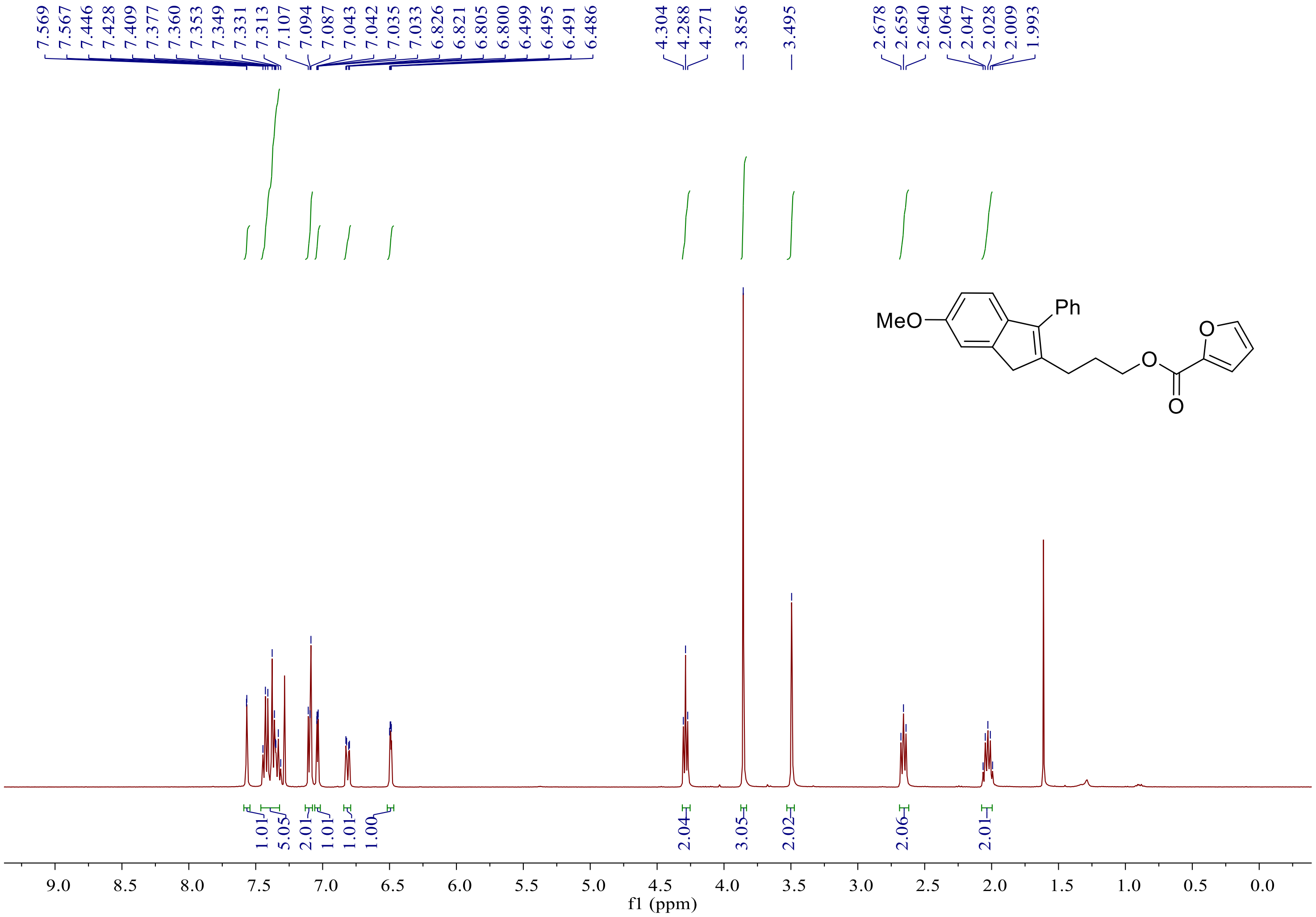

Supplementary Figure 70. ^{1}H NMR (400 MHz, $CDCl_3$) of **1ah**

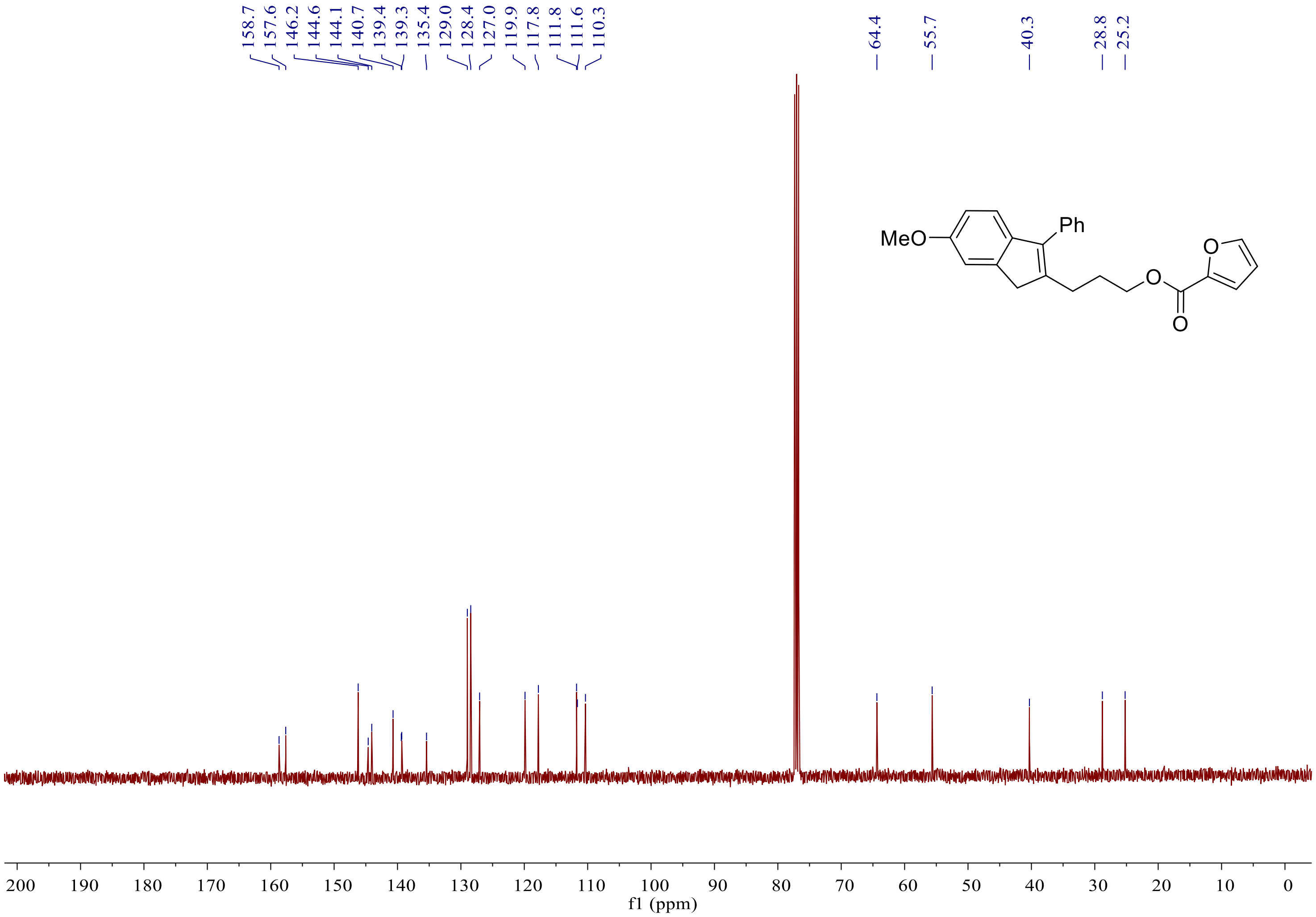

Supplementary Figure 71. ^{13}C NMR (100 MHz, $CDCl_3$) of **1ah**

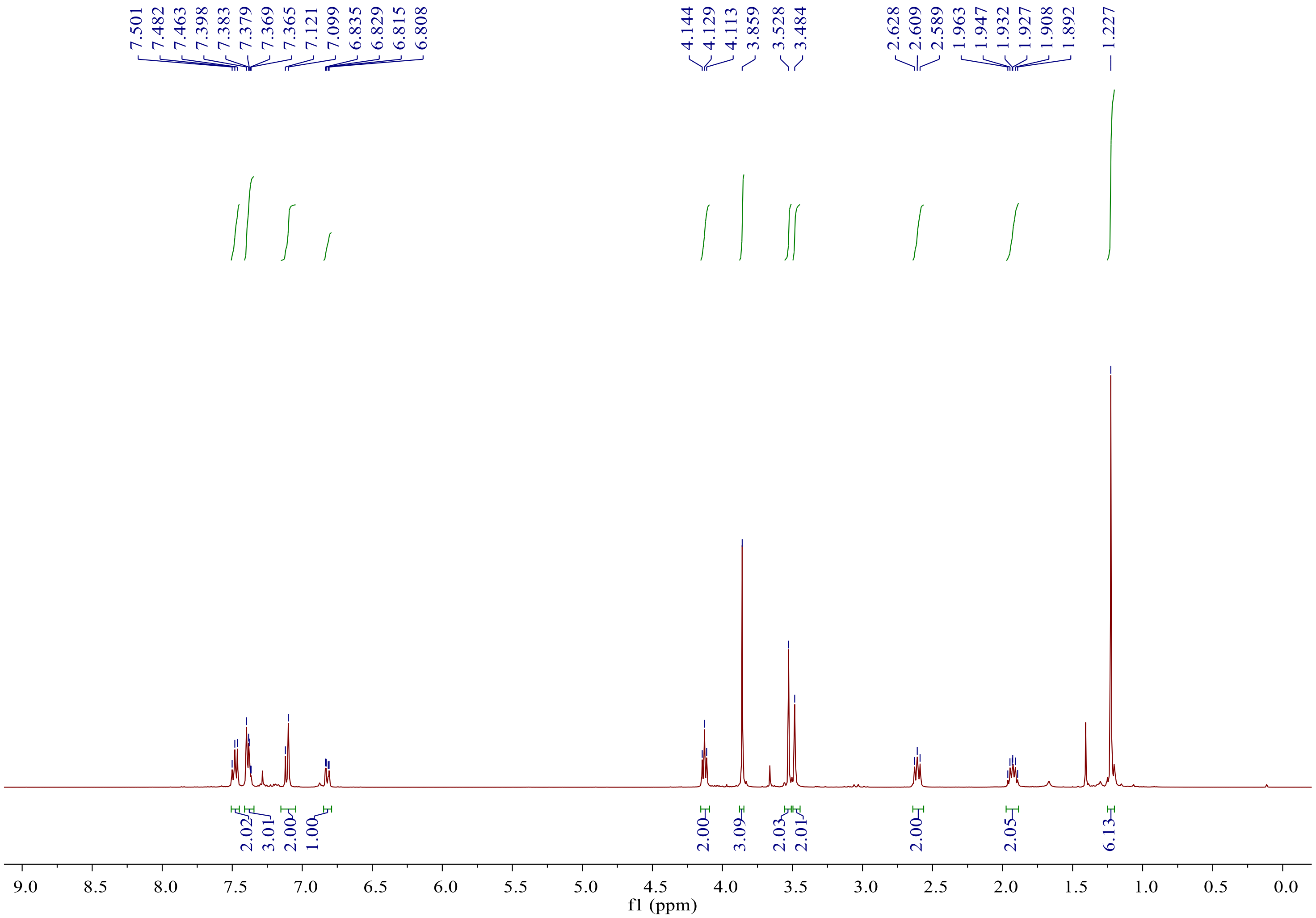

Supplementary Figure 72. ^{1}H NMR (400 MHz, $CDCl_3$) of **1ai**

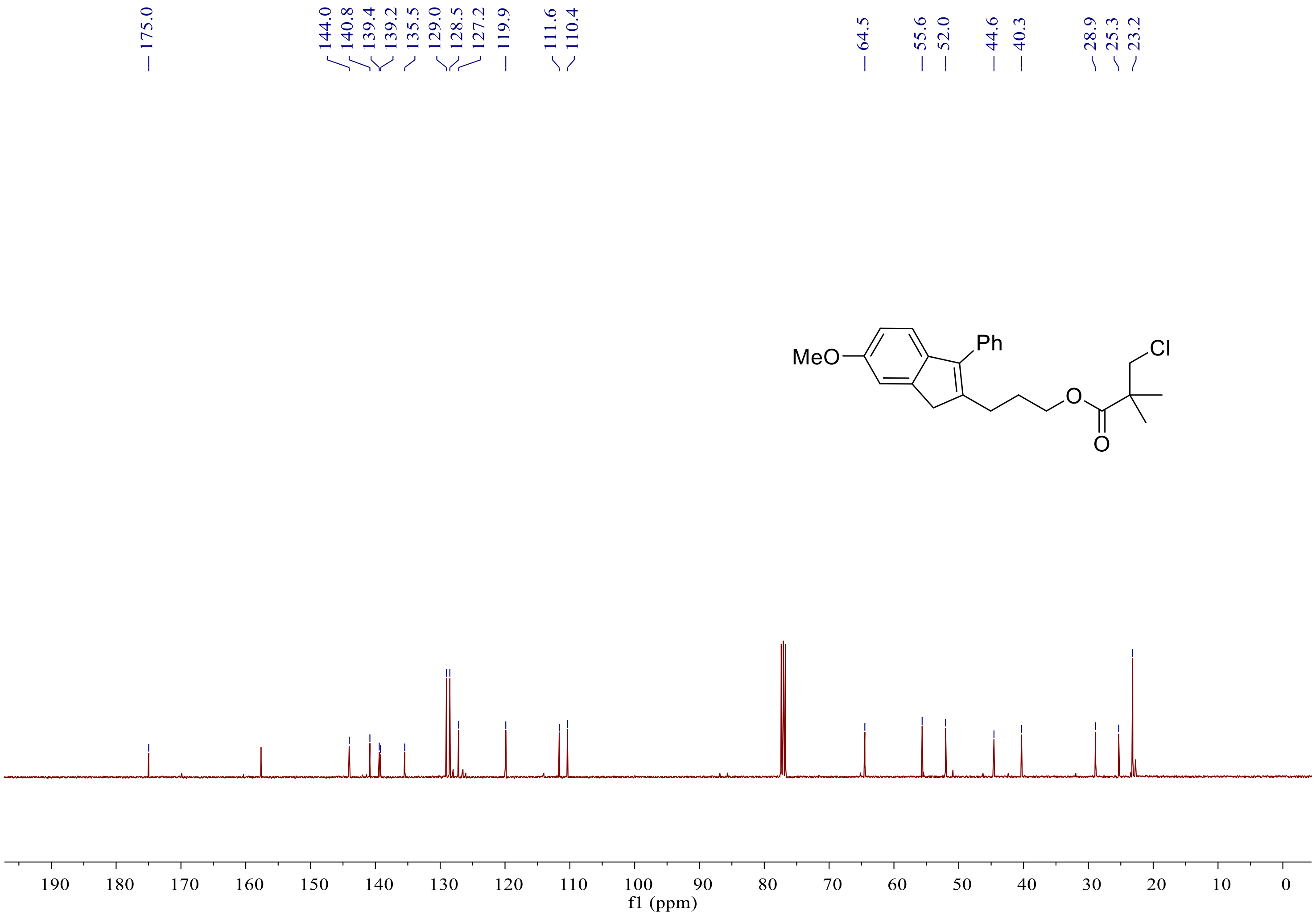

Supplementary Figure 73. ^{13}C NMR (100 MHz, $CDCl_3$) of **1ai**

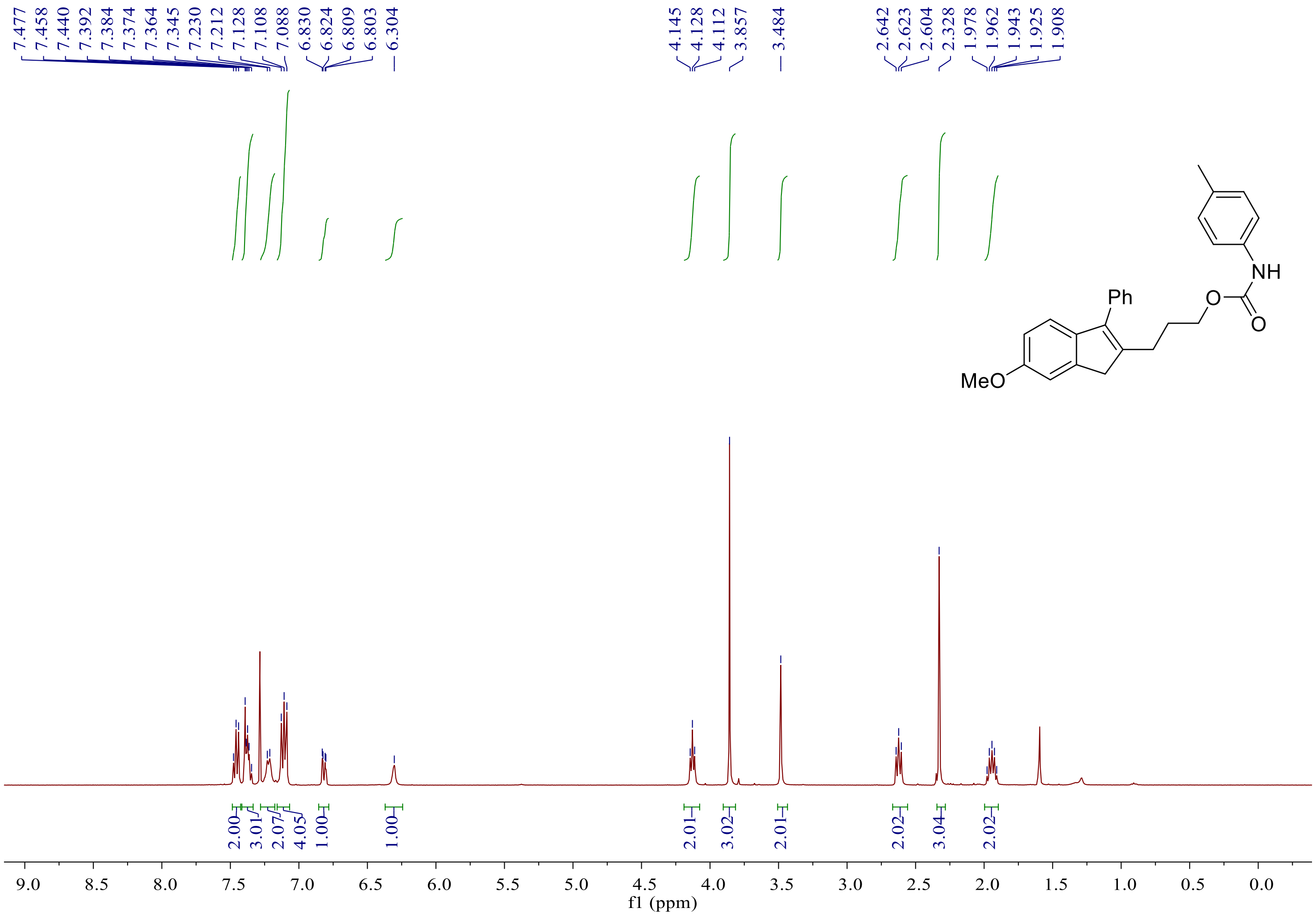

Supplementary Figure 74. ^{1}H NMR (400 MHz, $CDCl_3$) of **1aj**

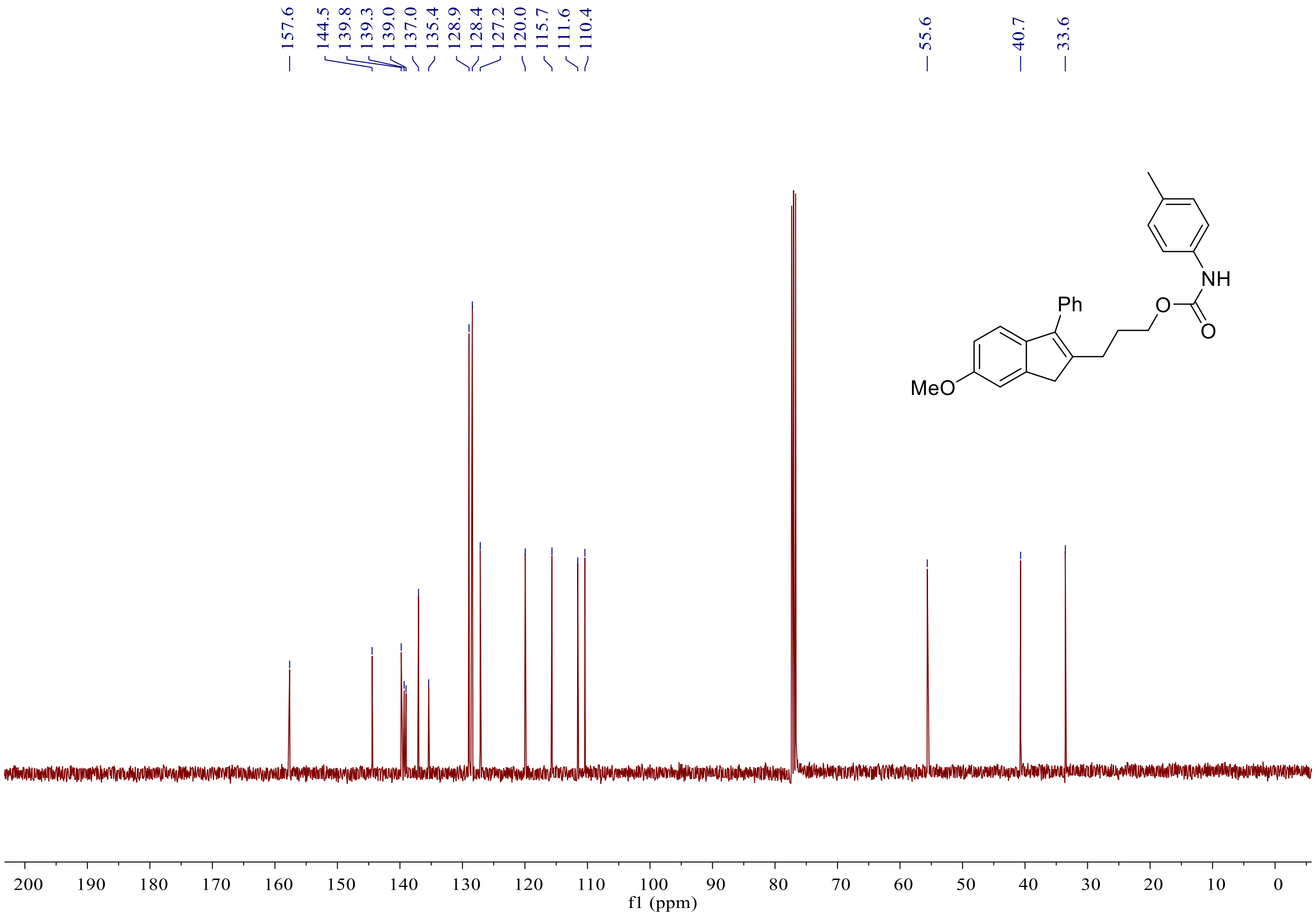

Supplementary Figure 75. ^{13}C NMR (100 MHz, $CDCl_3$) of **1aj**

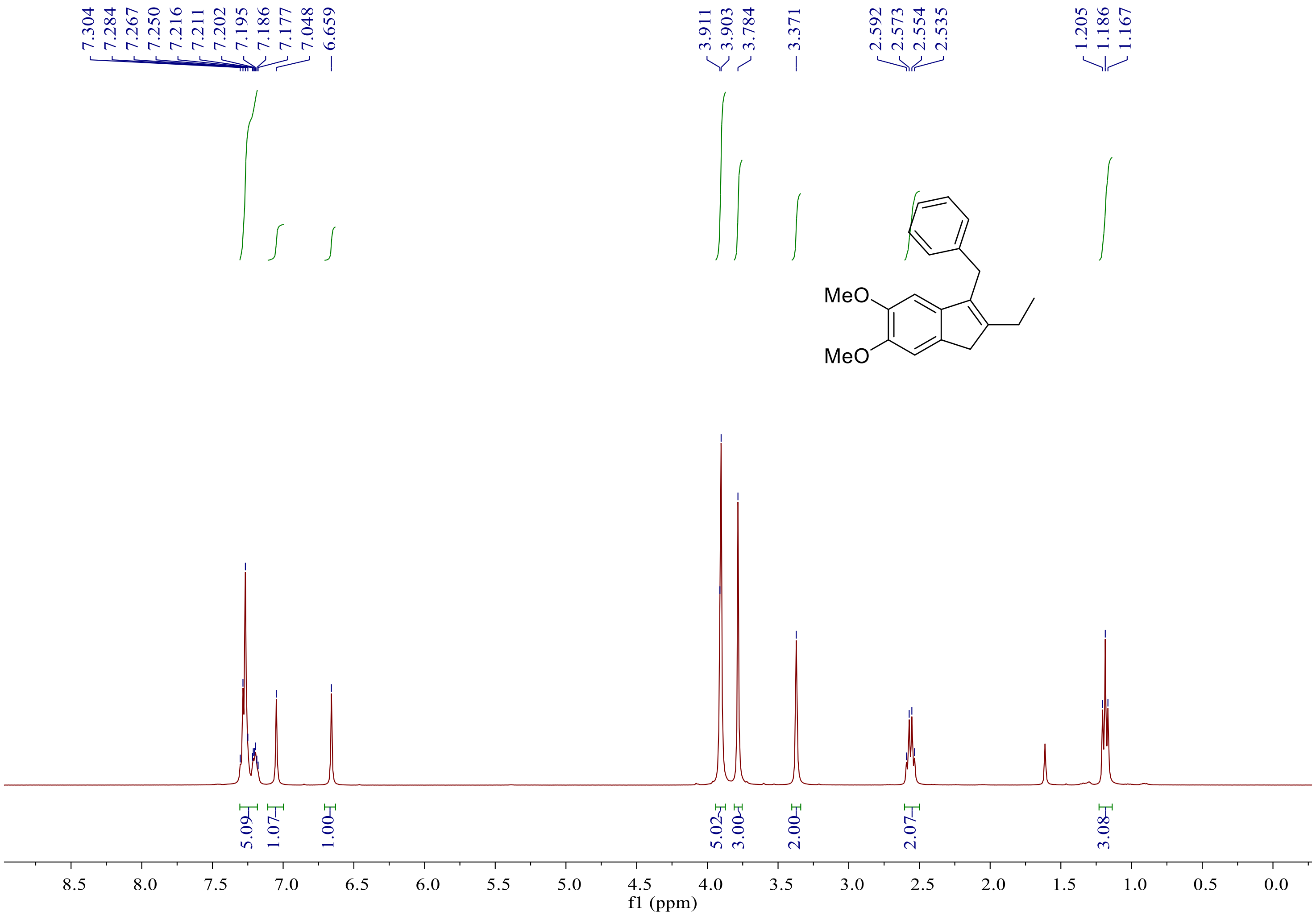

Supplementary Figure 76. ^{1}H NMR (400 MHz, $CDCl_3$) of **1ar**

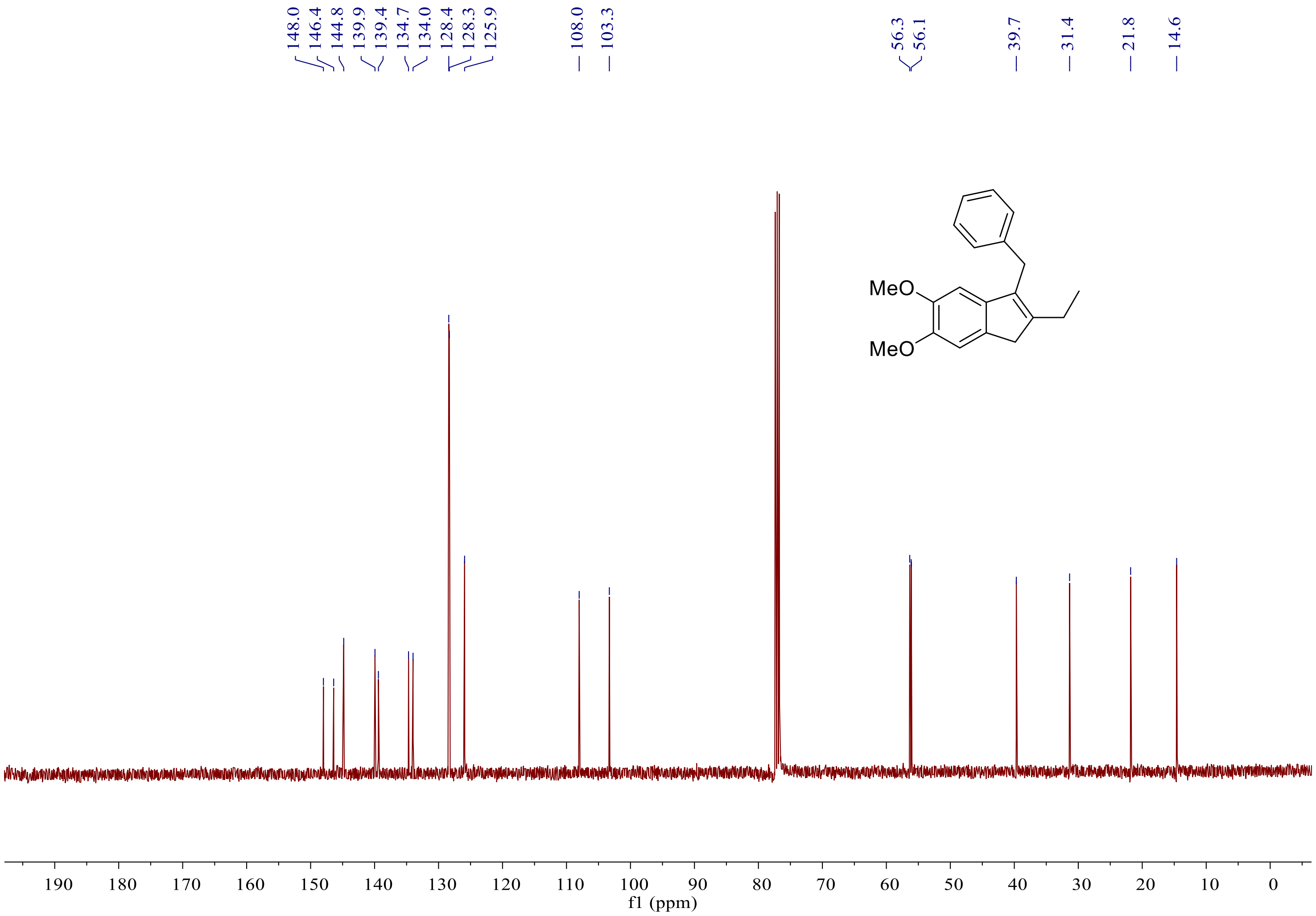

Supplementary Figure 77. ^{13}C NMR (100 MHz, $CDCl_3$) of **1ar**

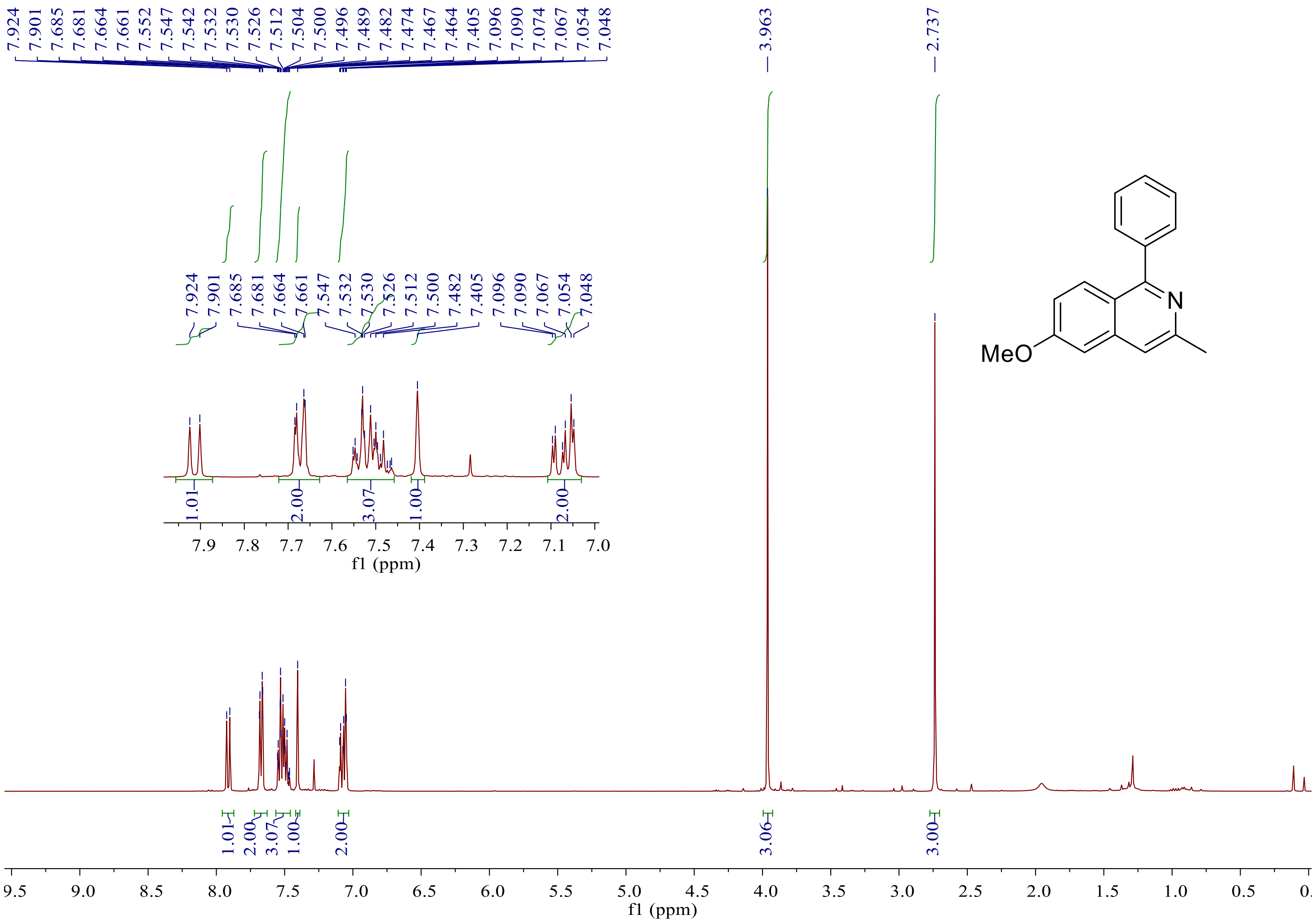

Supplementary Figure 78. ^{1}H NMR (400 MHz, $CDCl_3$) of **2a**

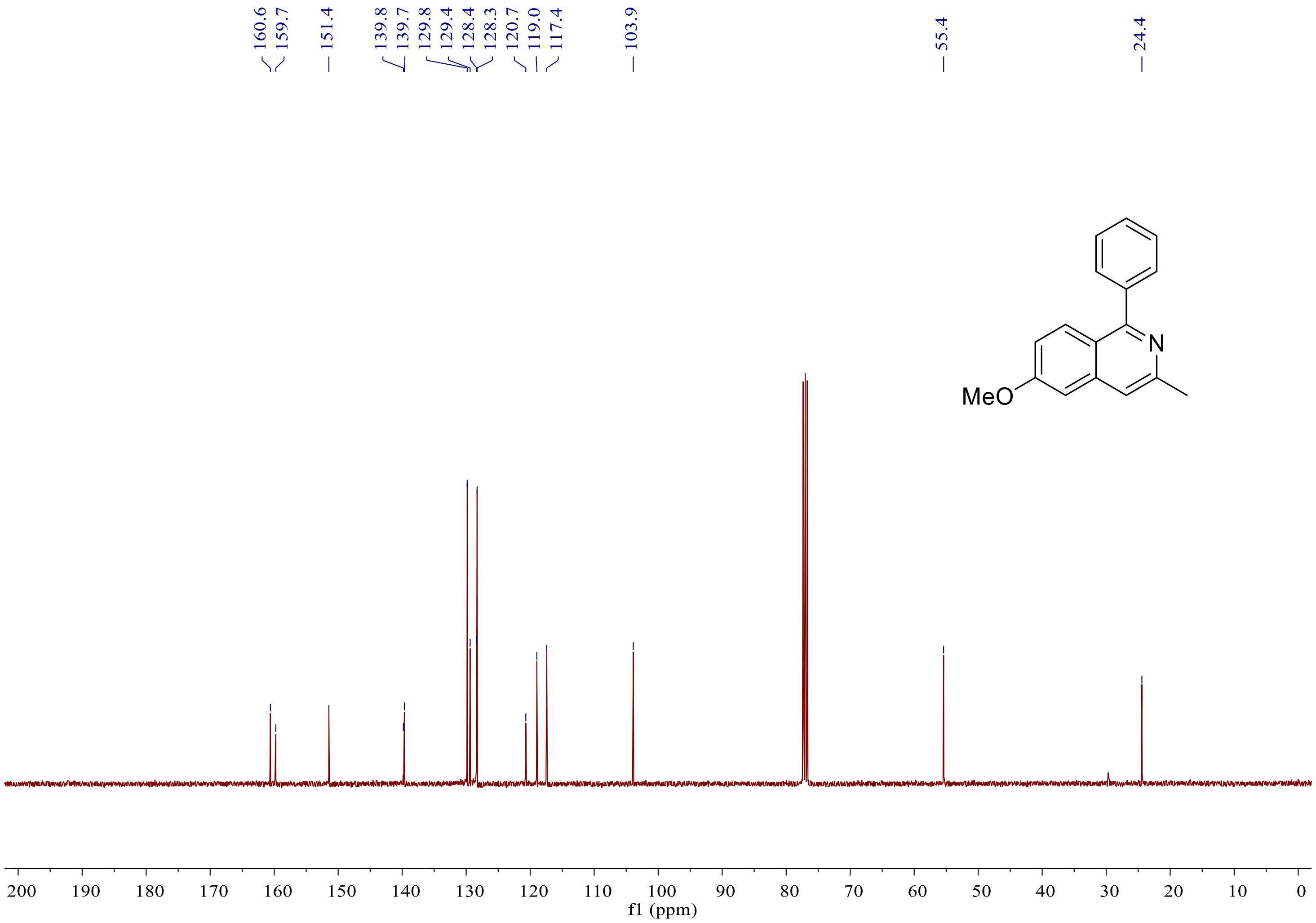

Supplementary Figure 79. ^{13}C NMR (100 MHz, $CDCl_3$) of **2a**

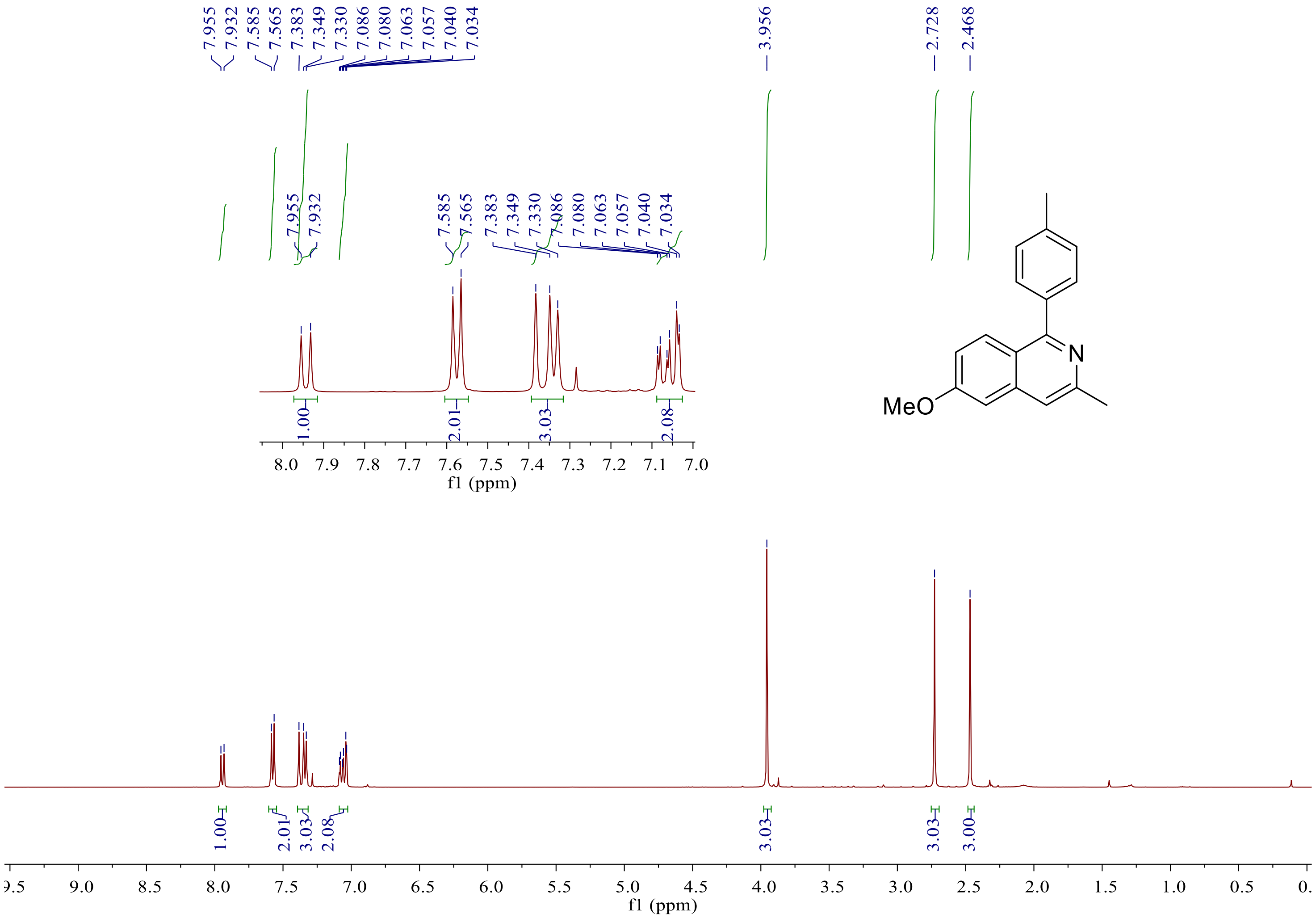

Supplementary Figure 80. ^{1}H NMR (400 MHz, $CDCl_3$) of **2b**

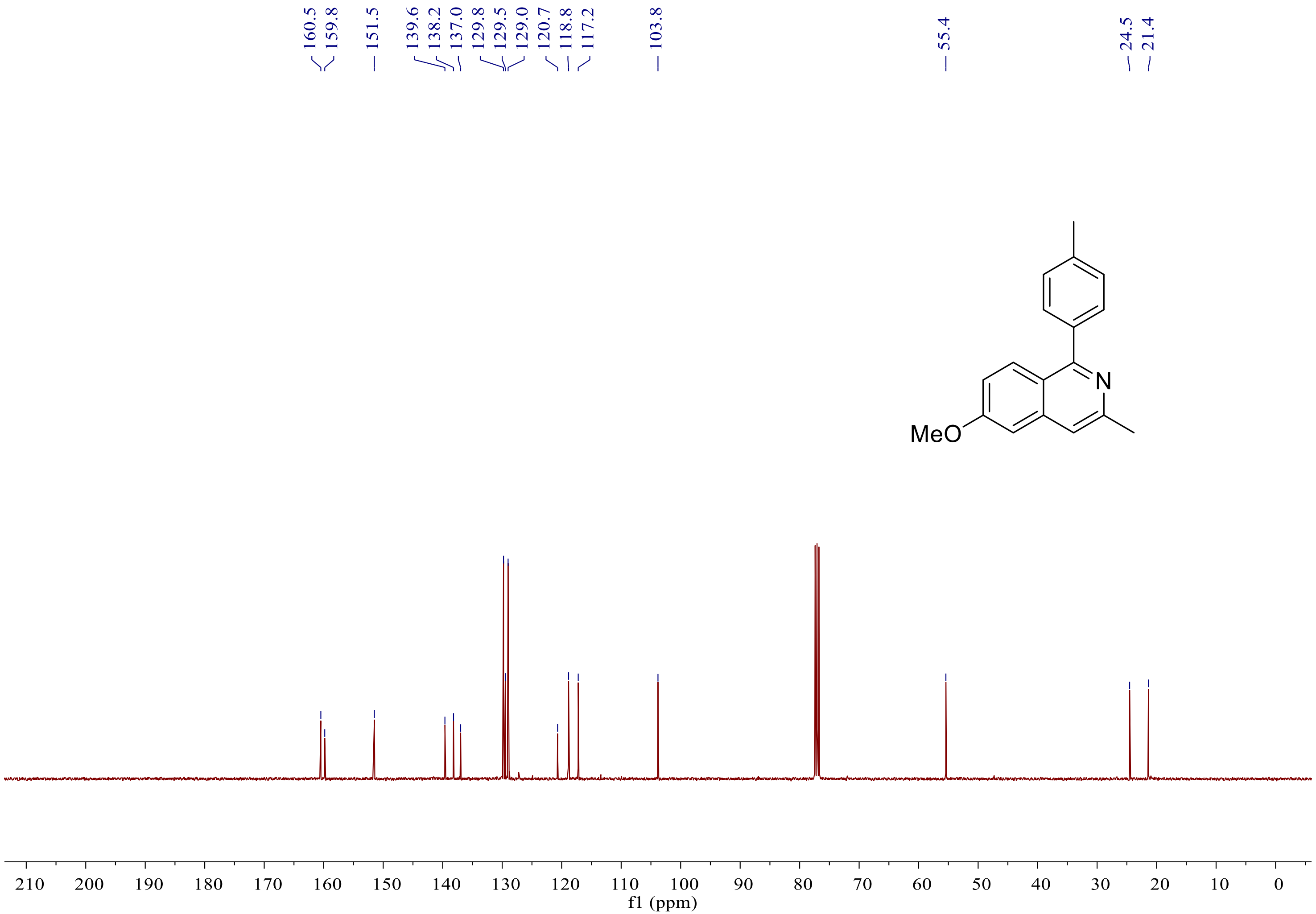

Supplementary Figure 81 ^{13}C NMR (100 MHz, $CDCl_3$) of **2b**

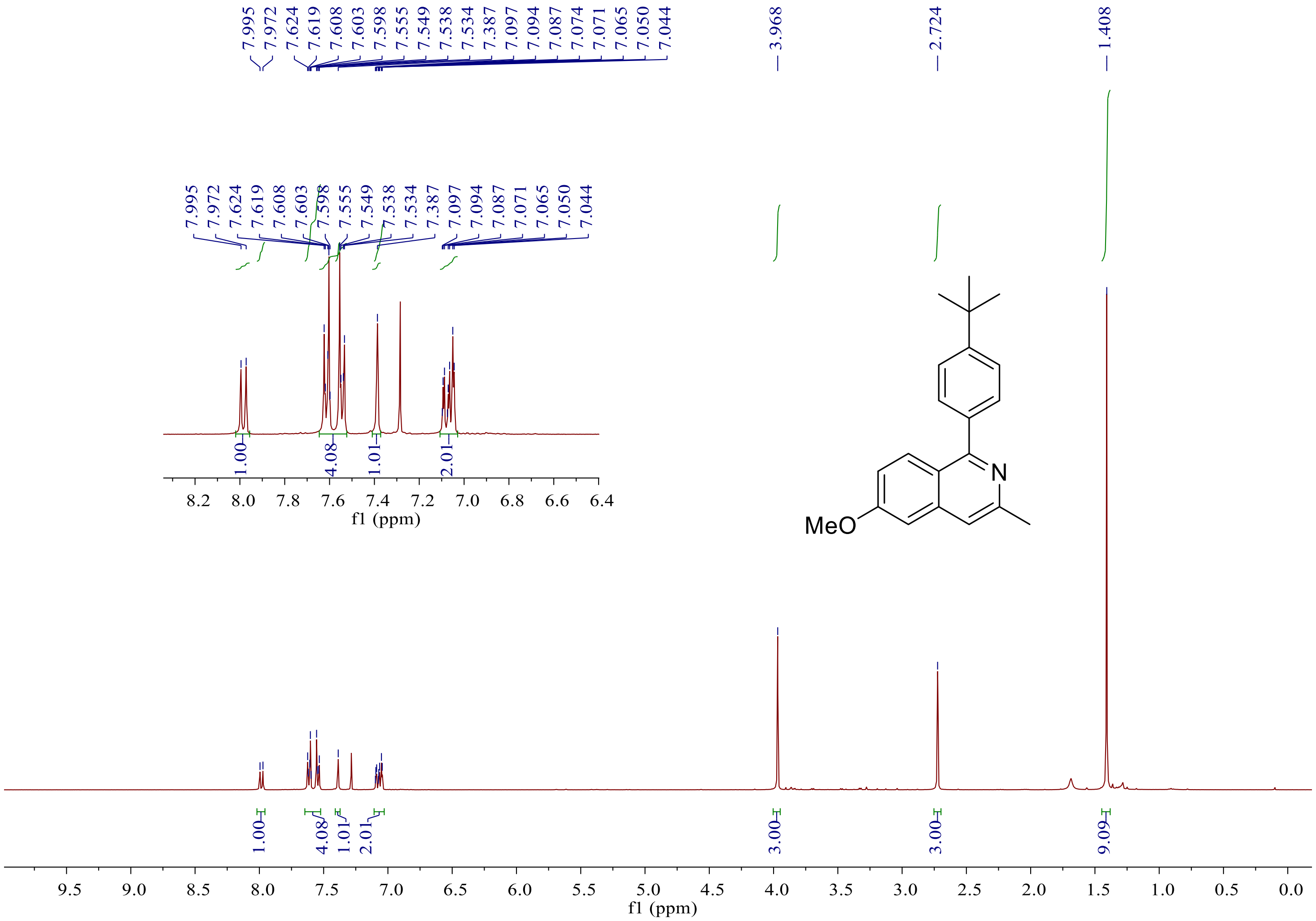

Supplementary Figure 82. ^{1}H NMR (400 MHz, $CDCl_3$) of **2c**

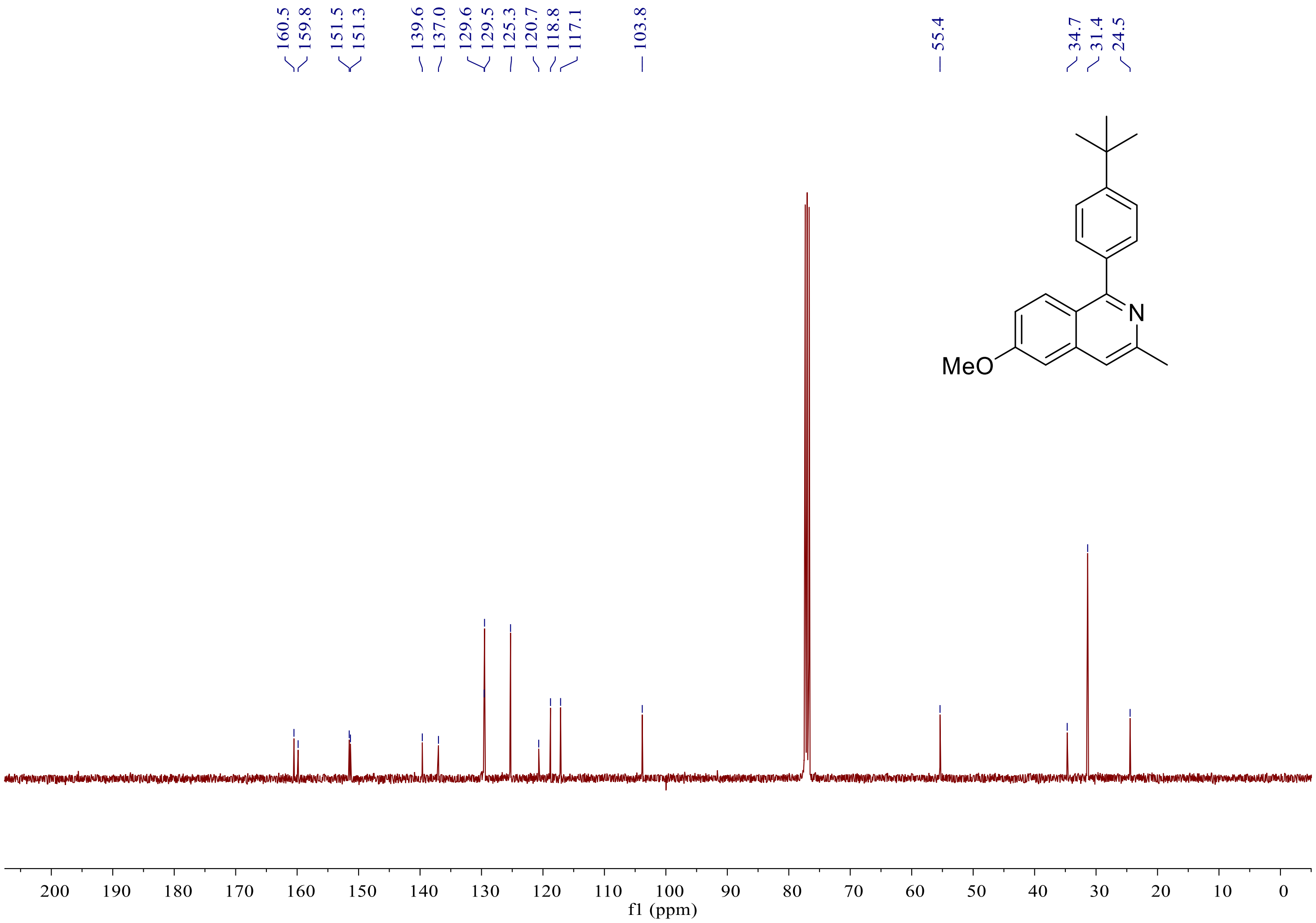

Supplementary Figure 83. ^{13}C NMR (100 MHz, $CDCl_3$) of **2c**

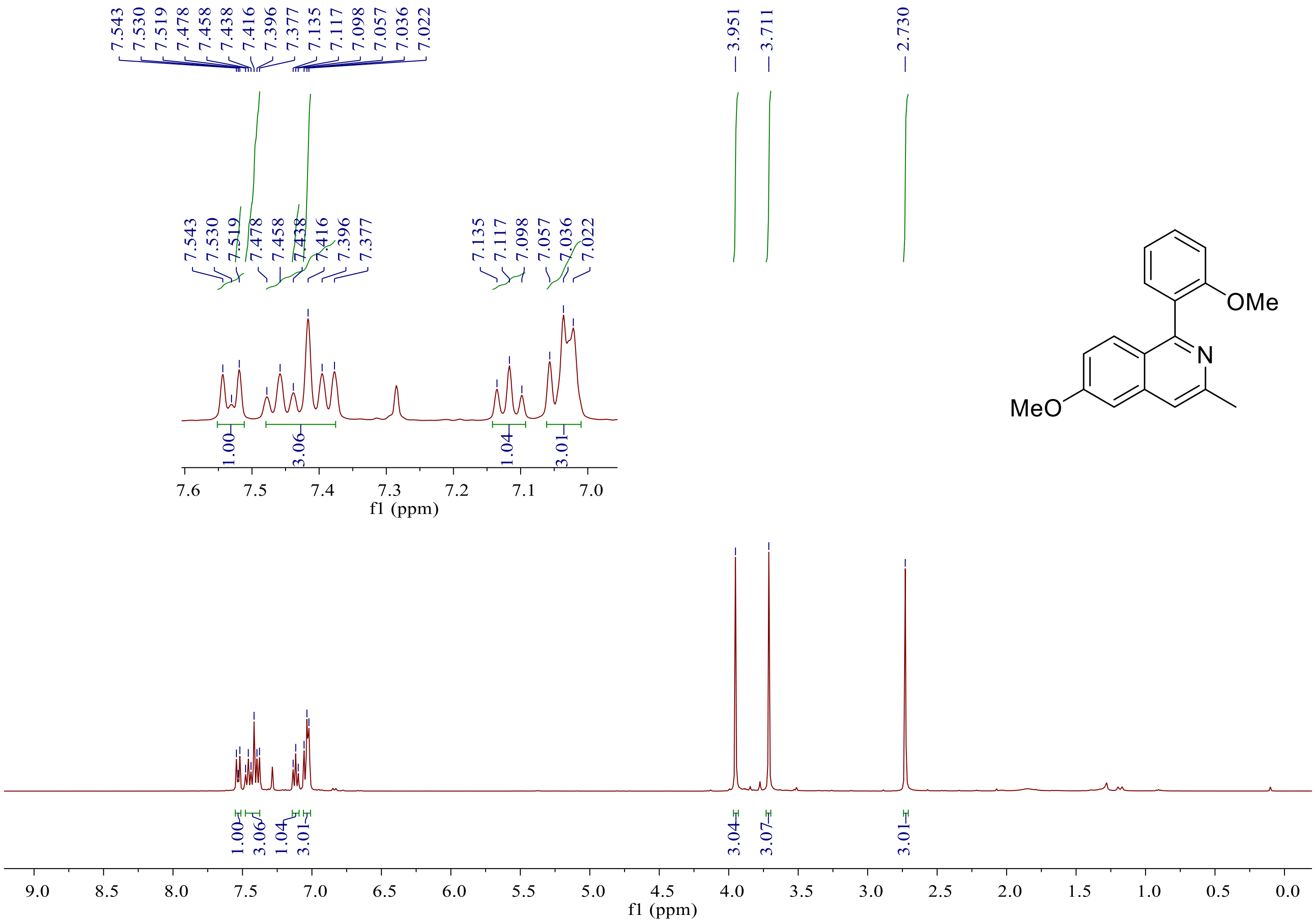

Supplementary Figure 84. ^{1}H NMR (400 MHz, $CDCl_3$) of **2d**

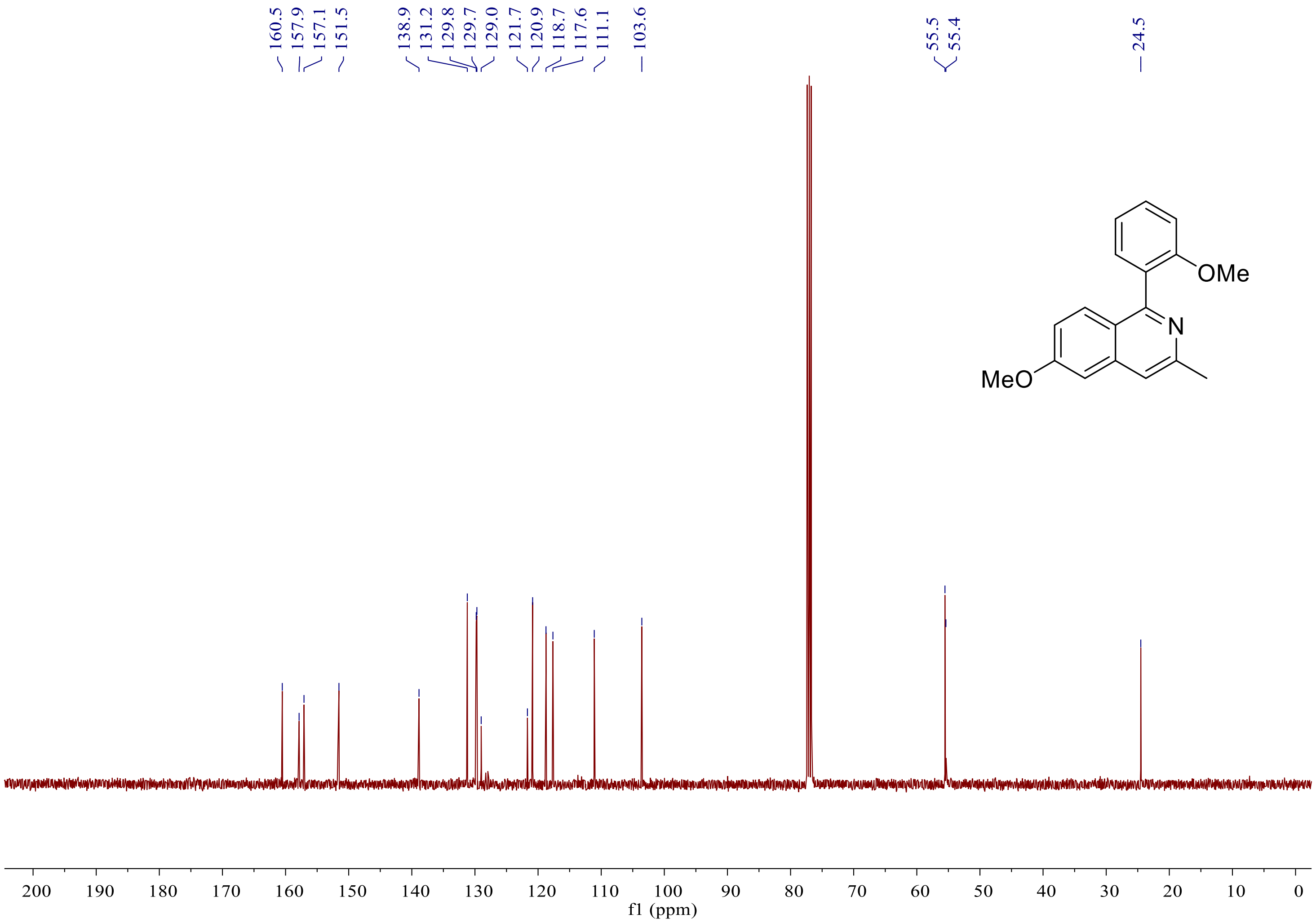

Supplementary Figure 85. ^{13}C NMR (100 MHz, $CDCl_3$) of **2d**

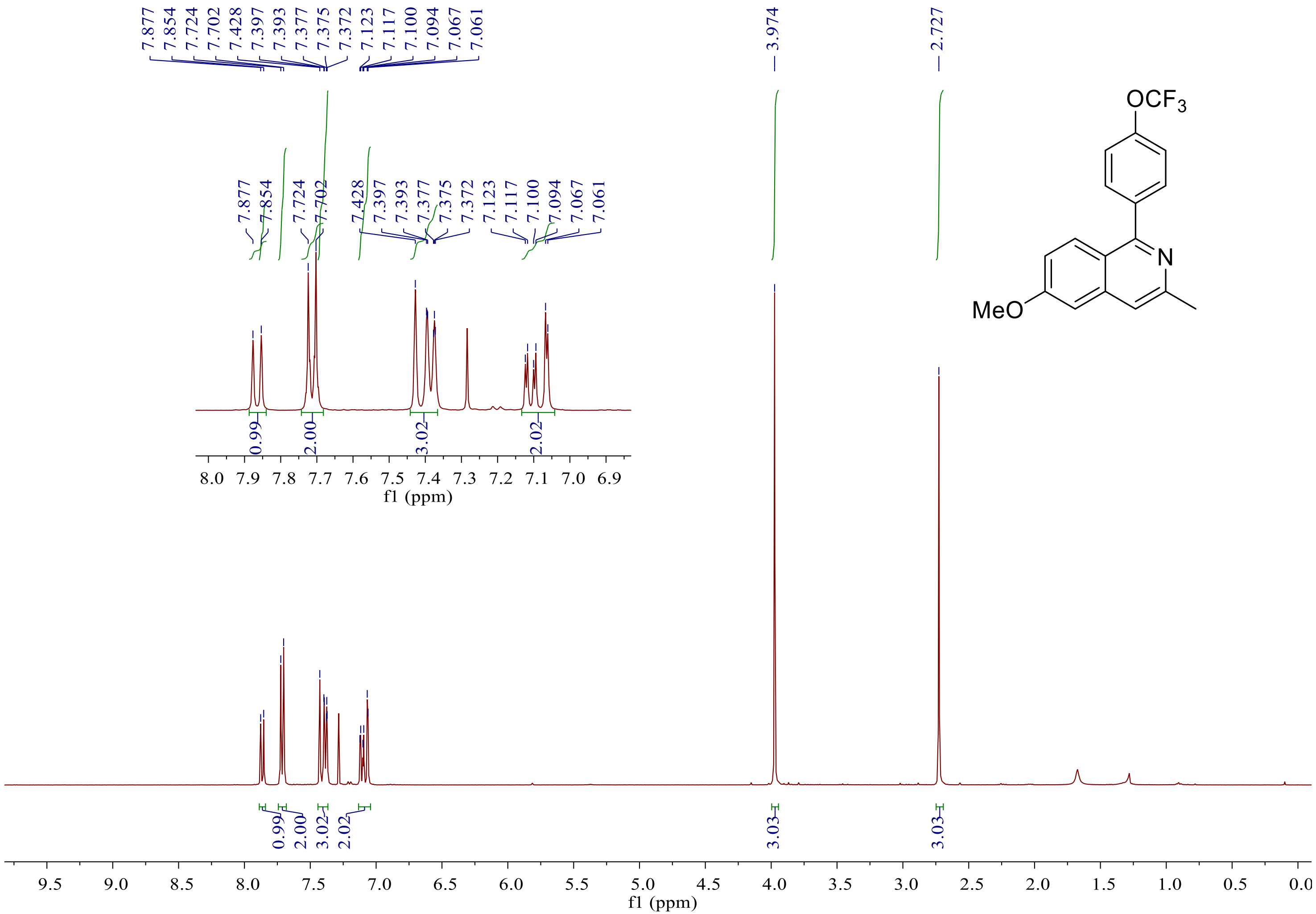

Supplementary Figure 86. ^{1}H NMR (400 MHz, $CDCl_3$) of **2e**

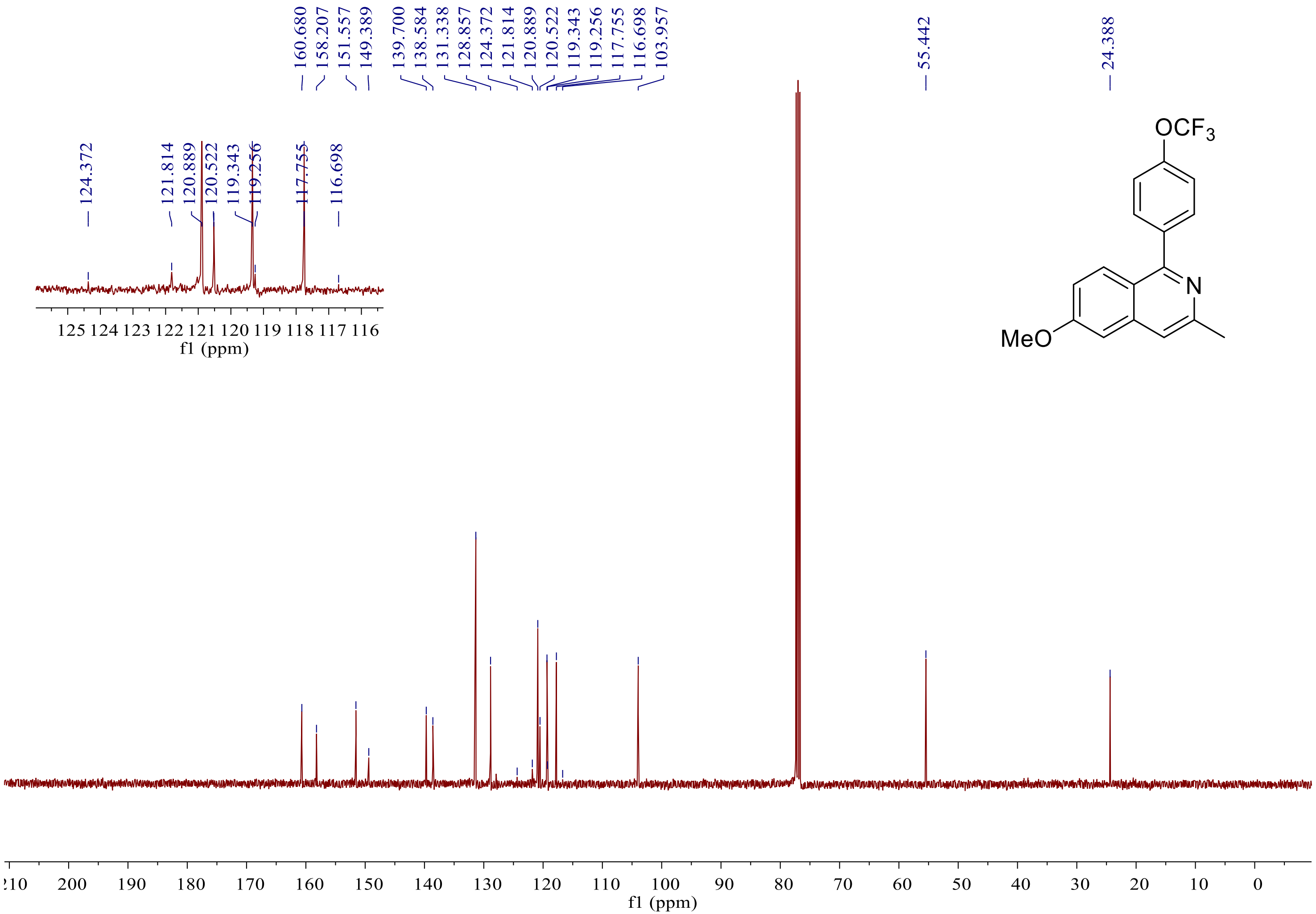

Supplementary Figure 87. ^{13}C NMR (100 MHz, $CDCl_3$) of **2e**

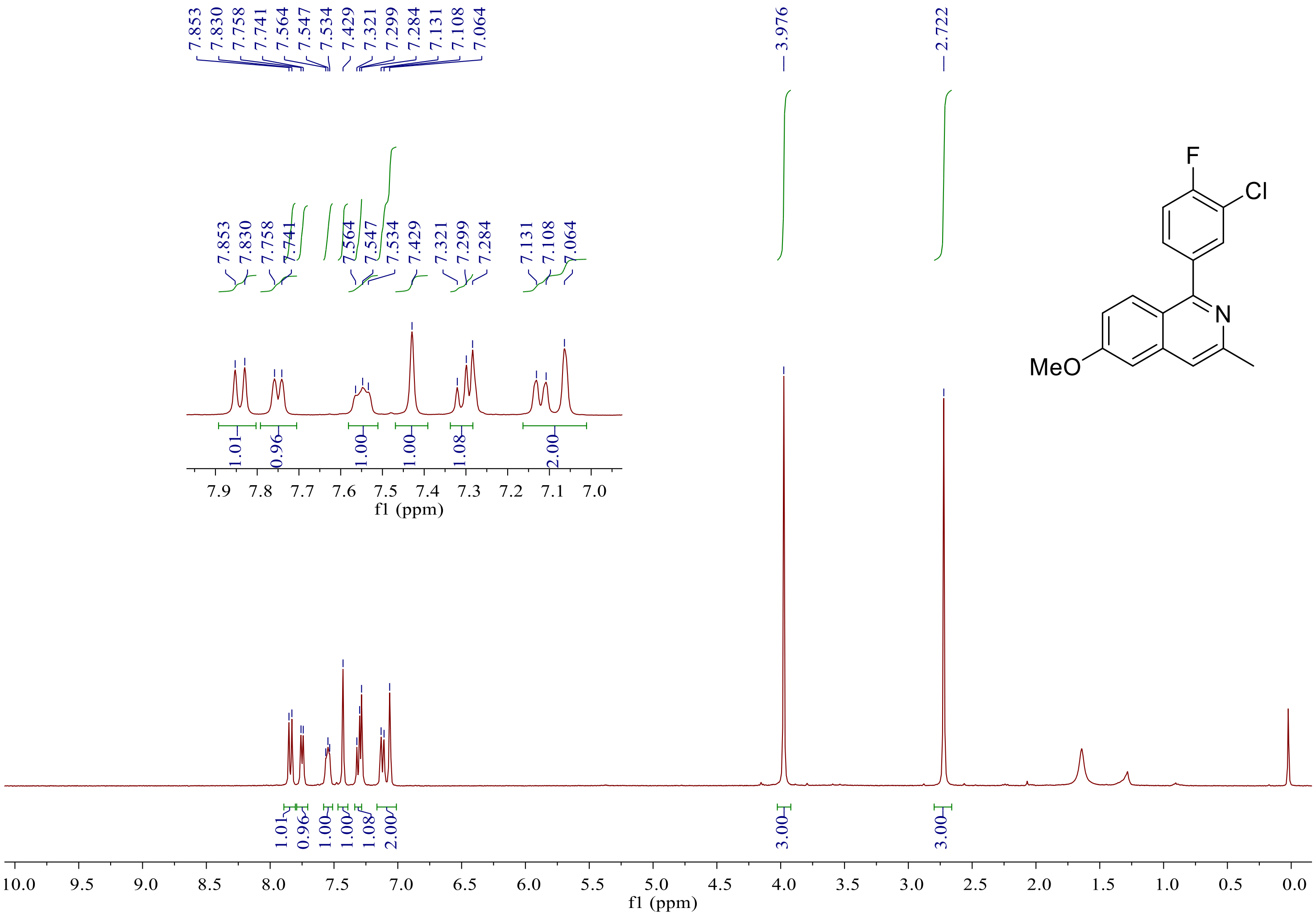

Supplementary Figure 88. ^{1}H NMR (400 MHz, $CDCl_3$) of **2f**

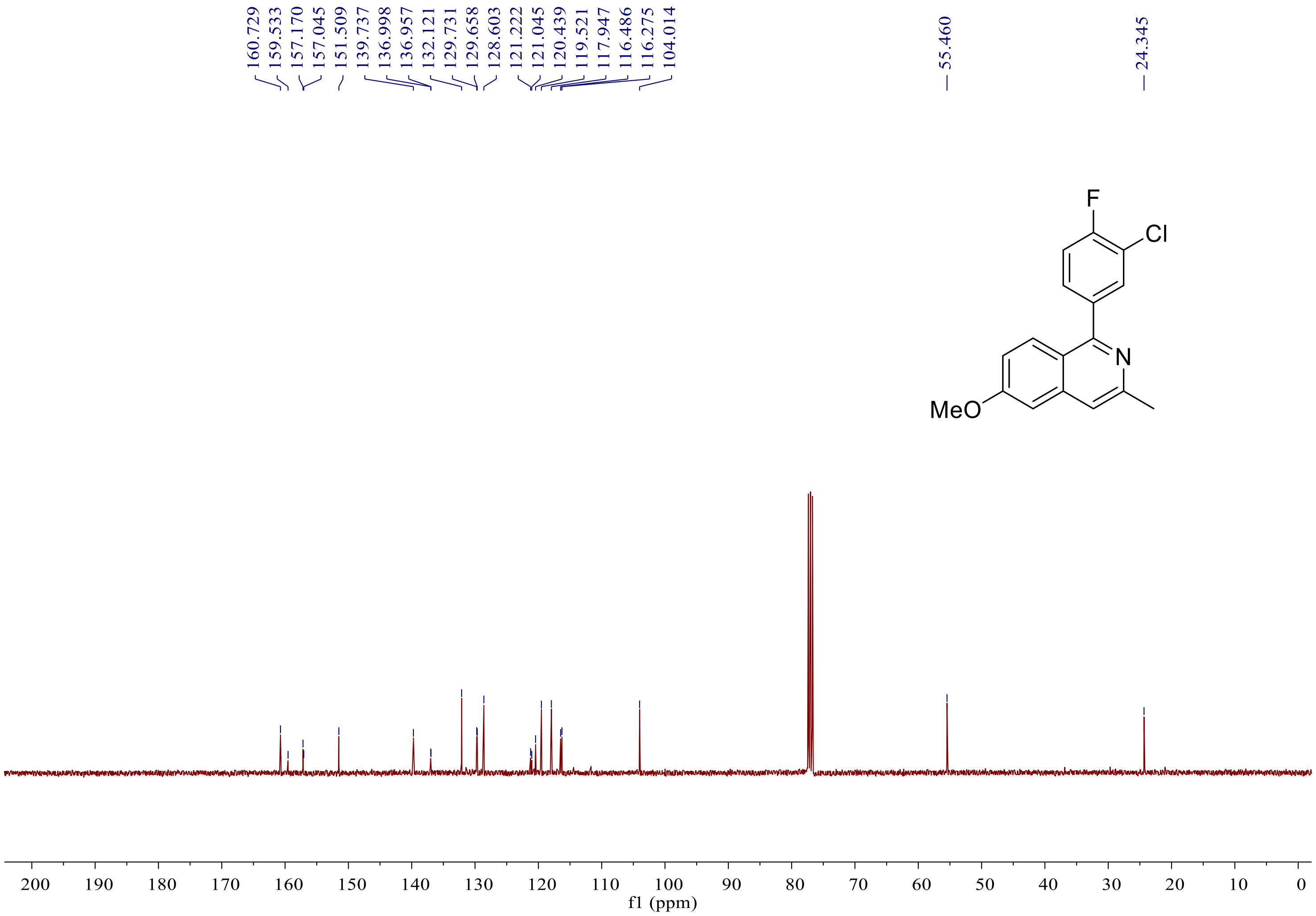

Supplementary Figure 89. ^{13}C NMR (100 MHz, $CDCl_3$) of **2f**

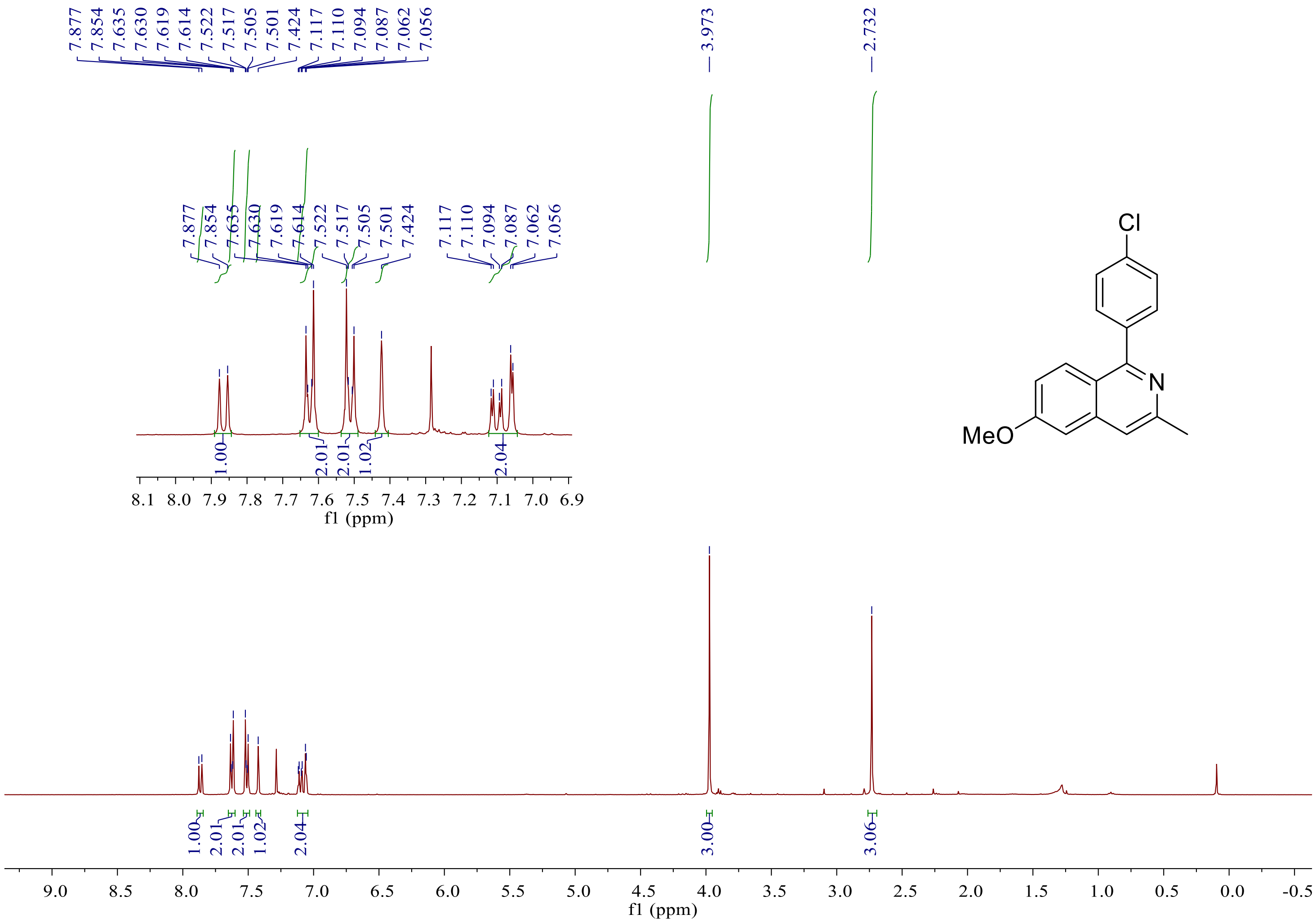

Supplementary Figure 90. ^{1}H NMR (400 MHz, $CDCl_3$) of **2g**

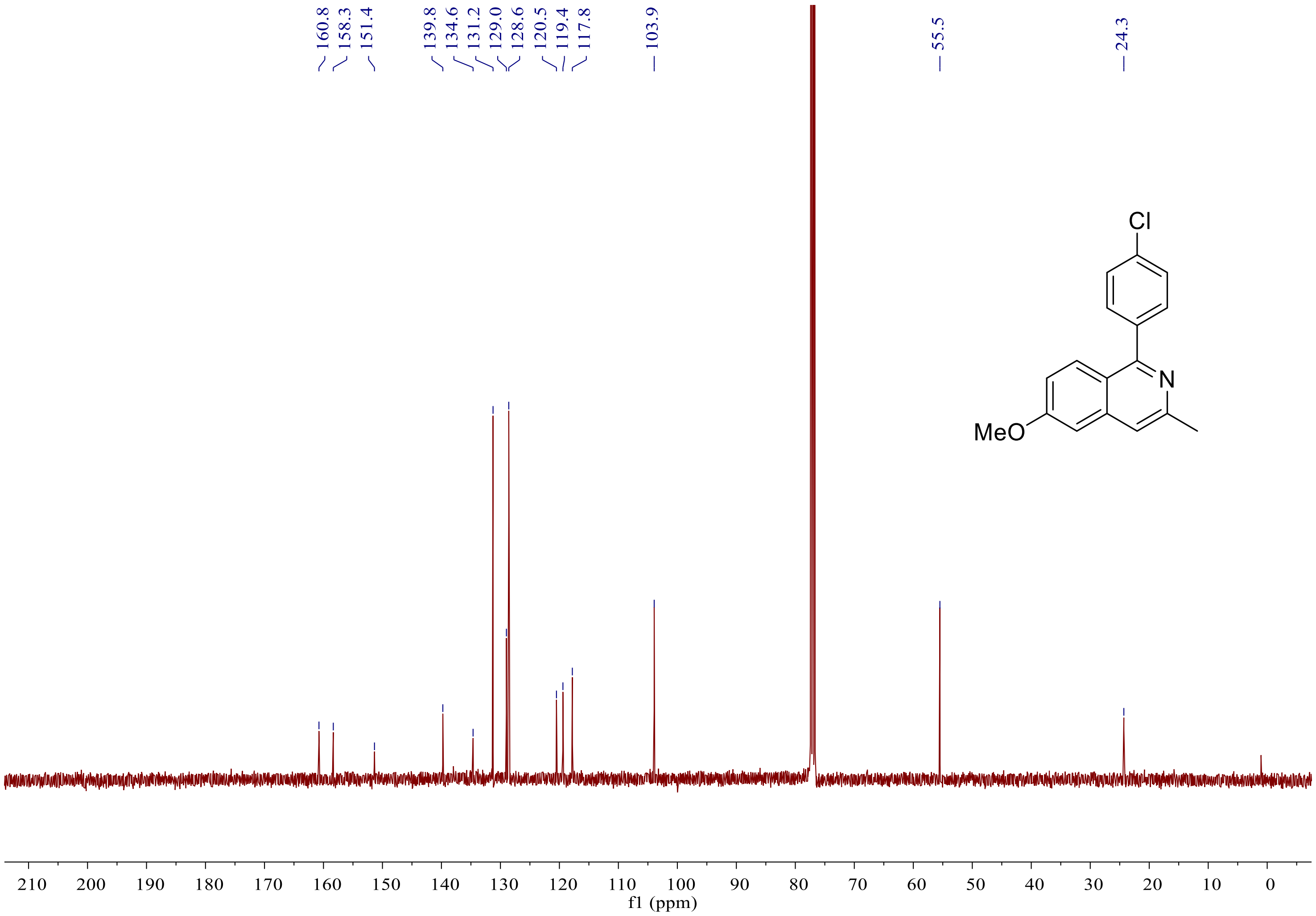

Supplementary Figure 91. ^{13}C NMR (100 MHz, $CDCl_3$) of **2g**

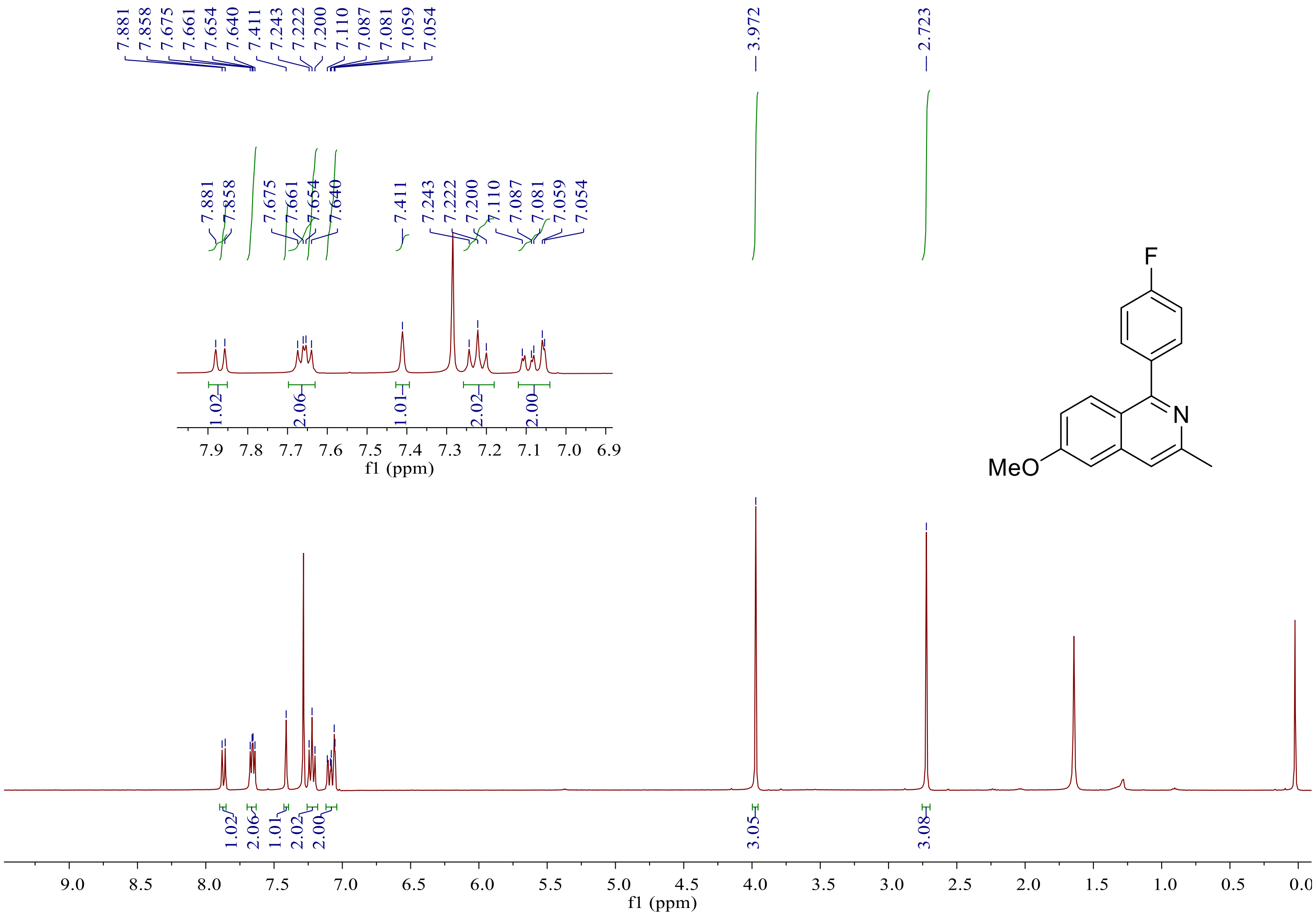

Supplementary Figure 92. ^{1}H NMR (400 MHz, $CDCl_3$) of **2h**

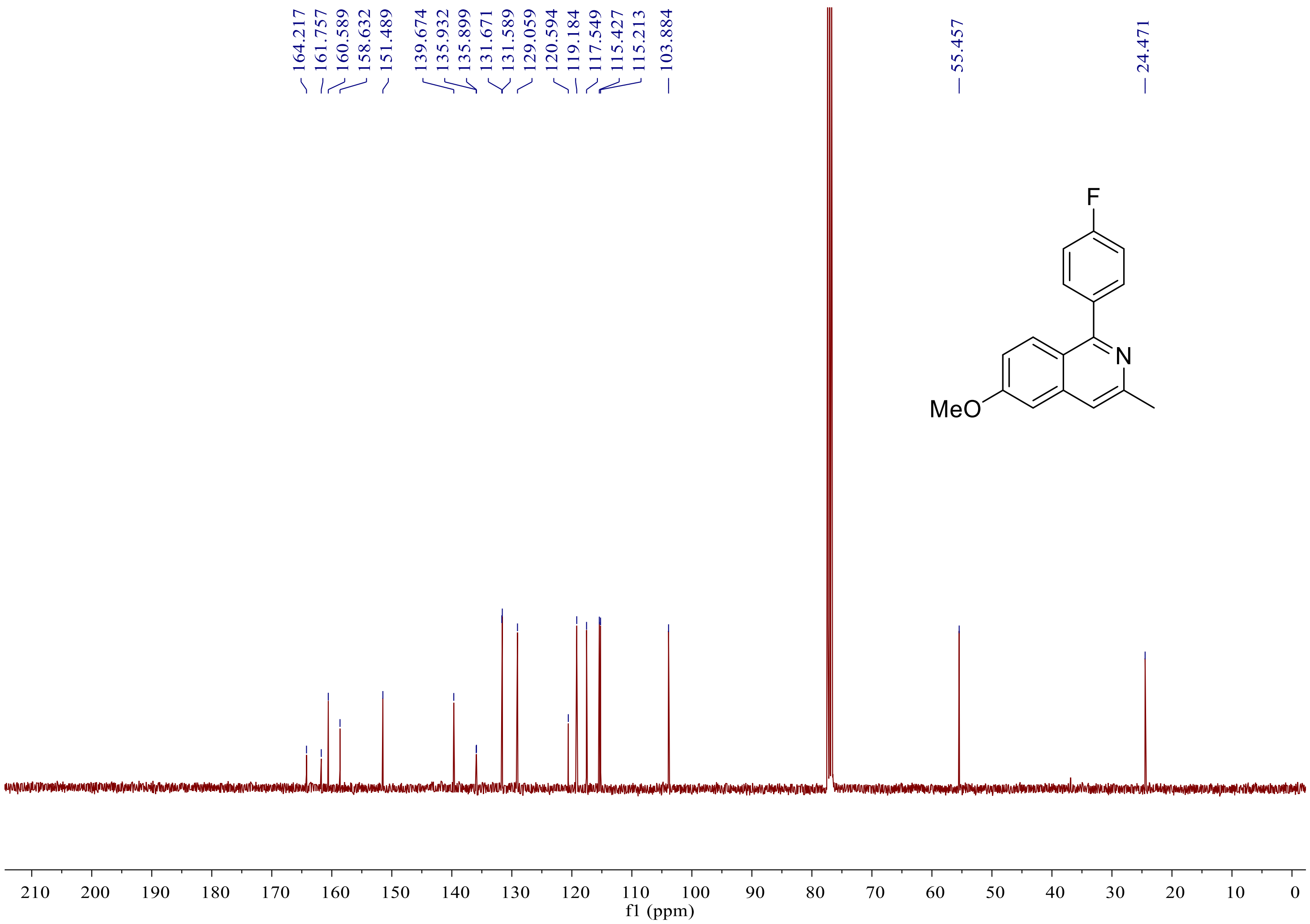

Supplementary Figure 93. ^{13}C NMR (100 MHz, $CDCl_3$) of **2h**

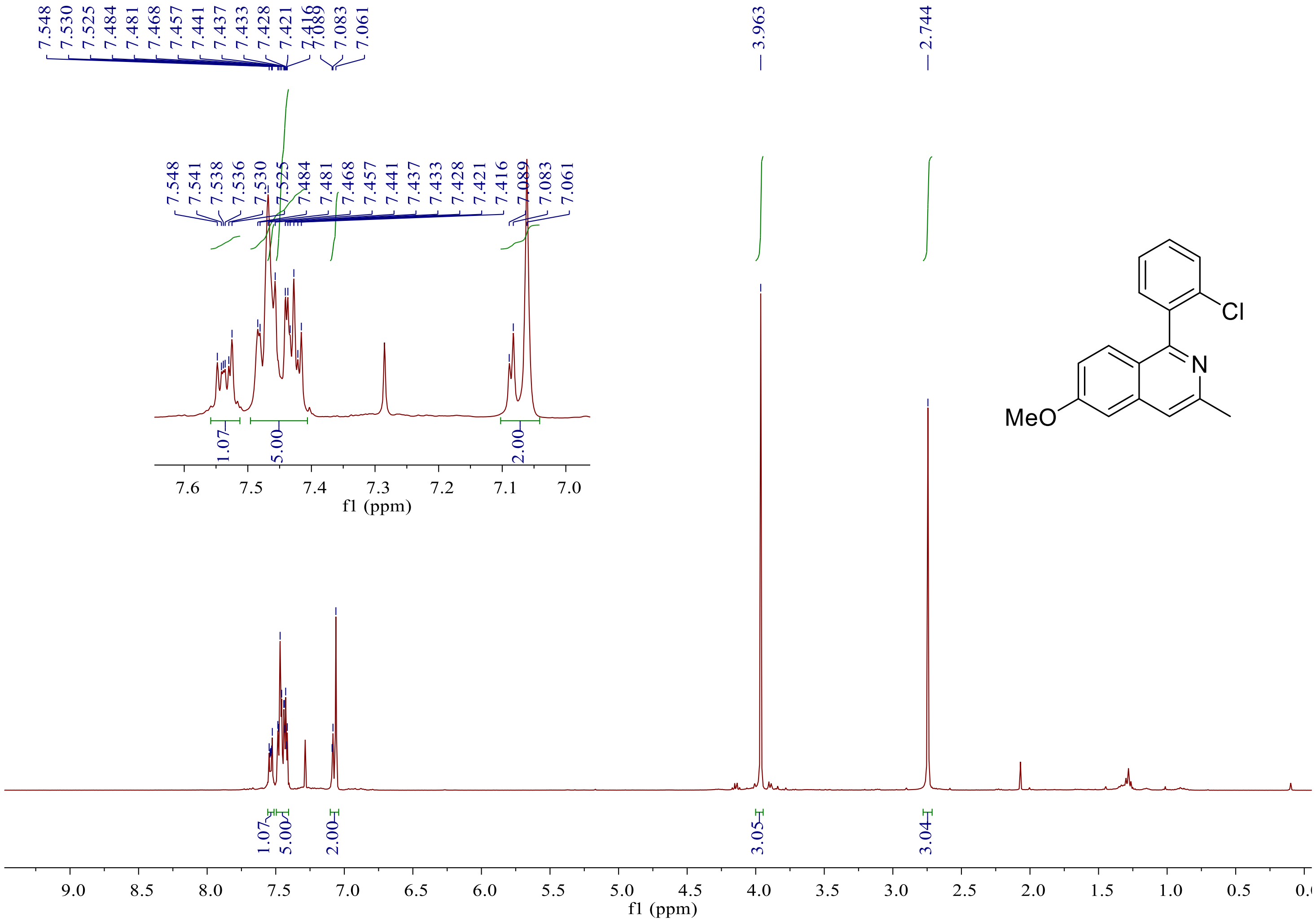

Supplementary Figure 94. ^{1}H NMR (400 MHz, $CDCl_3$) of **2i**

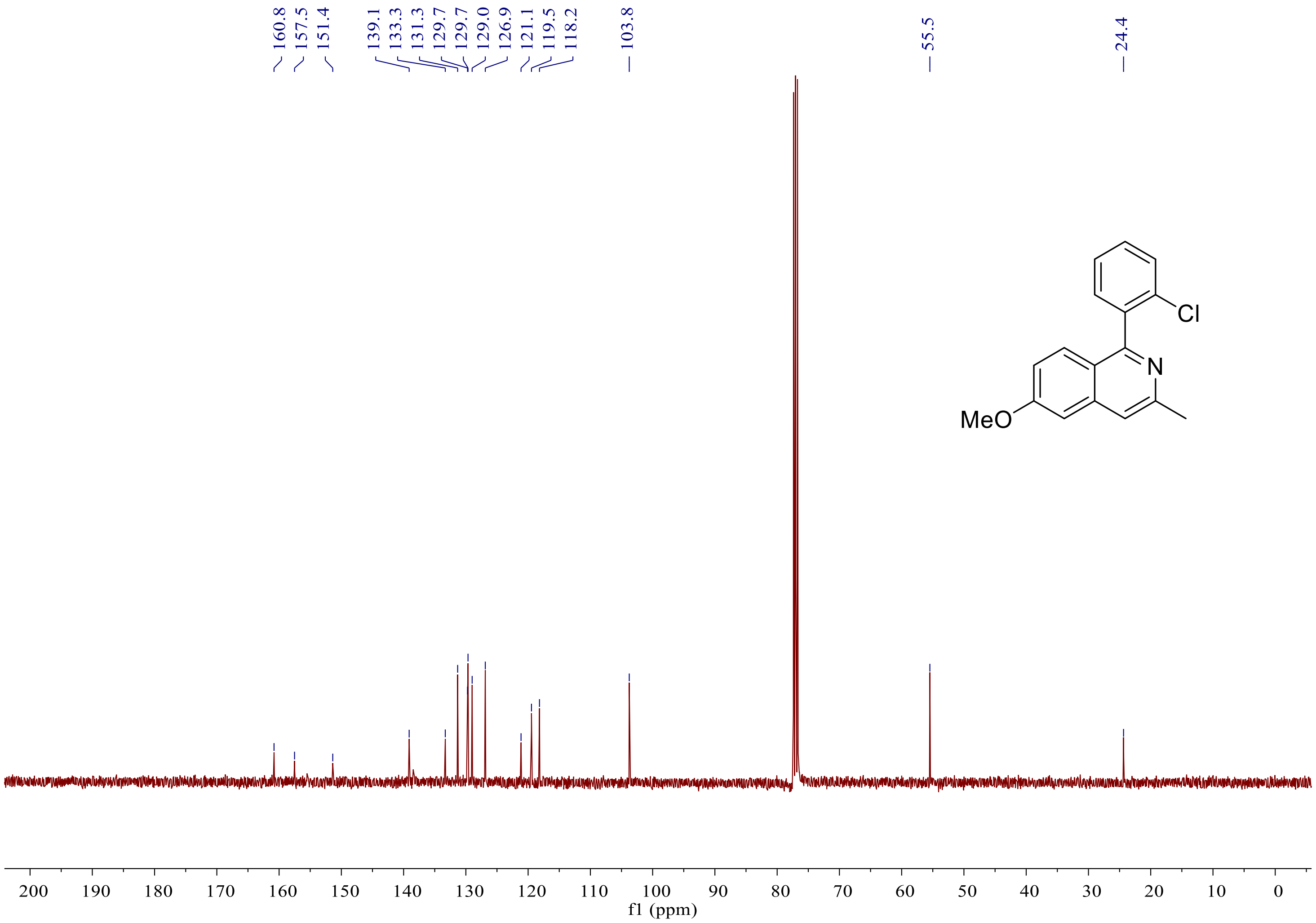

Supplementary Figure 95. ^{13}C NMR (100 MHz, $CDCl_3$) of **2i**

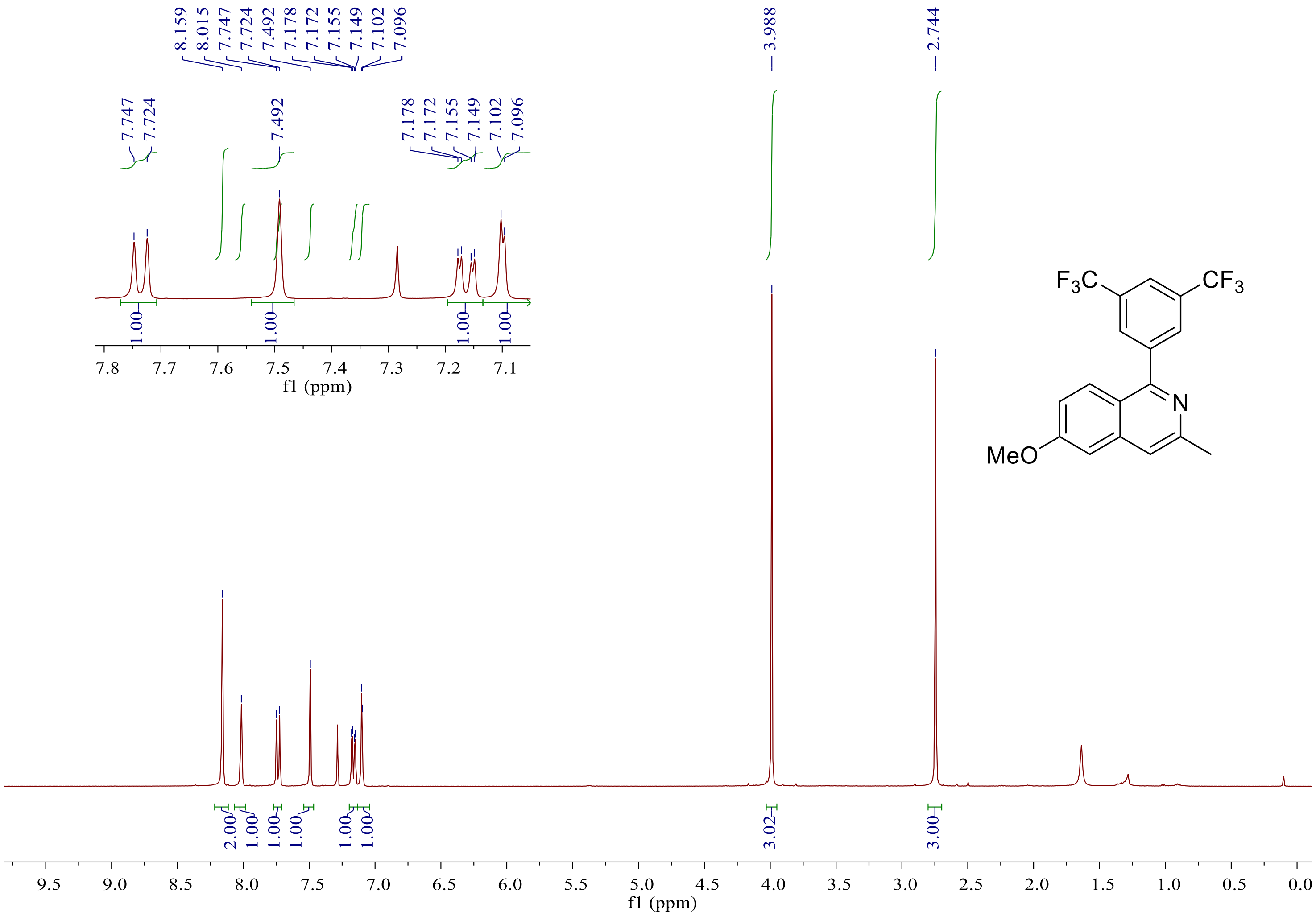

Supplementary Figure 96. ^{1}H NMR (400 MHz, $CDCl_3$) of **2j**

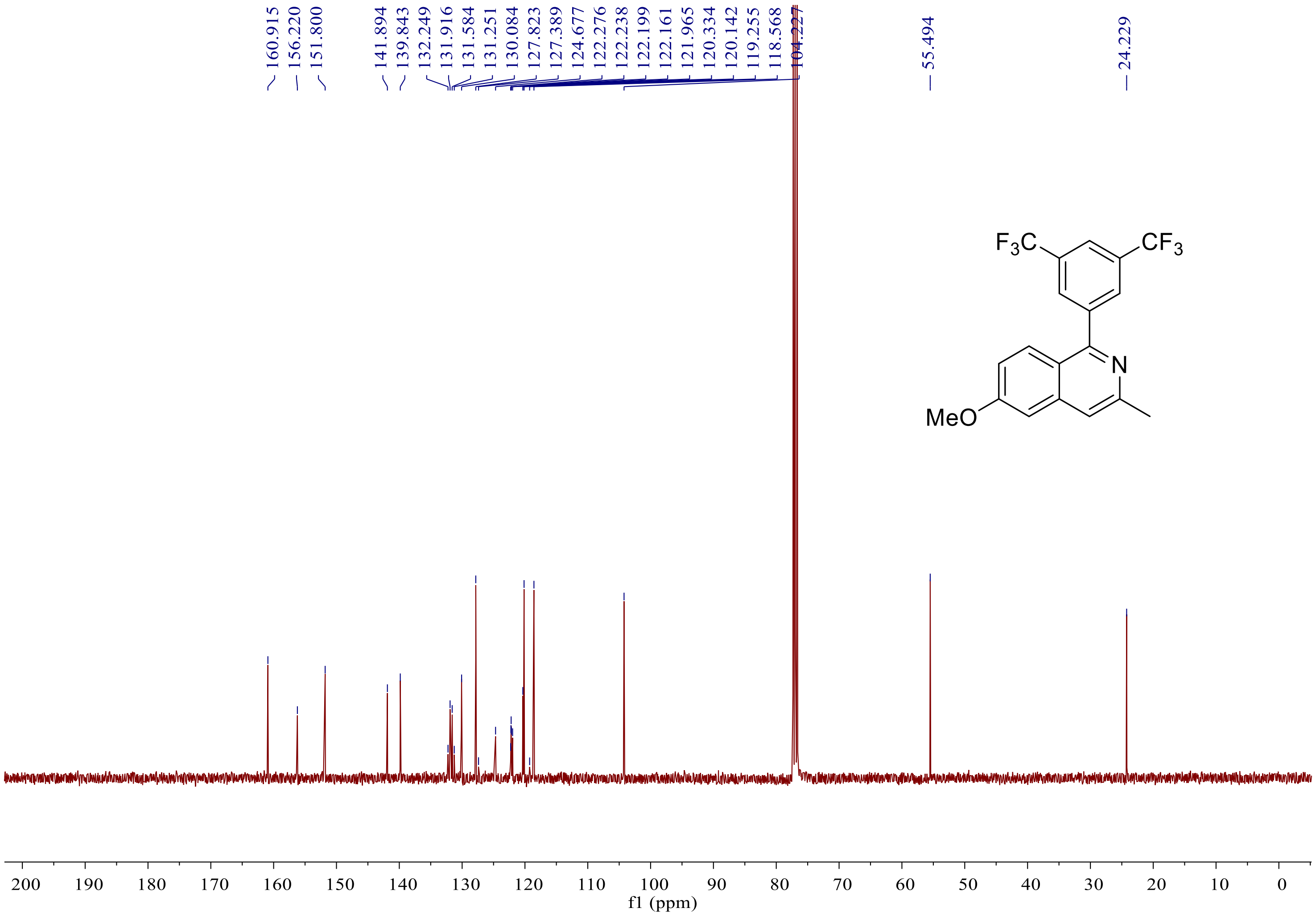

Supplementary Figure 97. ^{13}C NMR (100 MHz, $CDCl_3$) of **2j**

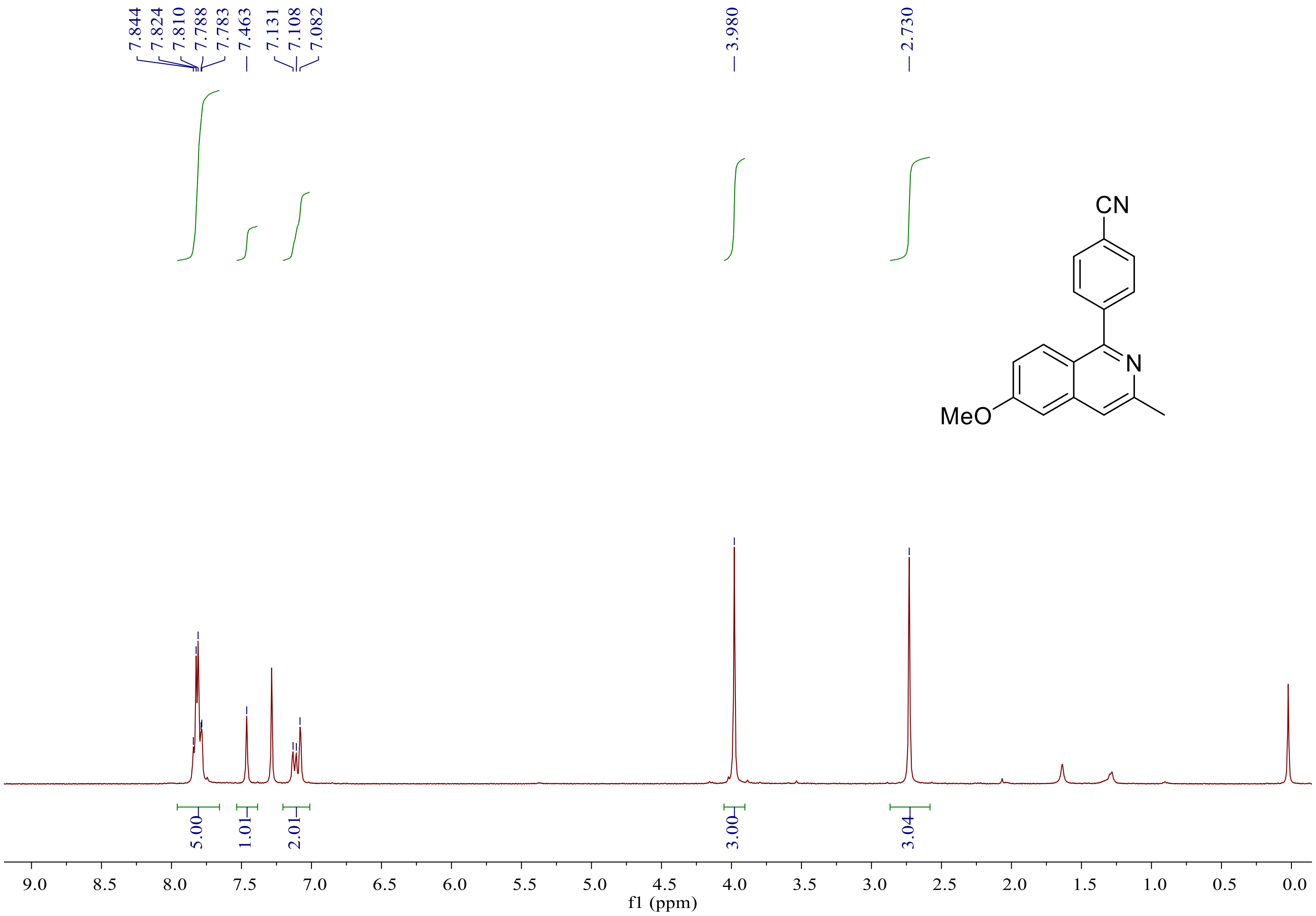

Supplementary Figure 98. ^{1}H NMR (400 MHz, $CDCl_3$) of **2k**

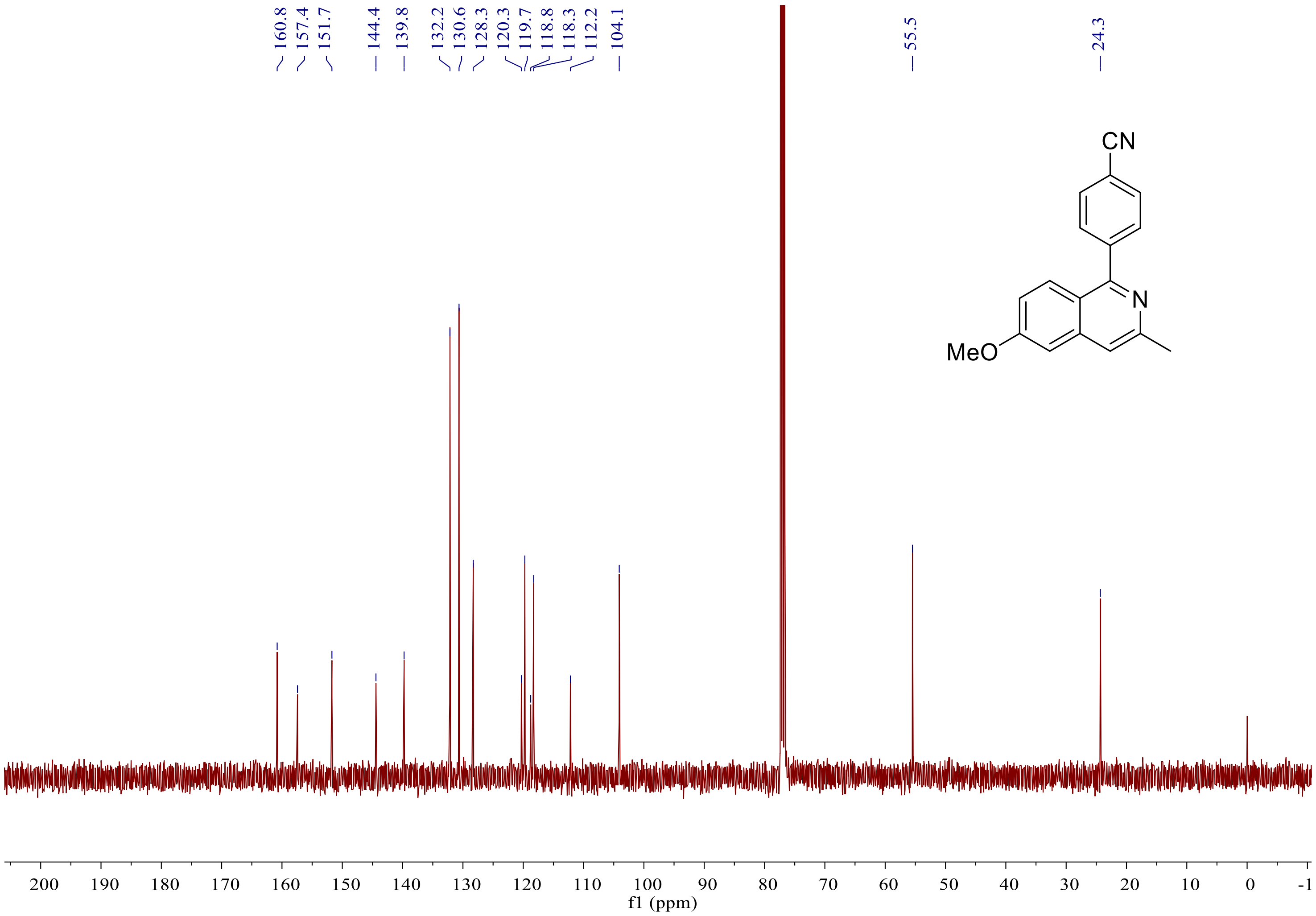

Supplementary Figure 99. ^{13}C NMR (100 MHz, $CDCl_3$) of **2k**

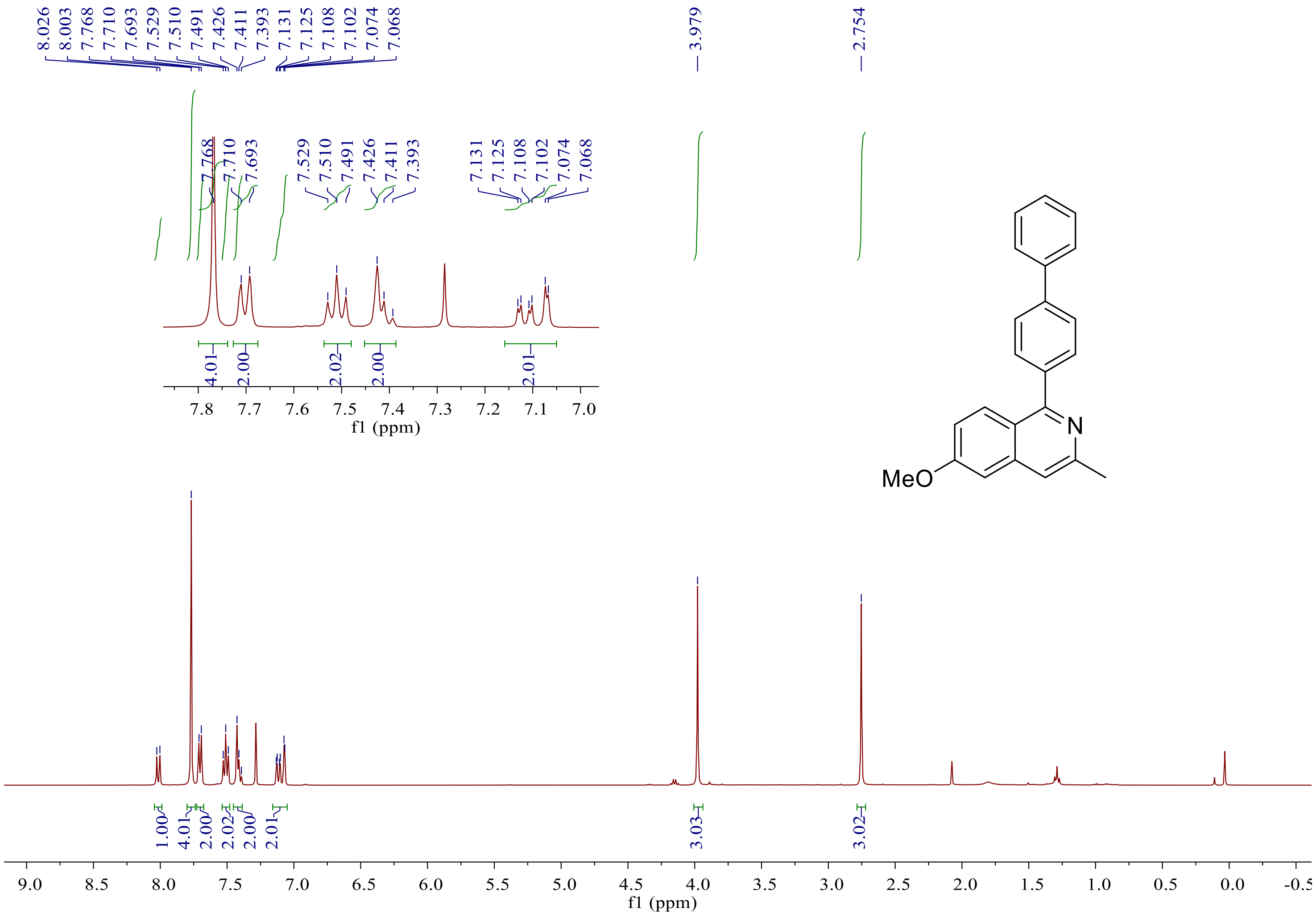

Supplementary Figure 100. ^{1}H NMR (400 MHz, $CDCl_3$) of **2l**

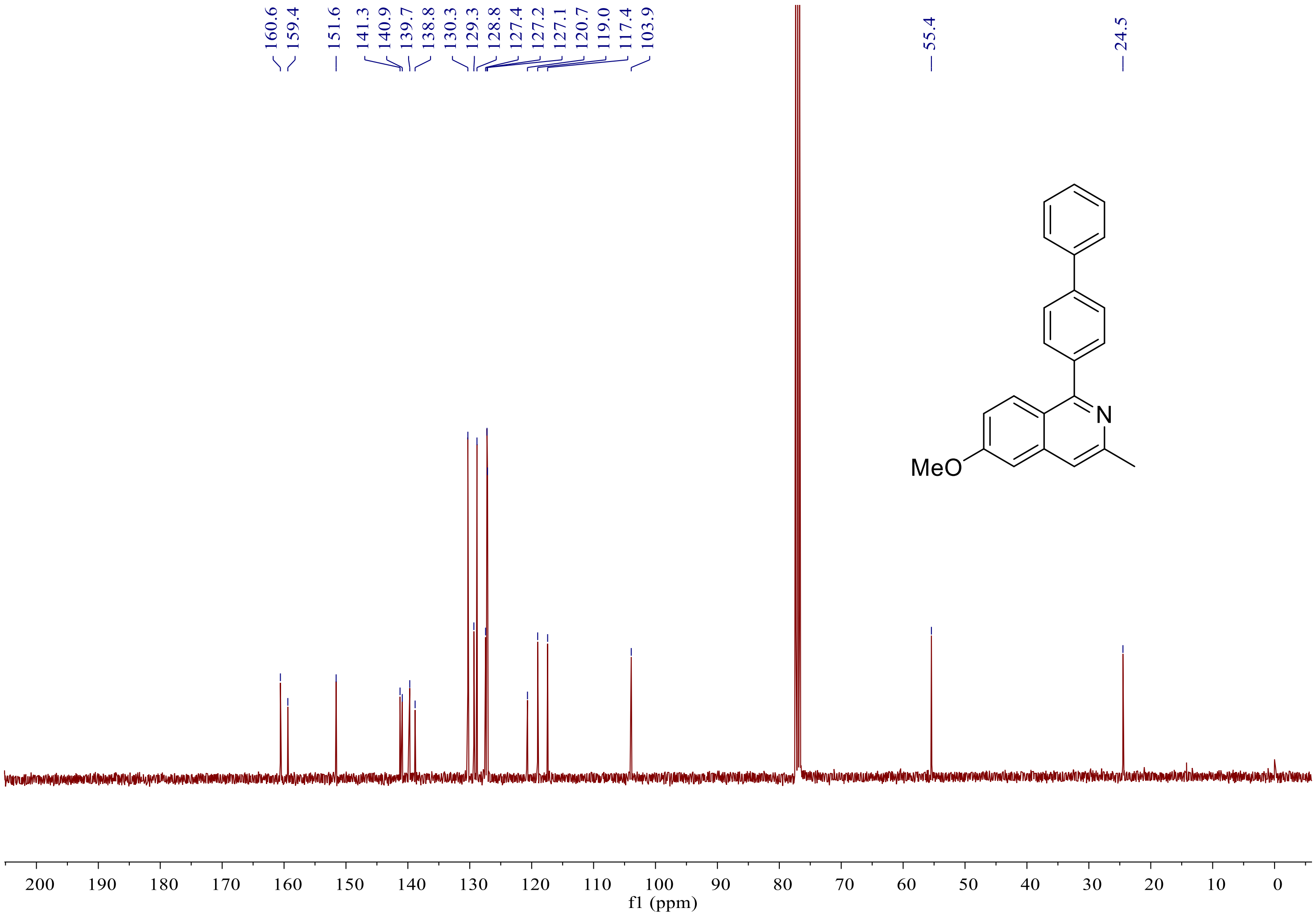

Supplementary Figure 101. ^{13}C NMR (100 MHz, $CDCl_3$) of **2l**

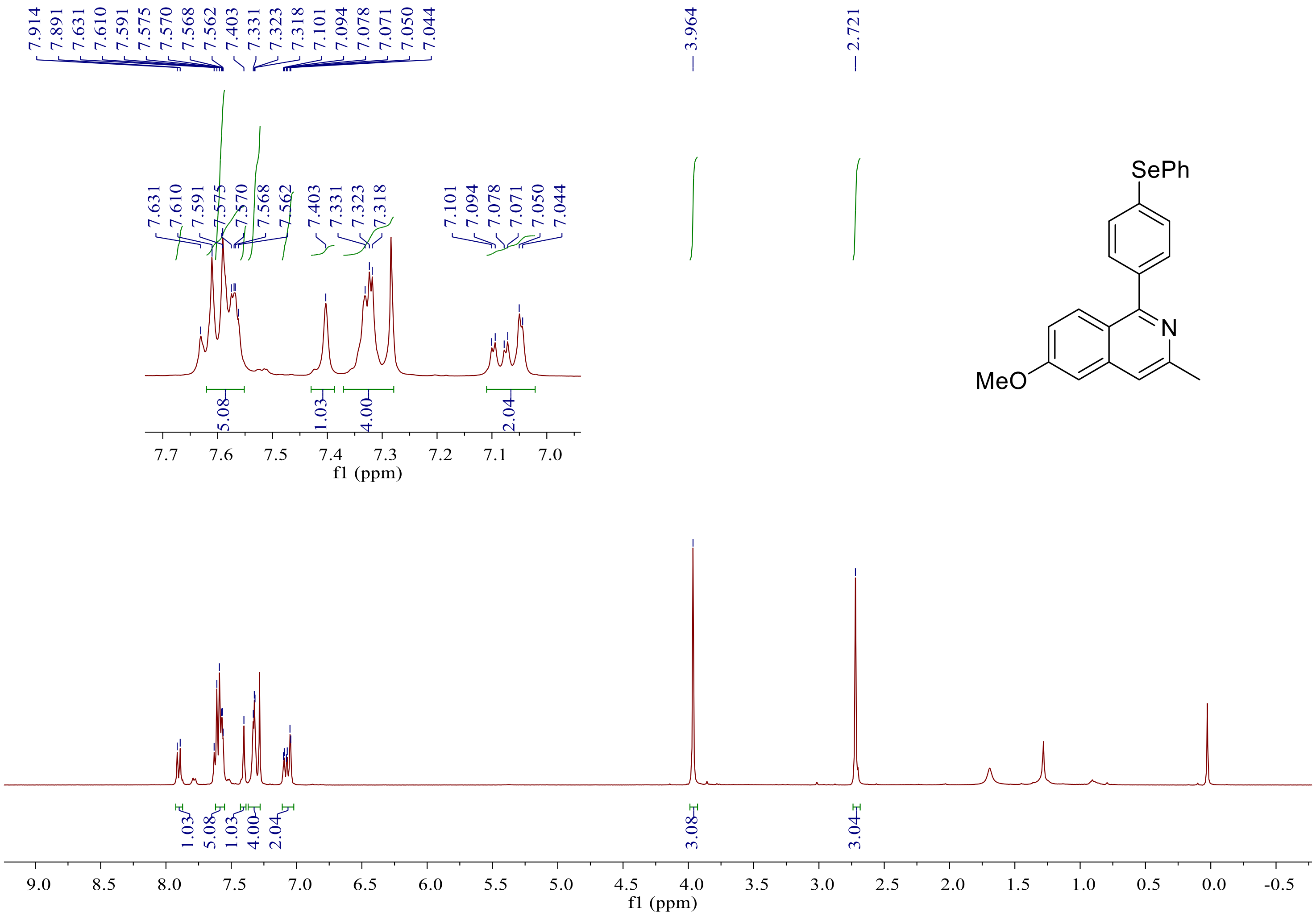

Supplementary Figure 102. ^{1}H NMR (400 MHz, $CDCl_3$) of **2m**

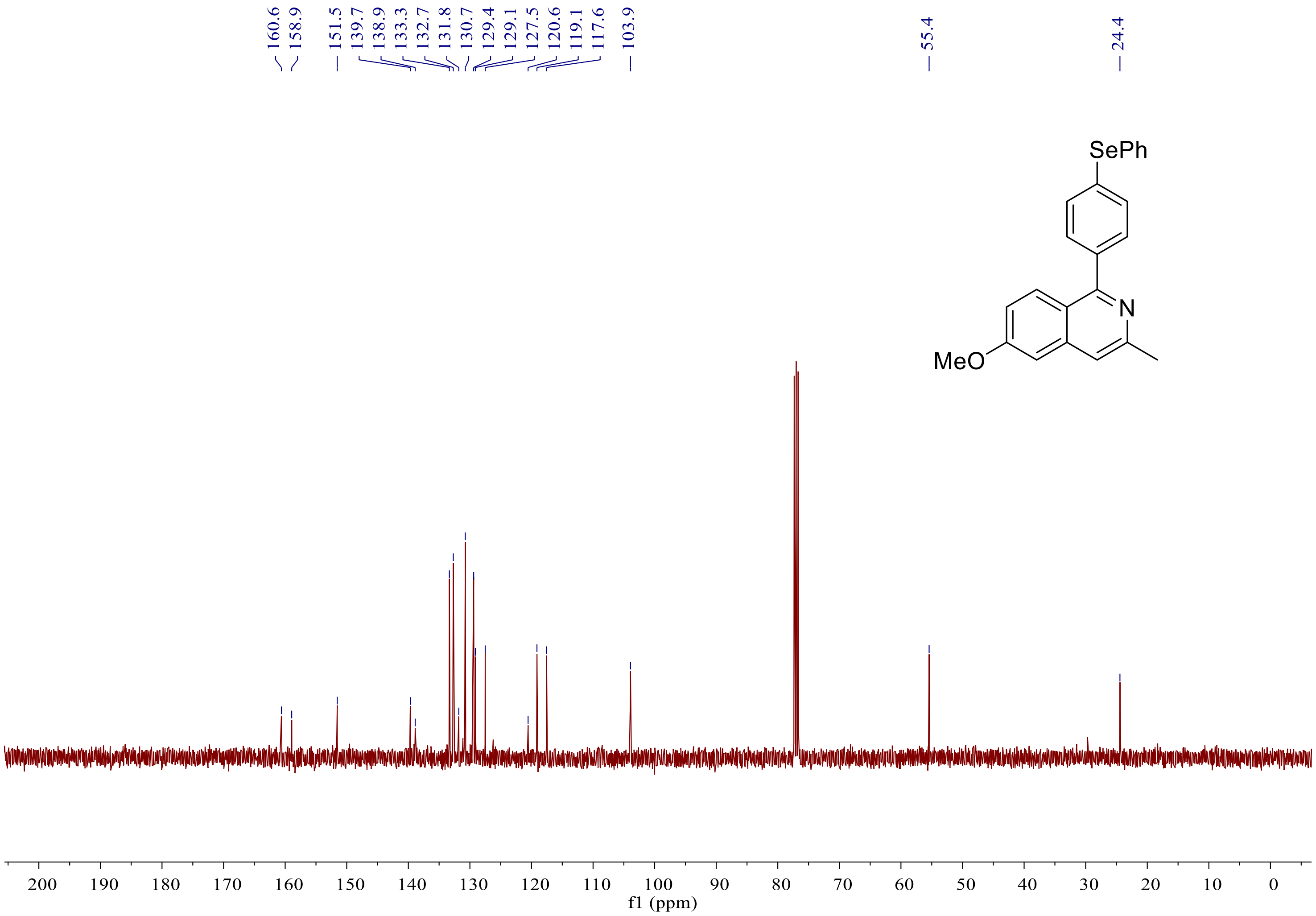

Supplementary Figure 103. ^{13}C NMR (100 MHz, $CDCl_3$) of **2m**

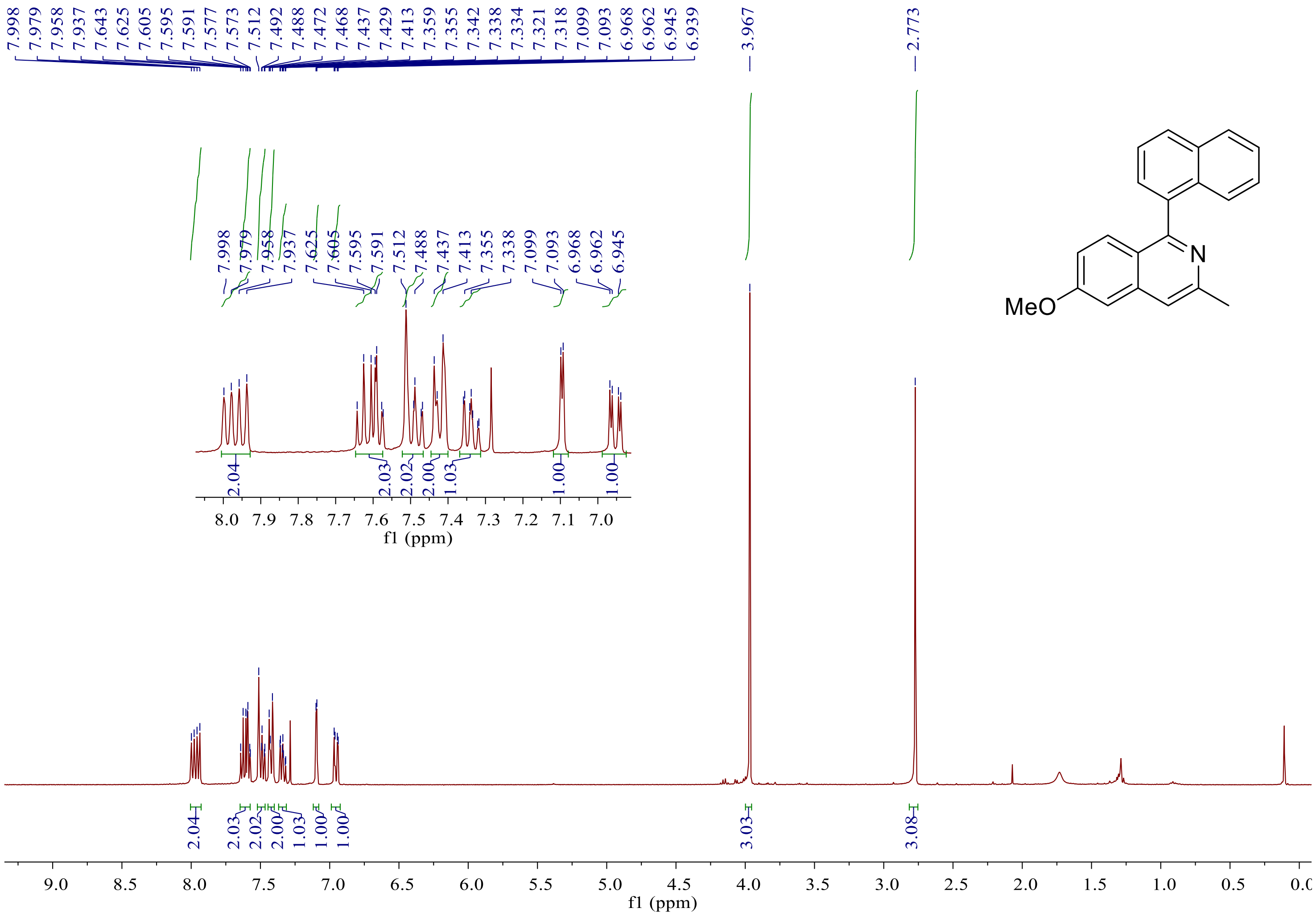

Supplementary Figure 104. ^{1}H NMR (400 MHz, $CDCl_3$) of **2n**

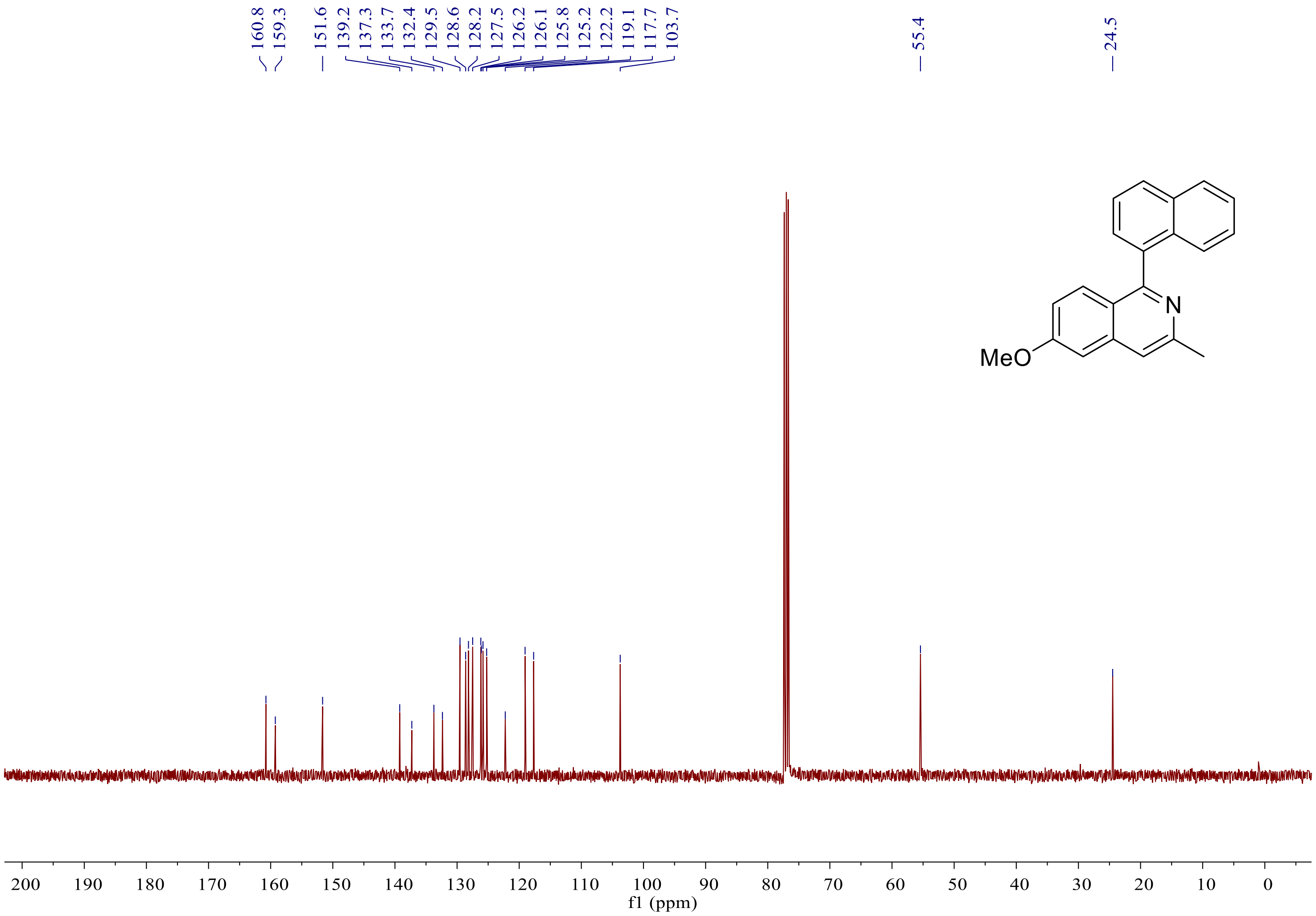

Supplementary Figure 105. ^{13}C NMR (100 MHz, $CDCl_3$) of **2n**

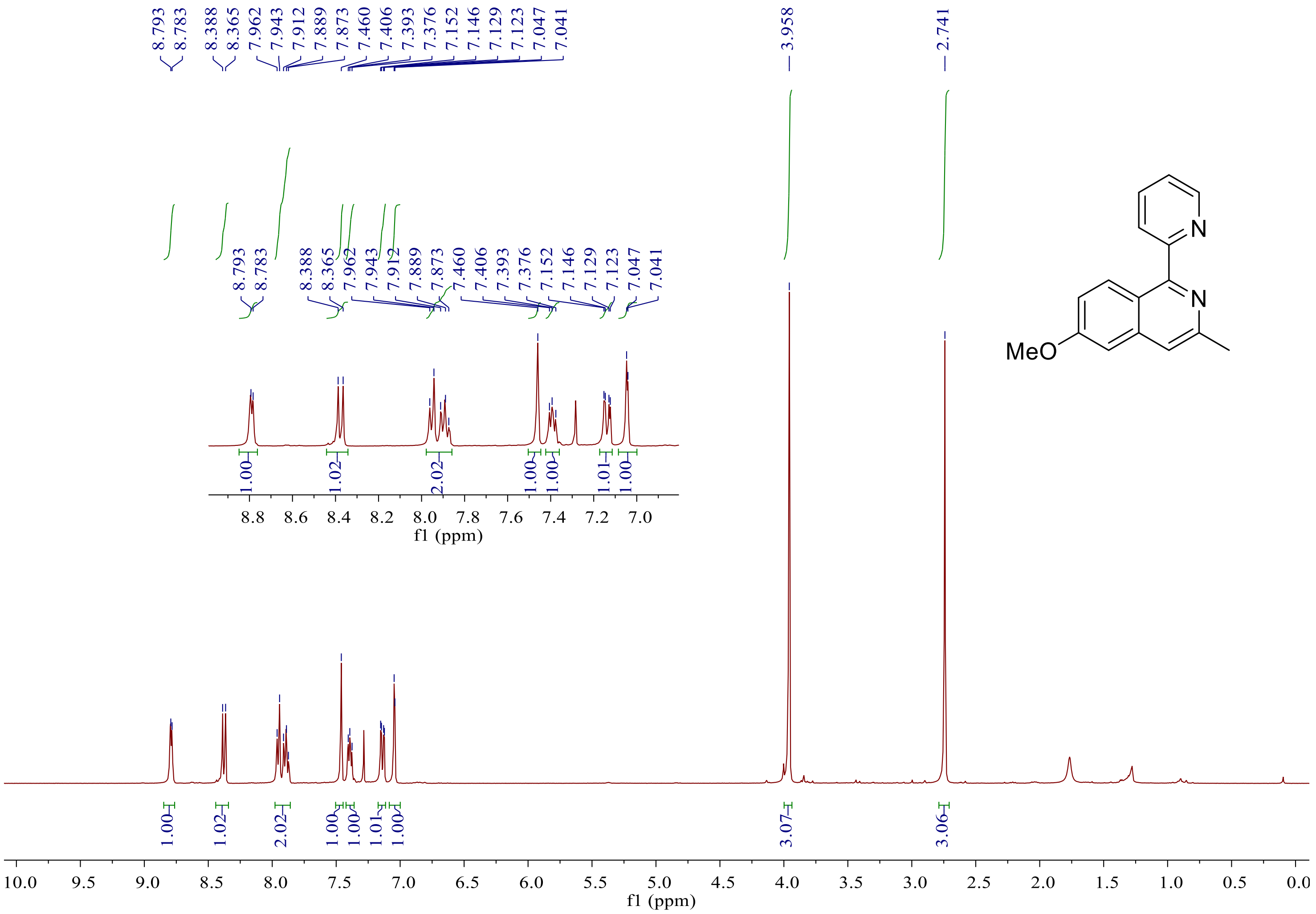

Supplementary Figure 106. ^{1}H NMR (400 MHz, $CDCl_3$) of **2o**

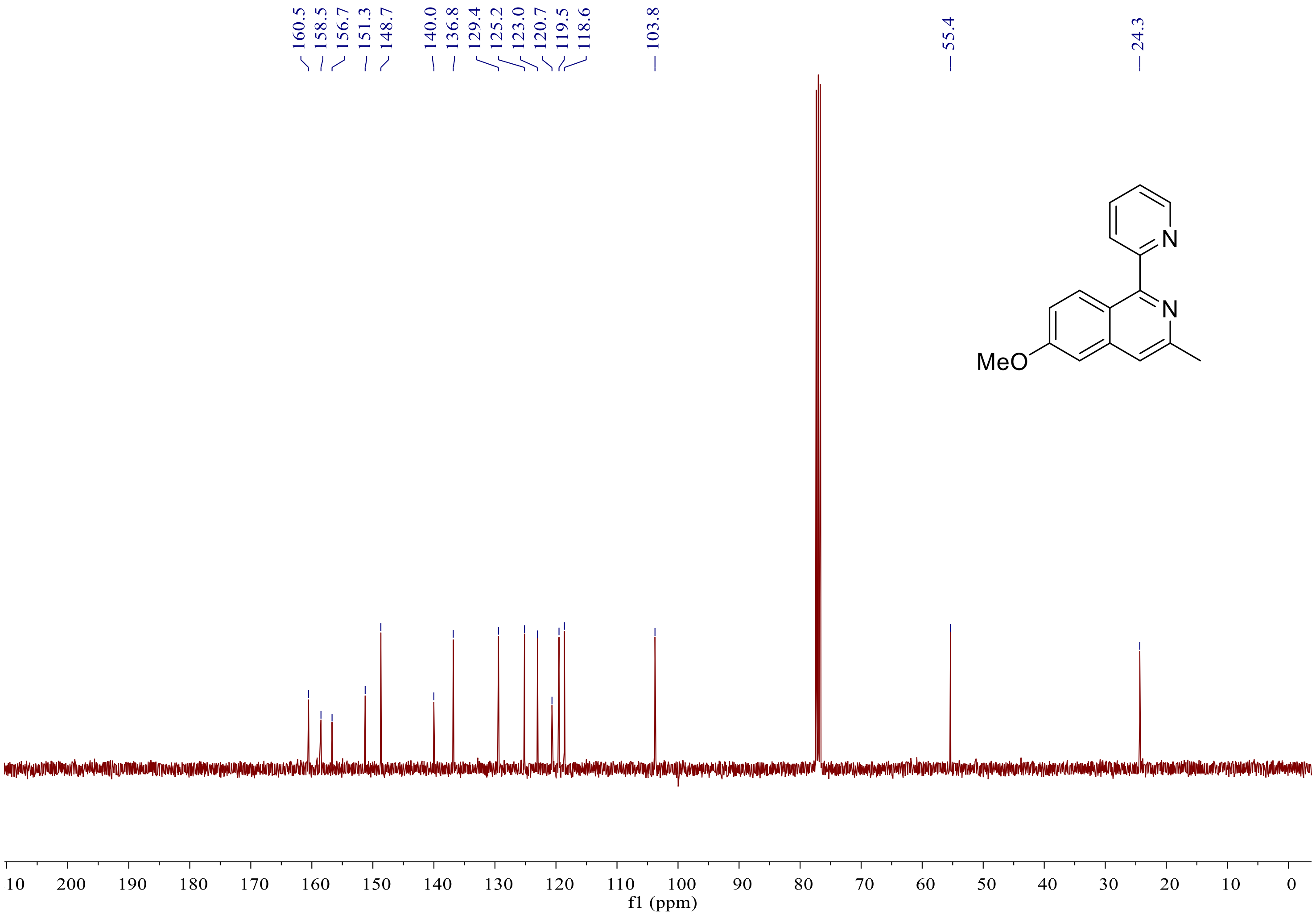

Supplementary Figure 107. ^{13}C NMR (100 MHz, $CDCl_3$) of **2o**

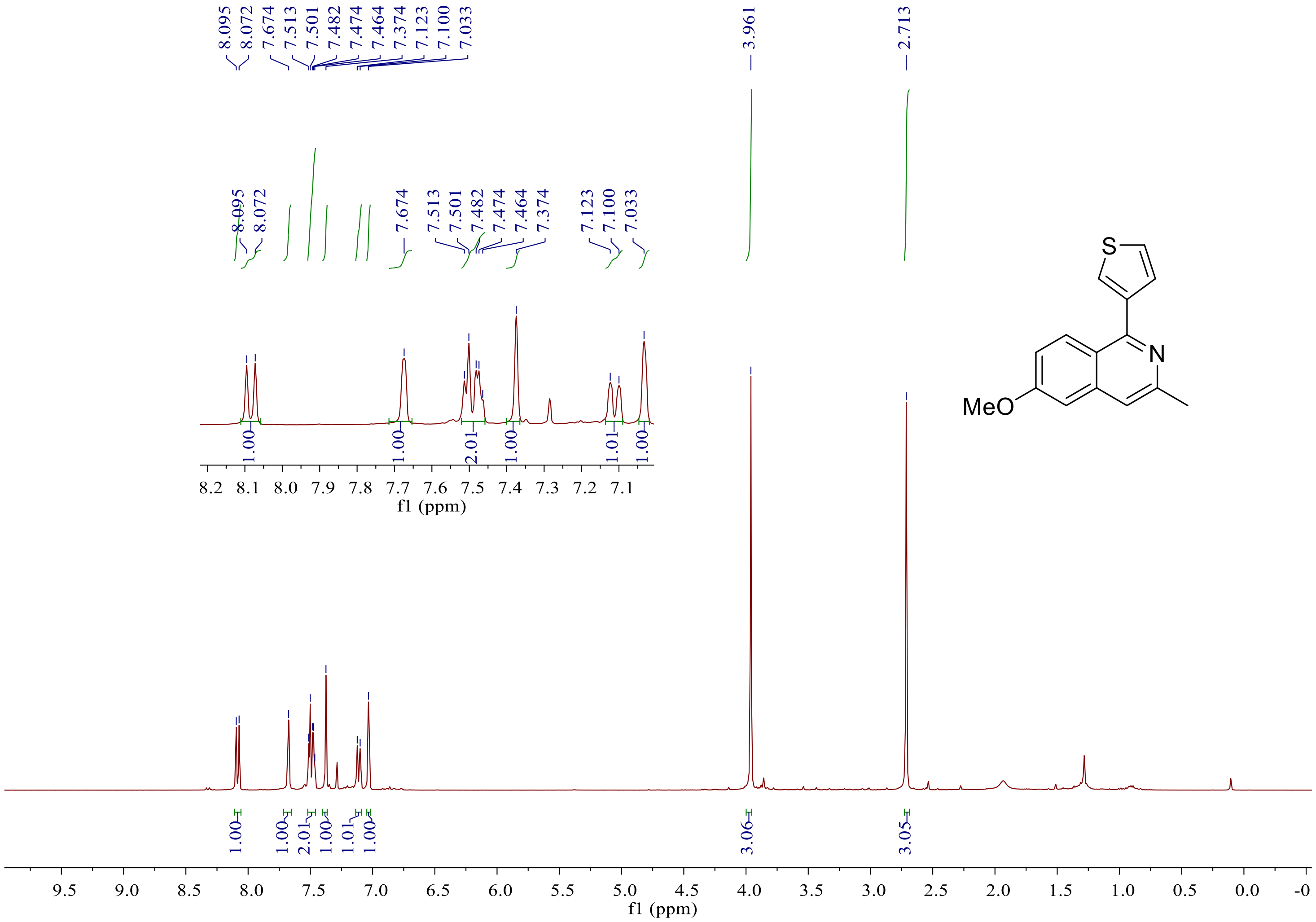

Supplementary Figure 108. ^{1}H NMR (400 MHz, $CDCl_3$) of **2p**

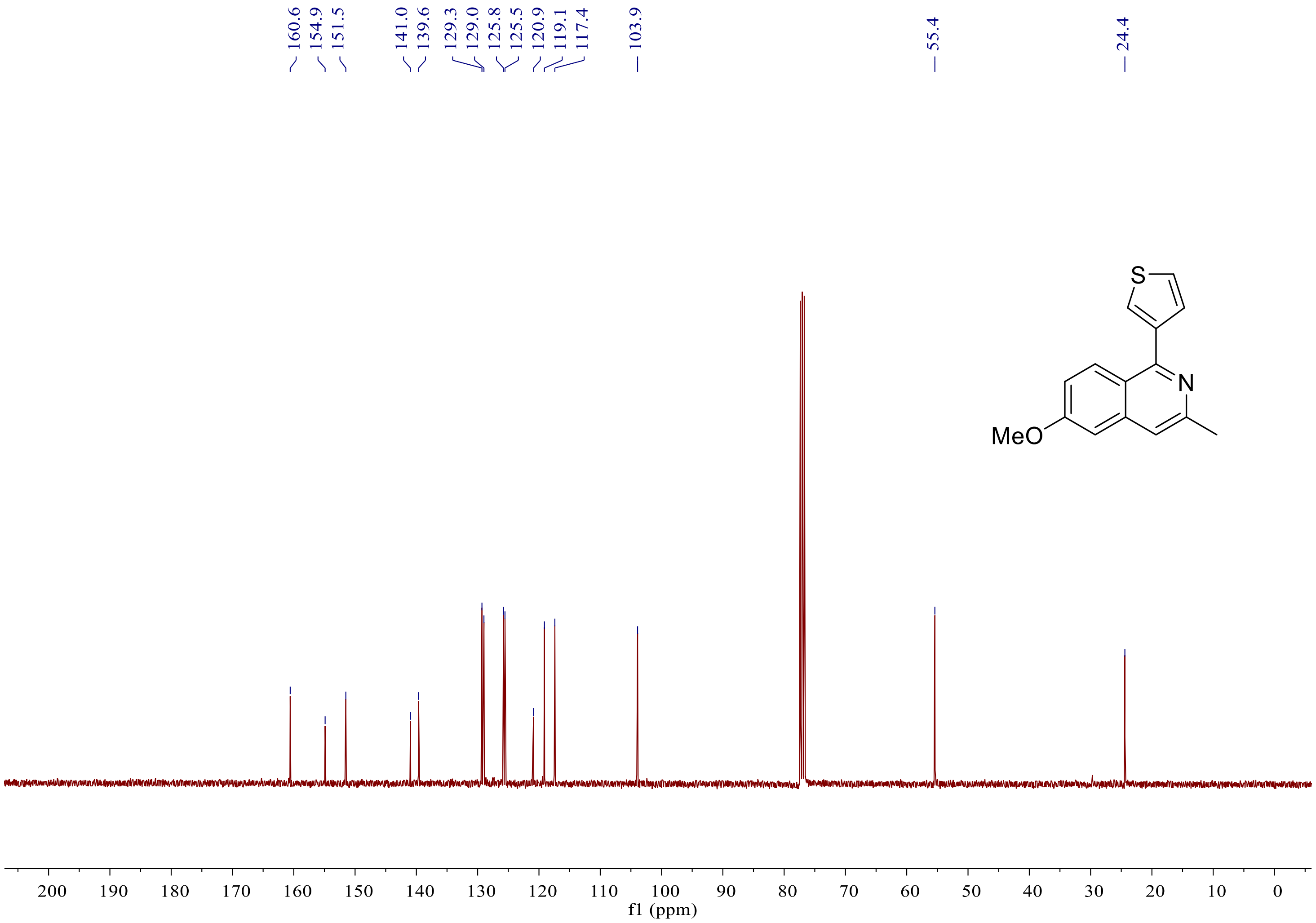

Supplementary Figure 109. ^{13}C NMR (100 MHz, $CDCl_3$) of **2p**

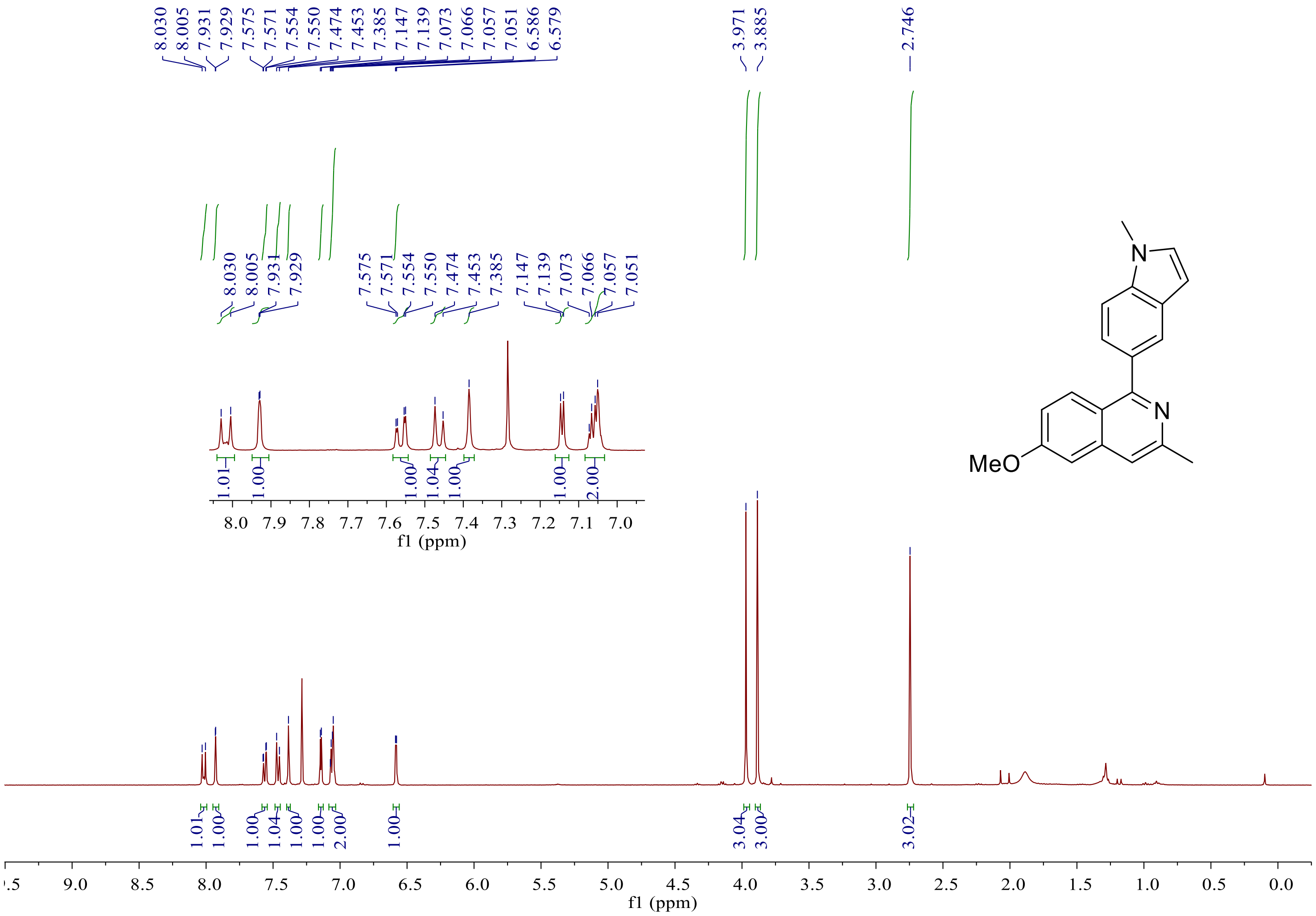

Supplementary Figure 110. ^{1}H NMR (400 MHz, $CDCl_3$) of **2q**

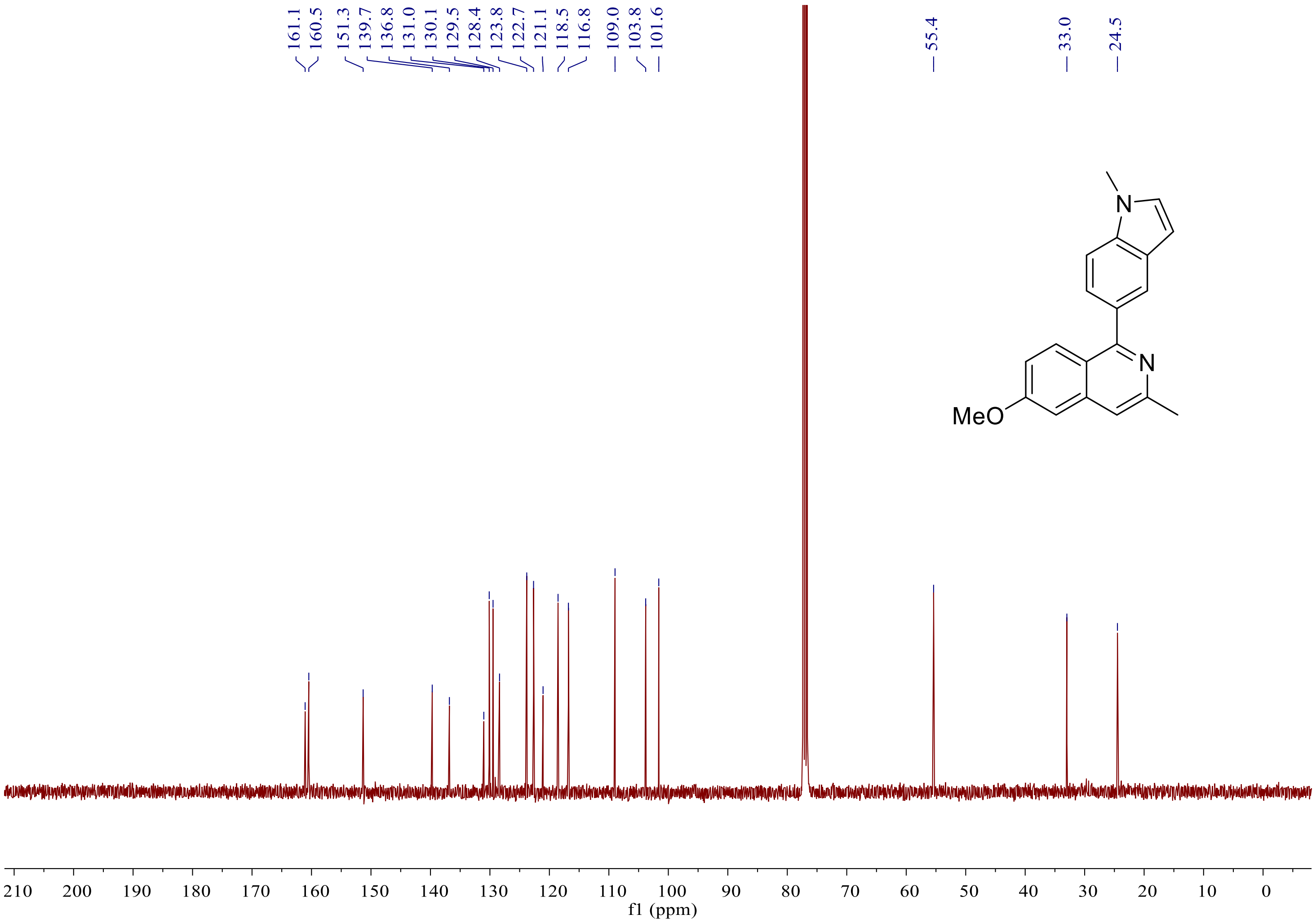

Supplementary Figure 111. ^{13}C NMR (100 MHz, $CDCl_3$) of **2q**

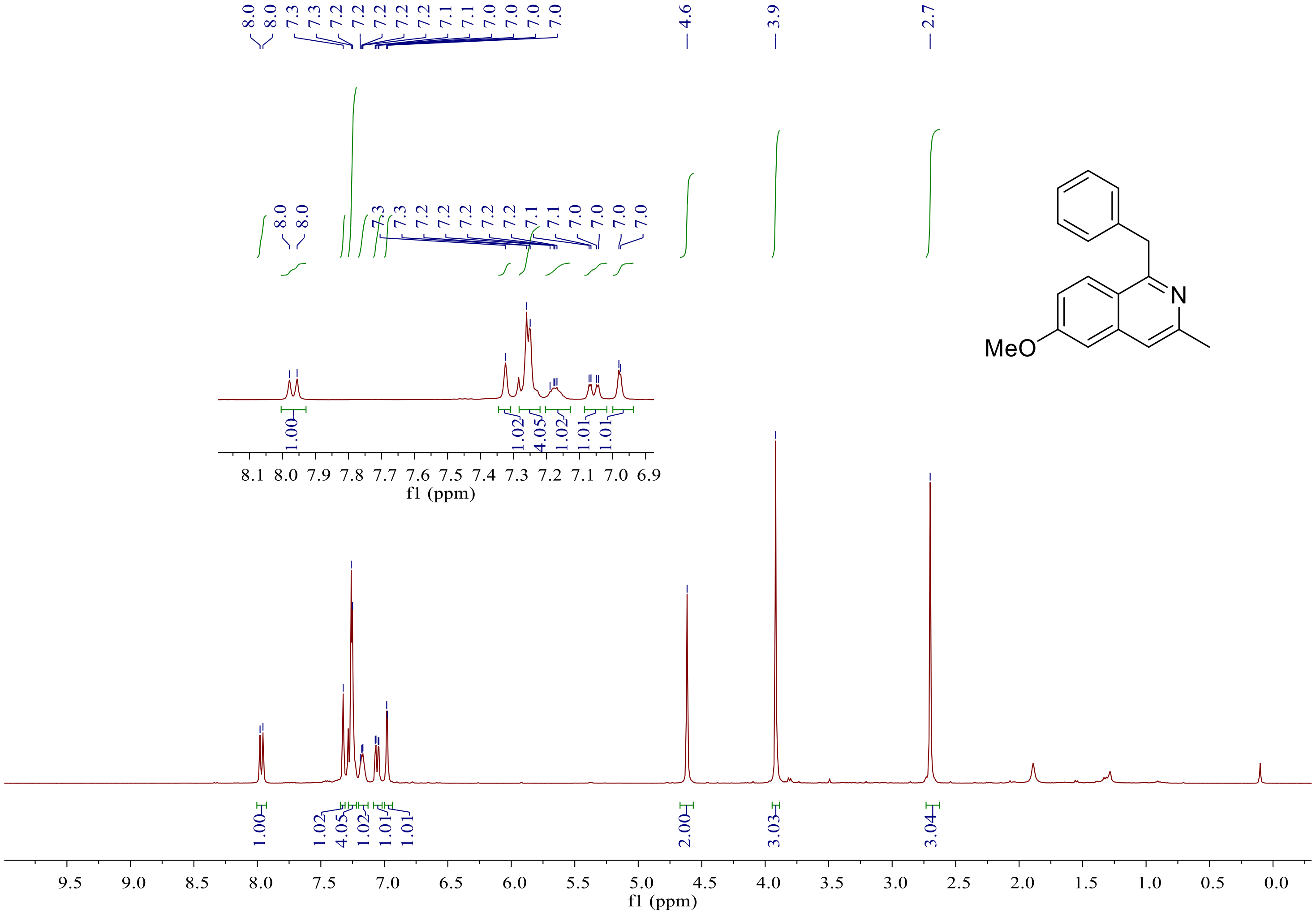

Supplementary Figure 112. ^{1}H NMR (400 MHz, $CDCl_3$) of **2r**

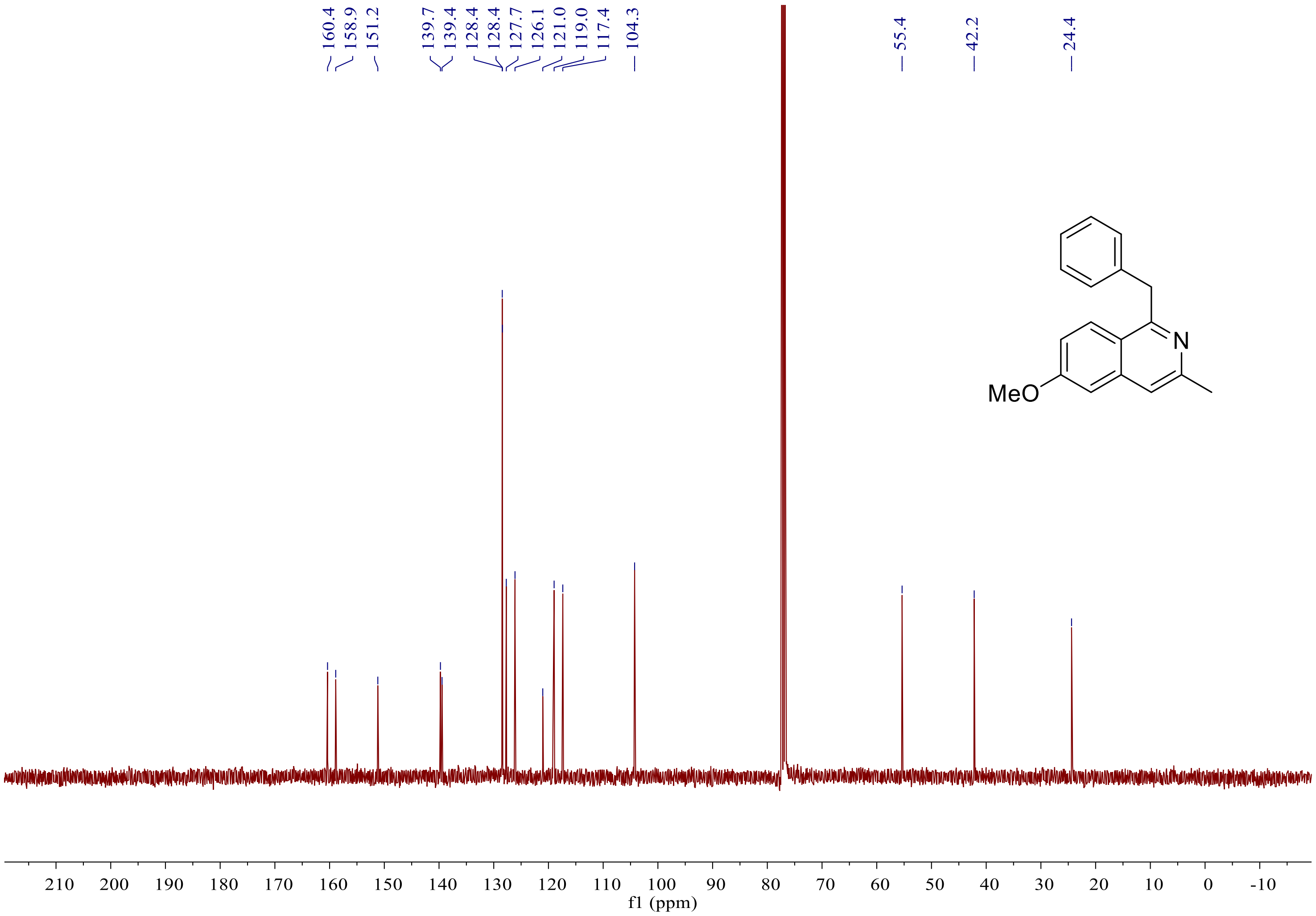

Supplementary Figure 113. ^{13}C NMR (100 MHz, $CDCl_3$) of **2r**

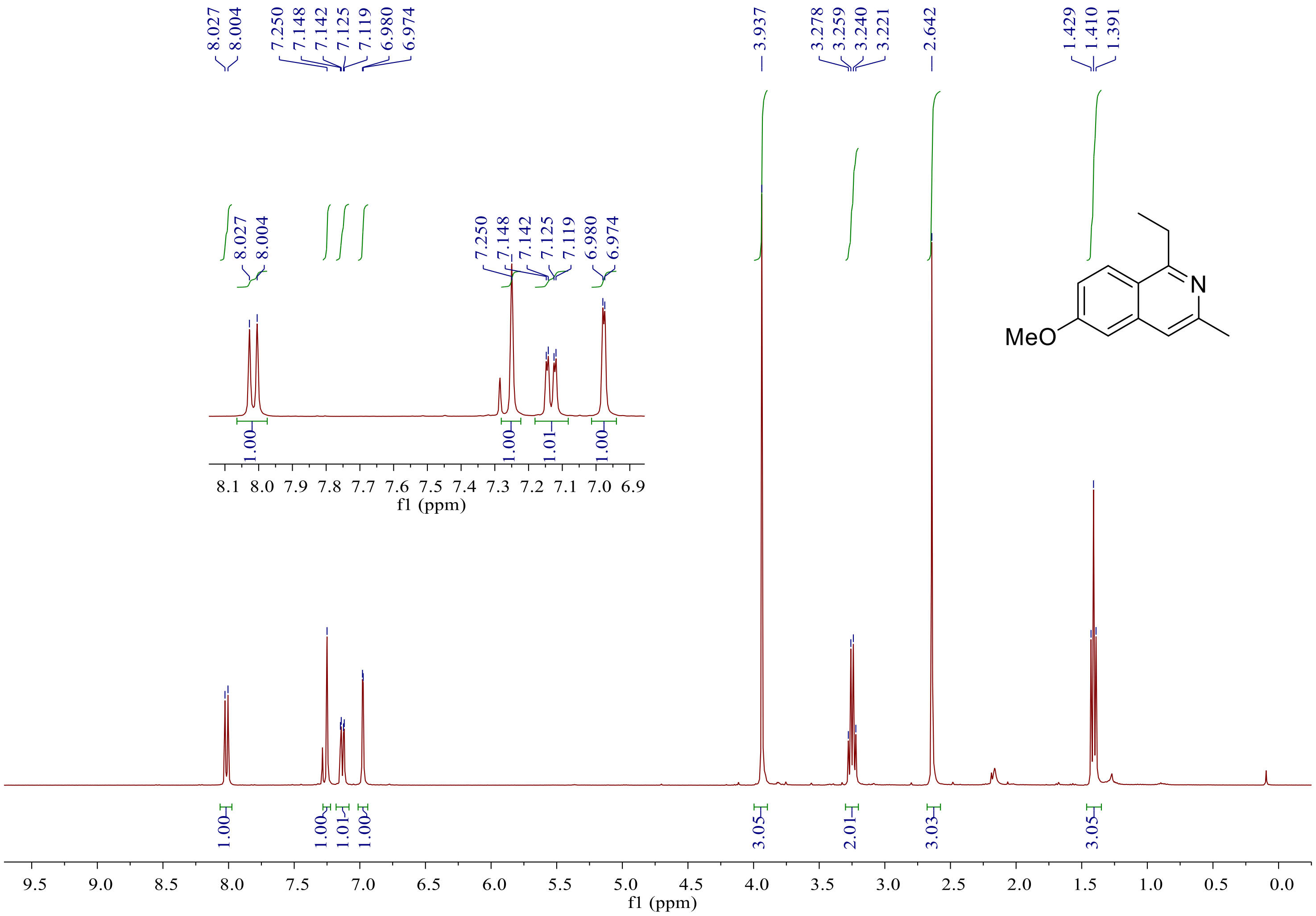

Supplementary Figure 114. ^{1}H NMR (400 MHz, $CDCl_3$) of **2s**

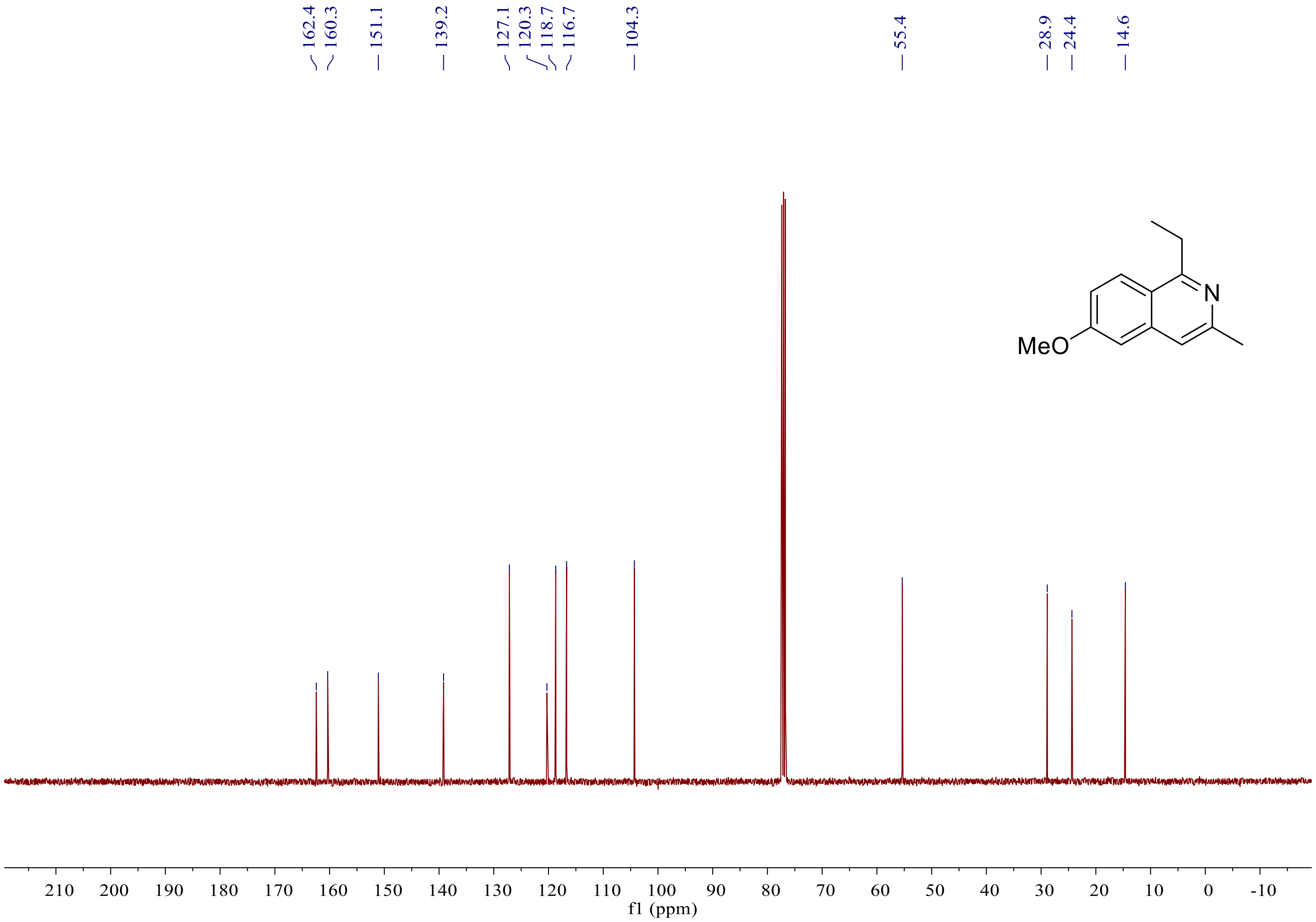

Supplementary Figure 115. ^{13}C NMR (100 MHz, $CDCl_3$) of **2s**

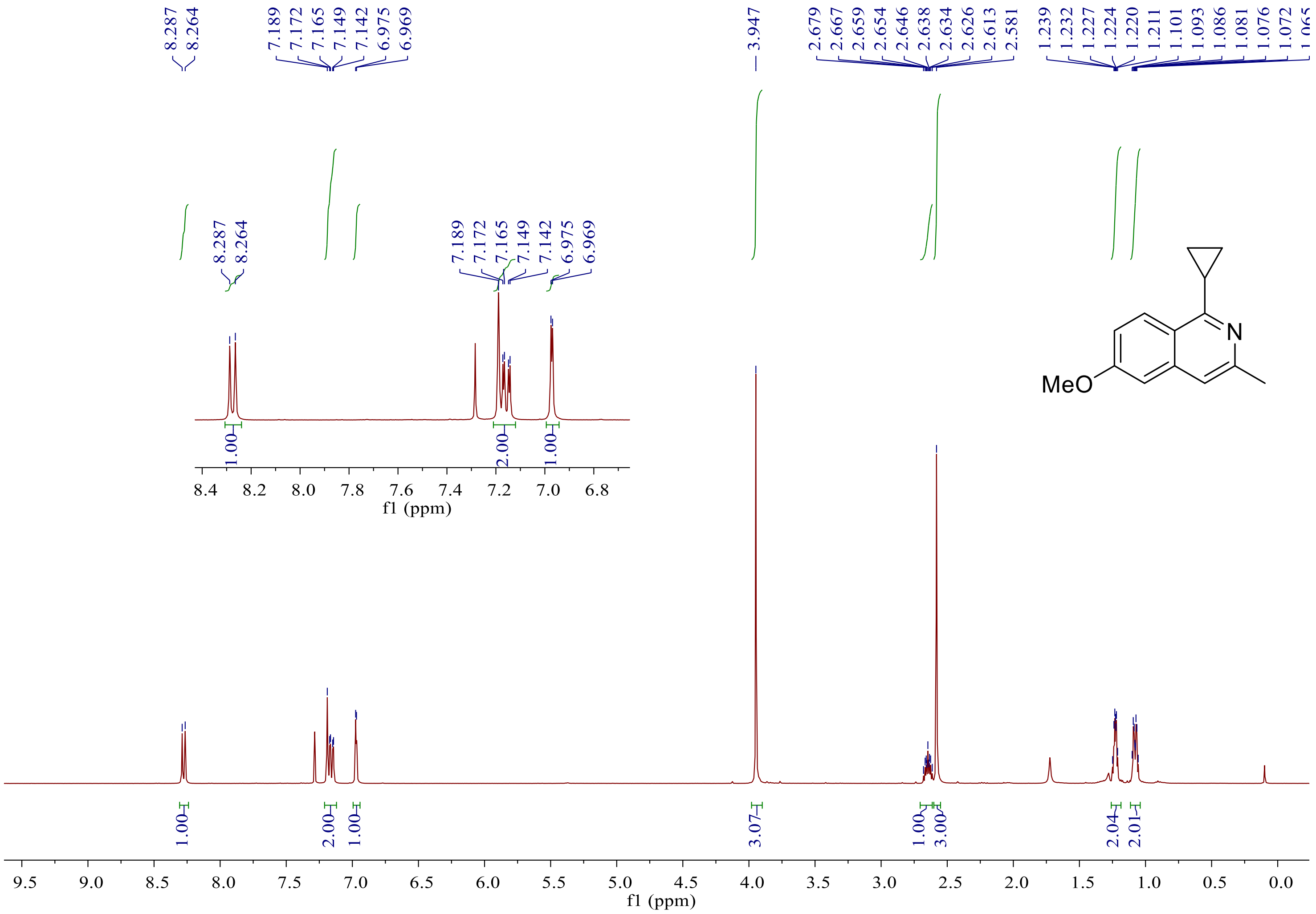

Supplementary Figure 116. ^{1}H NMR (400 MHz, $CDCl_3$) of **2t**

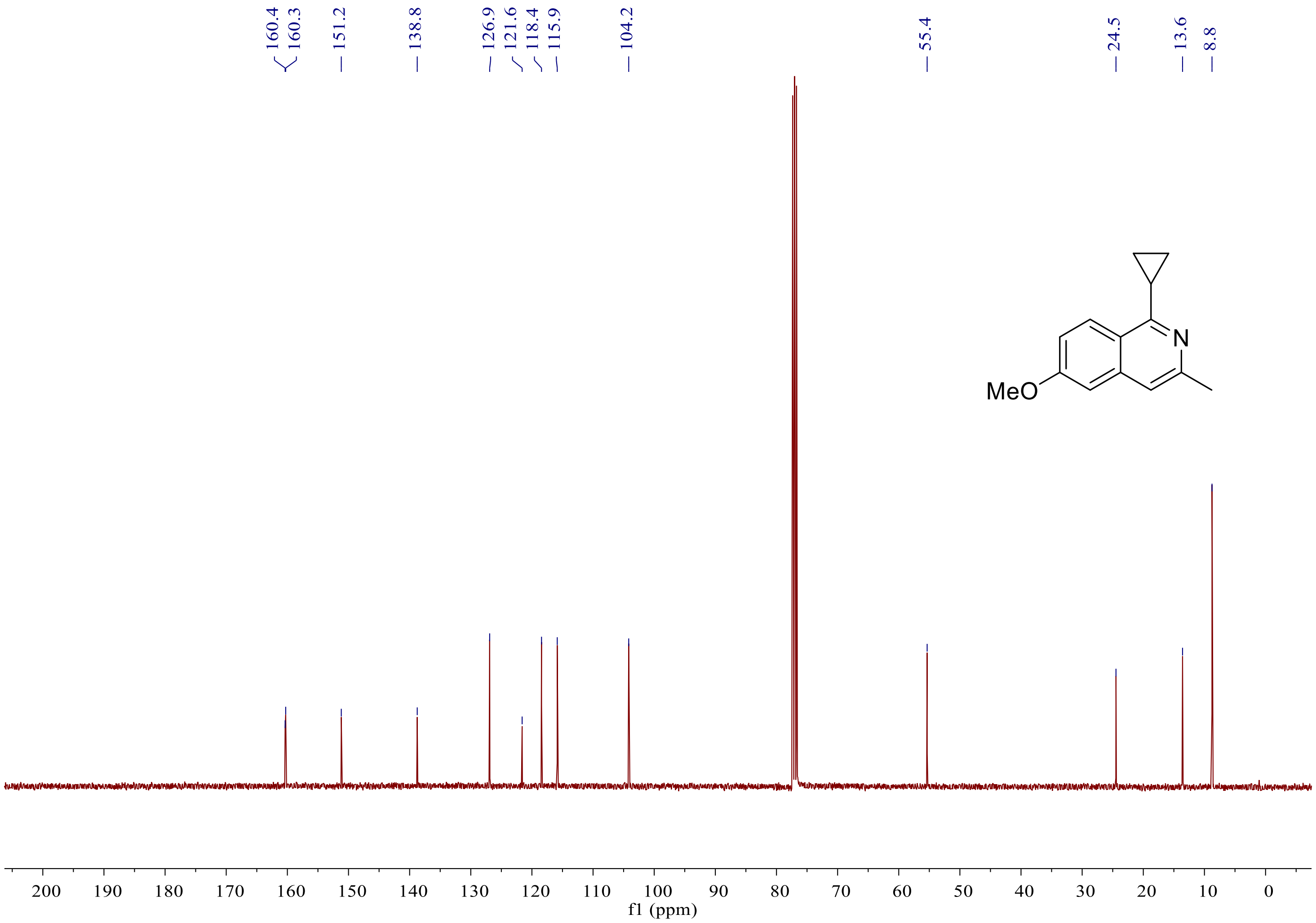

Supplementary Figure 117. ^{13}C NMR (100 MHz, $CDCl_3$) of **2t**

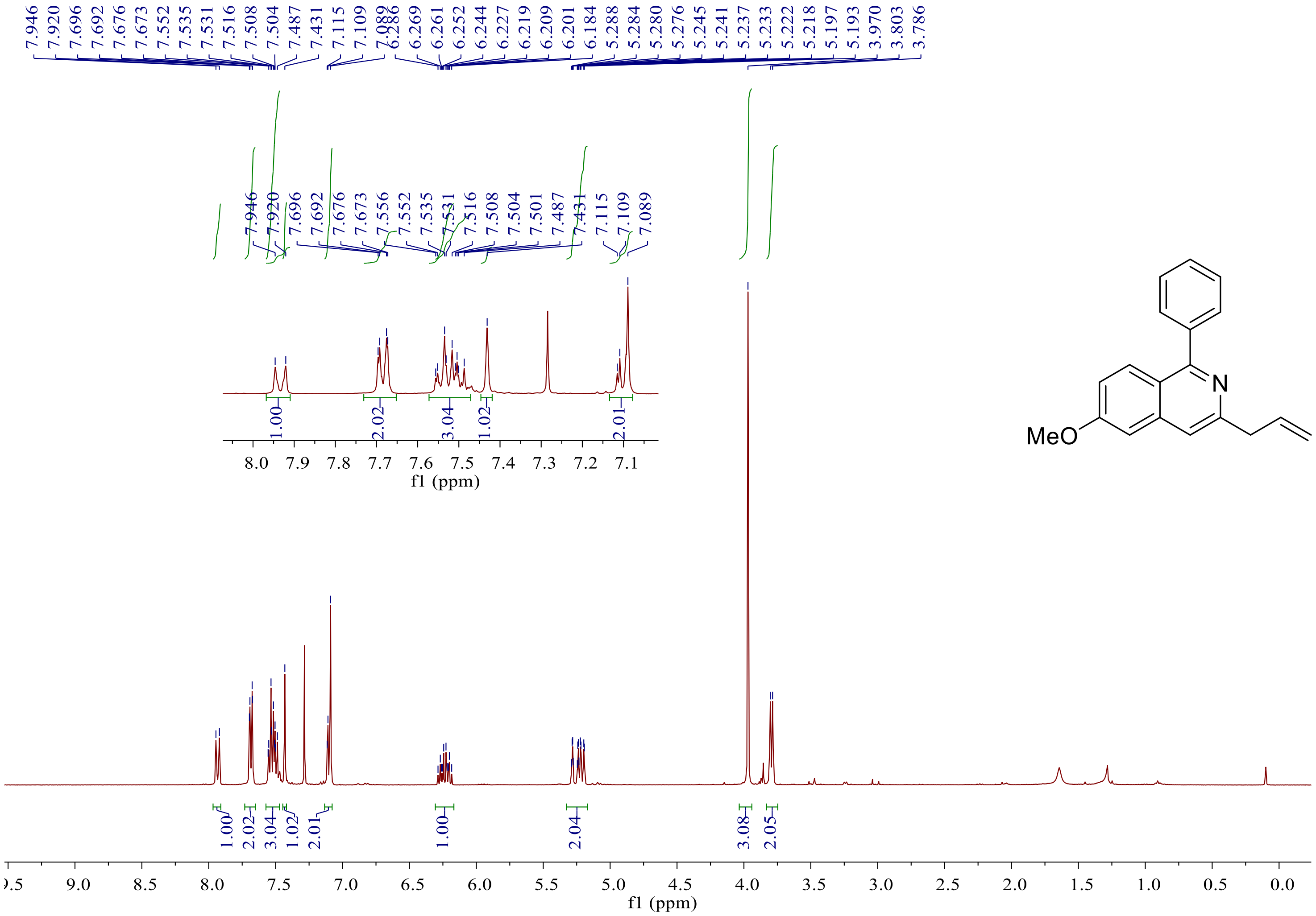

Supplementary Figure 118. ^{1}H NMR (400 MHz, $CDCl_3$) of **2u**

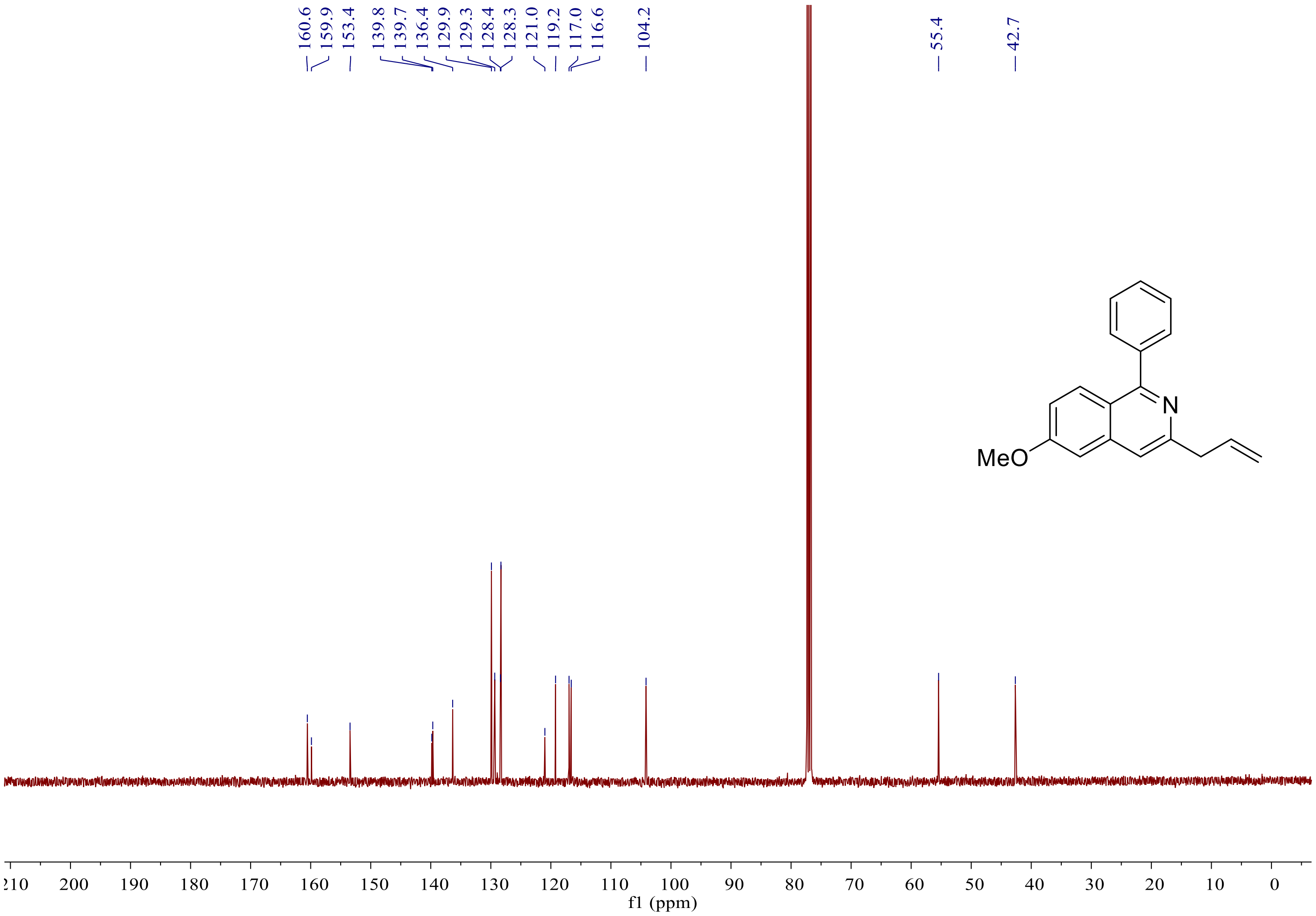

Supplementary Figure 119. ^{13}C NMR (100 MHz, $CDCl_3$) of **2u**

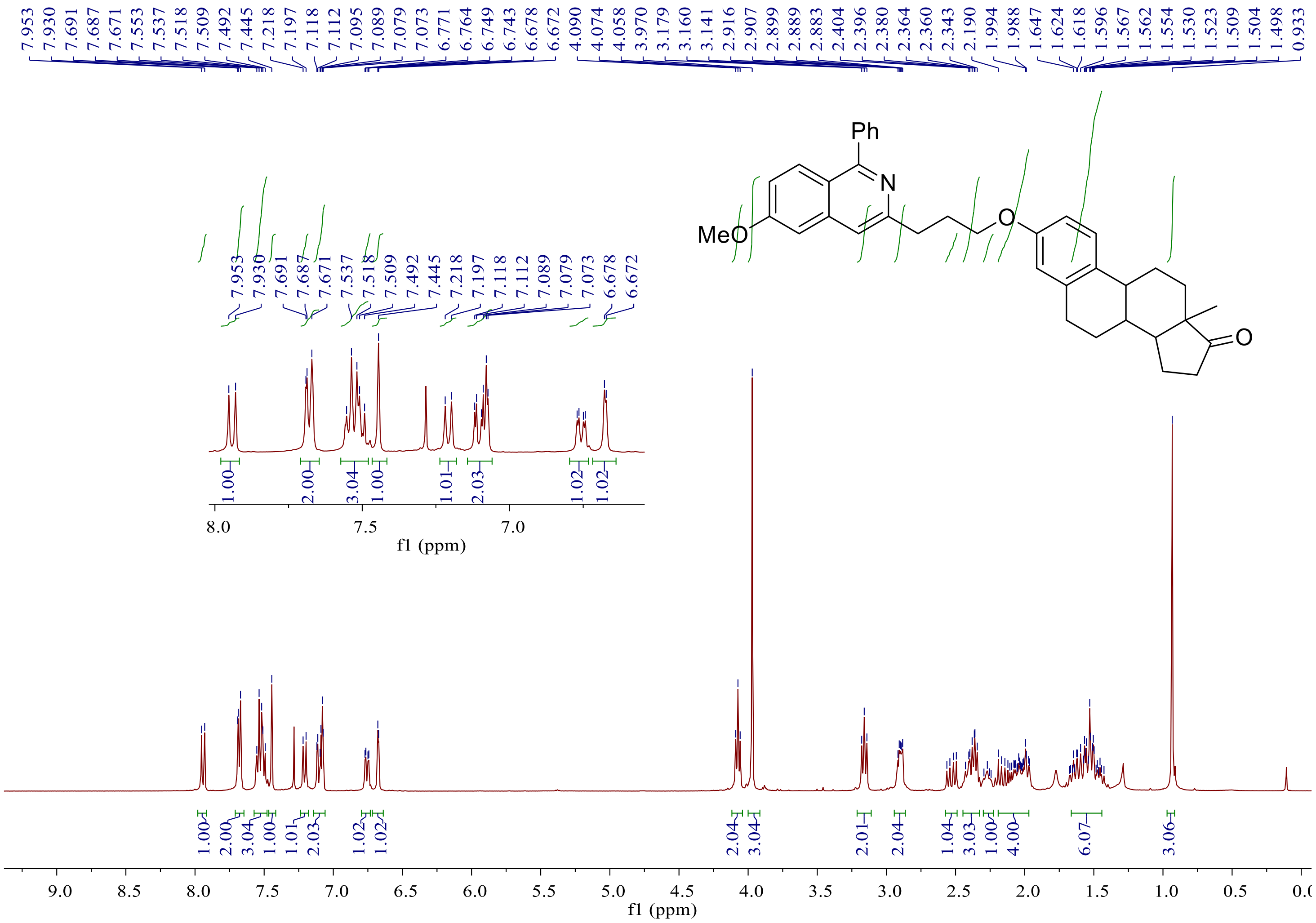

Supplementary Figure 120. ^{1}H NMR (400 MHz, $CDCl_3$) of **2v**

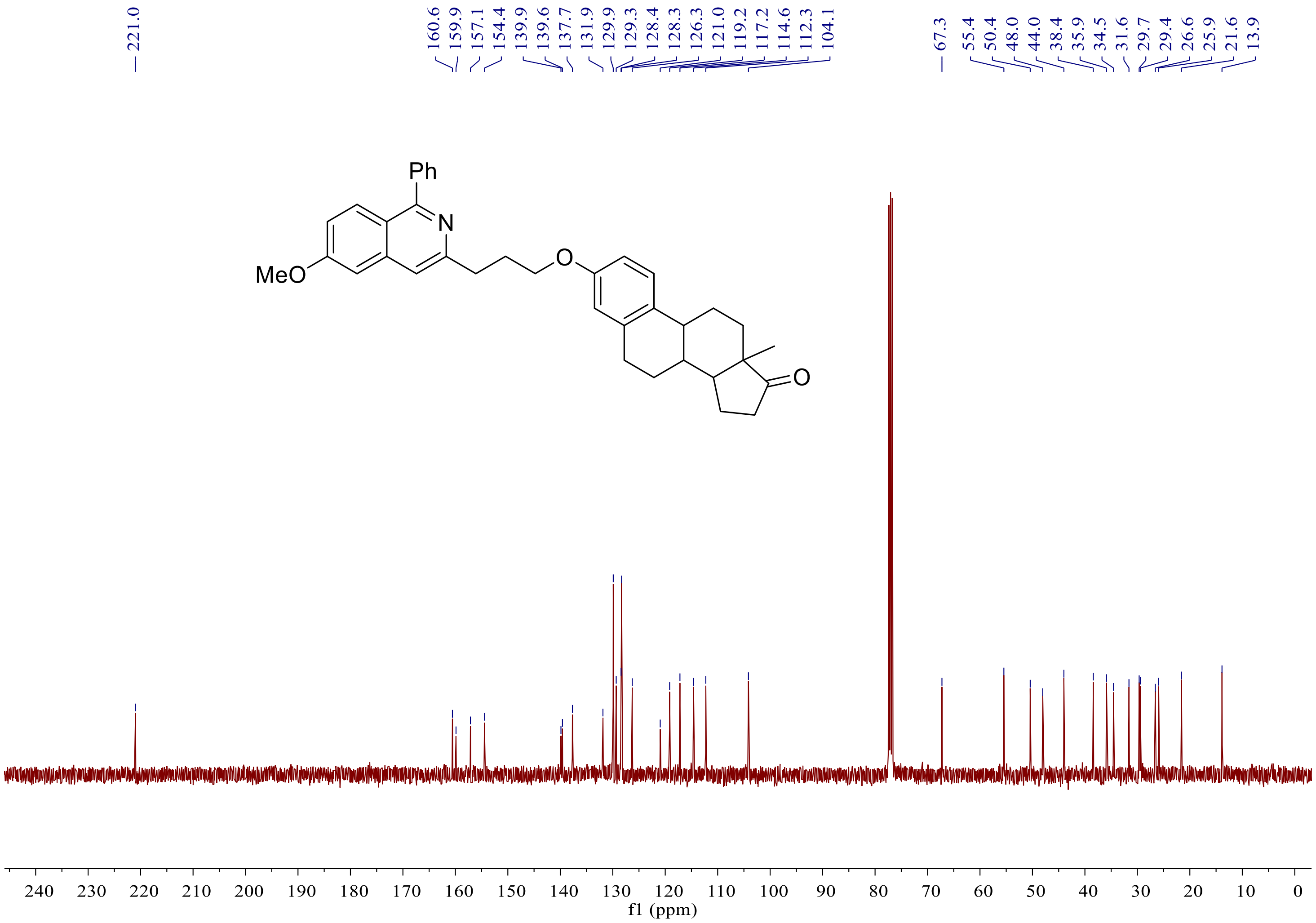

Supplementary Figure 121. ^{13}C NMR (100 MHz, $CDCl_3$) of **2v**

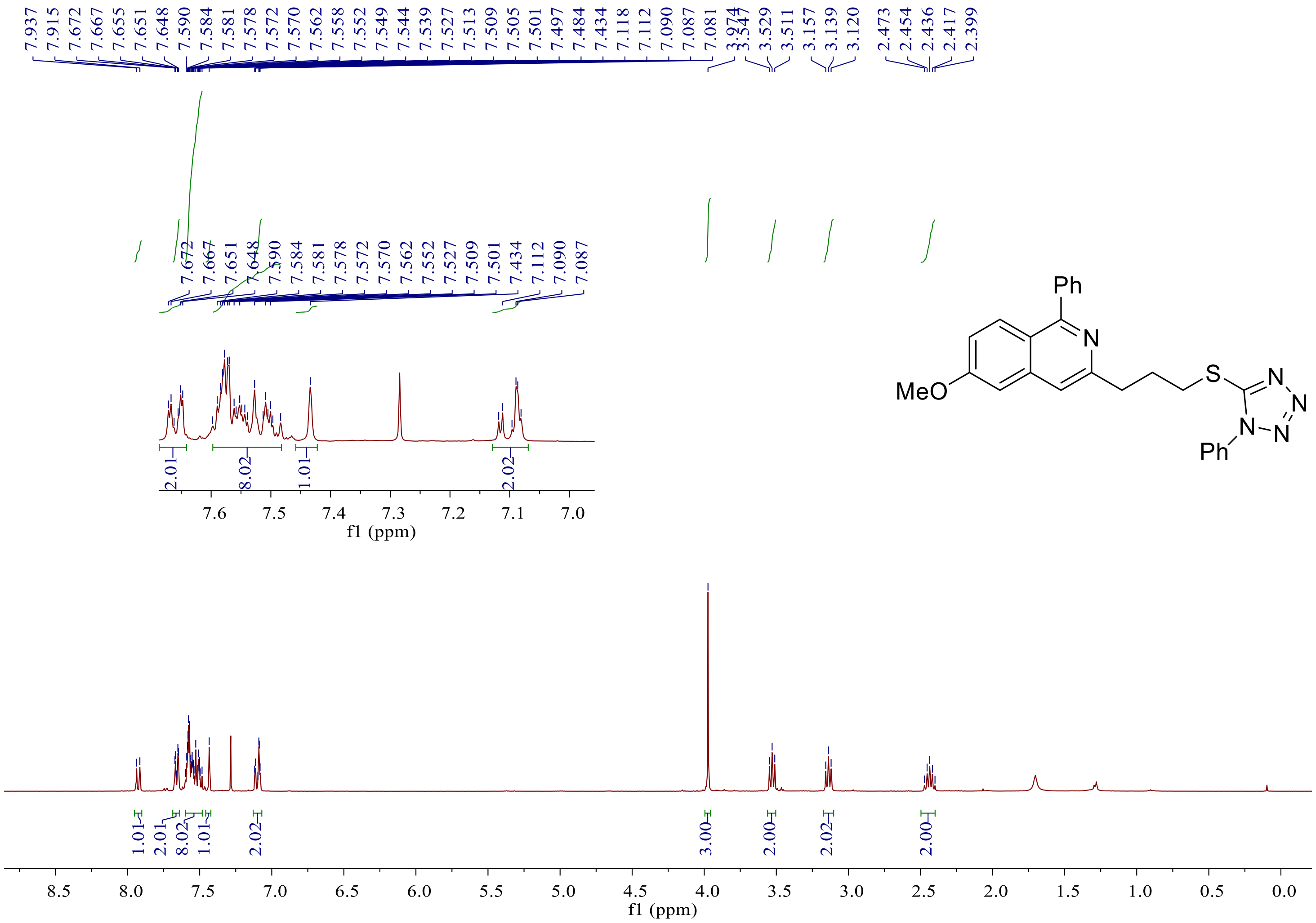

Supplementary Figure 122. ^{1}H NMR (400 MHz, $CDCl_3$) of **2w**

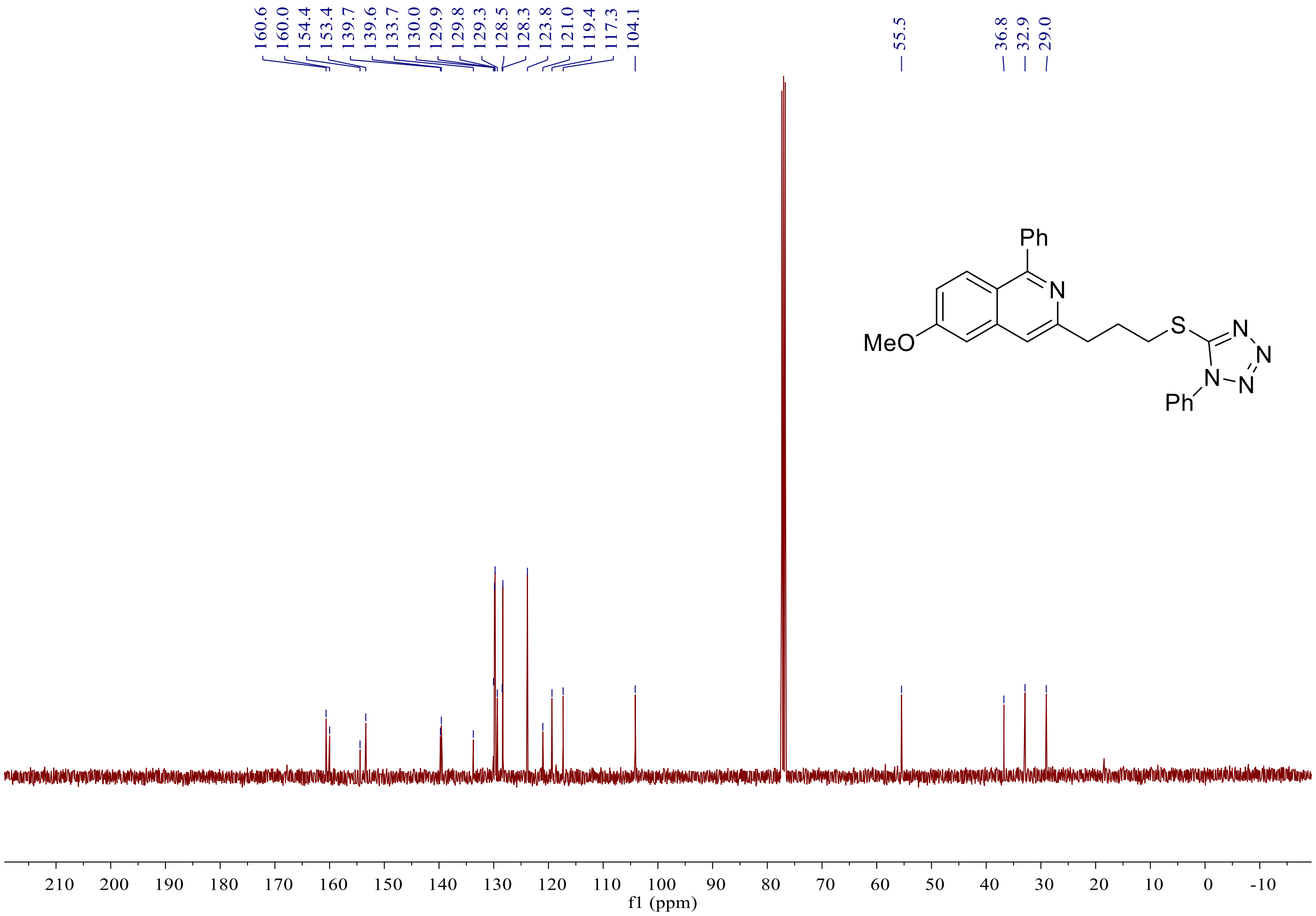

Supplementary Figure 123. ^{13}C NMR (100 MHz, $CDCl_3$) of **2w**

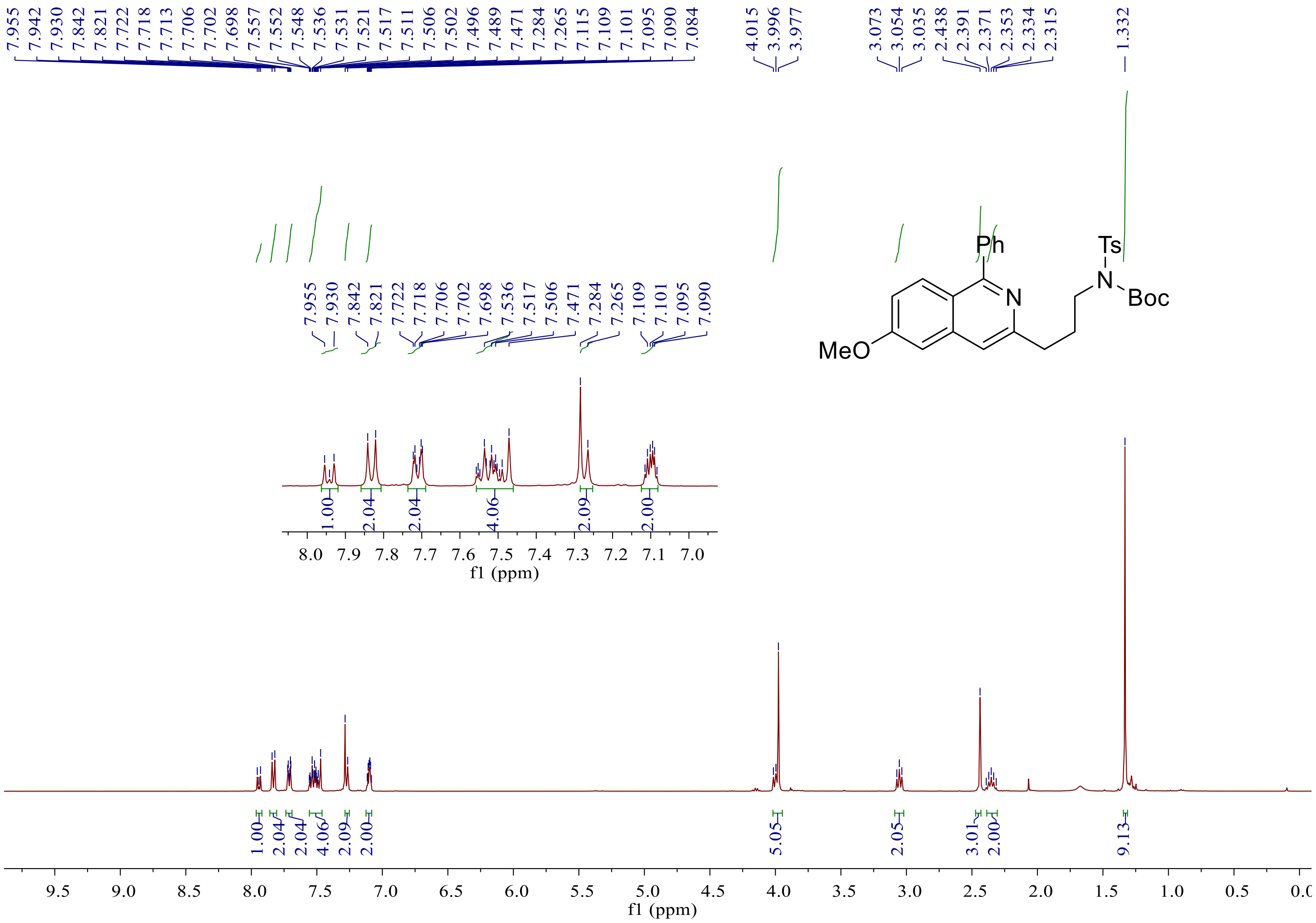

Supplementary Figure 124. ^{1}H NMR (400 MHz, $CDCl_3$) of **2x**

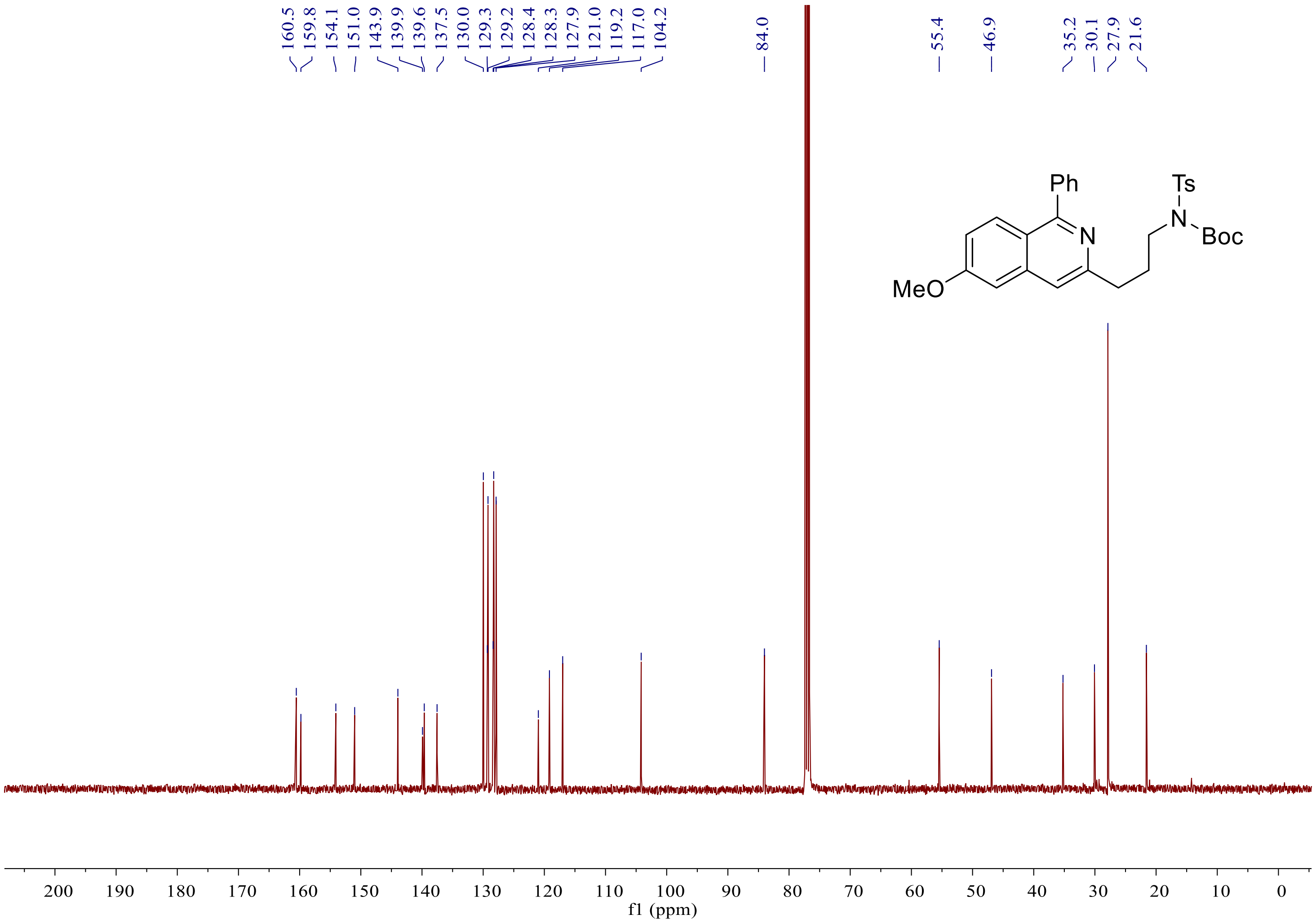

Supplementary Figure 125. ^{13}C NMR (100 MHz, $CDCl_3$) of **2x**

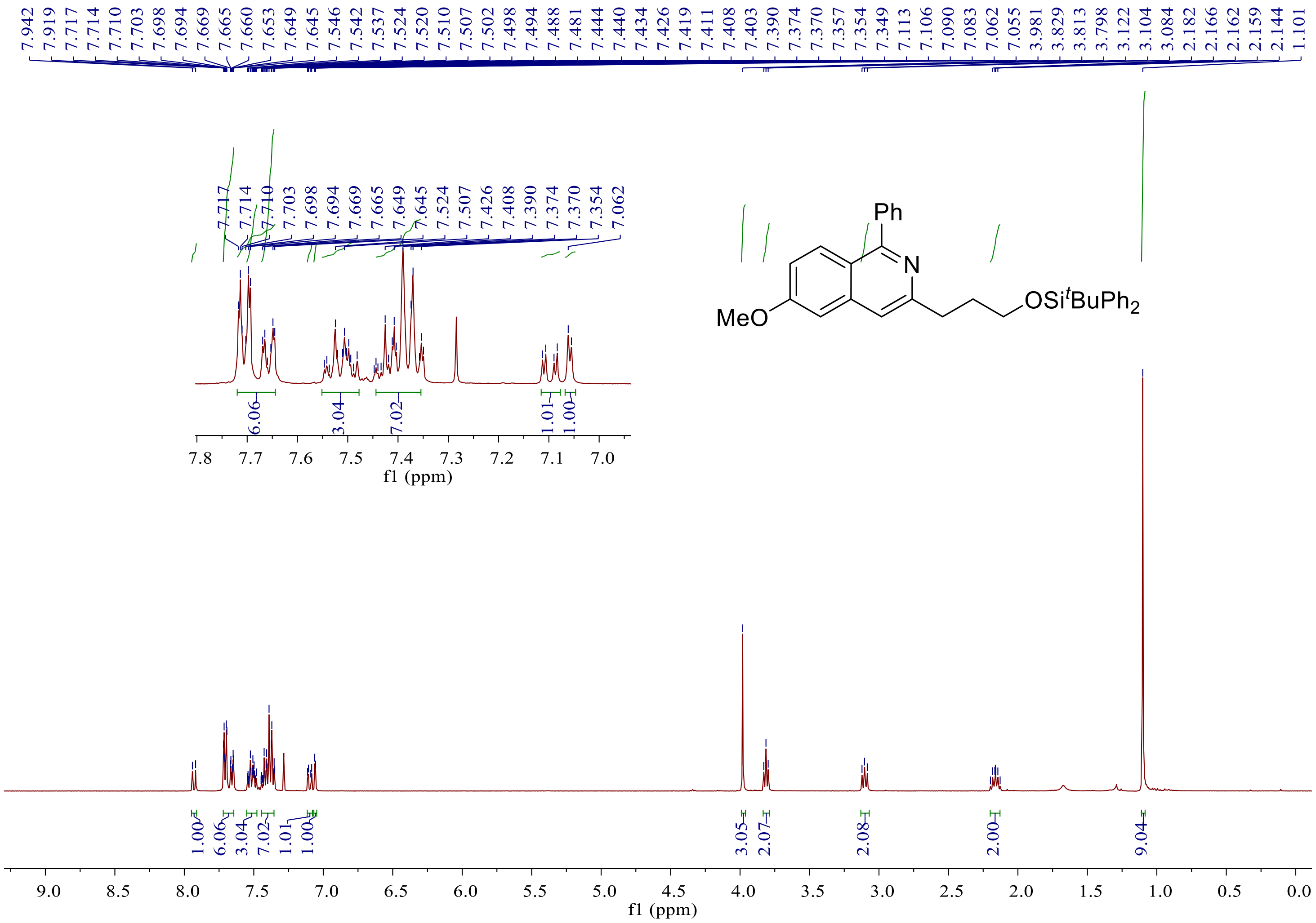

Supplementary Figure 126. ^{1}H NMR (400 MHz, $CDCl_3$) of **2y**

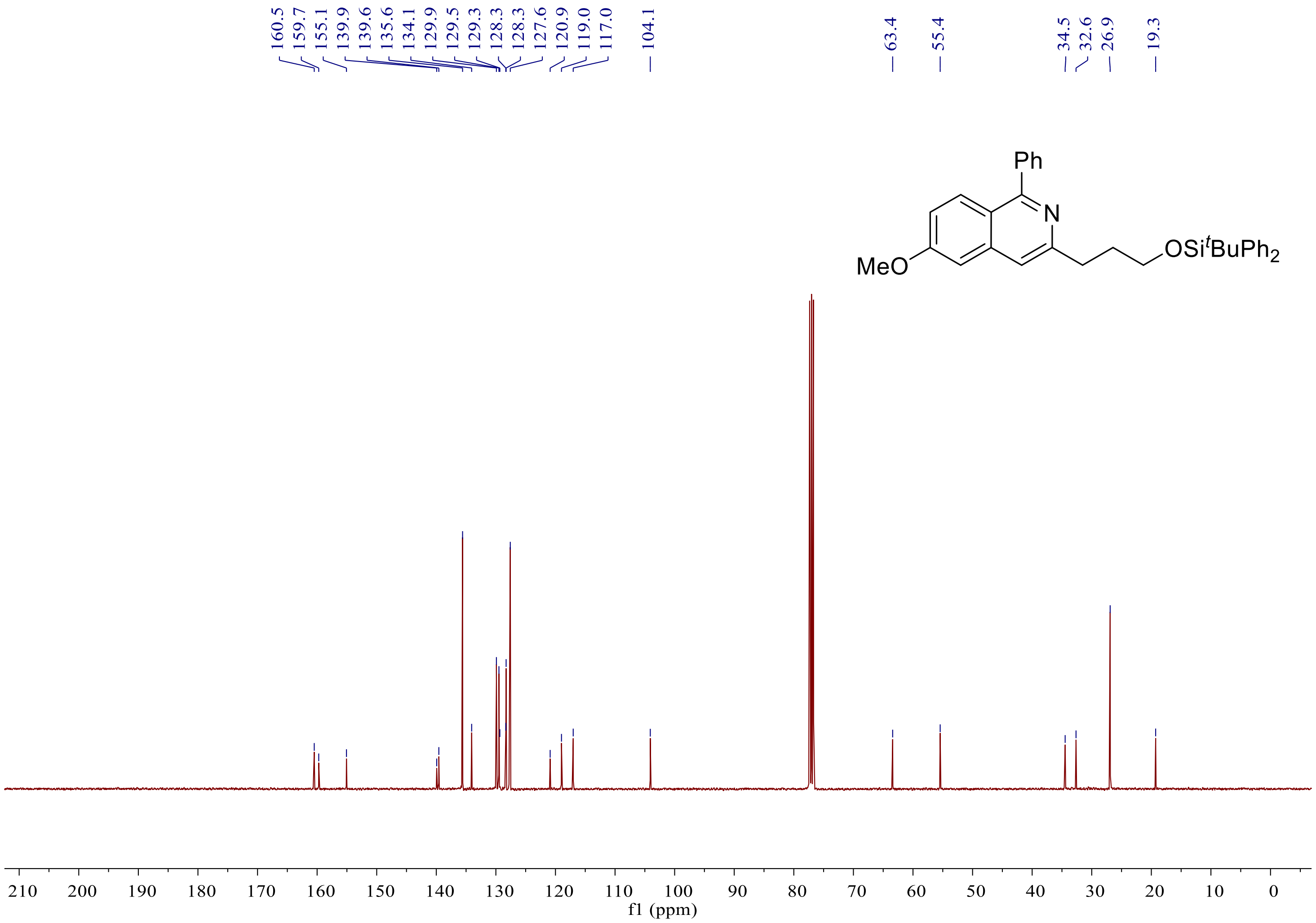

Supplementary Figure 127. ^{13}C NMR (100 MHz, $CDCl_3$) of **2y**

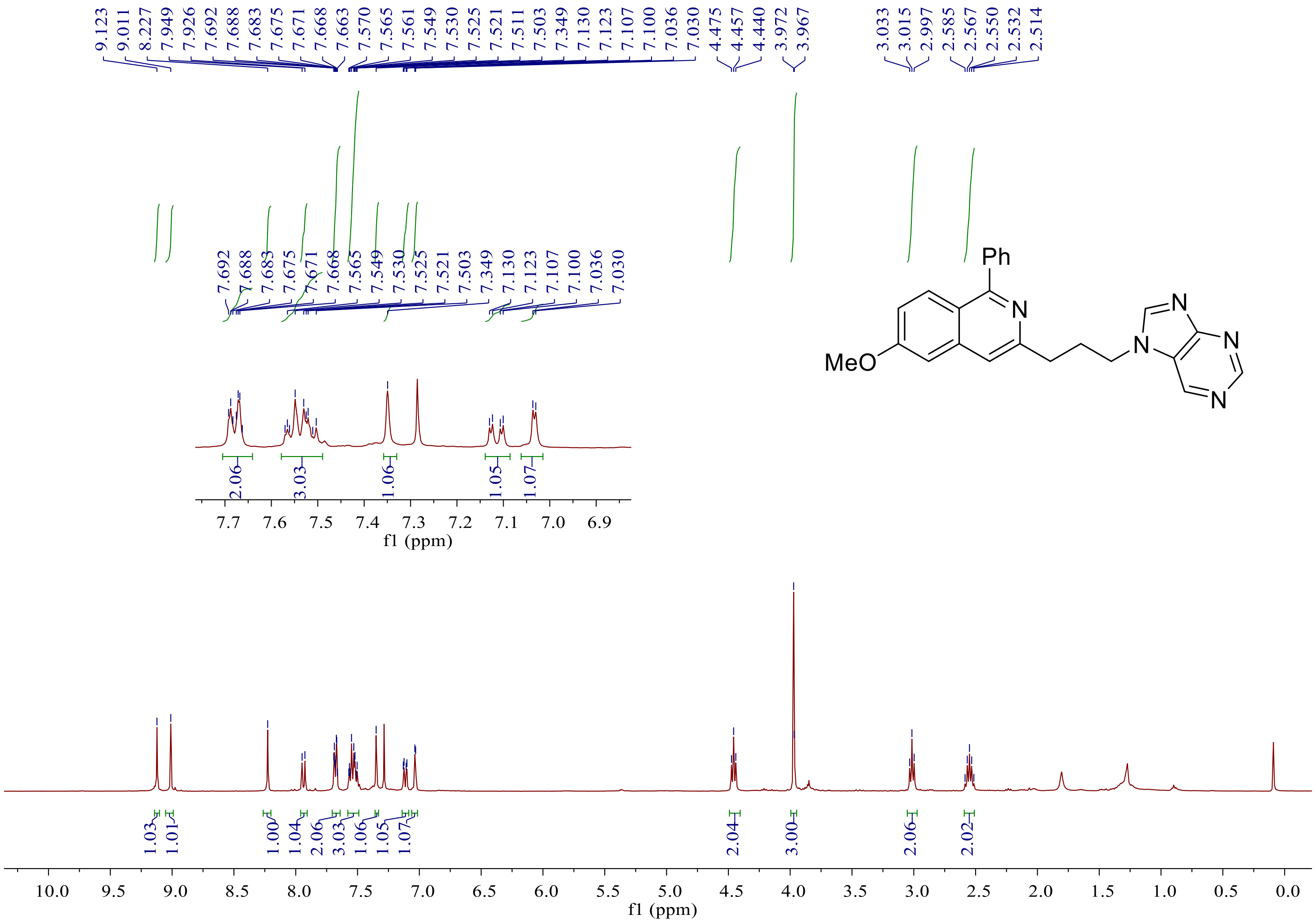

Supplementary Figure 128. ^{1}H NMR (400 MHz, $CDCl_3$) of **2z**

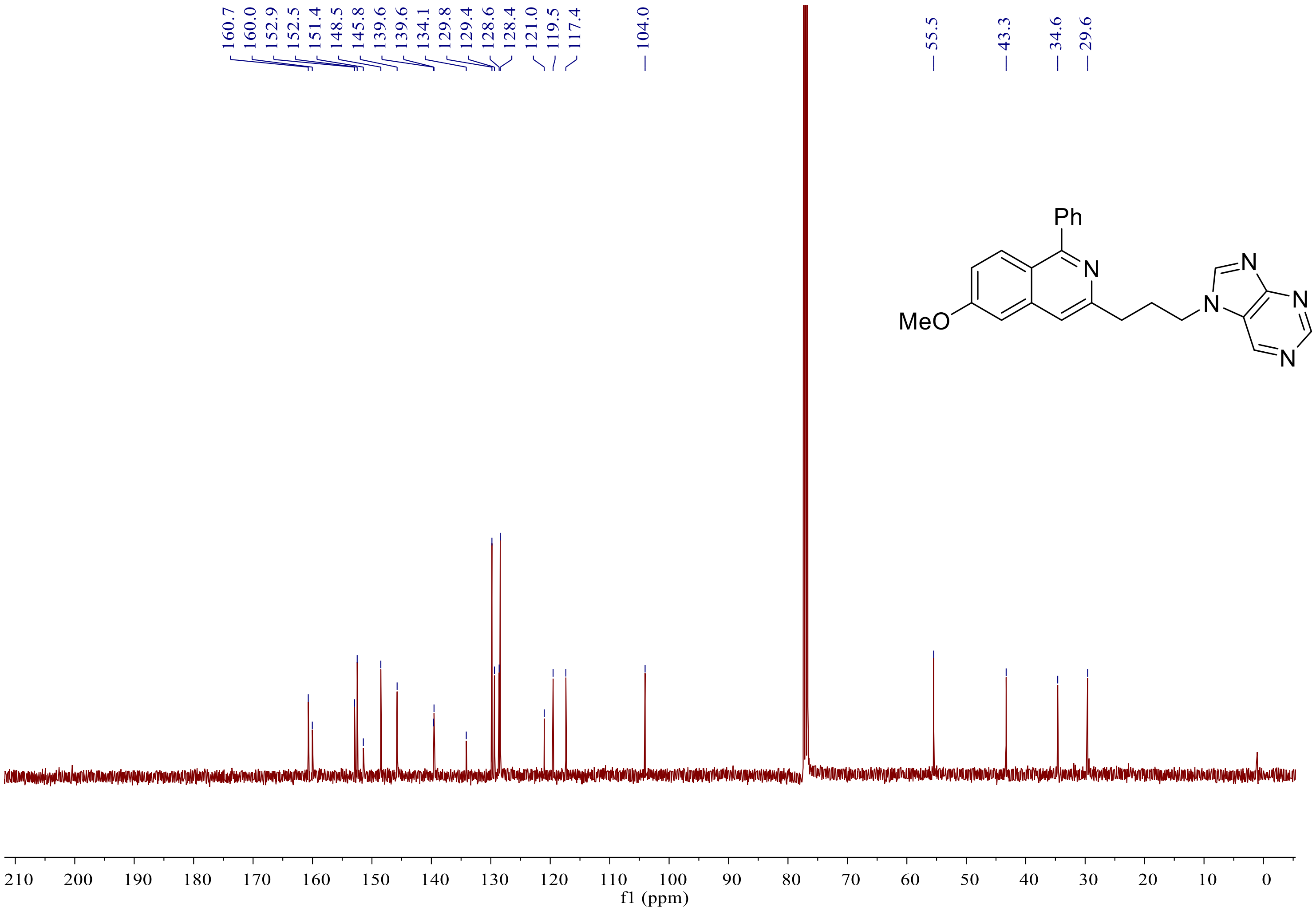

Supplementary Figure 129. ^{13}C NMR (100 MHz, $CDCl_3$) of **2z**

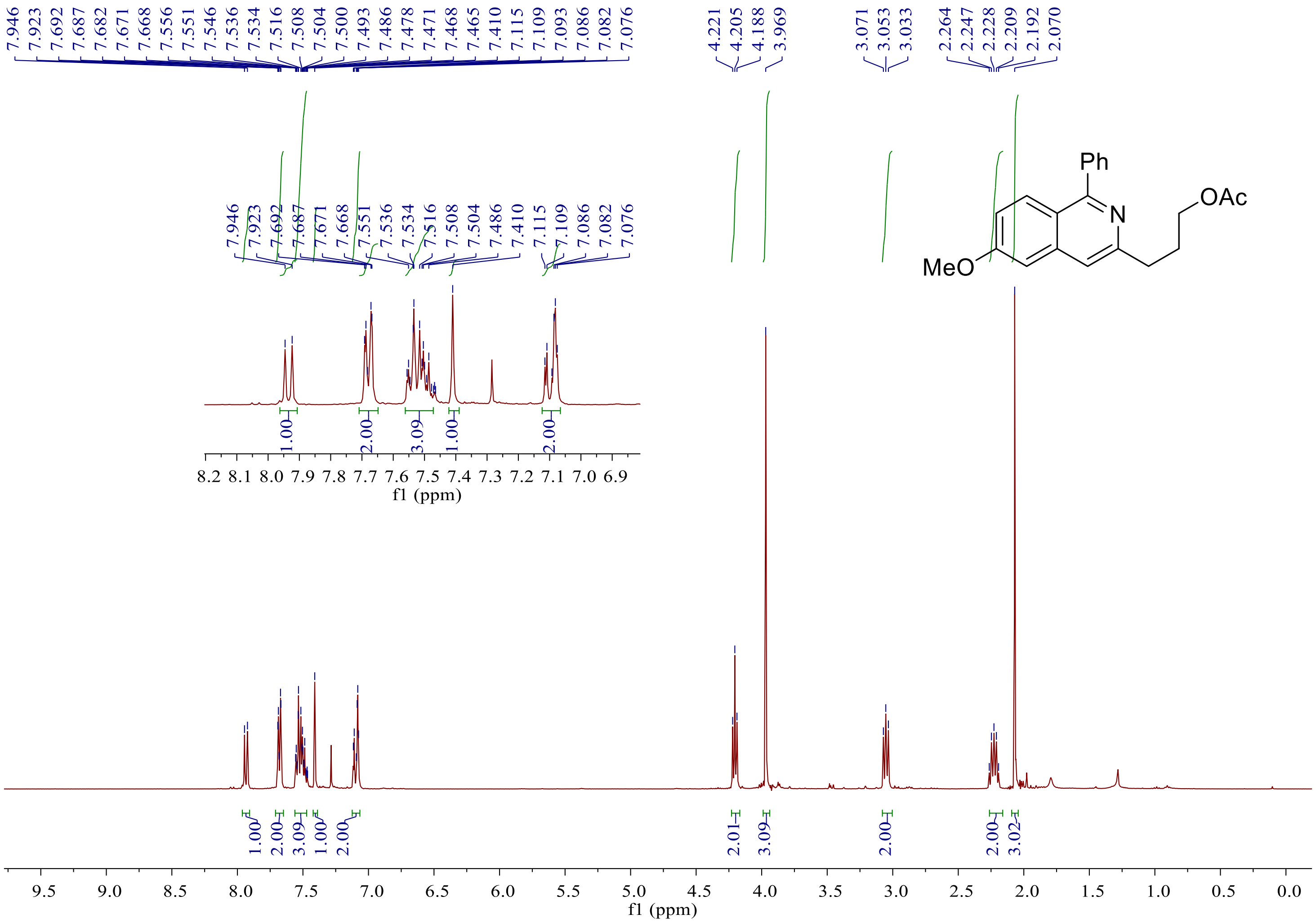

Supplementary Figure 130. ^{1}H NMR (400 MHz, $CDCl_3$) of **2aa**

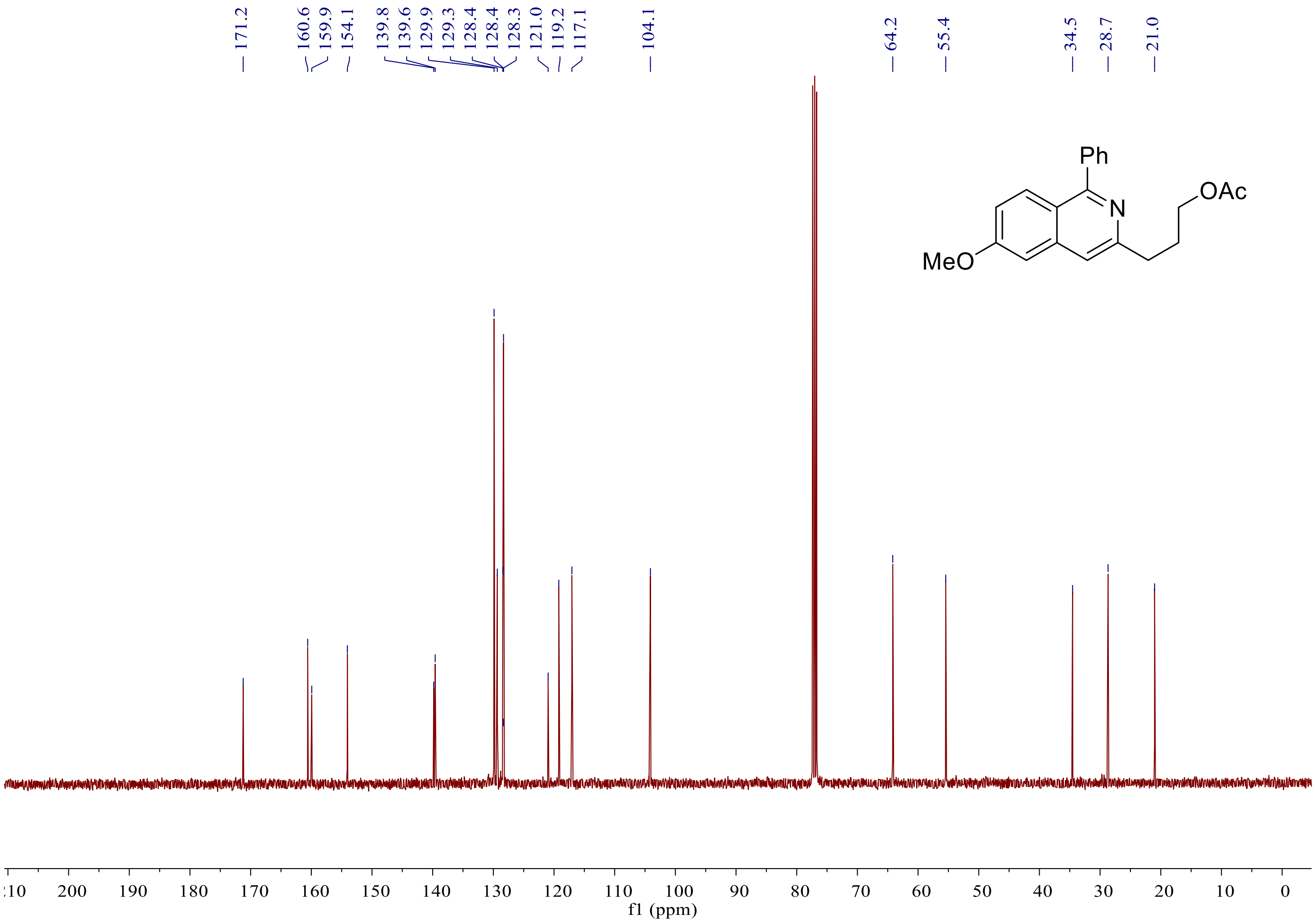

Supplementary Figure 131. ^{13}C NMR (100 MHz, $CDCl_3$) of **2aa**

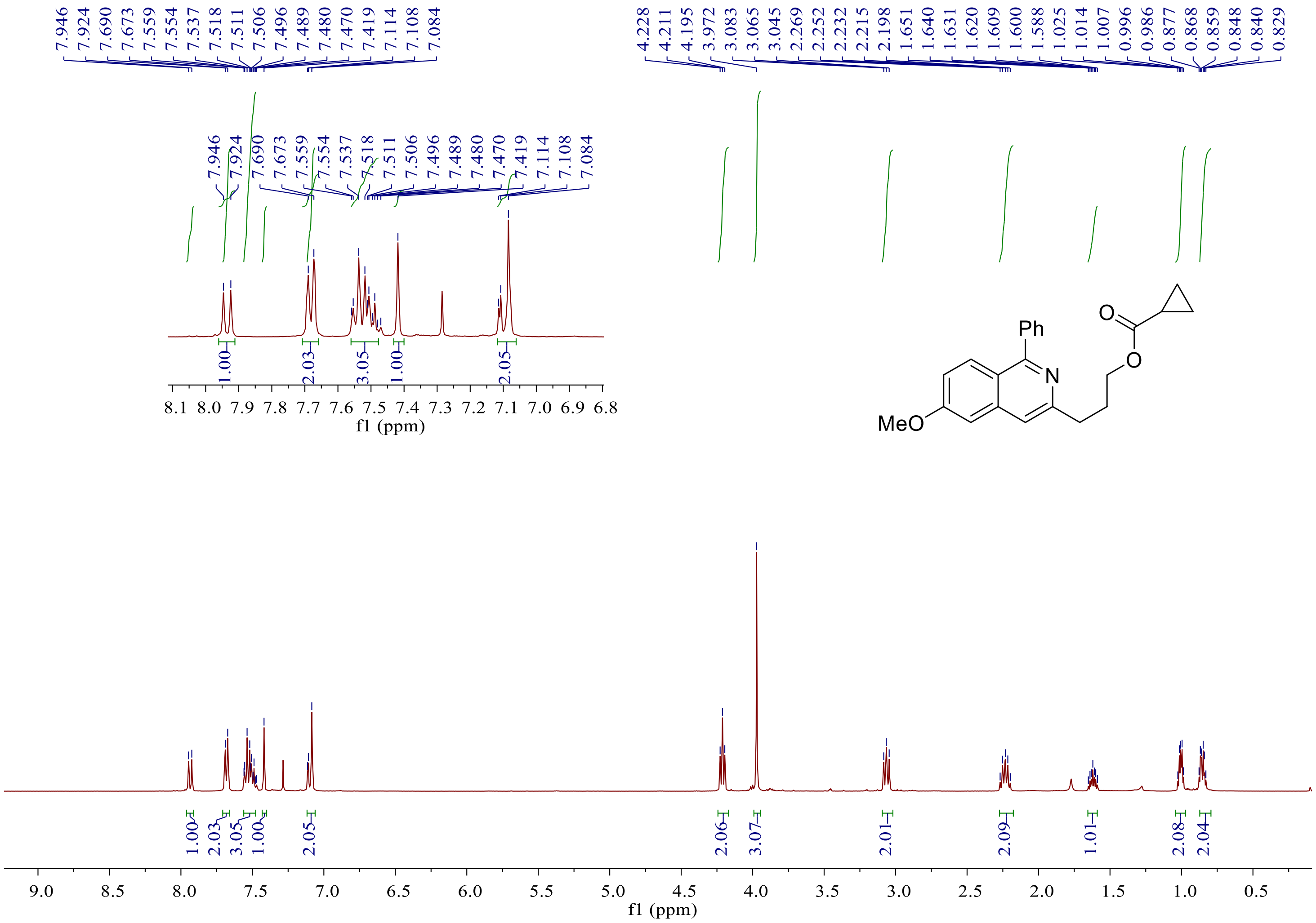

Supplementary Figure 132. ^{1}H NMR (400 MHz, $CDCl_3$) of **2ab**

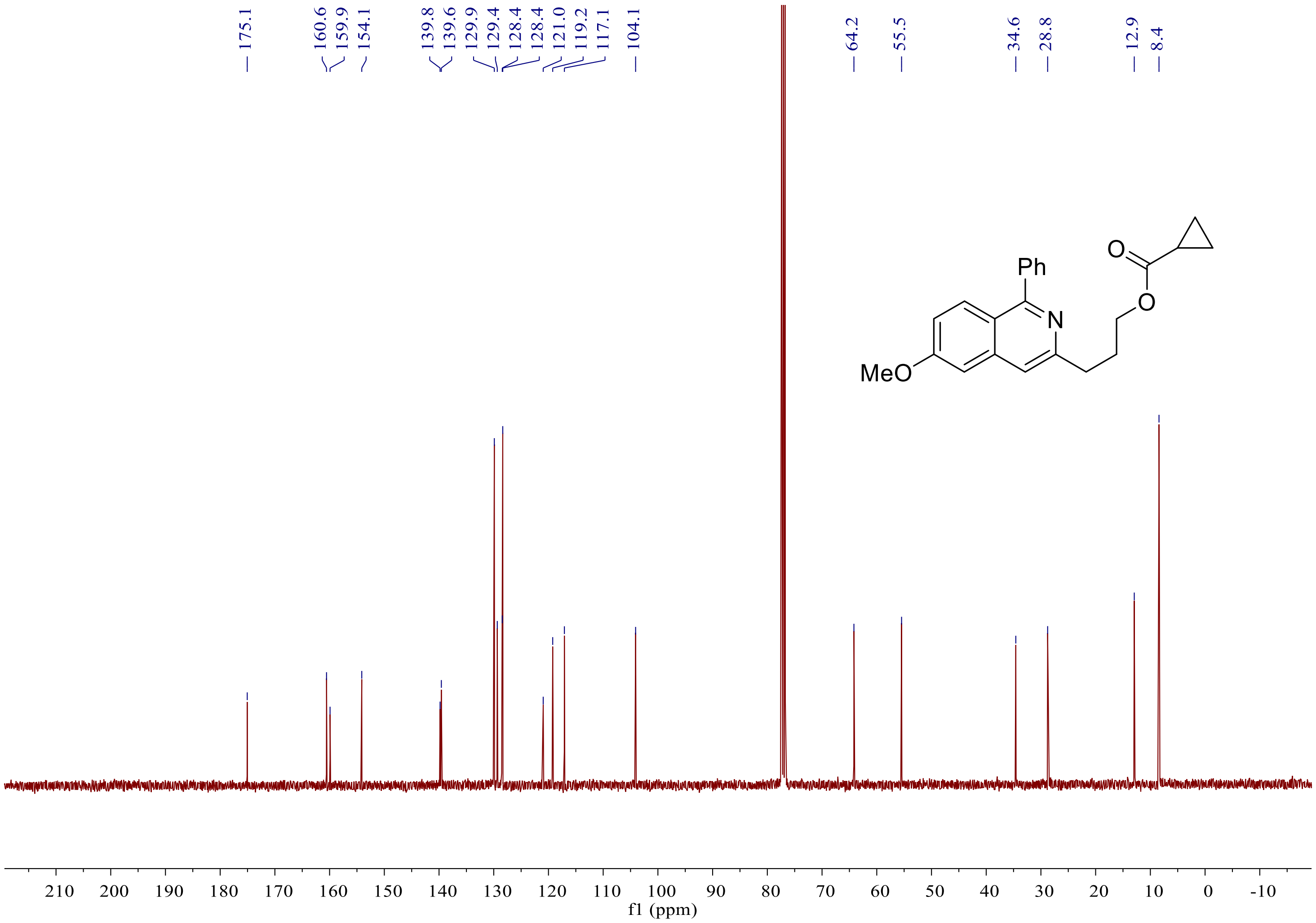

Supplementary Figure 133. ^{13}C NMR (100 MHz, $CDCl_3$) of **2ab**

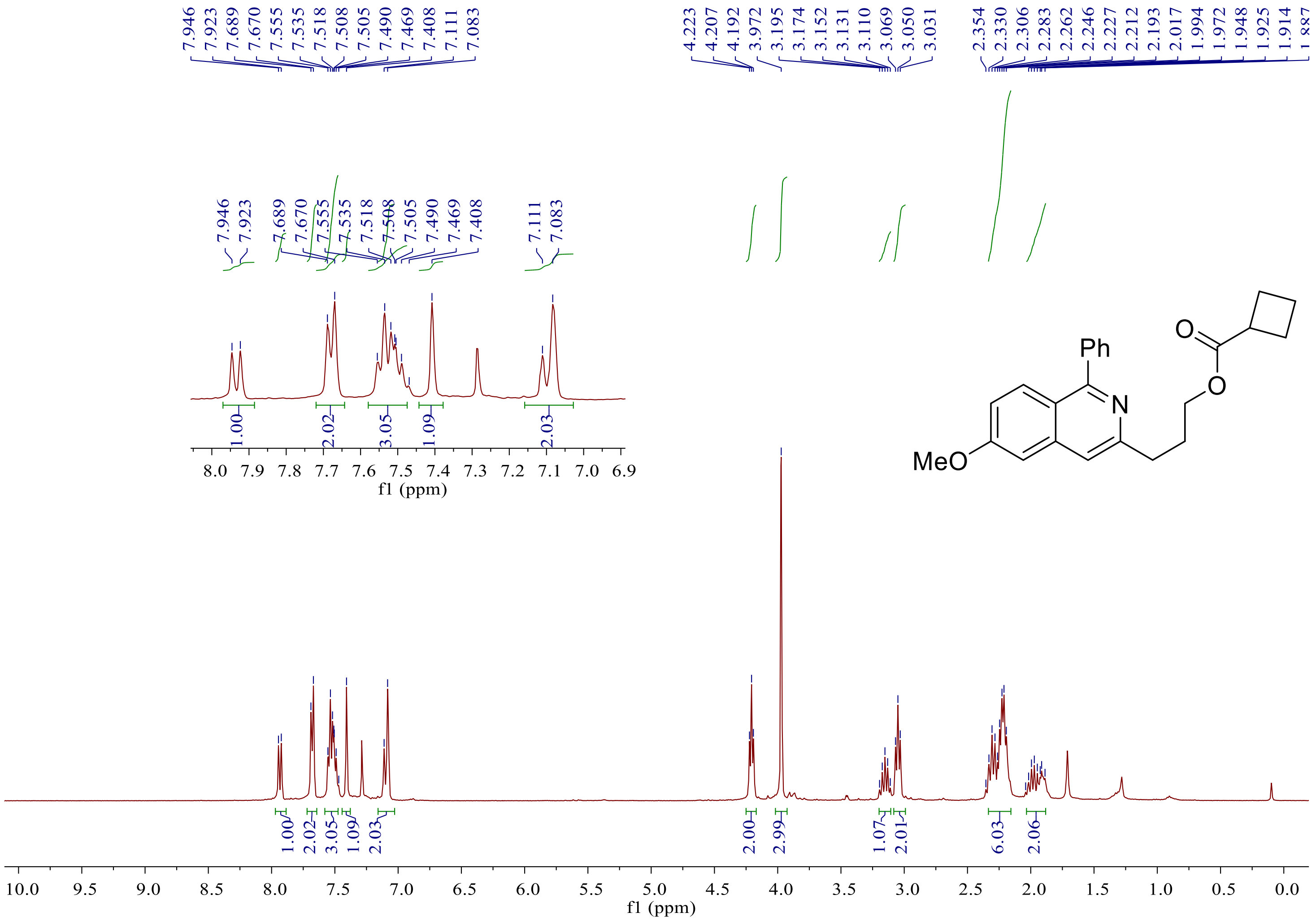

Supplementary Figure 134. ^{1}H NMR (400 MHz, $CDCl_3$) of **2ac**

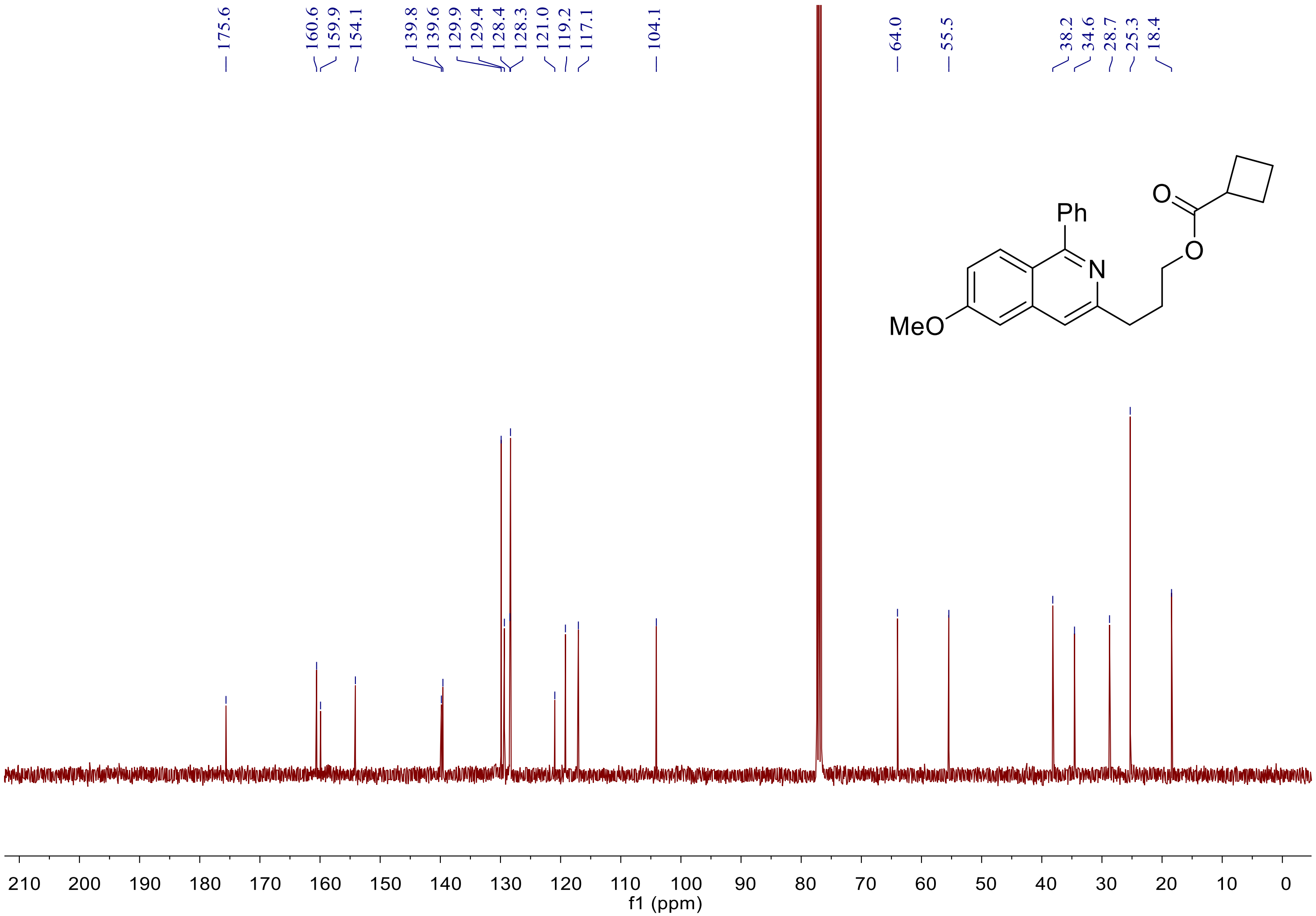

Supplementary Figure 135. ^{13}C NMR (100 MHz, $CDCl_3$) of **2ac**

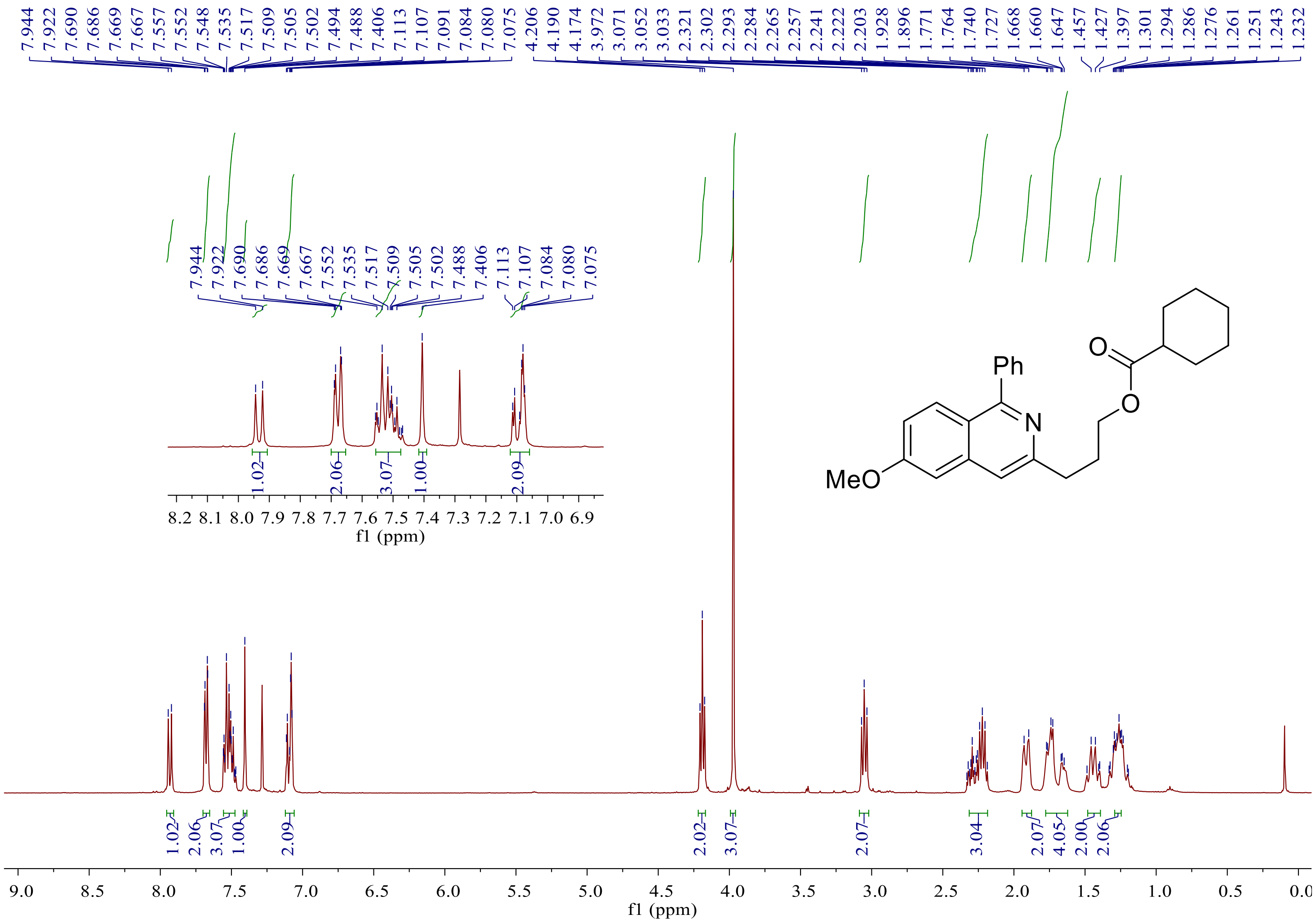

Supplementary Figure 136. ^{1}H NMR (400 MHz, $CDCl_3$) of **2ad**

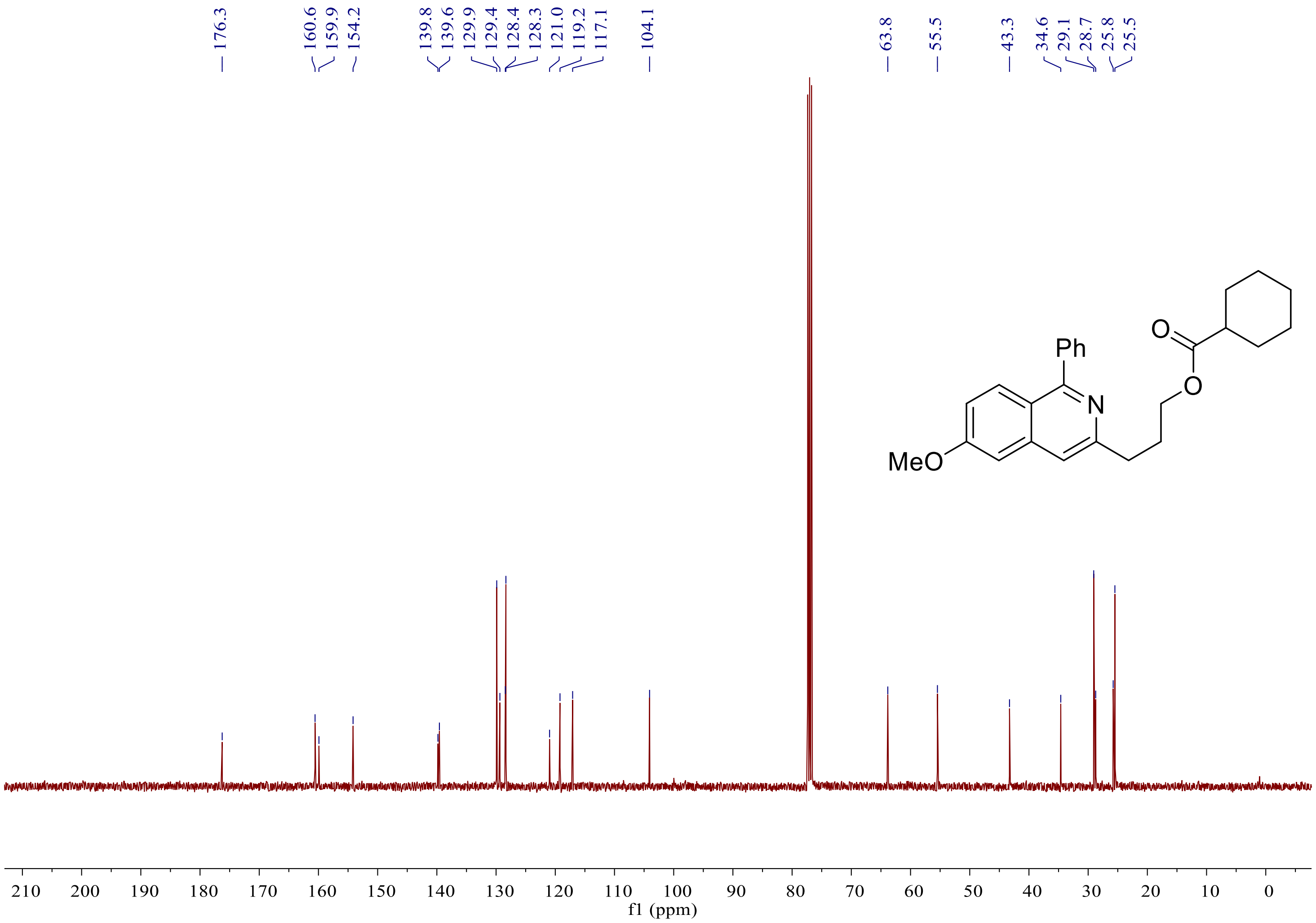

Supplementary Figure 137. ^{13}C NMR (100 MHz, $CDCl_3$) of **2ad**

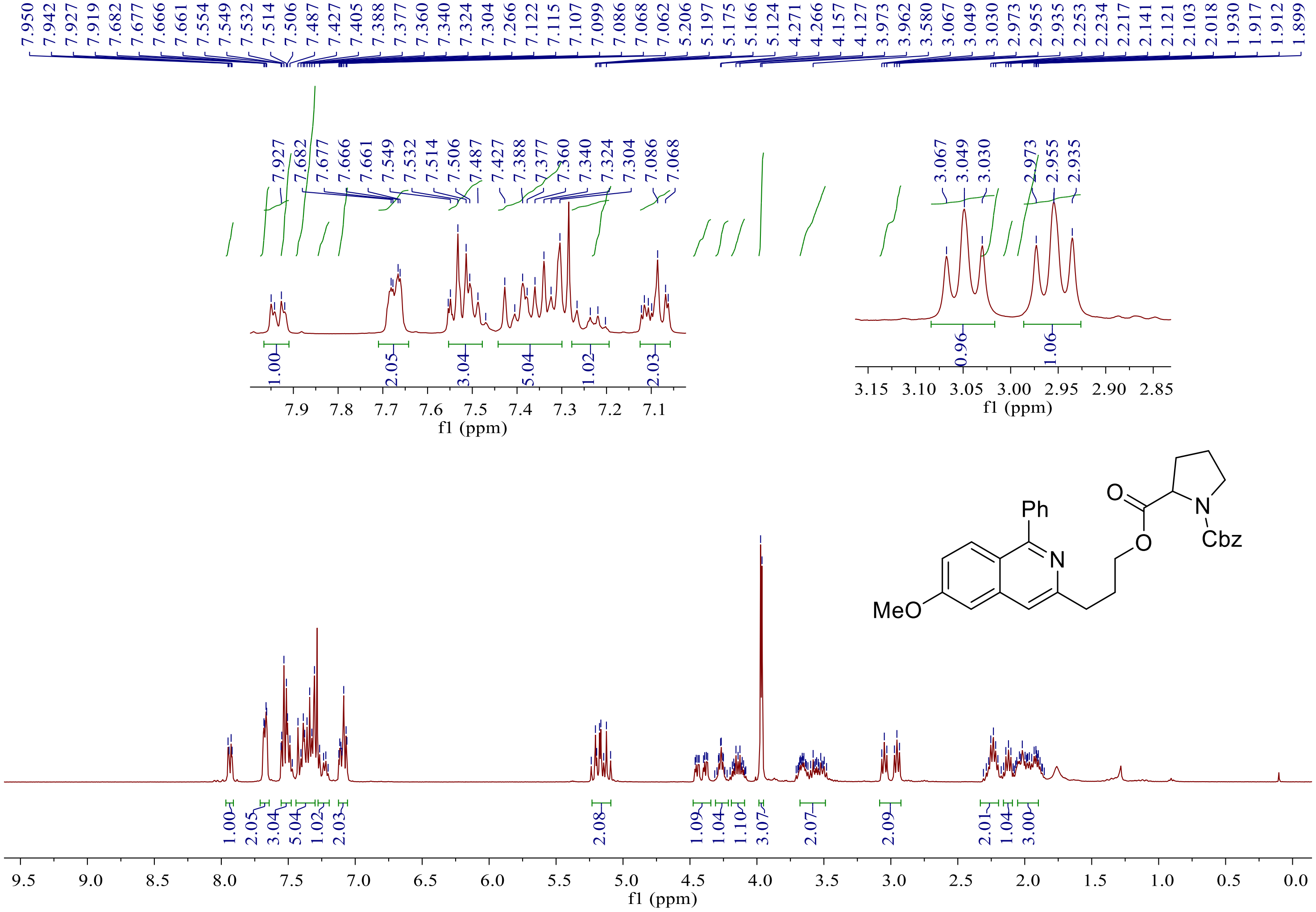

Supplementary Figure 138. ^{1}H NMR (400 MHz, $CDCl_3$) of **2ae**

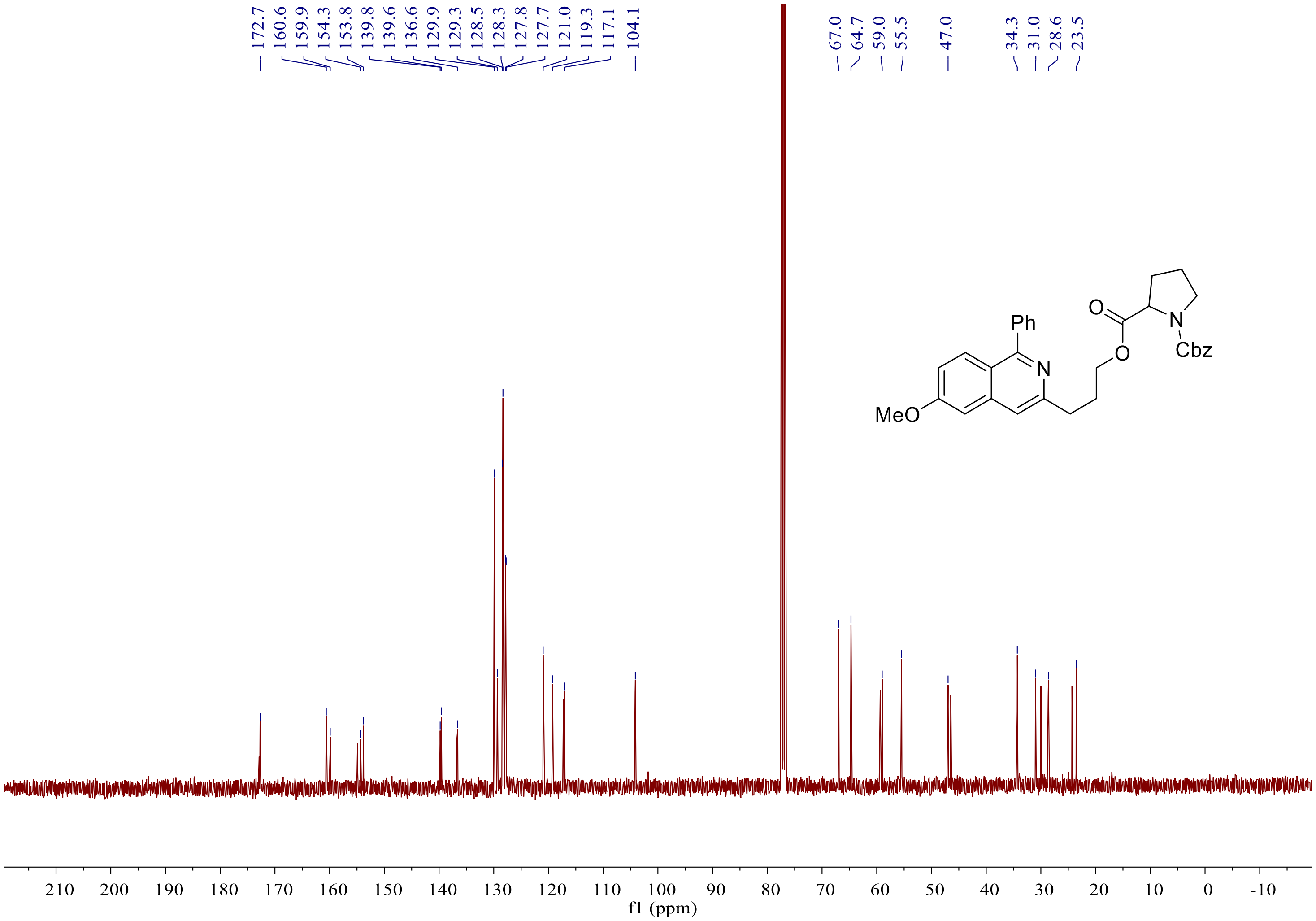

Supplementary Figure 139. ^{13}C NMR (100 MHz, $CDCl_3$) of **2ae**

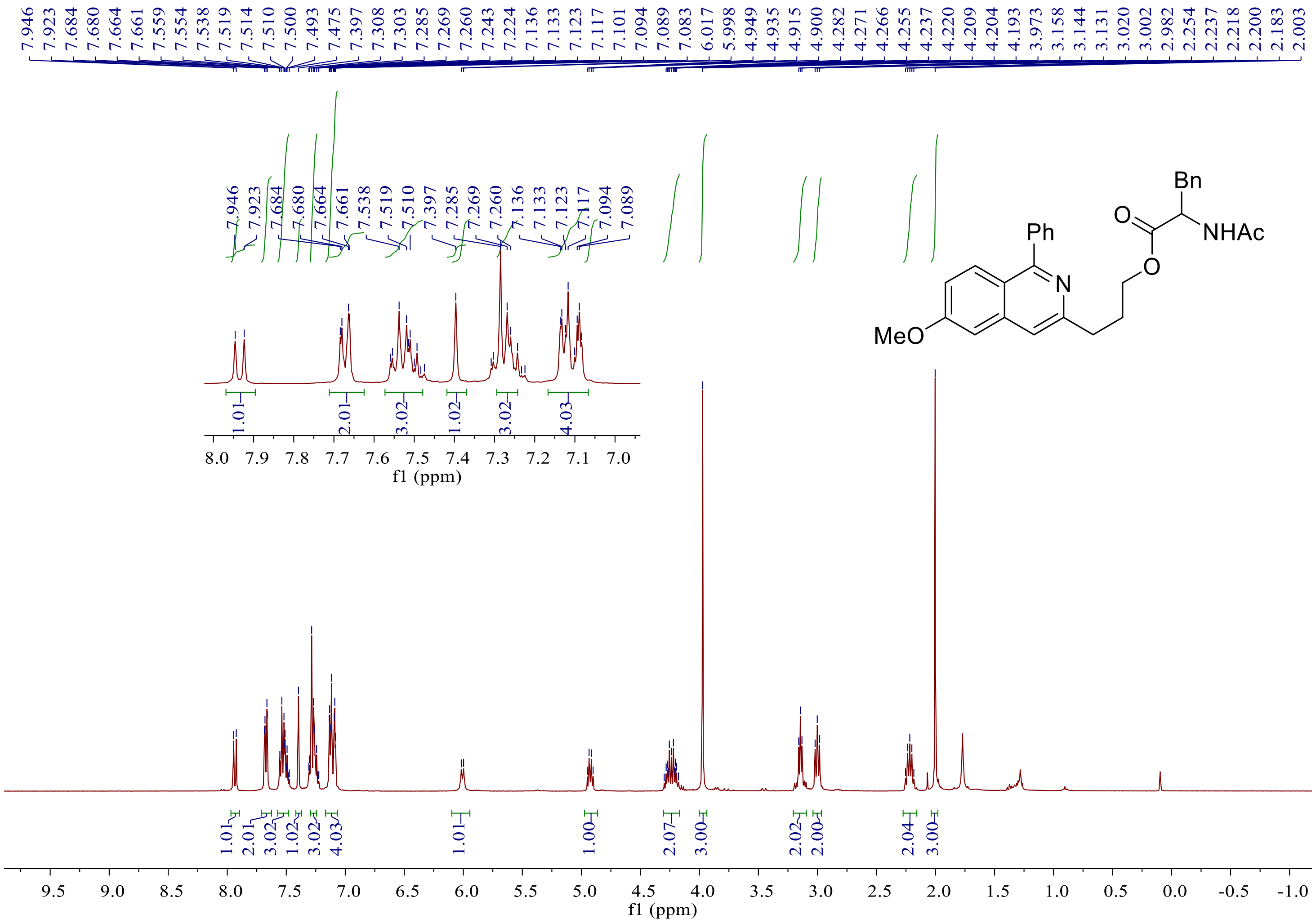

Supplementary Figure 140. ^{1}H NMR (400 MHz, $CDCl_3$) of **2af**

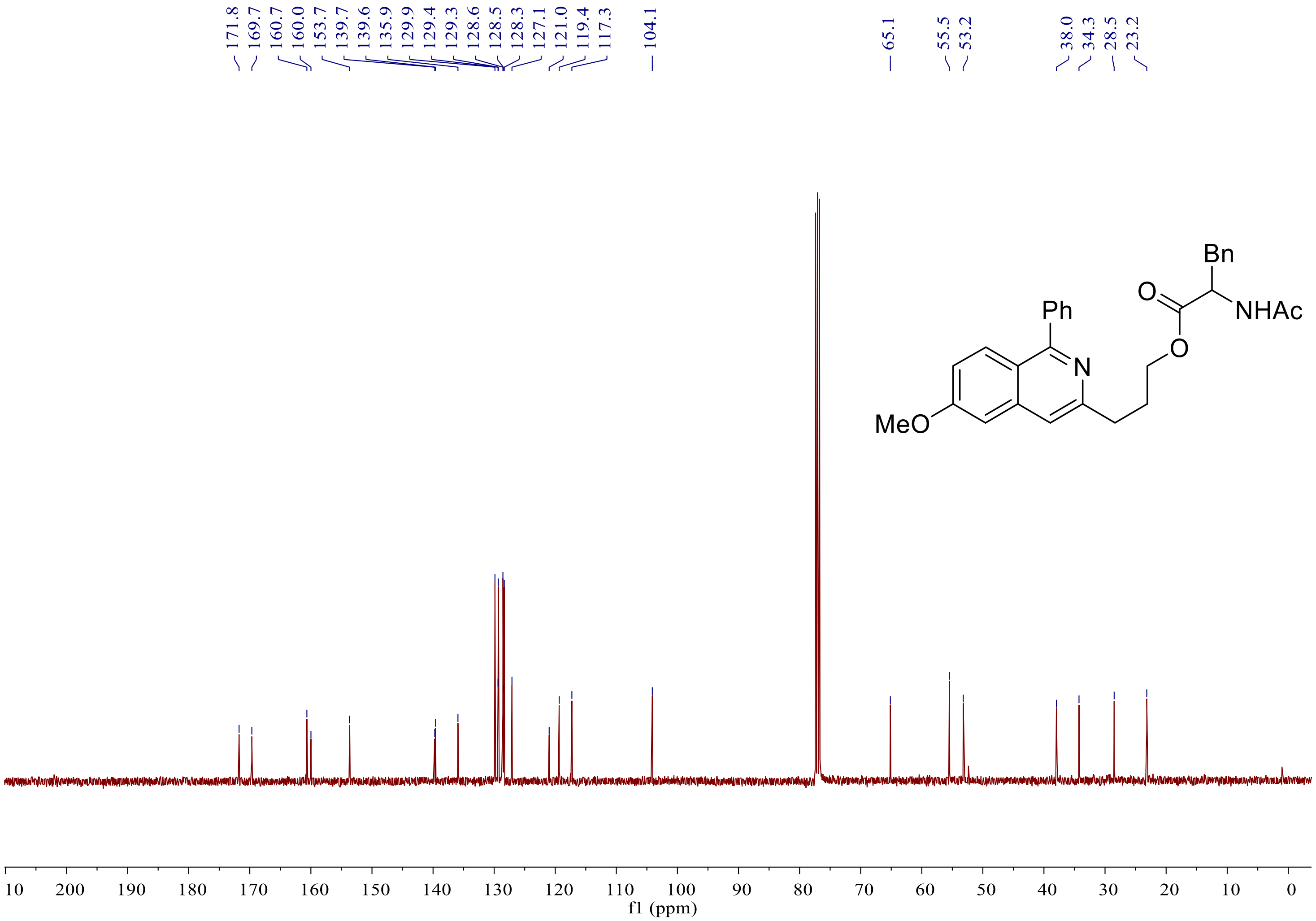

Supplementary Figure 141. ^{13}C NMR (100 MHz, $CDCl_3$) of **2af**

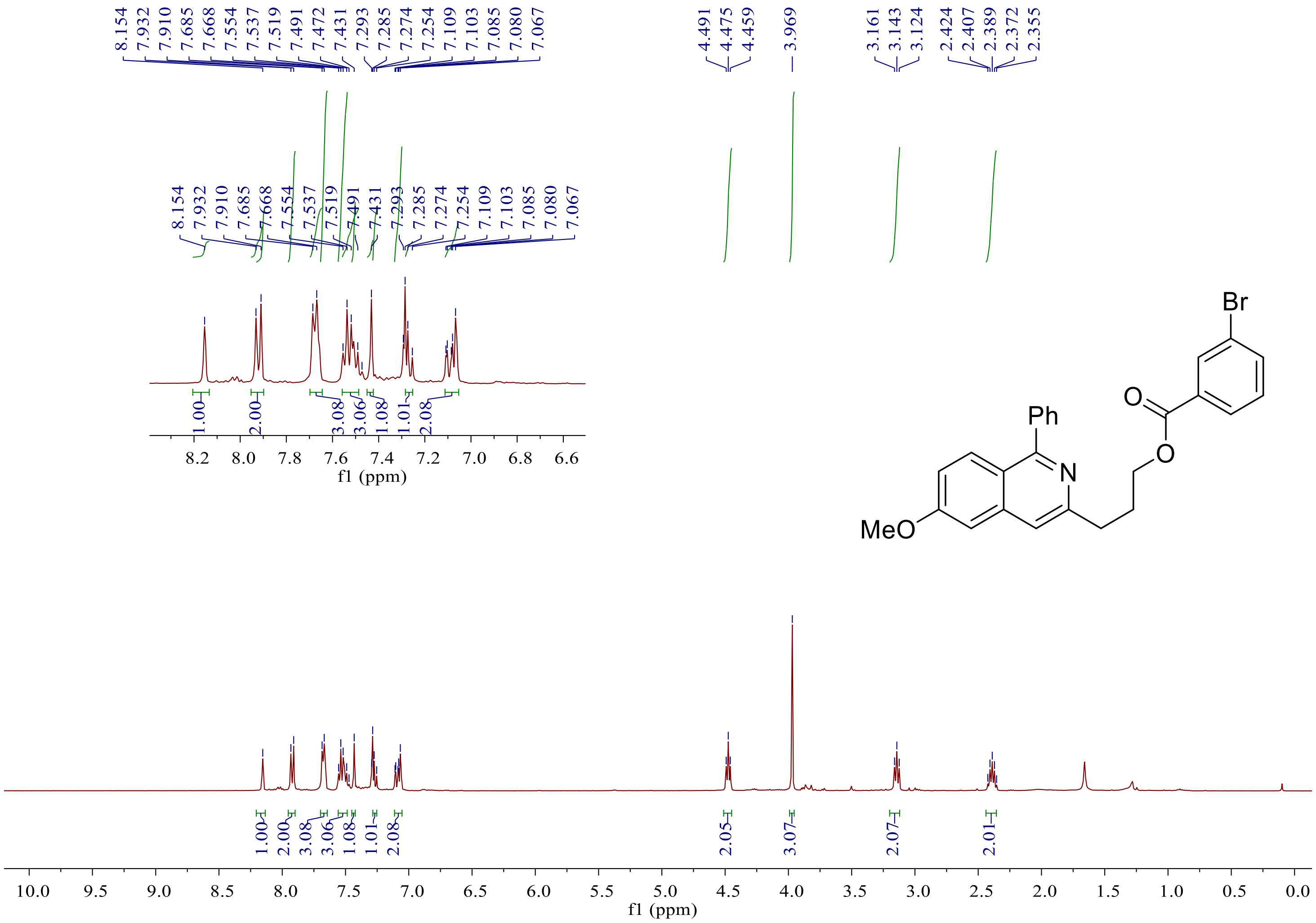

Supplementary Figure 142. ^{1}H NMR (400 MHz, $CDCl_3$) of **2ag**

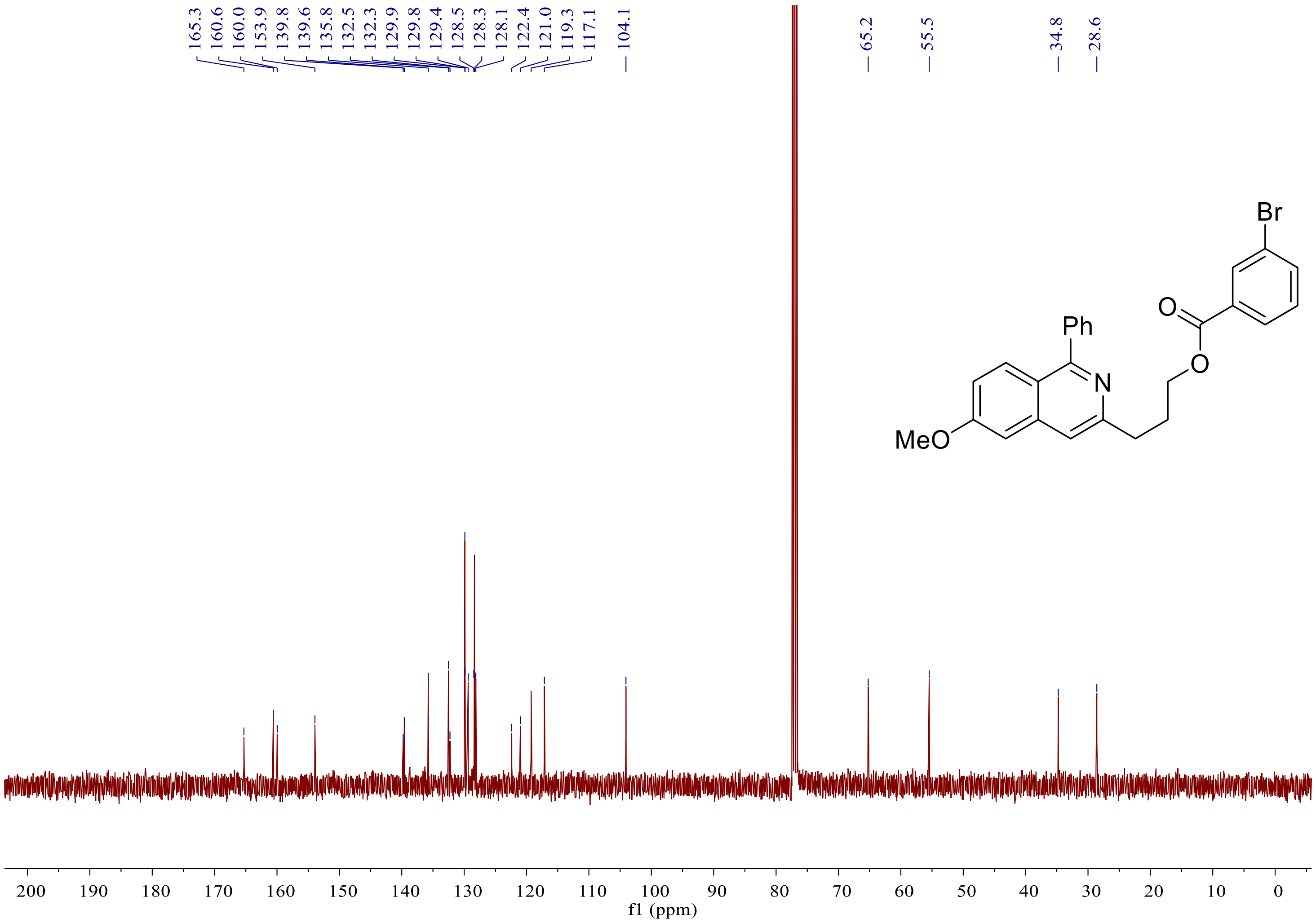

Supplementary Figure 143. ^{13}C NMR (100 MHz, $CDCl_3$) of **2ag**

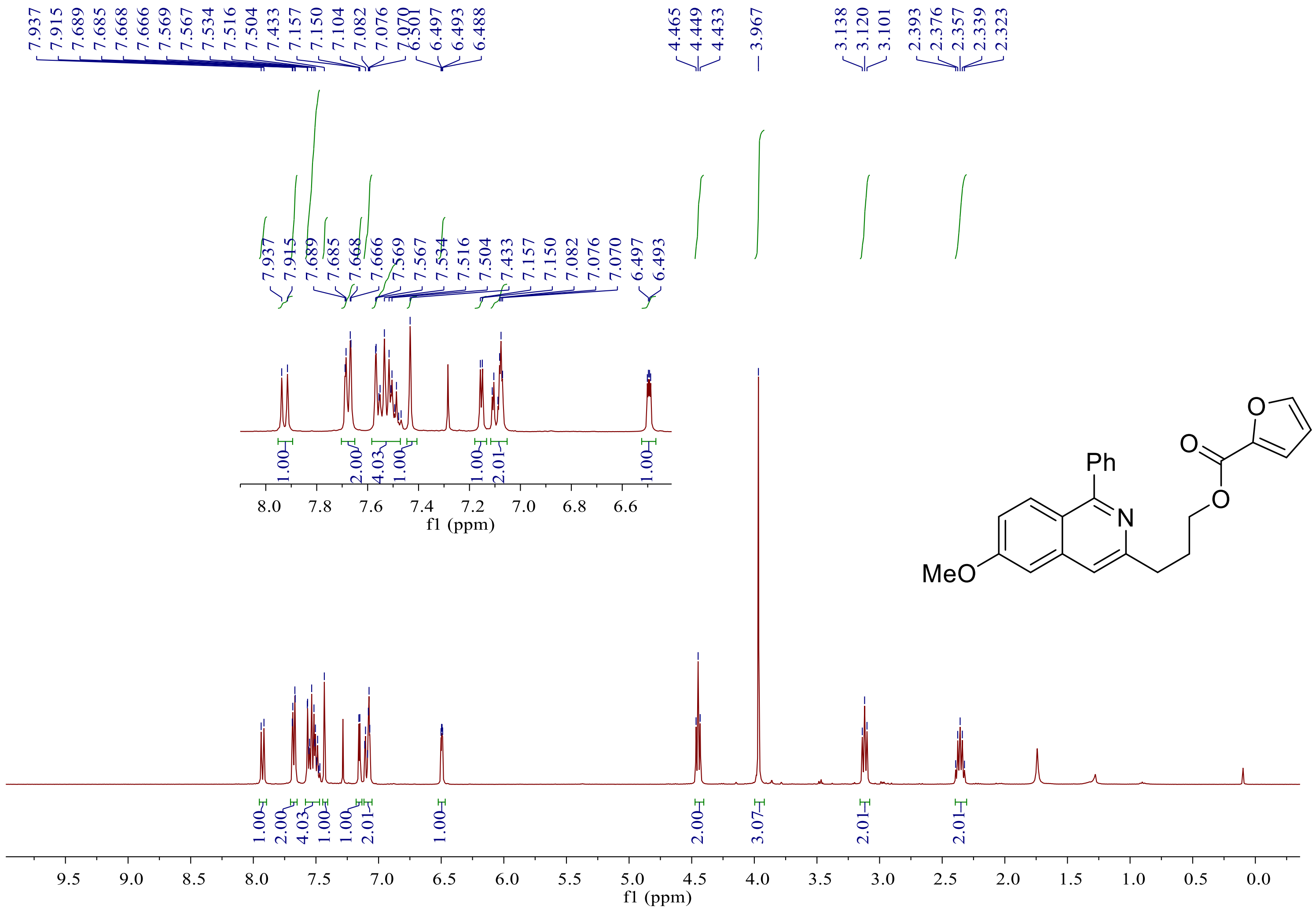

Supplementary Figure 144. ^{1}H NMR (400 MHz, $CDCl_3$) of **2ah**

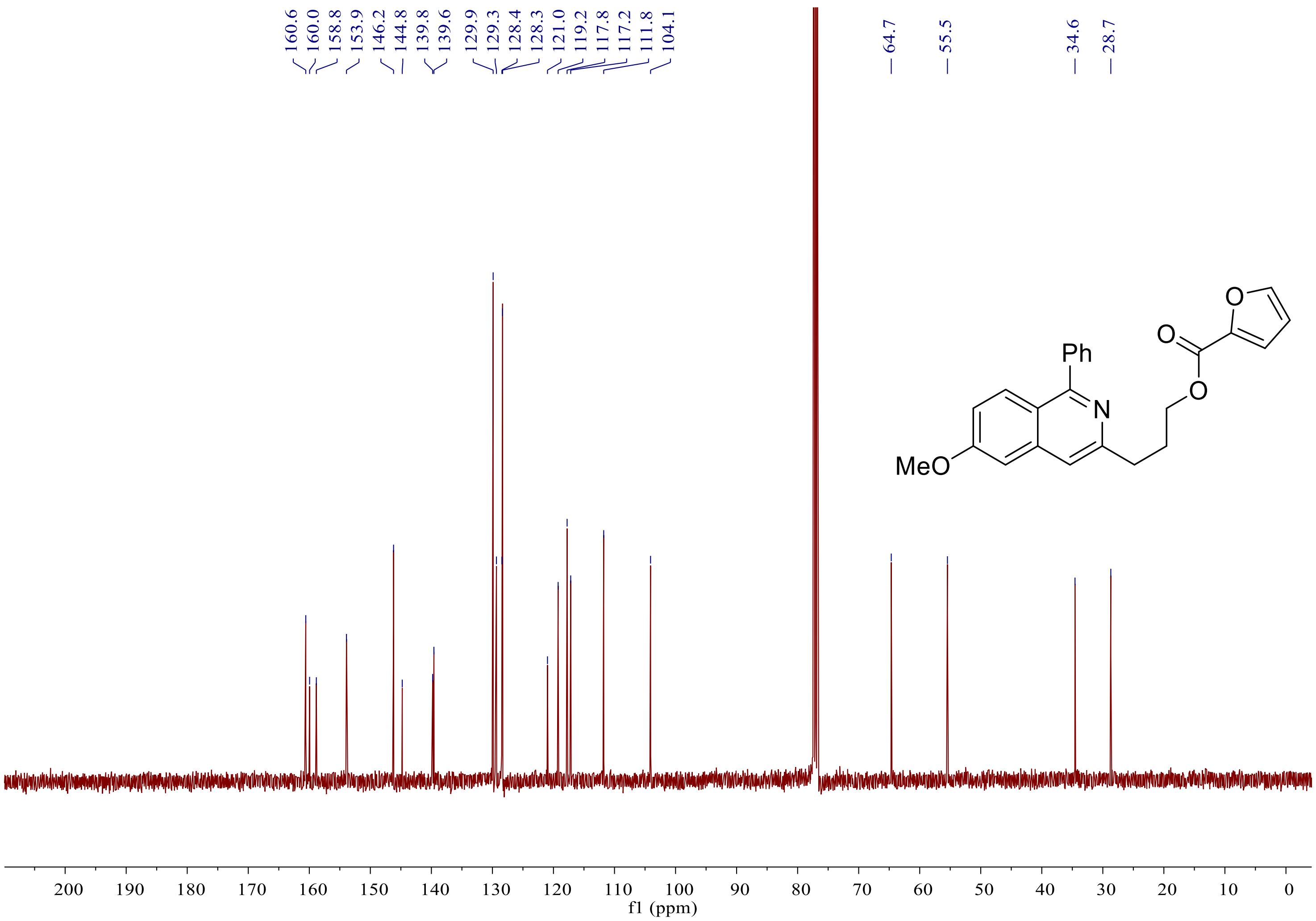

Supplementary Figure 145. ^{13}C NMR (100 MHz, $CDCl_3$) of **2ah**

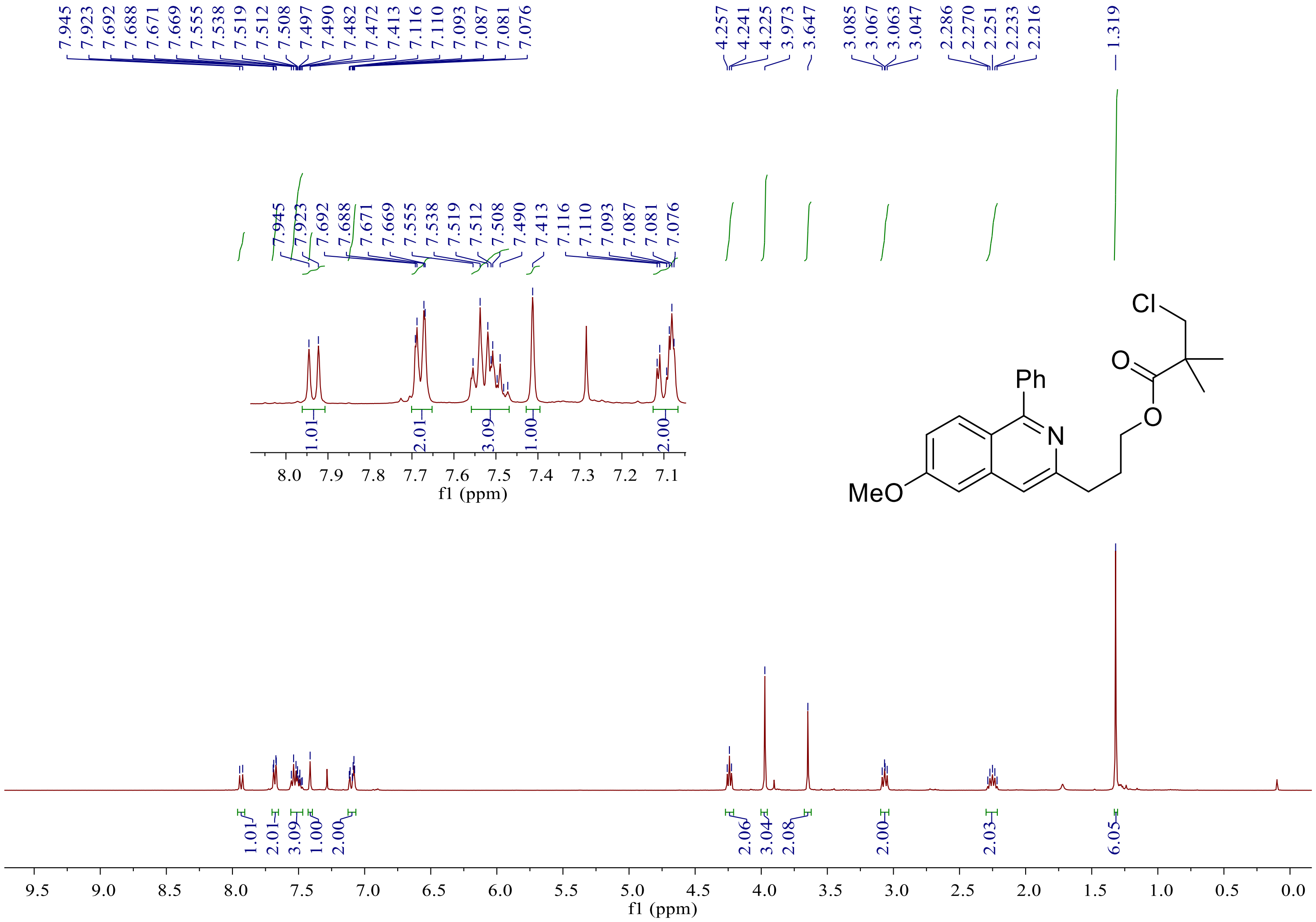

Supplementary Figure 146.^{1}H NMR (400 MHz, $CDCl_3$) of **2ai**

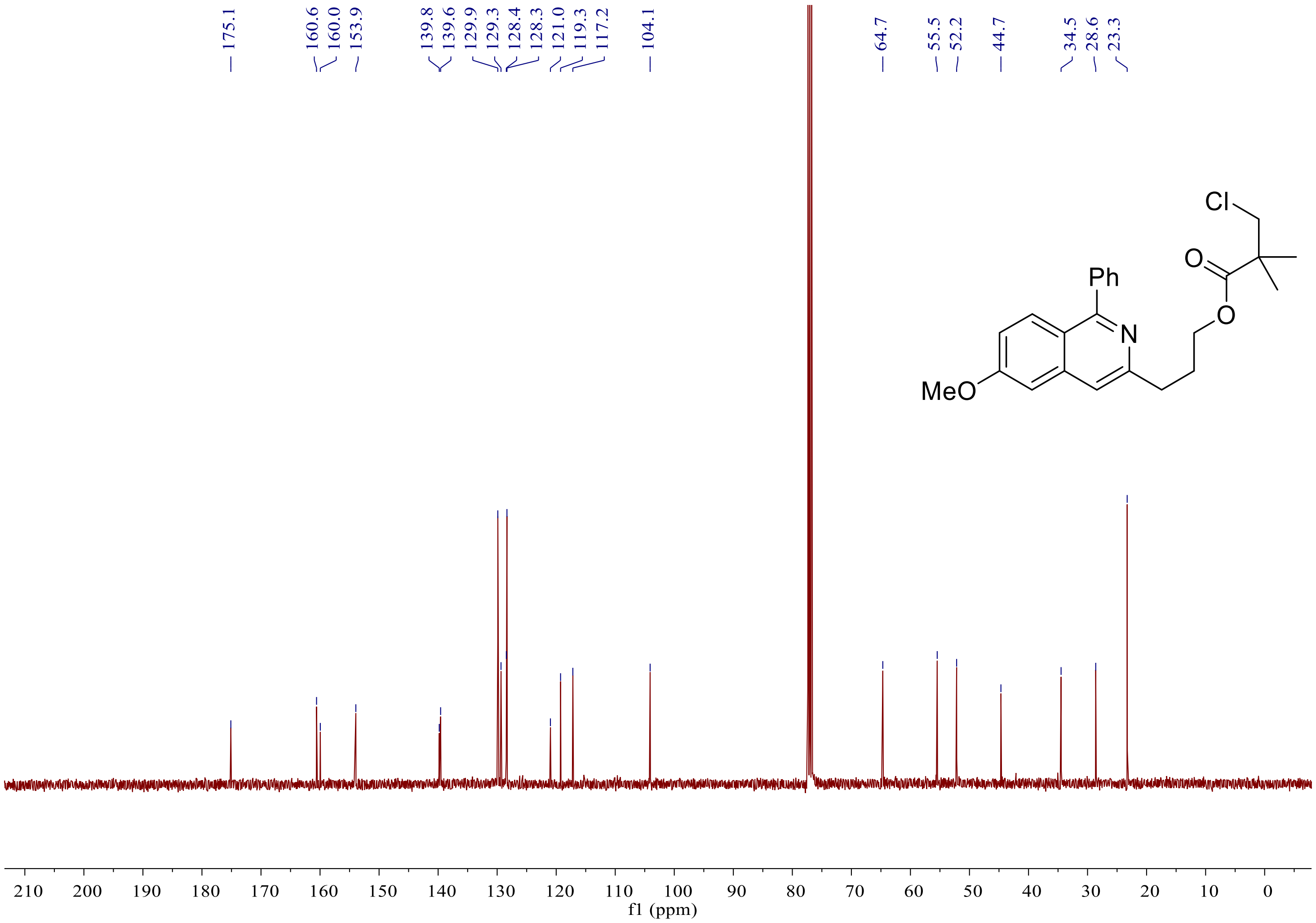

Supplementary Figure 147. ^{13}C NMR (100 MHz, $CDCl_3$) of **2ai**

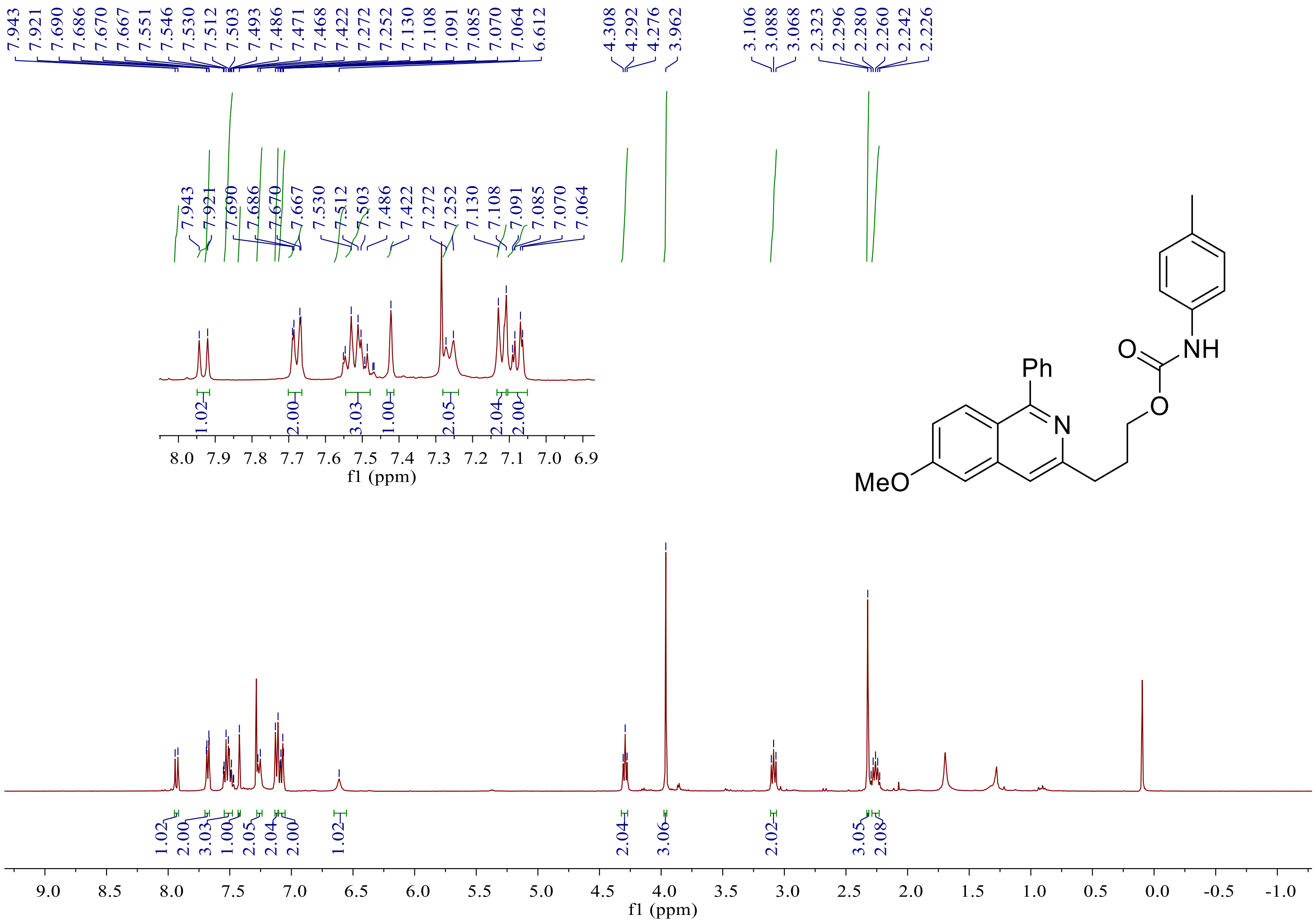

Supplementary Figure 148. ^{1}H NMR (400 MHz, $CDCl_3$) of **2aj**

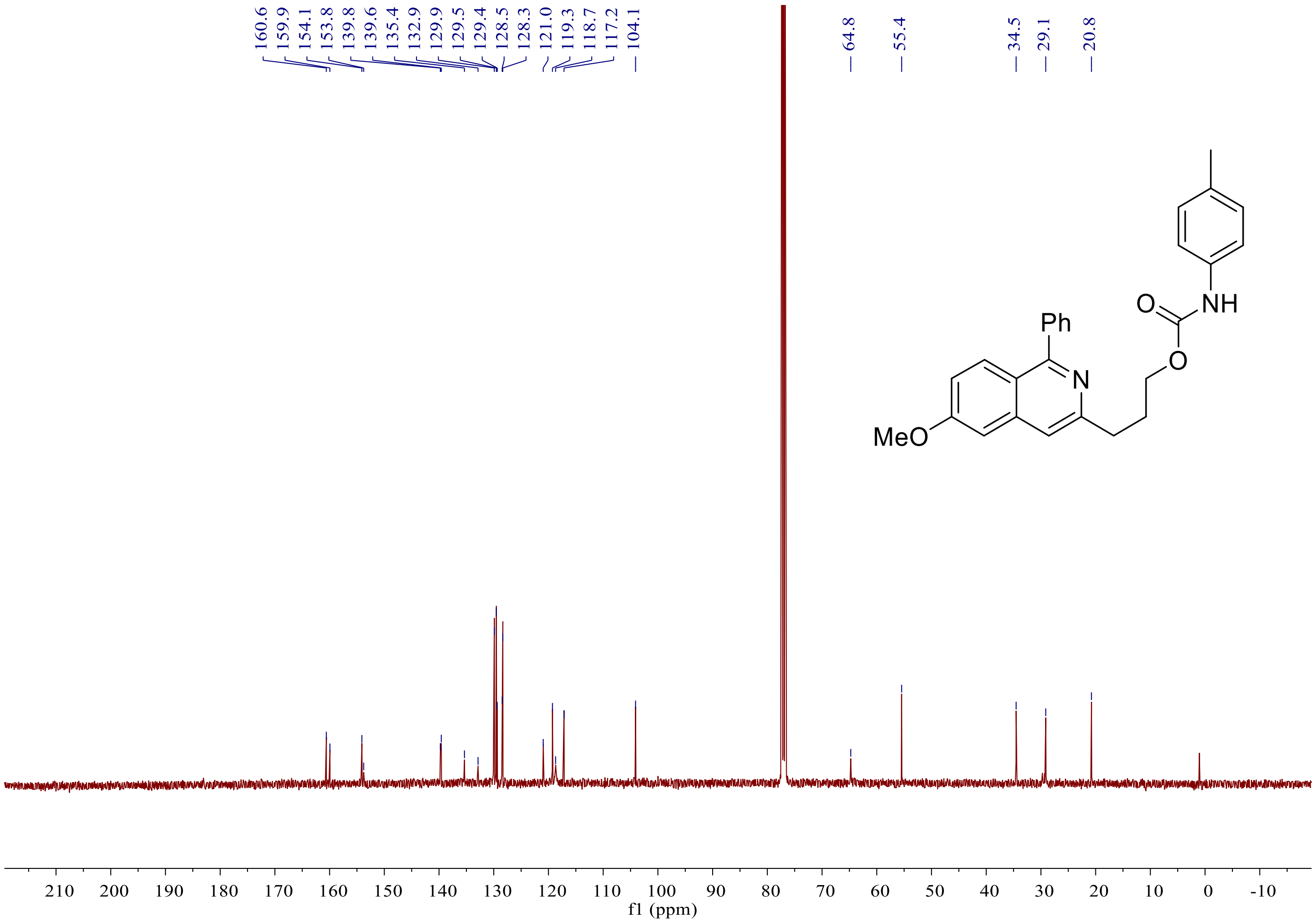

Supplementary Figure 149. ^{13}C NMR (100 MHz, $CDCl_3$) of **2aj**

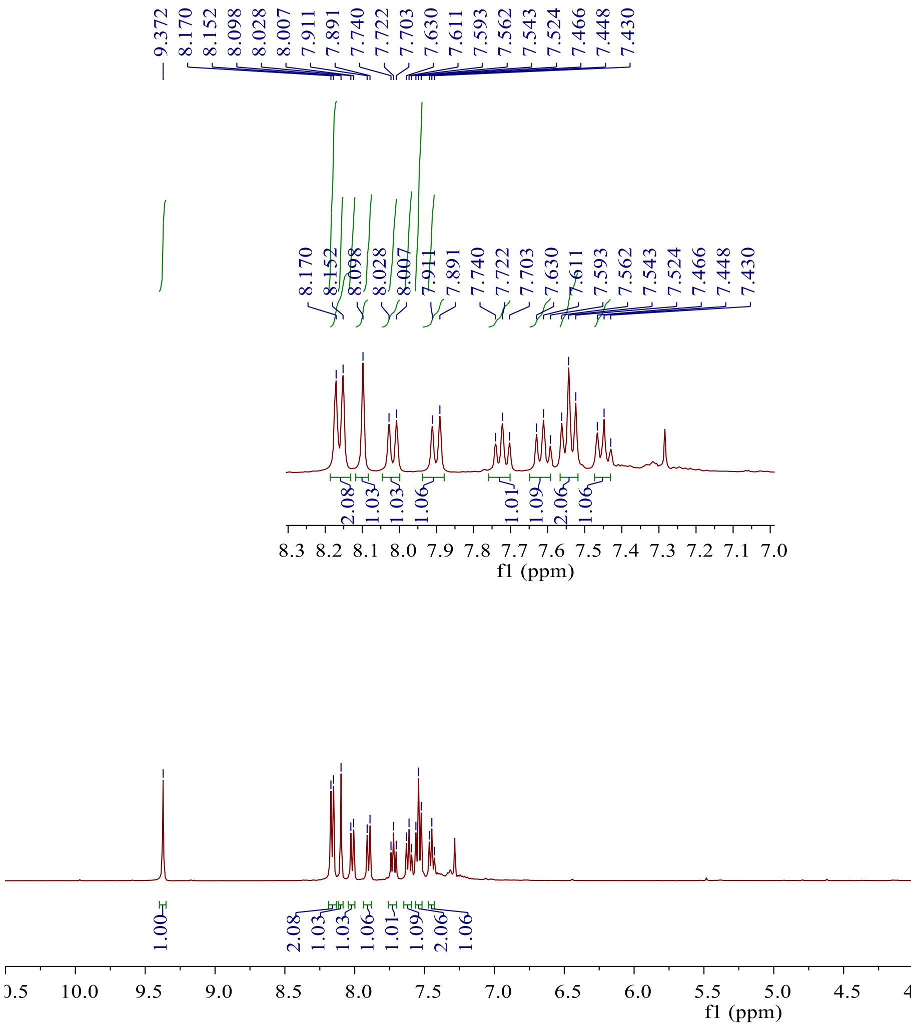

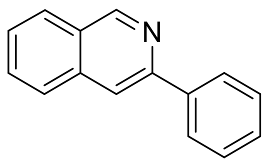

Supplementary Figure 150. ^{1}H NMR (400 MHz, $CDCl_3$) of **2ak**

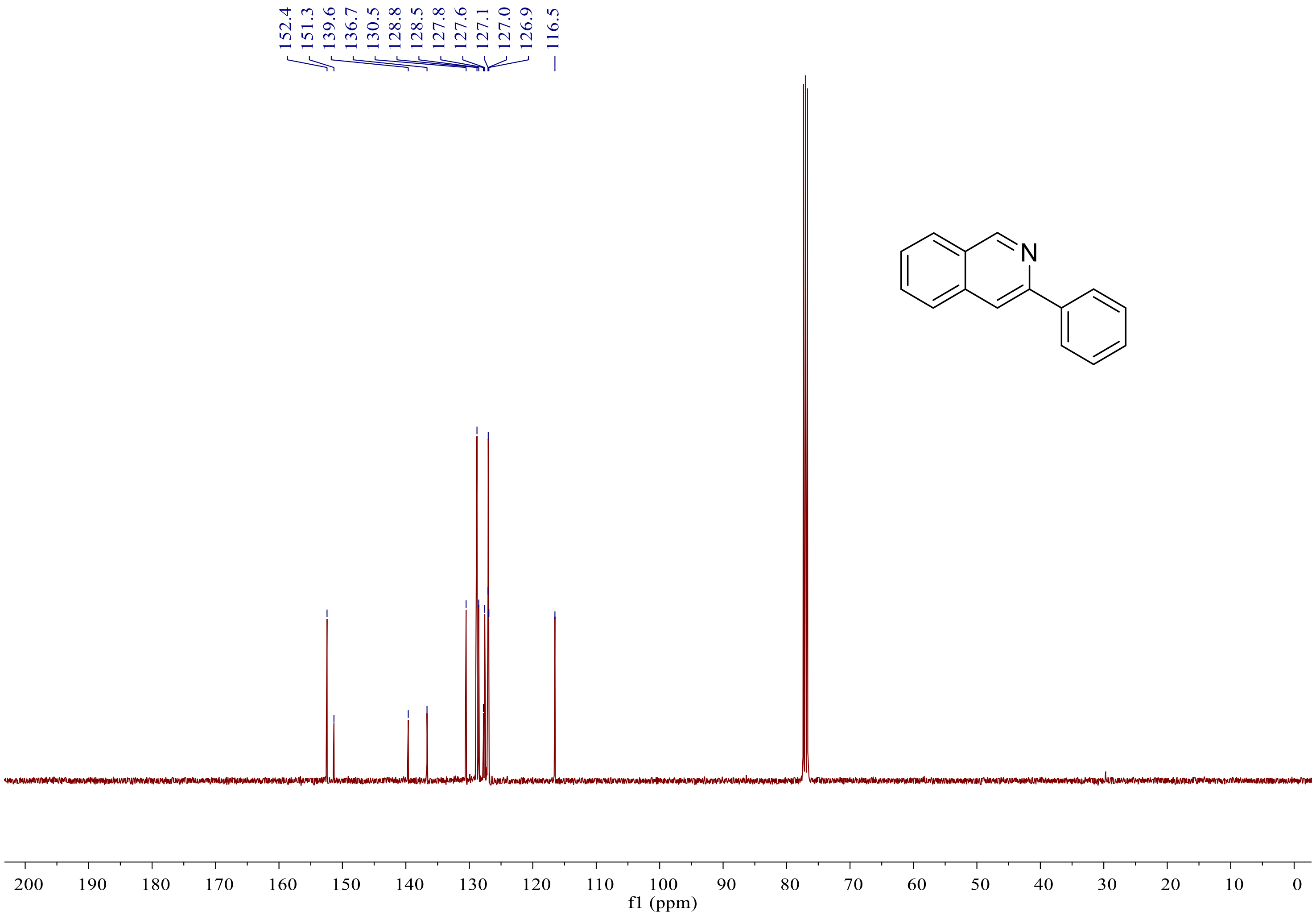

Supplementary Figure 151. ^{13}C NMR (100 MHz, $CDCl_3$) of **2ak**

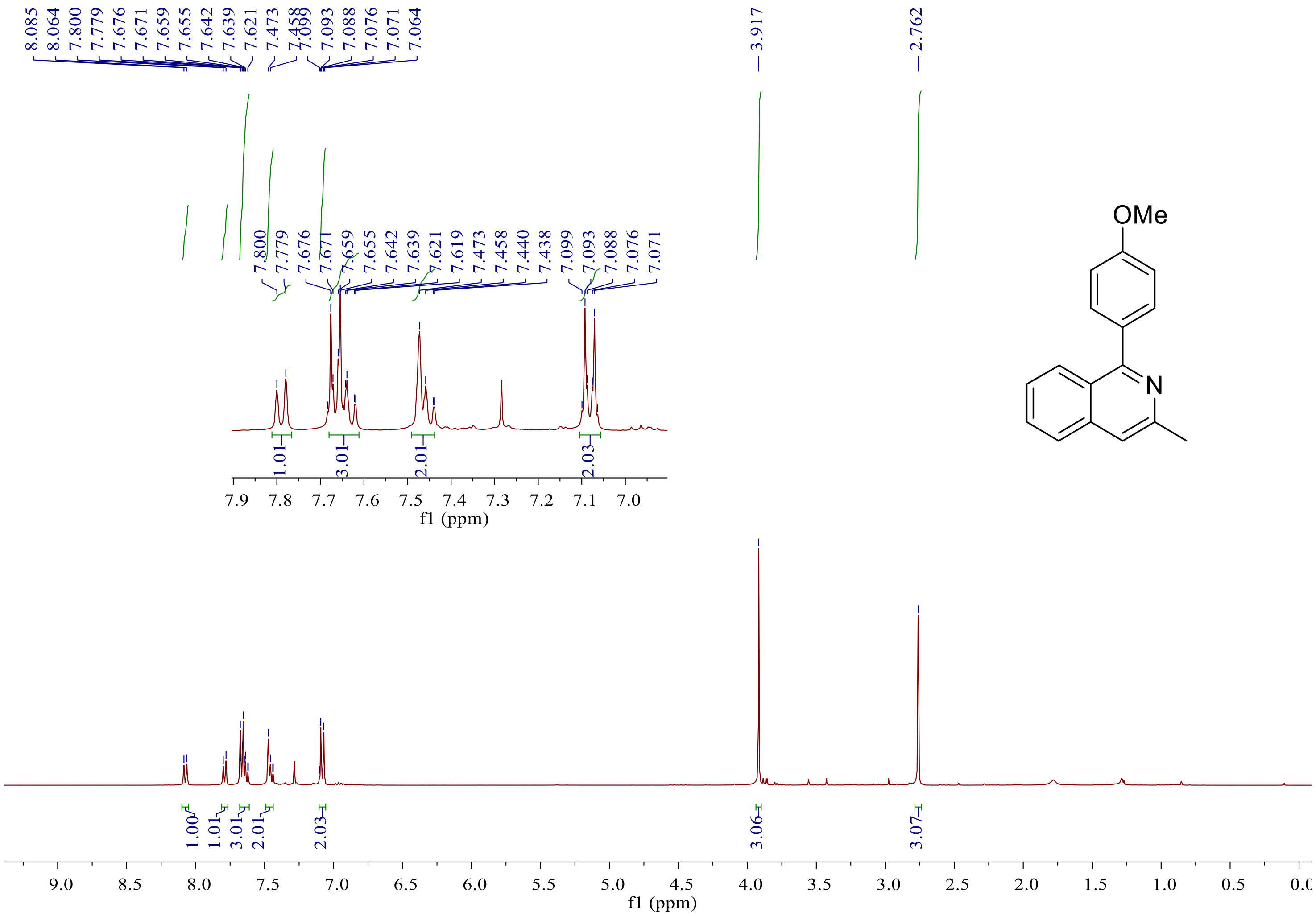

Supplementary Figure 152. ^{1}H NMR (400 MHz, $CDCl_3$) of **2al**

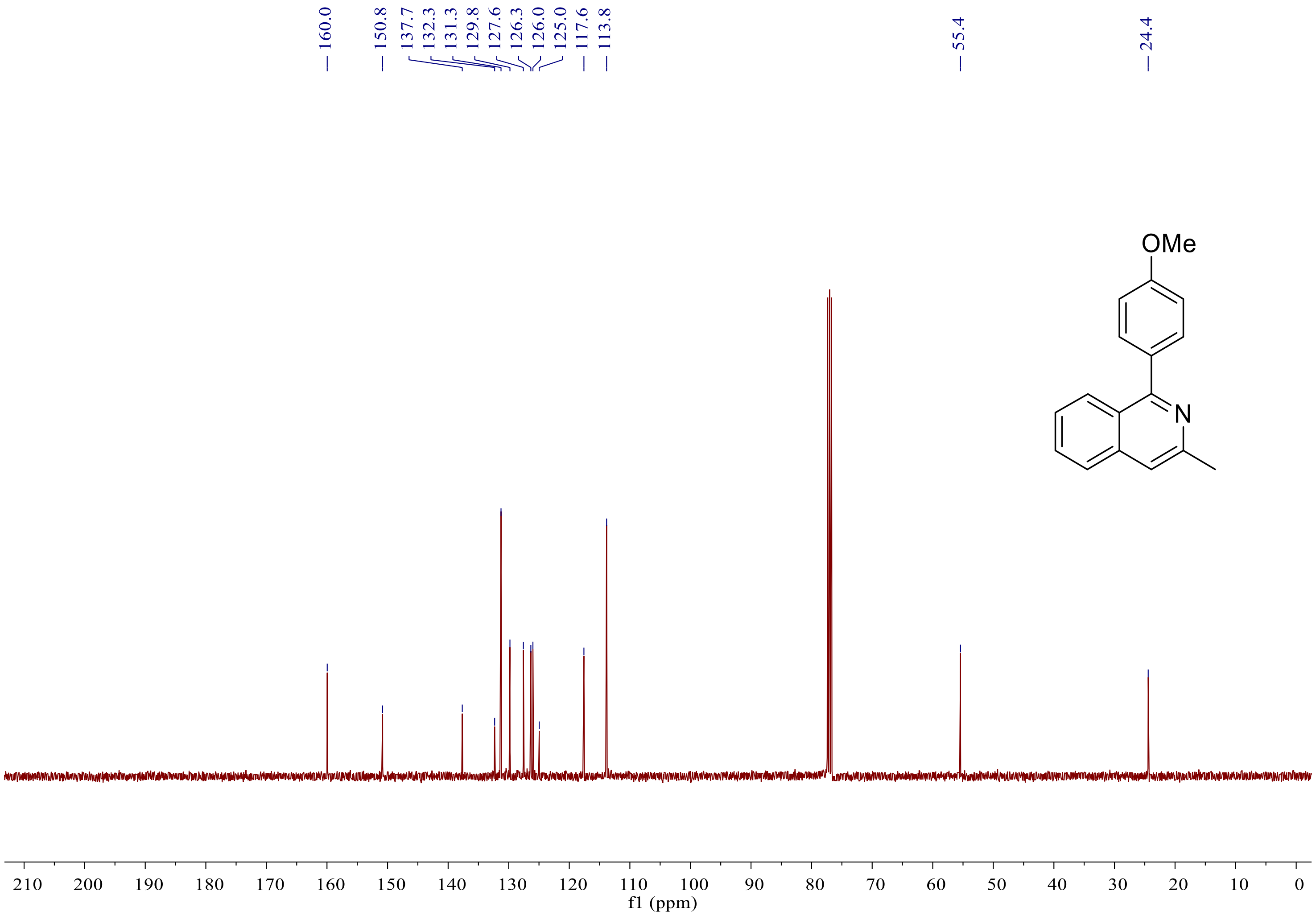

Supplementary Figure 153. ^{13}C NMR (100 MHz, $CDCl_3$) of **2al**

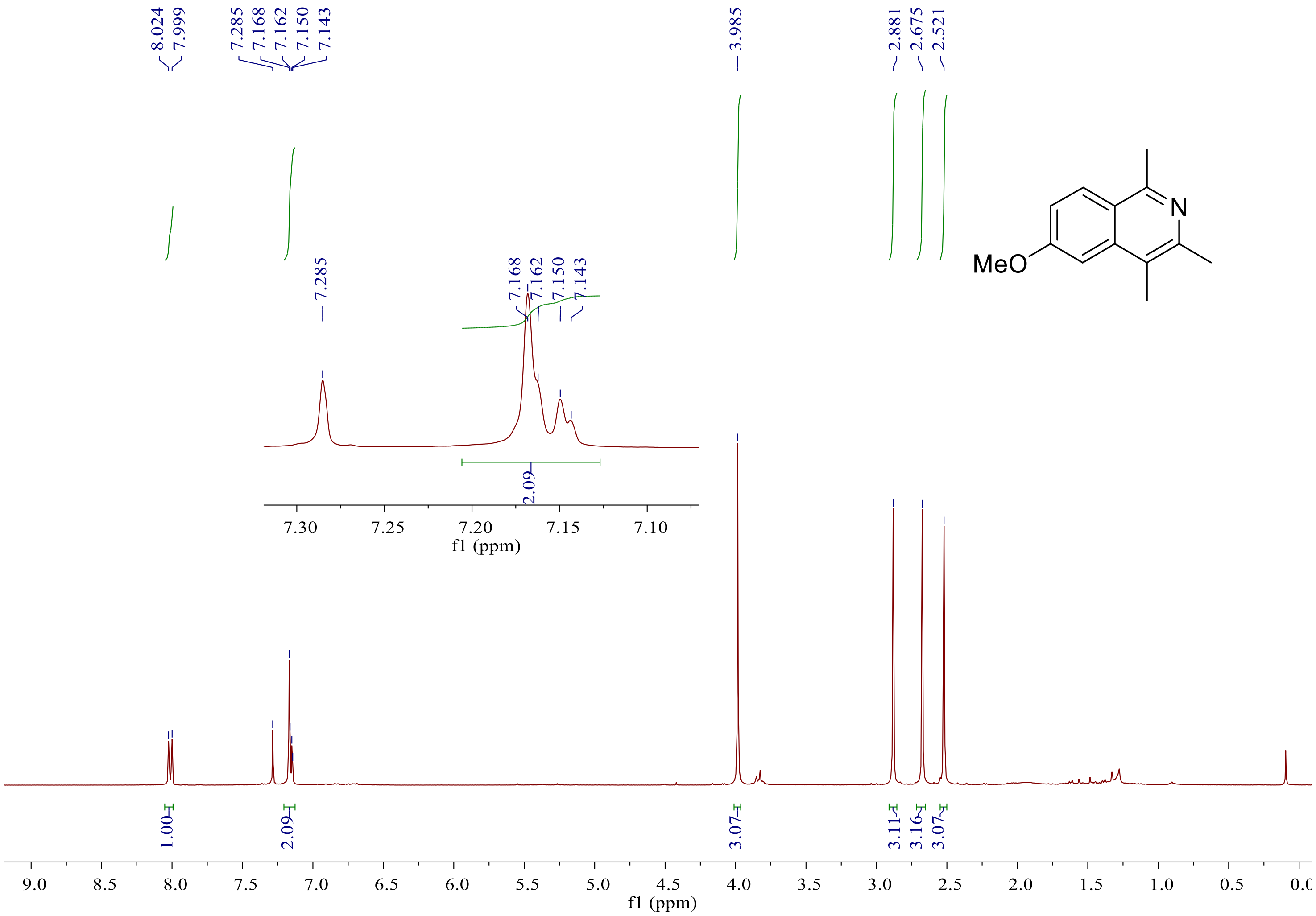

Supplementary Figure 154. ^{1}H NMR (400 MHz, $CDCl_3$) of **2am**

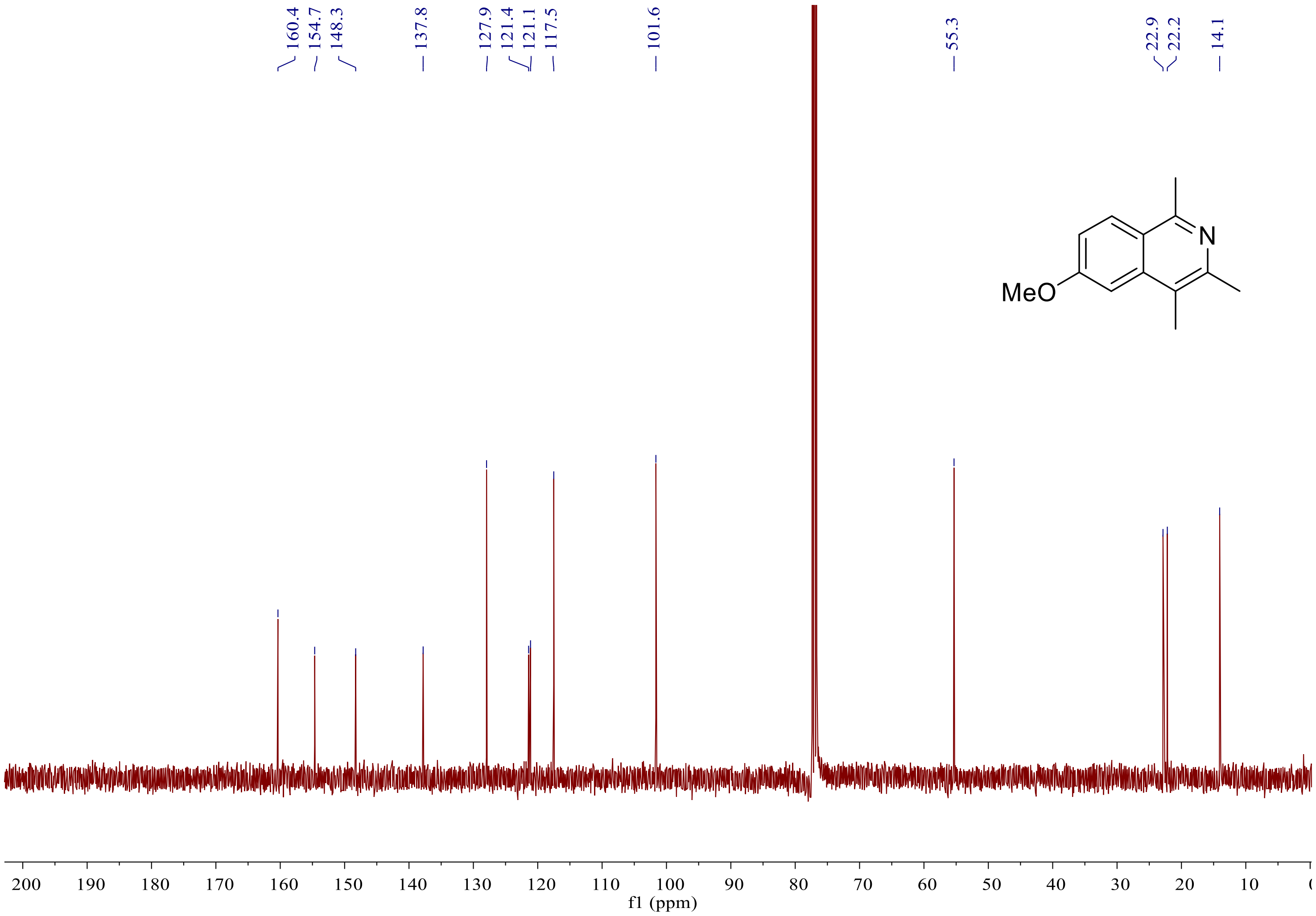

Supplementary Figure 155. ^{13}C NMR (100 MHz, $CDCl_3$) of **2am**

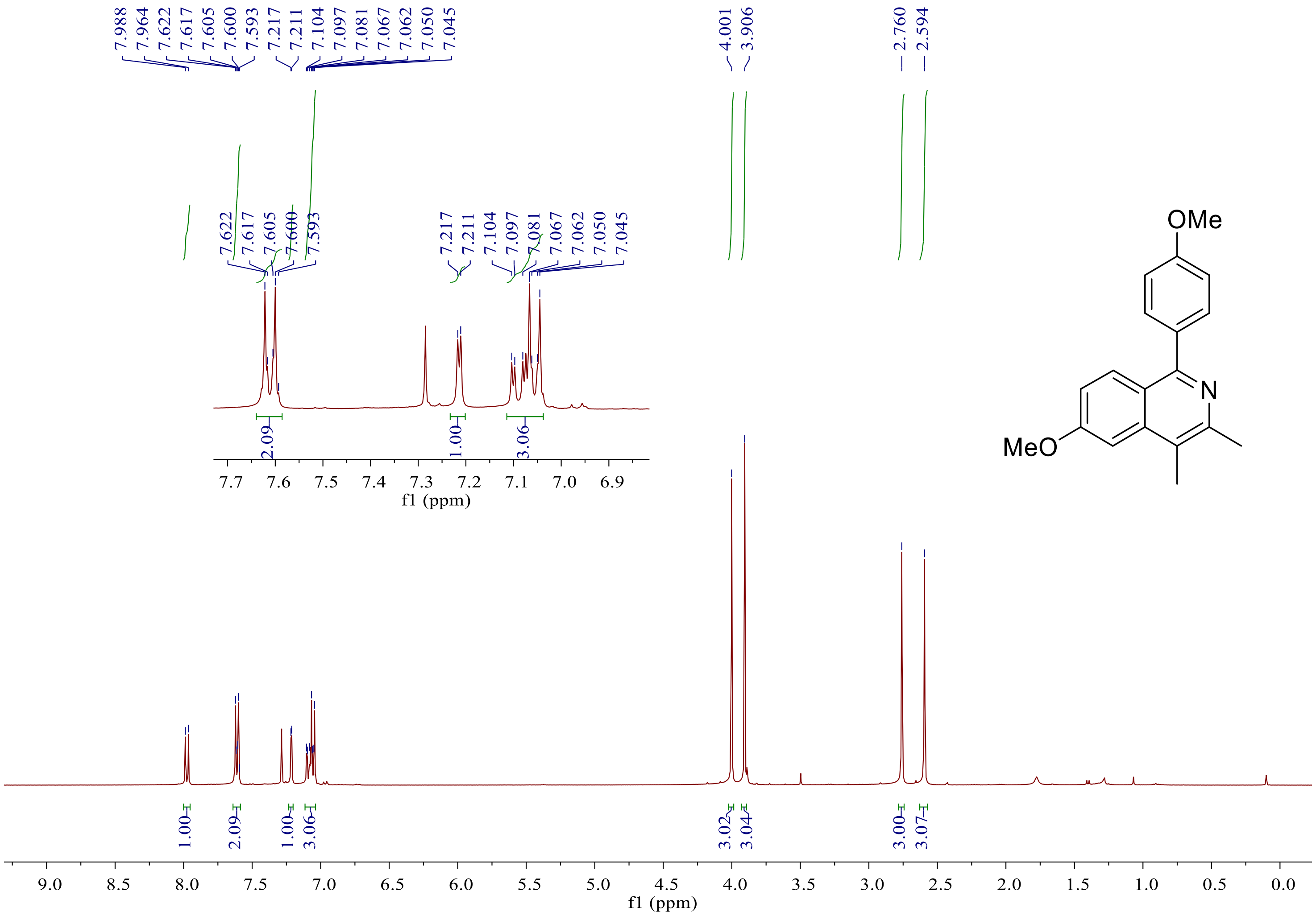

Supplementary Figure 156. ^{1}H NMR (400 MHz, $CDCl_3$) of **2an**

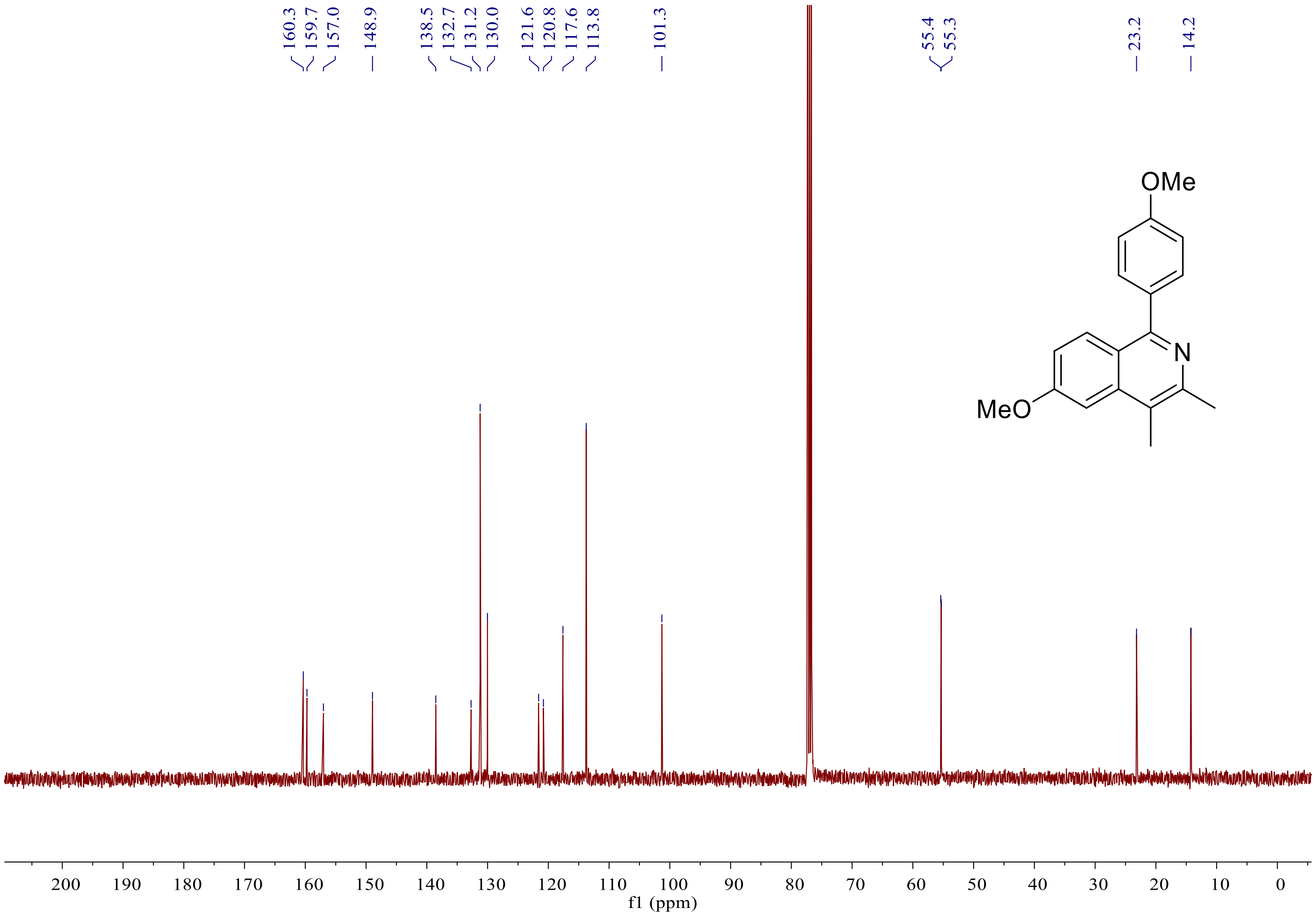

Supplementary Figure 157. ^{13}C NMR (100 MHz, $CDCl_3$) of **2an**

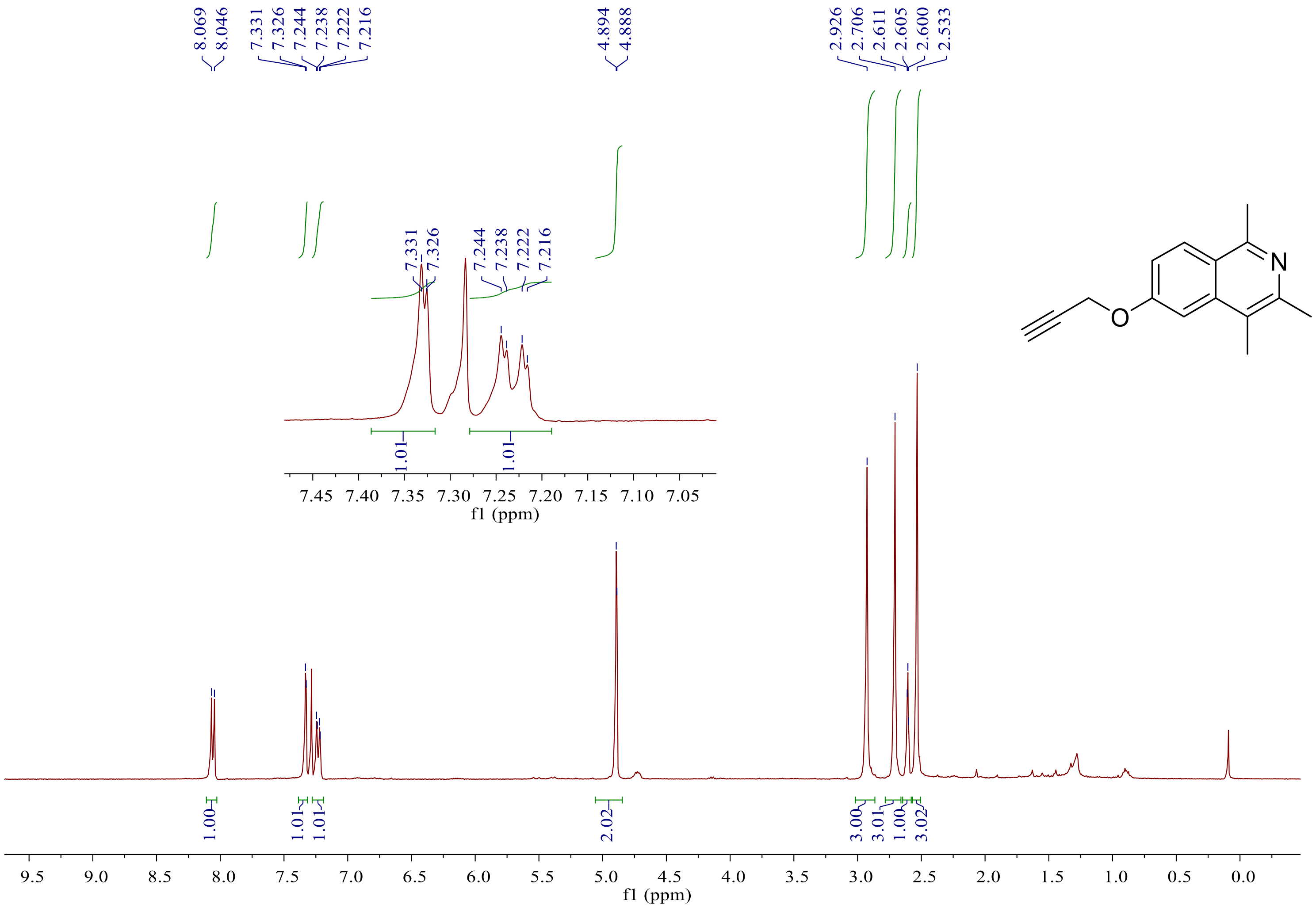

Supplementary Figure 158. ^{1}H NMR (400 MHz, $CDCl_3$) of **2ao**

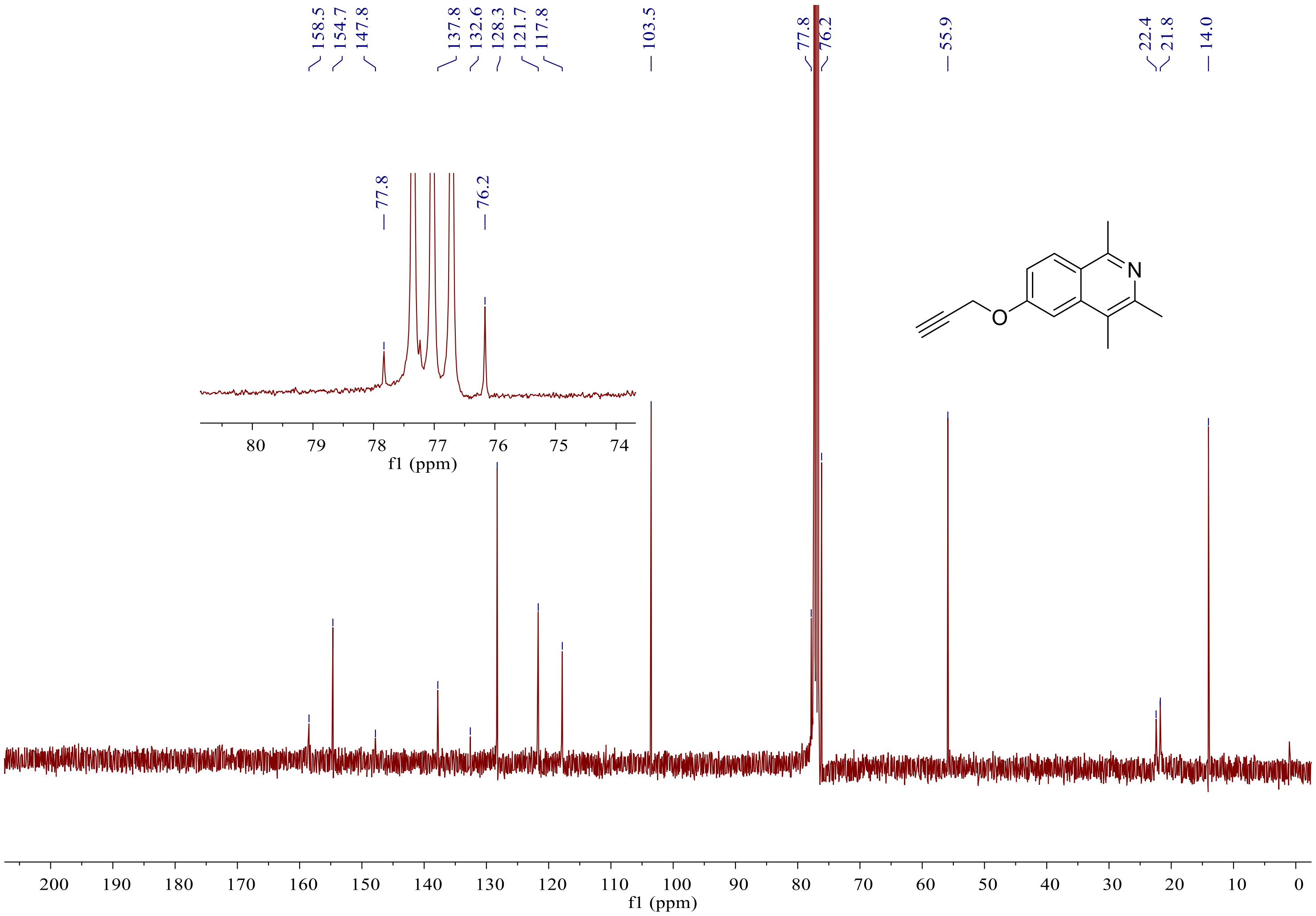

Supplementary Figure 159. ^{13}C NMR (100 MHz, $CDCl_3$) of **2ao**

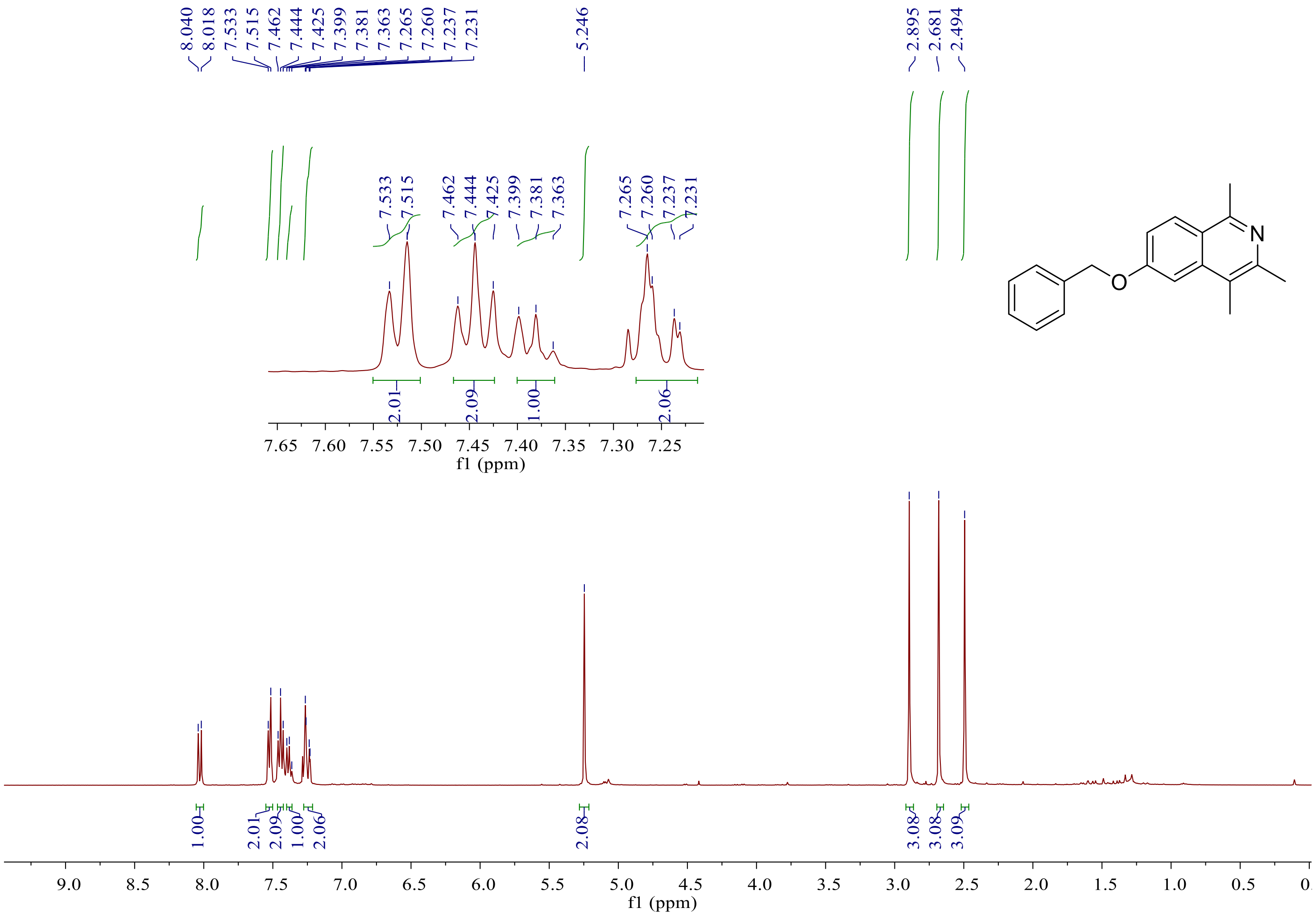

Supplementary Figure 160. ^{1}H NMR (400 MHz, $CDCl_3$) of **2ap**

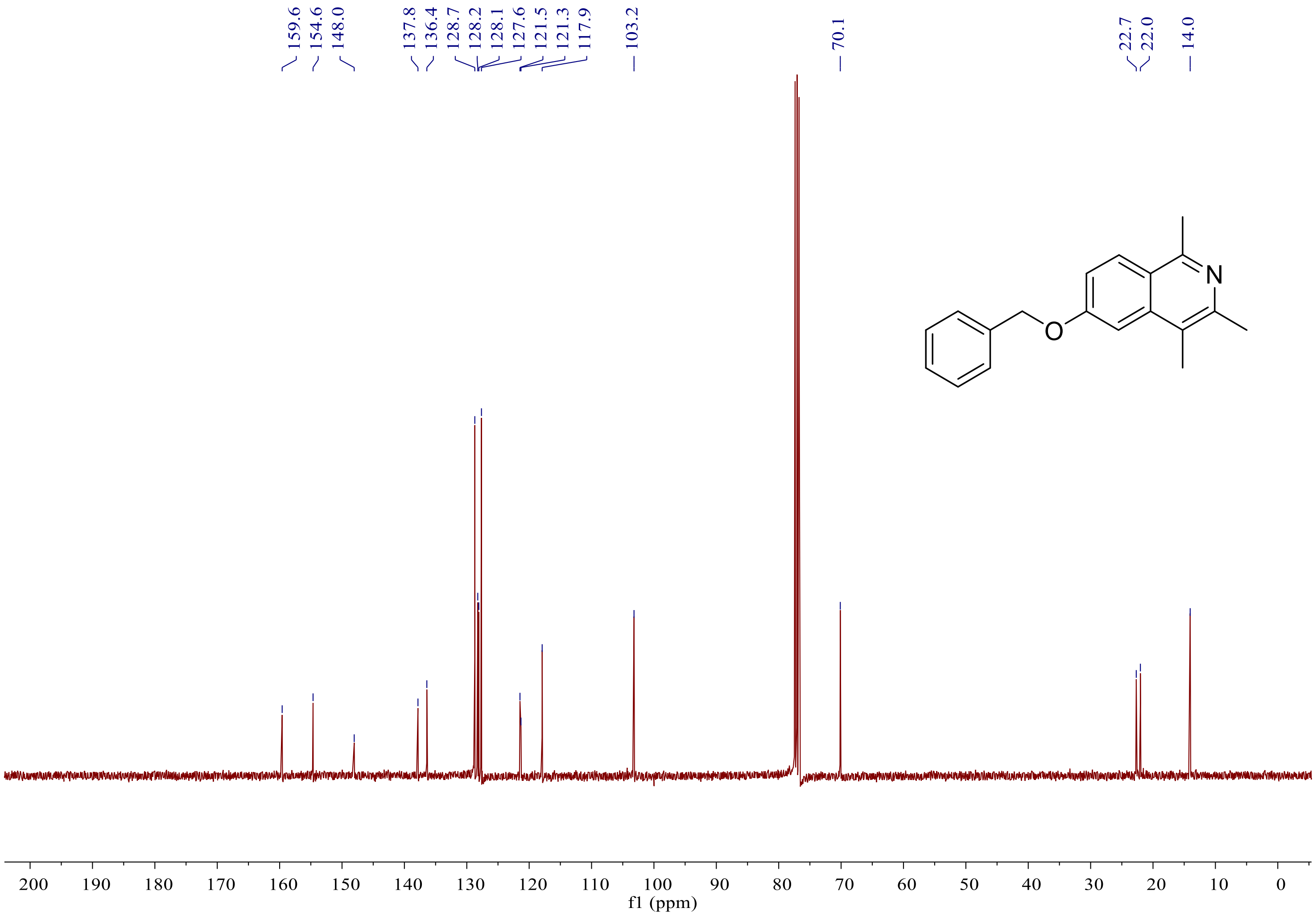

Supplementary Figure 161. ^{13}C NMR (100 MHz, $CDCl_3$) of **2ap**

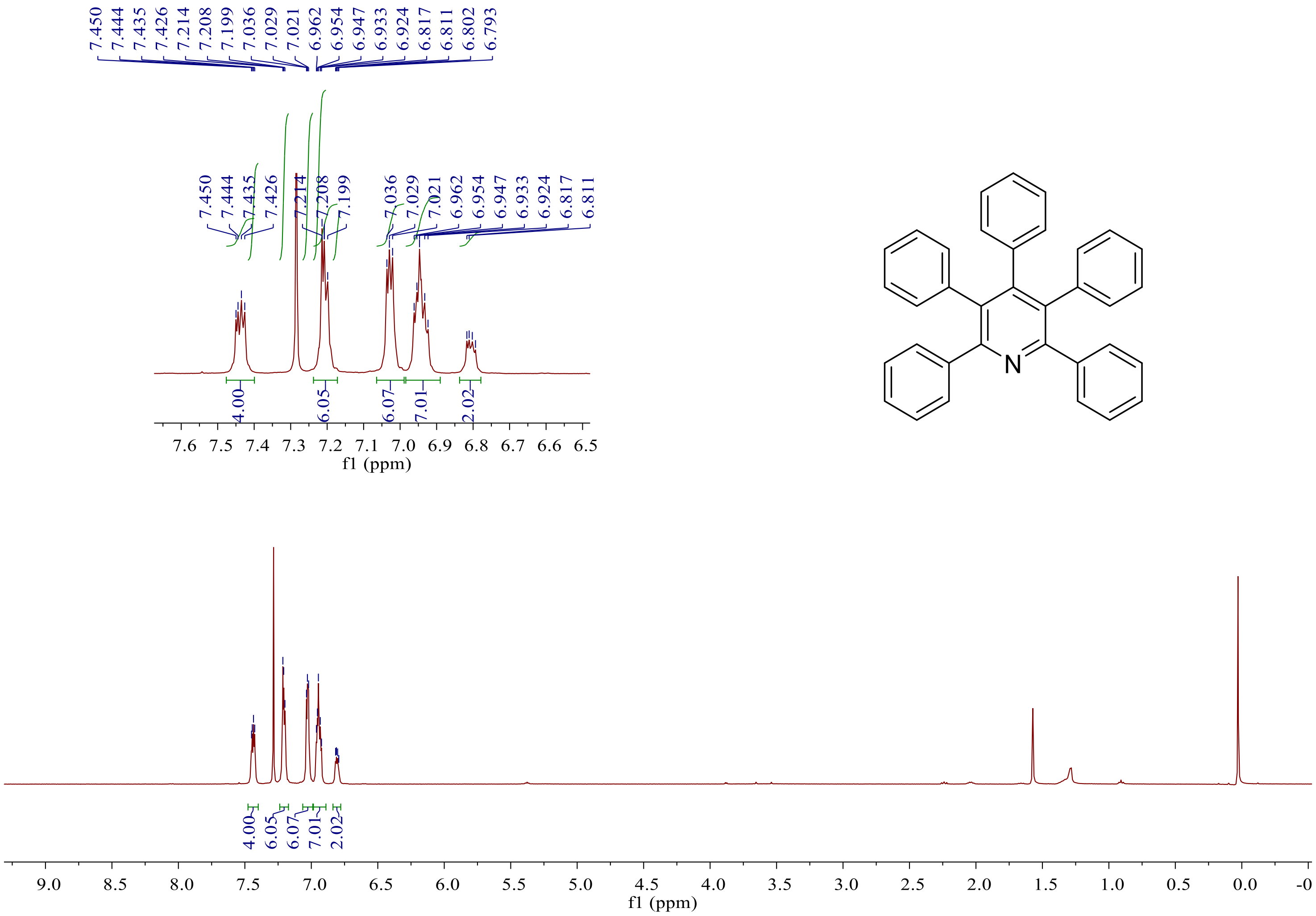

Supplementary Figure 162. ^{1}H NMR (400 MHz, $CDCl_3$) of **2aq**

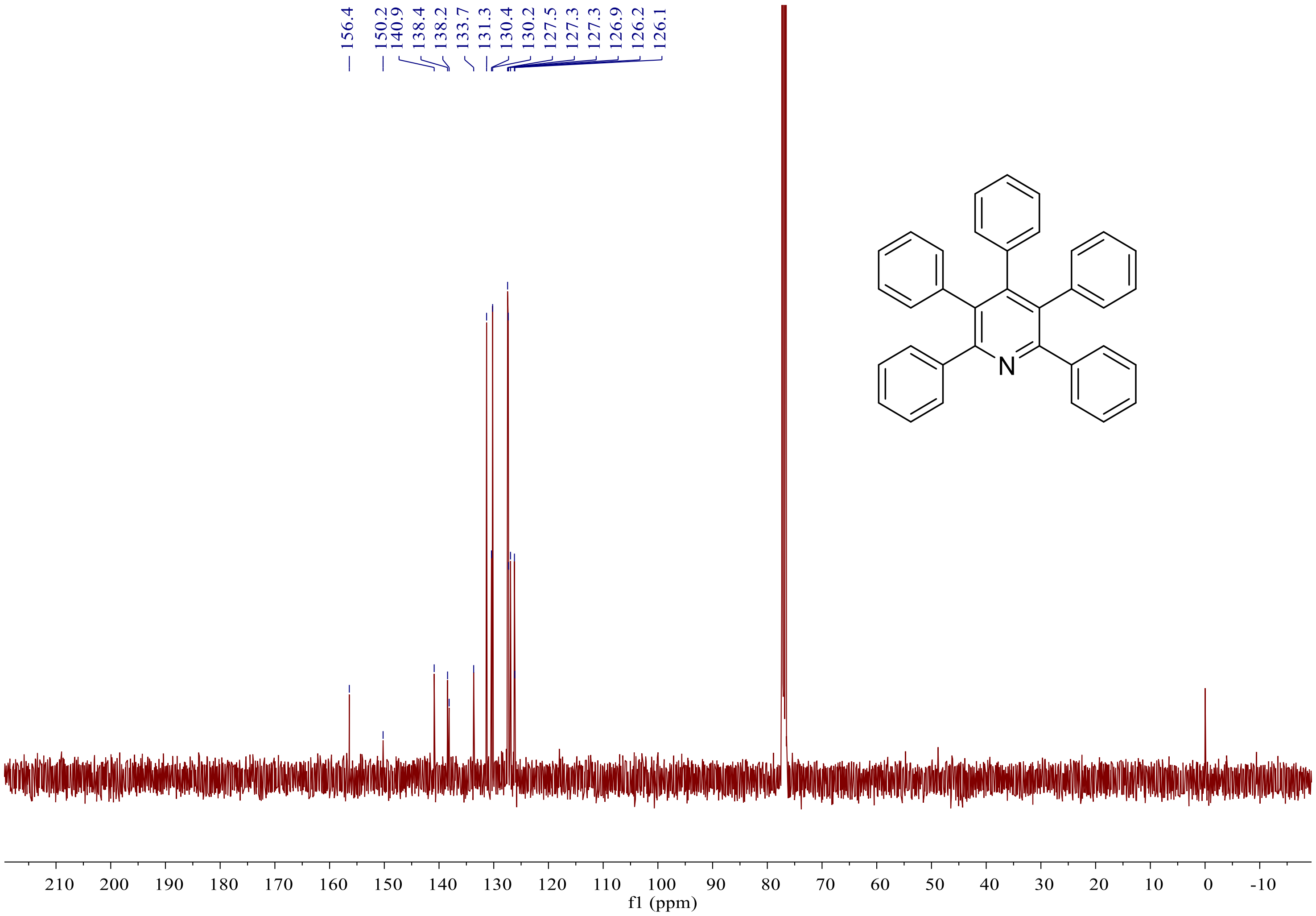

Supplementary Figure 163. ^{13}C NMR (100 MHz, $CDCl_3$) of **2aq**

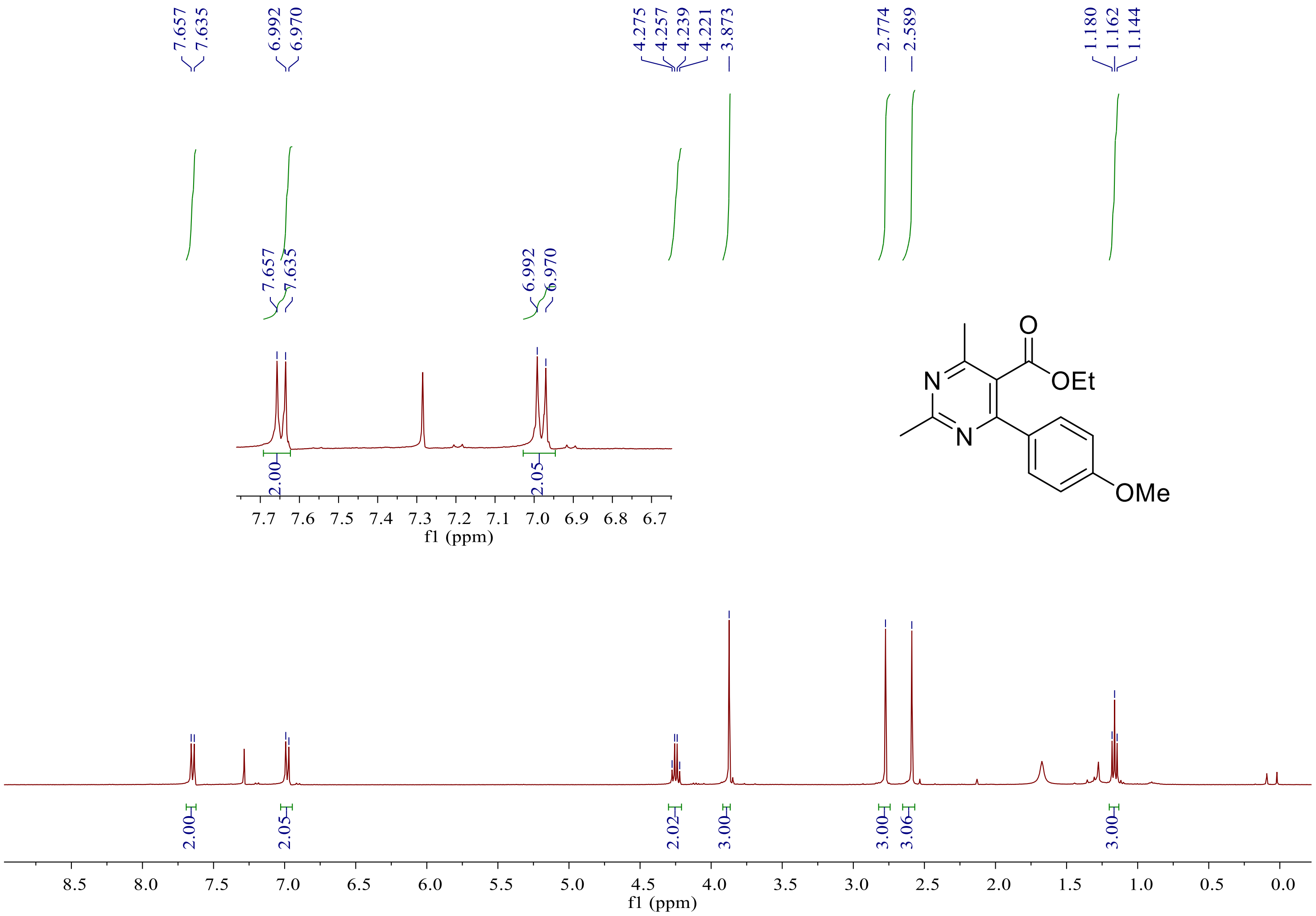

Supplementary Figure 164. ^{1}H NMR (400 MHz, $CDCl_3$) of **2ar**

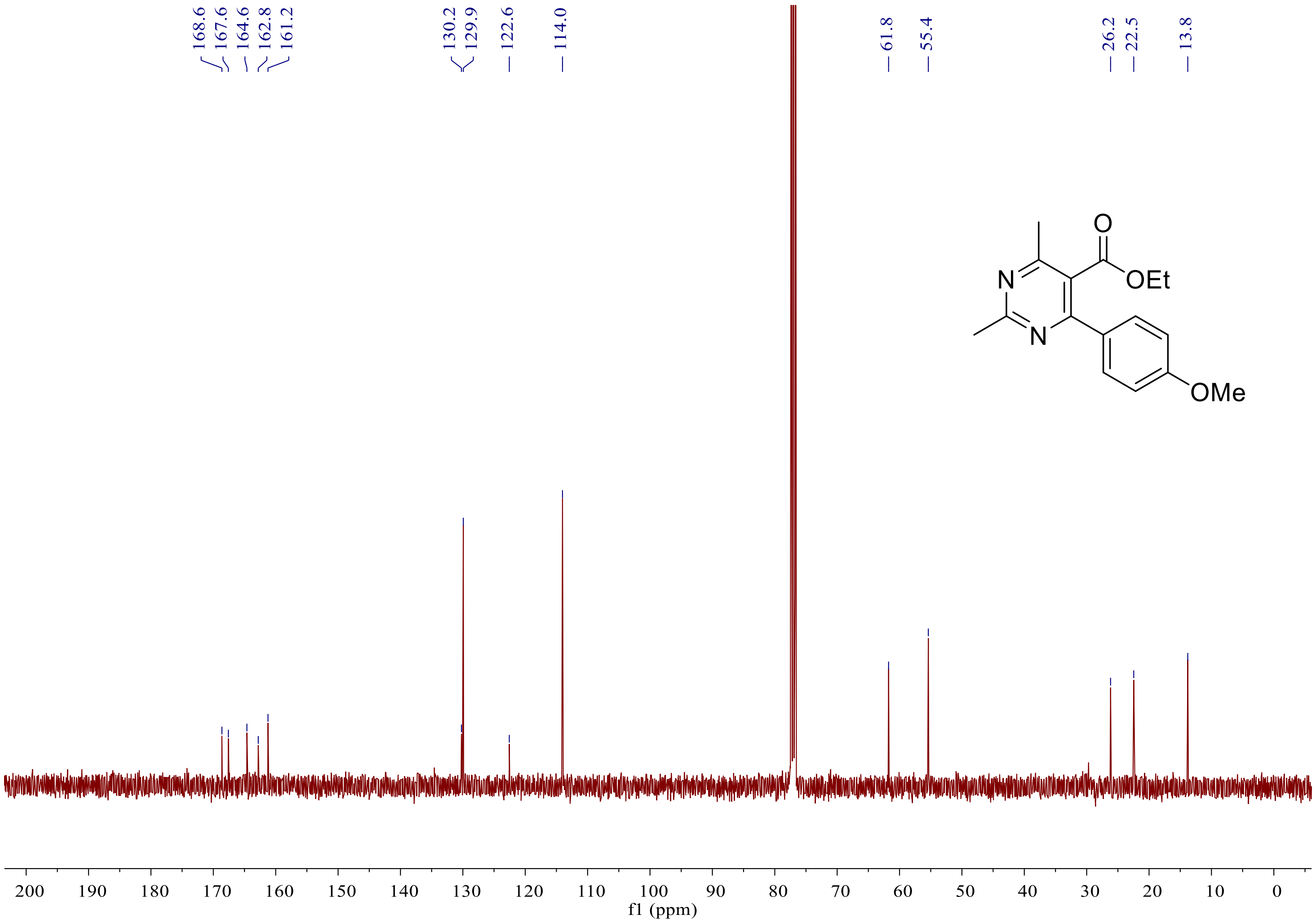

Supplementary Figure 165. ^{13}C NMR (100 MHz, $CDCl_3$) of **2ar**

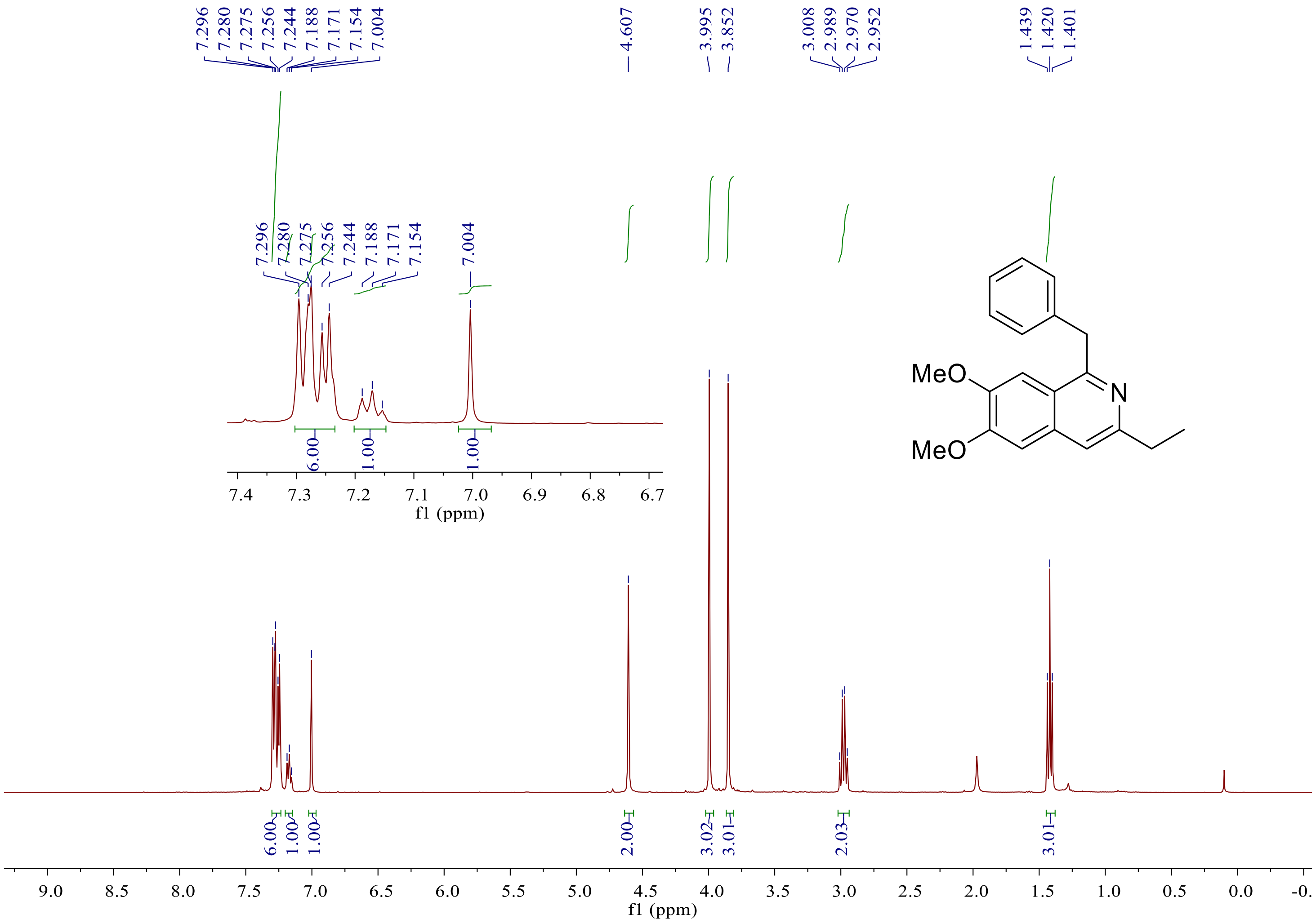

Supplementary Figure 166. ^{1}H NMR (400 MHz, $CDCl_3$) of **2as**

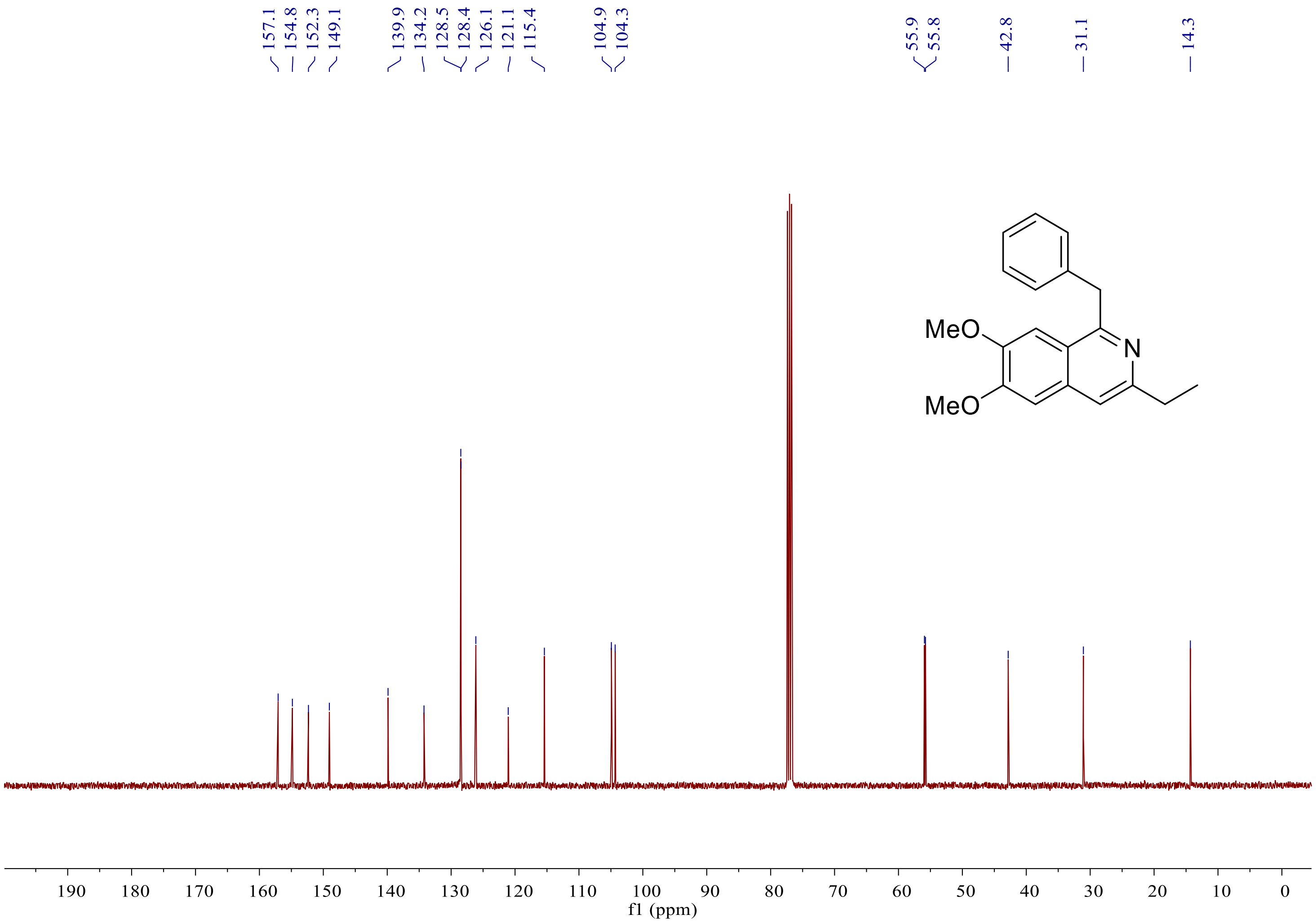

Supplementary Figure 167. ^{13}C NMR (100 MHz, $CDCl_3$) of **2as**

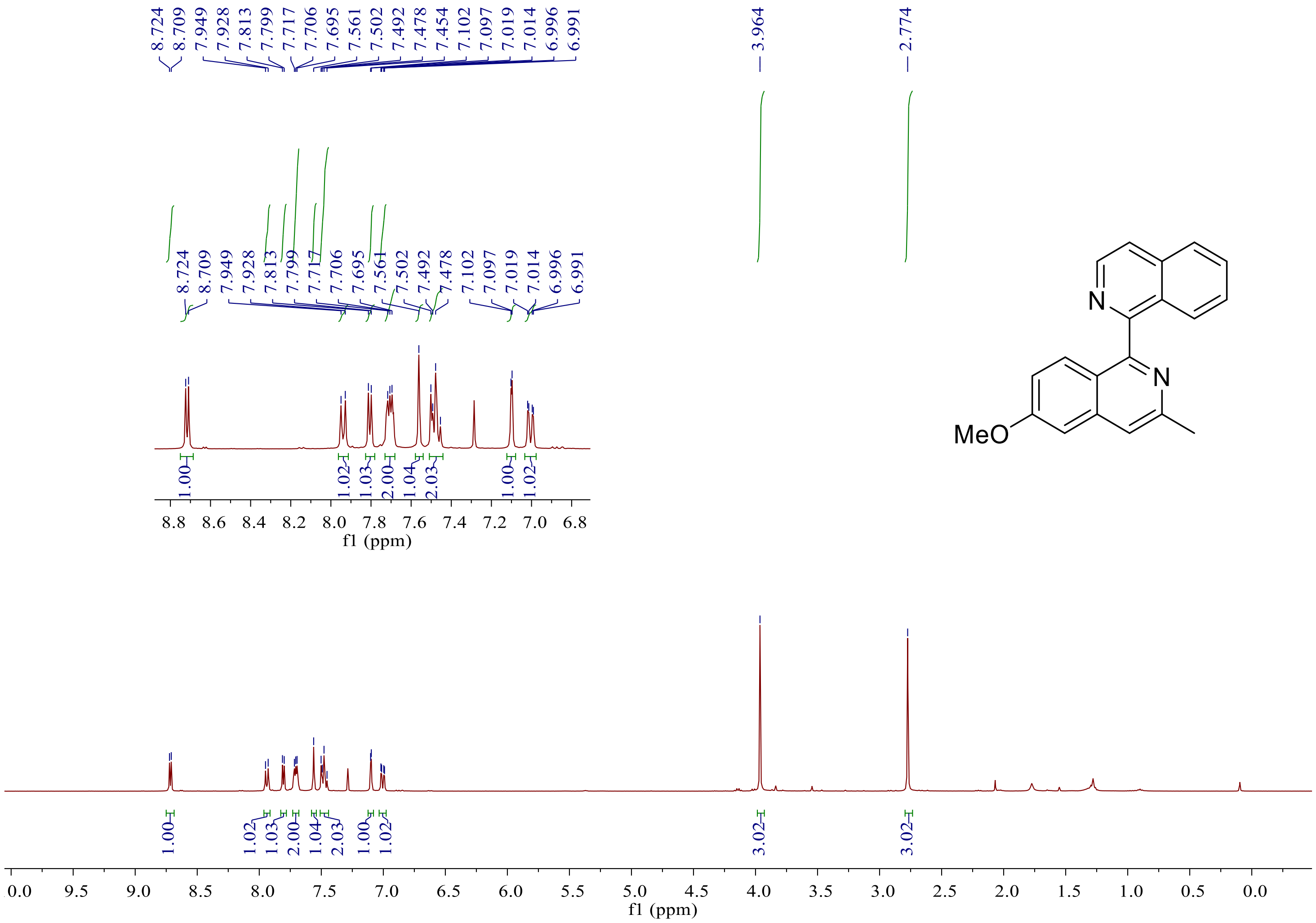

Supplementary Figure 168. ^{1}H NMR (400 MHz, $CDCl_3$) of **2at**

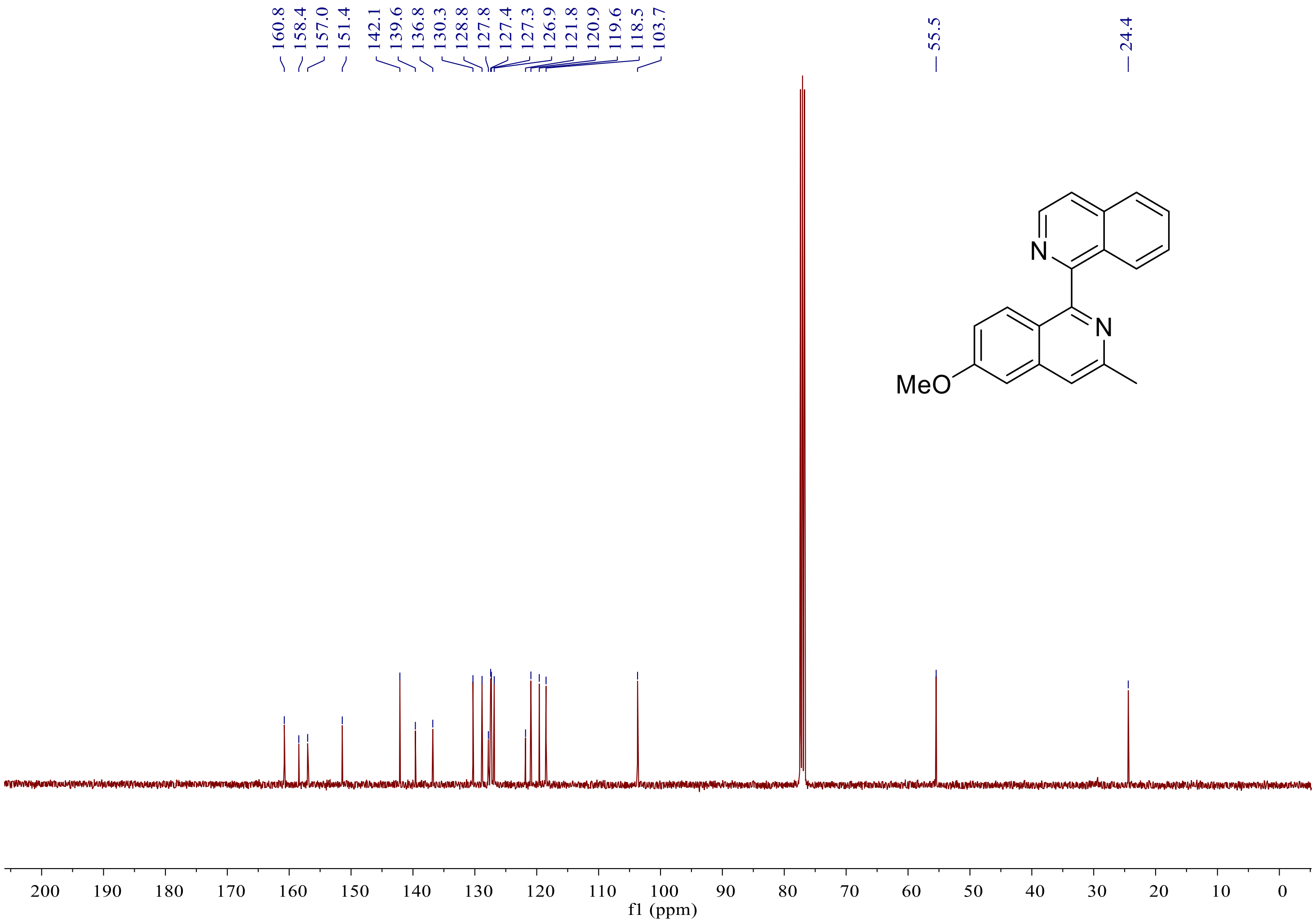

Supplementary Figure 169. ^{13}C NMR (100 MHz, $CDCl_3$) of **2at**

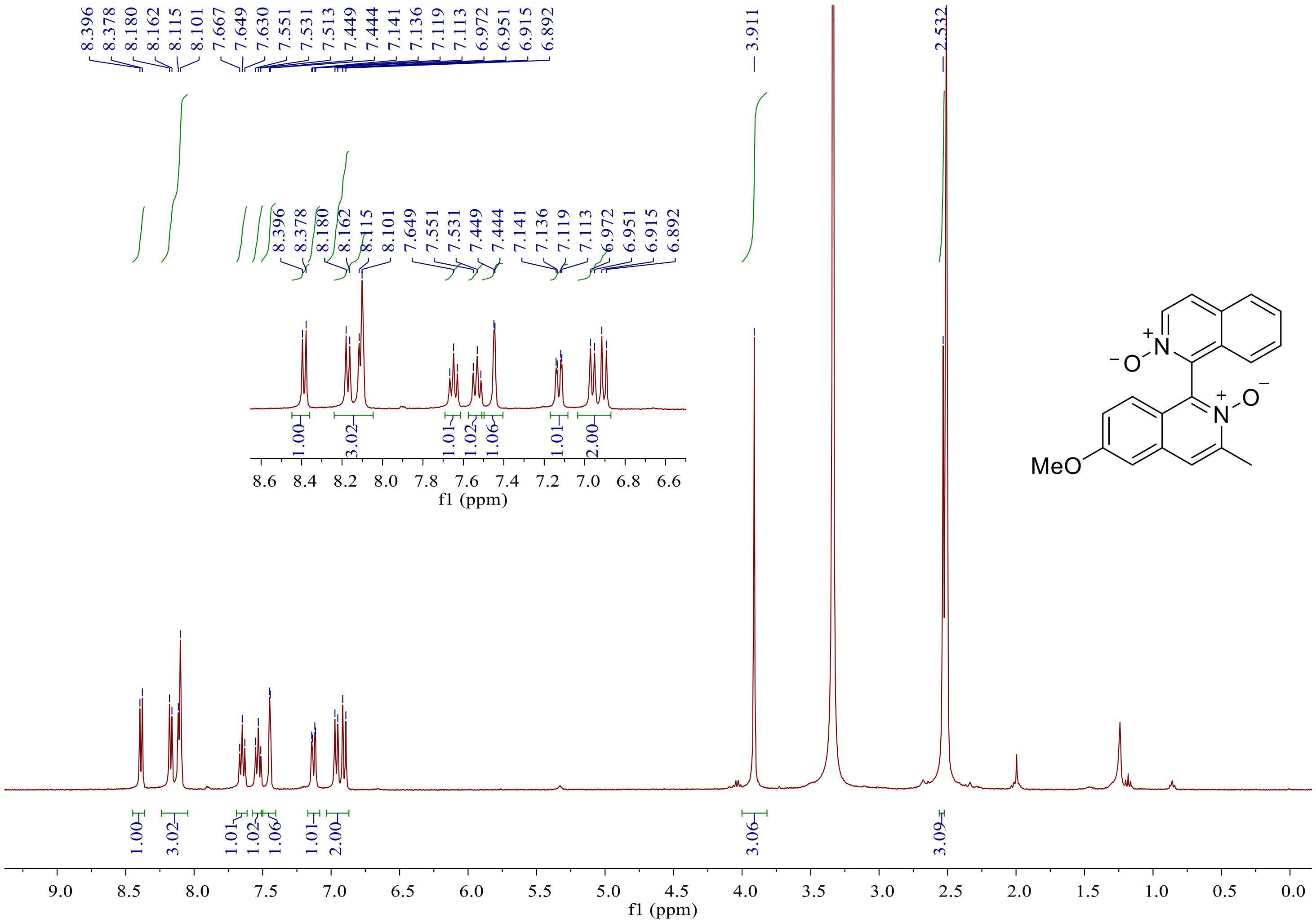

Supplementary Figure 170. ^{1}H NMR (400 MHz, DMSO-d_6) of **8**

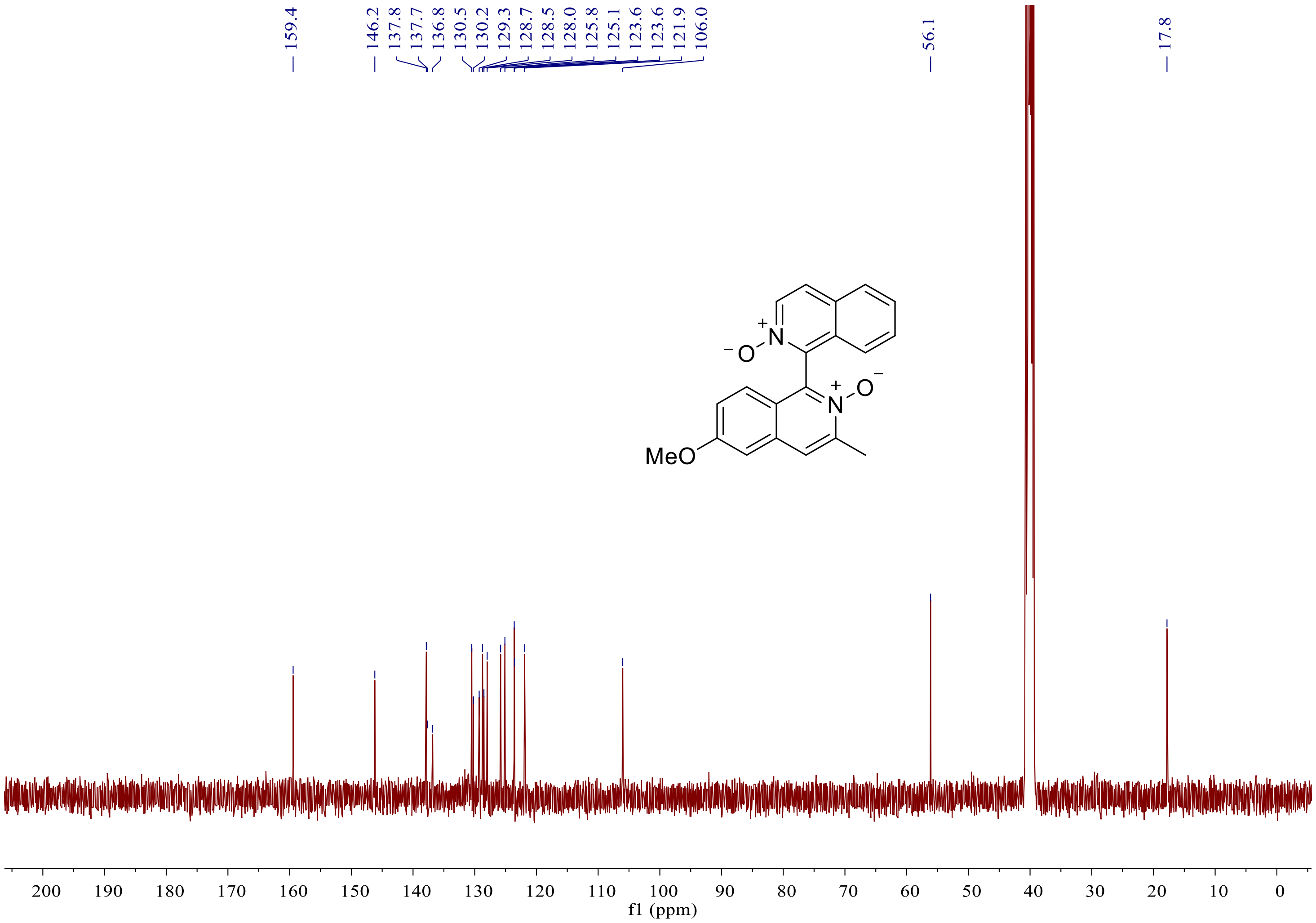

Supplementary Figure 171. ^{13}C NMR (100 MHz, DMSO-d_6) of **8**

14. Substrates giving complex mixtures

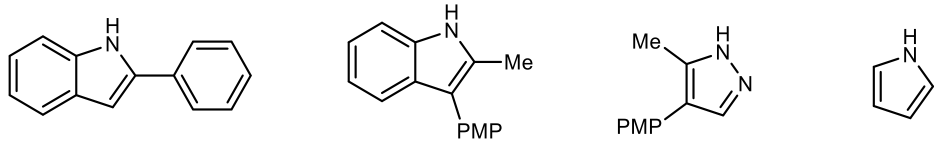

Supplementary Figure 172. Failed Substrates

Supplementary References

1 Liu, S. *et al.* Electrochemical Aziridination of Tetrasubstituted Alkenes with Ammonia. *CCS Chem.* **3**, 872-882, doi:10.31635/ccschem.021.202100826 (2021).

2 Rousseau, G., Le Perchec, P. & Conia, J. M. Petits cycles—XL: Sur le mecanisme de formation des hydroperoxydes allyliques dans la reaction de l'oxygene singulet avec les vinylcyclopropanes. *Tetrahedron* **34**, 3483-3494, doi: 10.1016/0040-4020(78)80236-1 (1978).

3 Newcomb, M., Choi, S.-Y. & Horner, J. H. Adjusting the Top End of the Alkyl Radical Kinetic Scale. Laser Flash Photolysis Calibrations of Fast Radical Clocks and Rate Constants for Reactions of Benzeneselenol. *J. Org. Chem.* **64**, 1225-1231, doi:10.1021/jo981930s (1999).

4 Zavitsas, A. A. & Chatgilialoglu, C. Energies of Activation. The Paradigm of Hydrogen Abstractions by Radicals. *J. Am. Chem. Soc.* **117**, 10645-10654, doi:10.1021/ja00148a006 (1995).

5 Jat, J. L. *et al.* Direct Stereospecific Synthesis of Unprotected N-H and N-Me Aziridines from Olefins. *Science* **343**, 61-65, doi:10.1126/science.1245727 (2014).

6 Jolit, A., Vazquez-Rodriguez, S., Yap, G. P. A. & Tius, M. A. Diastereospecific Nazarov Cyclization of Fully Substituted Dienones: Generation of Vicinal All-Carbon-Atom Quaternary Stereocenters. *Angew. Chem. Int. Ed.* **52**, 11102-11105, doi:10.1002/anie.201305218 (2013).

7 Troutman, M. V., Appella, D. H. & Buchwald, S. L. Asymmetric Hydrogenation of Unfunctionalized Tetrasubstituted Olefins with a Cationic Zirconocene Catalyst. *J. Am. Chem. Soc.* **121**, 4916-4917, doi:10.1021/ja990535w (1999).

8 Nikitin, K., Müller-Bunz, H., Ortin, Y. & McGlinchey, M. J. Joining the rings: the preparation of 2- and 3-indenyl-triptycenes, and curious related processes. *Org. Biomol. Chem.* **5**, 1952-1960, doi:10.1039/B703437G (2007).

9 Zhang, X. *et al.* Synthesis of highly substituted indene derivatives by Brønsted acid catalyzed Friedel–Crafts reaction of homoallylic alcohols. *Tetrahedron Lett.* **55**, 3881-3884, doi: 10.1016/j.tetlet.2014.05.027 (2014).

10 Gui, J. *et al.* Brønsted acid/visible-light-promoted Markovnikov hydroamination of vinylarenes with arylamines. *Org. Biomol. Chem.* **18**, 956-963, doi:10.1039/C9OB02457C (2020).

11 Domaradzki, M. E. *et al.* Gold-Catalyzed Ammonium Acetate Assisted Cascade Cyclization of 2-Alkynylarylketones. *J. Org. Chem.* **80**, 11360-11368, doi:10.1021/acs.joc.5b01939 (2015).

12 Reimann, S. *et al.* Site Selective Synthesis of Pentaarylpyridines via Multiple Suzuki–Miyaura Cross-Coupling Reactions. *Adv. Synth. Catal.* **356**, 1987-2008, doi: 10.1002/adsc.201400164 (2014).

13 Liu, X., Liu, C. & Cheng, X. Correction: Ring-contraction of hantzsch esters and their derivatives to pyrroles via electrochemical extrusion of ethyl acetate out of aromatic rings. *Green Chem.* **23**, 4620-4620, doi:10.1039/D1GC90054D (2021).

14 Chen, R., Qi, J., Mao, Z. & Cui, S. Rh(iii)-catalyzed C–H activation/cyclization of oximes with alkenes for regioselective synthesis of isoquinolines. *Org. Biomol. Chem.* **14**, 6201-6204, doi:10.1039/C6OB00942E (2016).

15 Reep, C., Morgante, P., Peverati, R. & Takenaka, N. Axial-Chiral Biisoquinoline N,N′-

Dioxides Bearing Polar Aromatic C-H Bonds as Catalysts in Sakurai-Hosomi-Denmark Allylation. *Org. Lett.* **20**, 5757-5761, doi:10.1021/acs.orglett.8b02457 (2018).
